# Synthesis of β-Carbolines with Electrocyclic Cyclization of 3-Nitrovinylindoles

**DOI:** 10.3390/ijms241713107

**Published:** 2023-08-23

**Authors:** Nicolai A. Aksenov, Nikolai A. Arutiunov, Alexander V. Aksenov, Nikita K. Kirilov, Inna V. Aksenova, Dmitrii A. Aksenov, Elena V. Aleksandrova, Michael Rubin, Alexander Kornienko

**Affiliations:** 1 Department of Chemistry, North Caucasus Federal University, 1a Pushkin St., Stavropol 355009, Russia; naarutiunov@ncfu.ru (N.A.A.); aaksenov@ncfu.ru (A.V.A.); lyncheron@gmail.com (N.K.K.); iaksenova@ncfu.ru (I.V.A.); daksenov@ncfu.ru (D.A.A.); elena.aleksandrova1607@gmail.com (E.V.A.); mrubin@ku.edu (M.R.); 2 Department of Chemistry and Biochemistry, Texas State University, 601 University Dr., San Marcos, TX 78666, USA

**Keywords:** beta-carboline, indole, electrocyclization, heterocycle, norharmane, harmane

## Abstract

The β-carboline motif is common in drug discovery and among numerous biologically active natural products. However, its synthetic preparation relies on multistep sequences and heavily depends on the type of substitution required in the core of the desired β-carboline target. Herein, we demonstrate that this structural motif can be accessed with the microwave-assisted electrocyclic cyclization of heterotrienic aci (alkylideneazinic acid) forms of 3-nitrovinylindoles. The reaction can start with 3-nitrovinylindoles themselves under two sets of conditions. The first one involves microwave irradiation of butanolic solutions of 3-nitrovinylindoles, whereas the second one consists of prior Boc protection of indolic nitrogen, where the protecting group cleanly comes off during the course of the reaction. Alternatively, the reaction can start with 3-nitrovinylindoles prepared in situ using various processes. Finally, the reaction may utilize indoles with β-nitrostyrenes, likely involving the intermediacy of spirocyclic oxazolines, which rearrange to similar heterotrienic systems undergoing cyclization to β-carbolines. As part of this study, several natural products, namely, alkaloids norharmane, harmane, and eudistomin N, were synthesized.

## 1. Introduction

**Figure 1:**
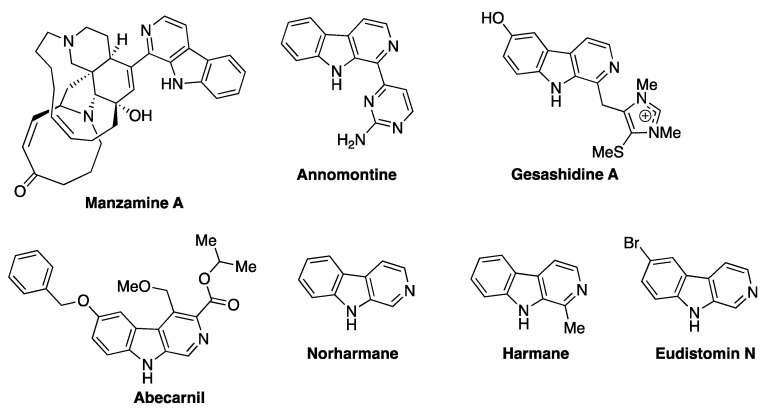
Structures of selected β-carboline-containing natural products and synthetic drugs.

The β-carboline heterocyclic core is a structural subunit of numerous natural products and thus highly prevalent in synthetic libraries of compounds utilized in drug discovery. Since it is a biological privileged structure [1,2], compounds containing this structural motif possess various biological activities. For example, marine alkaloid manzamine A has shown activity against Alzheimer’s disease, tropical parasites, HIV, and AIDS-related opportunistic infections [3,4,5,6,7] (Figure 1). Another marine alkaloid gesashidine was shown to possess antibiotic activity [8]. Annomontine, isolated from a South-American plant, was shown to have anti-leishmanial activity [9]. Abecarnil is a recently developed anxiolytic drug belonging to so-called nonbenzodiazepine family. It acts selectively at the benzodiazepine site of the GABA_A_ receptor but is based on the β-carboline core instead of the benzodiazepine one [10]. Structurally simpler β-carbolines, based on the β-carboline moiety with just one or two simple substituents, also commonly occur among natural and synthetic bioactive compounds. For example, harmane and norhamane are found in coffee, tobacco, and wild rue (*Peganum harmala*) [11,12]. They were shown to be potent tremor-inducing neurotoxins [13,14] and DNA-interacting genotoxins [15,16]. Eudistomin N, a member of a large family of marine alkaloids [17], was demonstrated to possess antibacterial and antiviral activities [18] (Figure 1).

**Figure 2:**
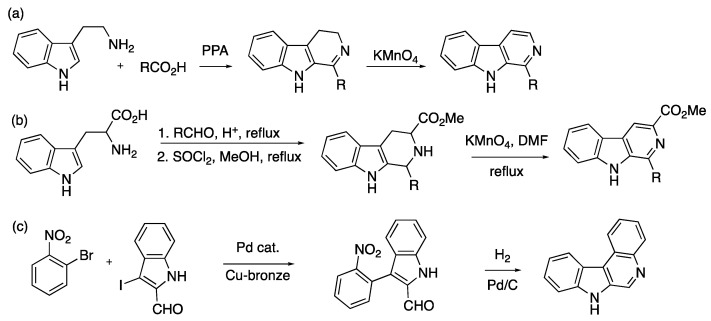
Common methods to synthesize the β-carboline scaffold. (**a**) Condensation of tryptamines with carboxylic acids in PPA followed by oxidation with KMnO4; (**b**) Pictet–Spengler reaction of tryptophan with aldehydes followed by oxidation with KMnO4 in refluxing DMF; (**c**) Pd-catalyzed-coupling with subsequent reduction of the nitro group followed by intramolecular cyclocondensation.

Common methods to obtain β-carboline-containing compounds involve the use of harsh reaction conditions, such as condensation of tryptamines with carboxylic acids in polyphosphoric acid (PPA) followed by oxidation with KMnO_4_ (Figure 2a) [19] or the Pictet–Spengler reaction of tryptophan with aldehydes followed by oxidation with KMnO_4_ in refluxing dimethyl formamide (DMF) (Figure 2b) [20]. Milder methods are generally quite laborious and multistep, requiring strategic placement of functional groups. For example, a method utilizing chemistry of the nitro group (Figure 2c) requires the placement of the nitro and the aldehyde groups into the correct positions and subsequent reduction of the nitro group followed by intramolecular cyclocondensation [21]. Novel methods to synthesize β-carbolines continue to draw significant interest within the synthetic community [22,23,24].

**Figure 3:**
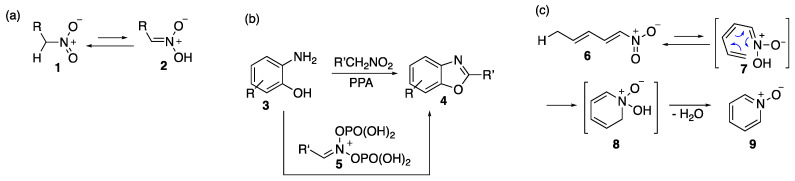
Previously studied chemistry of nitronates **2** (**a**,**b**) [25] and the proposed electrocyclization described in the present work (**c**).

Recent work in our laboratories has focused on studying the umpolung chemistry of nitroalkanes, specifically the activation of compounds **1** (Figure 3a) to nitronic acid forms **2**. This, for example, can be accomplished with PPA (Figure 3b) to give compounds such as **5**. Compound **5** can then be made to react with carbon, nitrogen, or oxygen nucleophiles [25,26,27,28,29,30]. The example shown in Figure 3b illustrates a reaction of aminophenol **3** with a nitroalkane in PPA (compound 5) to yield benzoxazole **4** [25]. We surmised that if the nitro group were to be conjugated with an unsaturated system, such as in **6** (Figure 3c), the formation of nitronate would lead to heterotriene **7**, capable of 6π electrocyclization to give, after the loss of water in **8**, pyridine oxide **9**. Furthermore, if one of the double bonds in **6** was part of an aromatic carbocyclic or heteroaromatic system, more complex fused polycyclic systems, such as β-carbolines, could arise.

**Figure 4:**
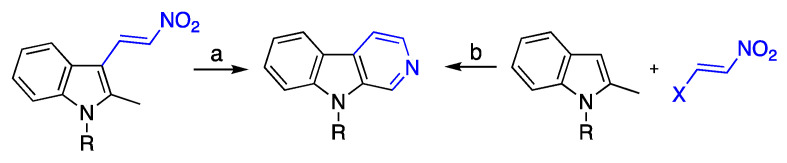
Two routes towards the β-carboline scaffold developed herein.

Herein, we would like to present an implementation of this strategy that resulted in the one-step synthesis of β-carbolines. This synthesis represents a new transformation involving 3-nitrovinylindoles (Figure 4). It can be initiated from 3-nitrovinylindoles themselves (route a), or the latter can be formed in situ with various processes (e.g., route b). The “route a” approach has been previously communicated by us [31].

## 2. Results and Discussion

**Figure 5:**
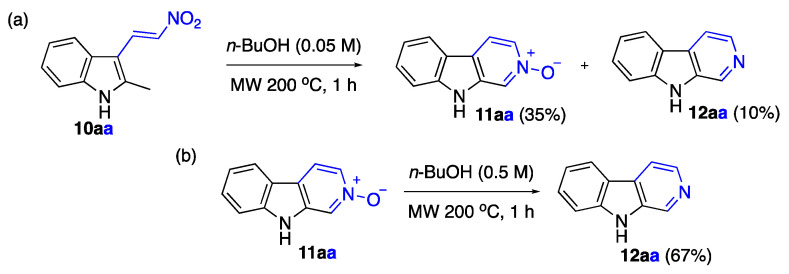
Initial experiment (**a**) and conversion of *N*-oxide to β-carboline under reaction conditions (**b**).

Our initial experimentation involved the reaction of (*E*)-2-methyl-3-(2-nitrovinyl)-1*H*-indole (**10aa**) in *n*-butanol using a microwave at 200 °C in a sealed pressure tube. After 1 h, the expected β-carboline *N*-oxide, **11aa**, was obtained in moderate yield (Figure 5). Unexpectedly, small amounts of β-carboline **12aa** were also isolated, indicating that the *N*-oxide underwent partial reduction under the reaction conditions. We undertook significant optimization efforts to make the method synthetically useful (Table 1).

**Figure 6:**
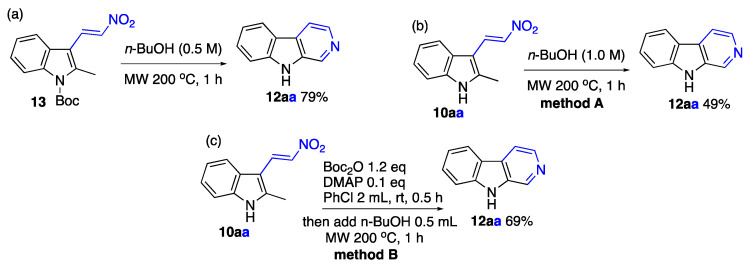
Reaction with Boc protection of indole nitrogen (**a**) and the two methods, A (**b**) and B (**c**), resulting from the optimization efforts.

Raising the temperature from 200 °C in the initial experiment involving the reaction in 1-butanol (entry 1) to 220 °C allowed us to shorten the reaction time from 1 h to 0.5 h, although the yield was slightly reduced (entry 2). The reaction proceeded sluggishly at temperatures below 200 °C. Next, the reaction was tried in ethylene glycol since this high-boiling solvent could potentially allow us to conduct the process at ambient pressure; however, this did not lead to improved yield (entry 3). To understand the role of solvent, the reaction was performed in undecane (entry 4), dimethyl sulfoxide (DMSO; entry 5), quinoline (entry 6), and molten pyridine•HCl (entry 7). In the first case, the reaction did not proceed at all, whereas the other solvents gave results comparable to those of the reaction in 1-butanol. The reaction in water (entry 8) or under neat conditions (entry 9) gave significantly lower yield, and we noticed that the starting material, under these conditions, underwent decomposition. This raised the question of what serves as a reducing agent for the transformation **11aa** → **12aa** (Figure 5b). We performed the reaction in the presence of reducing agents, such as P(OMe)_3_ (entry 10), Na_2_S_2_O_3_ (entry 11), SnCl_2_ (entry 12), Zn/NH_4_Cl (entry 13), all of which afforded poor results. We also attempted to use materials bearing functional groups that could have potentially served as more effective reducing moieties than those in the starting material, such as 2-methylindene (entry 14) and β-nitrostyrene (entry 15). This, however, did not lead to improved results. Thus, it appears that 1-butanol itself serves as the reducing agent in these reactions, which is also consistent with the detection of *n*-butanal in the analysis of crude reaction mixtures using GC/MS. Finally, we tested the idea of facilitating the tautomerization process involving C2-Me in starting material **10aa** by introducing electron-withdrawing groups on the nitrogen of the indole. We performed the reaction in the presence of acetic anhydride (Ac_2_O; entry 16), acetyl chloride (AcCl; entry 17), and tosyl chloride (TsCl; entry 18). This, however, did not result in improved reaction performance in contrast to the installation of the *tert*-butoxycarbonyl (Boc) protecting group, which raised the yield to 79% (Figure 6a). Thus, reaction optimization resulted in two practical procedures to conduct this transformation: method A, involving reaction with unprotected indole nitrogen (Figure 6b), and method B, which includes an extra step where the indole nitrogen is masked with the Boc group in a one-pot transformation (Figure 6c). It should be noted that the Boc protection is cleanly cleaved under the reaction conditions affording the same product, **12aa**.

**Figure 7:**
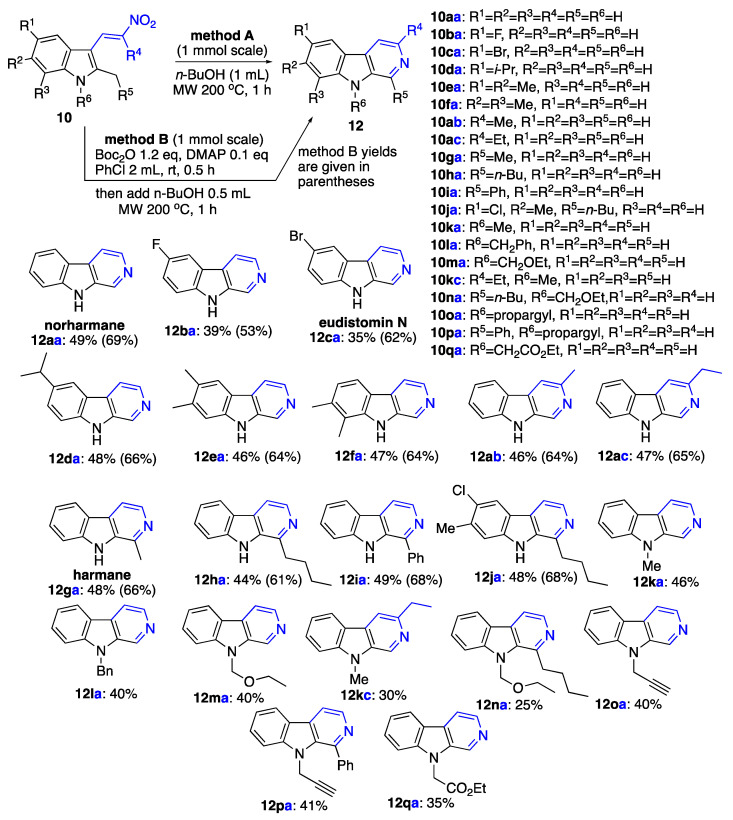
Preparative-scale synthesis of diversely substituted β-carbolines using methods A and B.

These optimized conditions allowed us to prepare a collection of β-carbolines with diverse substitution patterns on preparative scale using both methods A and B (Figure 7). Method B, involving the one-pot Boc protection of indole nitrogen and its subsequent deprotection under the reaction condition, consistently gave higher yield for this transformation. Among the β-carboline molecules synthesized using the developed methodology, three are natural products, alkaloids norharmane, harmane, and eudistomin N, whose significance and biological properties are discussed in the Introduction section. In order to accommodate the subsequent experiments, in which the nitroalkene came from a different molecule, we adopted a numbering system consisting of a number and two letters. One letter represents the type of substitution found on the indole, and the other letter represents the substitution pattern on the nitroalkene fragment. The nitroalkene fragment is also highlighted in blue color.

Next, we explored various methods involving the formation of 3-nitrovinylindoles in situ. For example, we explored the addition–elimination process of indoles with 1-(tertiary)amino-2-nitroethylenes (Table 2) [32,33].

**Figure 8:**
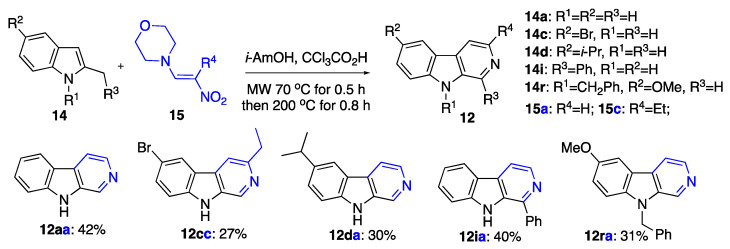
Preparative-scale synthesis of diversely substituted β-carbolines obtained with the reaction of indoles with 4-(2-nitrovinyl)morpholine.

Varying the reaction solvent (1-butanol, xylene, DMF, and isoamyl alcohol), the acid additive (CH_3_COOH, CCl_3_COOH, CF_3_COOH, NH_2_SO_3_H, MsOH), and the amino leaving group on 2-nitroethylenes **15** (4-morpholine and *N*,*N*-dimethylamine) allowed us to select the optimal combination of reagents shown as entry 6. The reaction gave the highest yield of 42% when it was conducted using a two-fold temperature regime, one involving a MW at 70 °C for 0.5 h, allowing for the addition–elimination process to proceed to form the intermediate 2-nitrovinylindole, and the other utilizing a MW at 200 °C for 0.8 h for the electrocyclic cyclization to take place, leading to the β-carboline skeleton. By utilizing these reaction conditions, diversely substituted β-carbolines on preparative scale were synthesized with variations in the substituents in each of the three rings of the β-carboline skeleton (Figure 8).

**Figure 9:**
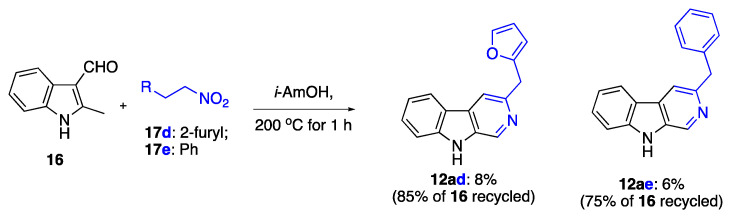
Attempted synthesis of β-carbolines using the Henry reaction of indole-3-carbaldehydes.

We also attempted to conduct this novel transformation using the Henry reaction as a method for the in situ formation of 2-nitrovinylindoles. To this end, 2-methyl-indole-3-carbaldehyde (**16**) was made to react with saturated nitro compounds **17** in isoamyl alcohol (Figure 9). The desired β-carboline products were isolated, albeit in low yield, with the majority of the reaction mixture containing unreacted starting carbaldehyde **16**.

**Figure 10:**
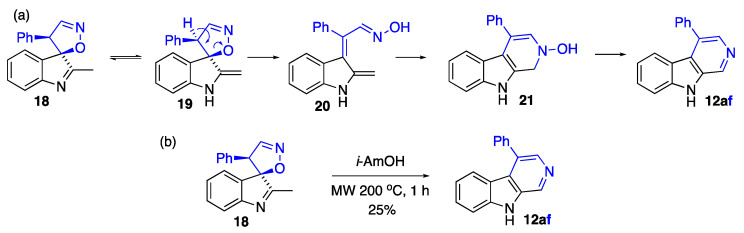
Mechanistic proposal for the conversion of spiro[indole-3,5′-isooxazoles] (18) to β-carbolines (**a**) and the experiment corroborating this hypothesis (**b**).

We have previously discovered a formal [4+1]-spirocyclizaton of indoles with nitroalkenes leading to spiro[indole-3,5′-isooxazoles] with complete diastereoselectivity (**18** in Figure 10) [34]. We speculated that following the mechanistic rationale shown in Figure 10a, these compounds could also transform into β-carbolines via an analogous electrocyclization process. Indeed, under the usual the reaction conditions involving heating in a microwave oven at 200 °C for 1 h in isoamyl alcohol, we were able to convert spiro-isoxazole **18** to β-carboline **12af** (Figure 10b) in modest yield (25%).

**Figure 11:**
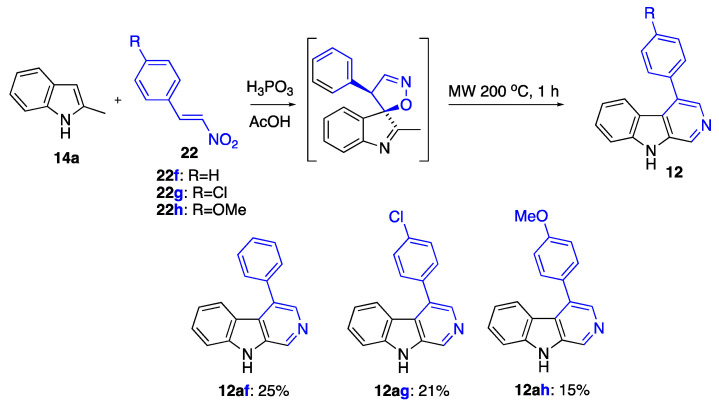
Synthesis of β-carbolines utilizing a methodology involving the diastereoselective formation of spiro[indole-3,5′-isooxazoles] [34] in situ.

We succeeded in demonstrating that this methodology can be conducted in one pot, starting with indoles and nitroalkenes with the formation of spiro-isoxazoles in situ, although the yield remained modest (Figure 11). So far, our optimization of this process has not been successful in improving the reaction yield.

## 3. Materials and Methods

### 3.1. General Information

^1^H and ^13^C{^1^H} NMR spectra were recorded on a Bruker Avance-III spectrometer (Bruker BioSpin AG, Fällanden, Switherland; 400 and 101 MHz, respectively) equipped with a broad-band observation (BBO) probe in CDCl_3_ or DMSO-*d*_6_ using tetramethylsilane (TMS) as an internal standard. High-resolution mass spectra were registered with a Bruker Maxis spectrometer (Bruker Daltonics GmbH & Co. KG Fahrenheitstraße 4, Bremen, Germany; electrospray ionization, in MeCN solution, using HCO_2_Na–HCO_2_H for calibration). IR spectra were measured with an FT-IR spectrometer Shimadzu IR Affinity-1S equipped with an attenuated total reflectance (ATR) sampling module (Shimadzu corporation, Nishinokyo Kuwabara-cho, Nakagyo-ku, Kyoto, Japan). Melting points were measured with a Stuart SMP30 apparatus (Stuart scientific Co. Ltd., Redhill, UK). MW-assisted reactions were conducted in G10 or G30 vials using an Anton Paar Monowave 300 reactor (Anton Paar GmbH, Graz, Austria; serial number 81552252), employing constant temperature mode with temperature control using an IR sensor (see part 9 for temperature–time, pressure–time, and power–time relationships). Warning! The pressure in the reactor due to the presence of volatile solvents and the release of CO_2_ in some experiments can reach 20 bar. Only certified pressure vials capable of withstanding such pressure should be used. The reaction progress and the purity of isolated compounds were controlled using thin layer chromatography (TLC) on ALUGRAM Xtra SIL G UV 254 plates. Column chromatography was performed with Macherey Nagel Silica gel 60 (particle size: 0.063–0.20 mm). (*E*)-5-bromo-2-methyl-3-(2-nitrovinyl)-1*H*-indole (**10ca**) [35] and *tert*-butyl (*E*)-2-methyl-3-(2-nitrovinyl)-1*H*-indole-1-carboxylate (**13**) [36] were synthesized according to published procedures, and their physical and spectral properties were identical to those described in the literature. All other reagents and solvents were purchased from commercial vendors and used as received. 

### 3.2. Preparation of Starting Materials

#### 3.2.1. Synthesis of Starting 4-Chloro-2-iodo-5-methylaniline (**28j**)

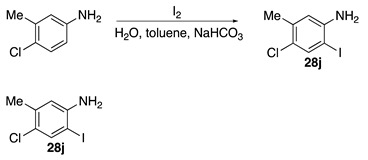



4-Chloro-2-iodo-5-methylaniline (**28j**): This compound was synthesized using a procedure originally reported for the modification of 4-chloroaniline [37]. A suspension of 4-chloro-3-methylaniline (141 mg, 1.00 mmol) in water (3 mL) and toluene (0.3 mL) magnetically stirred at 18 °C was treated with NaHCO_3_ (134 mg, 1.60 mmol) and iodine (203 mg, 0.80 mmol) over a period of 0.5 h. The reaction mixture was then poured into water (15 mL). The aqueous layer was extracted with ethyl acetate (2 × 10 mL), and the combined organic phases were washed with Na_2_S_2_O_3_ (2 × 5 mL of a 5% aqueous solution) and brine (1 × 5 mL), then dried (Na_2_SO_4_), and concentrated under reduced pressure. The resulting yellow oil was subjected to flash chromatography (EtOAc/hexane, 1:8, *v*/*v*). The titled compound was obtained as light-brown crystals, m.p. 56–58 °C (Hexane), lit [38] m.p. 65 °C, R*_f_* 0.80 (EtOAc/hexane, 1:4, *v*/*v*). Yield of 240 mg (0.90 mmol, 90%). ^1^H NMR (400 MHz, CDCl_3_) δ 7.47 (s, 1H), 6.75 (s, 1H), 3.99 (br. s, 2H), 2.21 (s, 3H); ^13^C{^1^H} NMR (101 MHz, CDCl_3_) δ 145.8, 140.2, 135.3, 127.5, 114.8, 81.6, 18.7; FTIR, *v_max_*: 3420, 3347, 2920, 2728, 1703, 1487, 1447, 1384, 1369, 1296, 1258, 1194, 1054 cm^−1^; HRMS (ESI TOF) *m*/*z* calc’d. for C_7_H_8_ClIN [M+H]^+^: 267.9385, found: 267.9387 (−0.8 ppm).

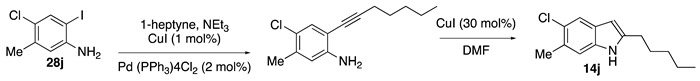



#### 3.2.2. Synthesis of Starting Indole (**14j**)

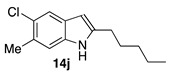



5-Chloro-6-methyl-2-pentyl-1*H*-indole (**14j**): This compound was prepared using a modified method, previously shown in the literature [39]. A suspension of 4-chloro-2-iodo-5-methylaniline (**28j**; 803 mg, 3.00 mmol), PdCl_2_(PPh_3_)_2_ (42.0 mg, 0.06 mmol, 2.0 mol%), and CuI (6.0 mg, 0.03 mmol, 1.0 mol%) in 15.0 mL triethylamine was degassed with argon and evacuated/backfilled with argon (3 cycles). The reaction mixture was stirred at 30 °C for 10 min. After the addition of the alkyne (3.6 mmol), the suspension was stirred for 24 h at 30 °C under argon atmosphere (progress of reaction monitored using TLC). The removal of the solvent was performed under reduced pressure, and the residue was used in next stage without purification. To the crude material, we added 10 mol. % (57 mg) CuI and 15 mL of DMF, and the mixture was kept at reflux under nitrogen for 10 h and then concentrated to dryness. The residue was taken up in ethyl acetate and filtered through Celite. The titled compound was purified using column chromatography (EtOAc/hexane, 1:6, *v*/*v*). The titled compound was obtained as colorless crystals, m.p. 91–92 °C, R*_f_* 0.69 (EtOAc/hexane, 1:3, *v*/*v*). Yield of 601 mg (2.55 mmol, 85%). ^1^H NMR (400 MHz, CDCl_3_) δ 7.76 (s, 1H), 7.34 (s, 1H), 7.29 (s, 1H), 6.13 (s, 1H), 2.71 (t, *J* = 7.6 Hz, 2H), 2.42 (s, 3H), 1.74–1.67 (m, 2H), 1.38–1.33 (m, 4H), 0.92–0.88 (m, 3H); ^13^C{^1^H} NMR (101 MHz, CDCl_3_) δ 141.0, 134.9, 128.0, 127.5, 126.9, 121.0, 110.6, 99.1, 31.6, 28.9, 28.4, 22.6, 20.4, 14.1; FTIR, *v_max_*: 3394, 2950, 1734, 1679, 1635, 1555, 1520, 1505, 1472, 1459, 1376, 1298 cm^−1^; HRMS (ESI TOF) *m*/*z* calc’d. for C_14_H_17_ClN [M-H]^−^: 234.1055, found: 234.1059 (−1.6 ppm).

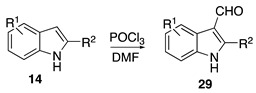



#### 3.2.3. Synthesis of Starting 1H-indole-3-carbaldehydes (**29**)

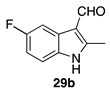



5-Fluoro-2-methyl-1*H*-indole-3-carbaldehyde (**29b**), Typical Procedure C for preparation of 1*H*-indole-3-carboxaldehydes (**29**): The procedure was adapted from the previously described synthetic protocol [40]. POCl_3_ (0.35 mL, 3.7 mmol) was added dropwise into stirred and ice-cooled DMF (1.0 mL). The reaction mixture was stirred at 1–5 °C for 20 min; then, a solution of 5-fluoro-2-methyl-1*H*-indole (**14b**) (462 mg, 3.10 mmol) in DMF (1.0 mL) was slowly added. The resulting reaction mixture was slowly warmed to 35 °C and kept at this temperature for 40 min. It was then allowed to cool down to room temperature. To this mixture, ice (~10 g) was added, followed by 5 M NaOH solution (6 mL, 31.00 mmol). The reaction mixture was heated at 95 °C for 30 min; then, it was allowed to cool down to room temperature. To this mixture, ice (~10 g) was again added, and the resulting reaction mixture was stirred for 30 min. The desired product was collected by filtrating it and washed several times with water. The titled compound was purified using column chromatography (EtOAc/hexane, 1:2, *v*/*v*). The titled compound was obtained as colorless solid, m.p. 217.6–219.9 °C (EtOAc), lit [40]. m.p. 176–173 °C, R*_f_* 0.64 (EtOAc/hexane, 1:1). Yield of 472 mg (2.67 mmol, 86%). ^1^H NMR (400 MHz, DMSO-*d_6_*) δ 12.11 (br. s, 1H), 10.02 (s, 1H), 7.71 (dt, *J* = 9.8, 1.8 Hz, 1H), 7.39 (ddd, *J* = 8.7, 4.5, 1.2 Hz, 1H), 7.06–6.97 (m, 1H), 2.67 (s, 3H); ^13^C{^1^H} NMR (101 MHz, DMSO-*d_6_*) δ 184.4, 158.7 (d, *J* = 234.7 Hz), 150.0, 132.0, 126.3 (d, *J* = 11.0 Hz), 113.8 (d, *J* = 4.3 Hz), 112.6 (d, *J* = 9.9 Hz), 110.5 (d, *J* = 25.7 Hz), 105.1 (d, *J* = 24.4 Hz), 11.6; ^19^F NMR (376 MHz, DMSO-*d*_6_) δ −121.59; FTIR, *v_max_*: 3265, 1620, 1585, 1481, 1454, 1383, 1255, 1189, 1176, 1121, 1099 cm^−1^; HRMS (ESI TOF) *m*/*z*: calc’d for C_10_H_8_FNNaO [M+Na]^+^: 200.0482, found 200.0486 (−2.0 ppm).

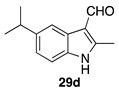



5-Isopropyl-2-methyl-1H-indole-3-carbaldehyde (**29d**)**:** This compound was prepared using Typical Procedure C employing 2-methyl-5-isopropyl-1*H*-indole (**14d**) (536 mg, 3.10 mmol). The titled compound was purified using column chromatography (EtOAc/hexane, 1:2, *v*/*v*). The titled compound was obtained as colorless solid, m.p. 182.4–183.7 °C (EtOH), R*_f_* 0.37 (EtOAc/hexane, 1:1). Yield 554 mg (2.76 mmol, 89%). ^1^H NMR (400 MHz, DMSO-*d_6_*) δ 11.88 (s, 1H), 10.02 (s, 1H), 7.90 (s, 1H), 7.28 (d, *J* = 8.3 Hz, 1H), 7.06 (d, *J* = 8.3 Hz, 1H), 3.04–2.89 (m, 1H), 2.66 (s, 3H), 1.24 (s, 3H), 1.22 (s, 3H); ^13^C{^1^H} NMR (101 MHz, DMSO-*d_6_*) δ 184.2, 148.6, 142.2, 133.9, 125.8, 121.6, 117.1, 113.6, 111.1, 33.7, 24.6 (2C), 11.5; FTIR, *v_max_*: 3278, 2960, 1635, 1622, 1584, 1479, 1459, 1373, 1245, 1202 cm^−1^; HRMS (ESI TOF) *m*/*z*: calc’d for C_13_H_15_NNaO [M+Na]^+^: 224.1046, found 224.1040 (2.7 ppm).

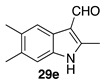



2,5,6-Trimethyl-1*H*-indole-3-carbaldehyde (**29e**): This compound was prepared using Typical Procedure C employing 2,5,6-trimethyl-1*H*-indole (14e) (493 mg, 3.10 mmol), The titled compound was purified using column chromatography (EtOAc/hexane, 1:3, *v*/*v*). The titled compound was obtained as colorless solid, m.p. 263.5–265.5 °C (EtOH), R*_f_* 0.24 (EtOAc/hexane, 1:1, *v*/*v*). Yield 510 mg (2.72.00 mmol, 88%). ^1^H NMR (400 MHz, DMSO-*d_6_*) δ 11.75 (s, 1H), 9.98 (s, 1H), 7.80 (s, 1H), 7.14 (s, 1H), 2.64 (s, 3H), 2.28 (s, 6H); ^13^C{^1^H} NMR (101 MHz, DMSO-*d_6_*) δ 183.9, 147.8, 134.2, 131.1, 130.1, 123.9, 120.4, 113.4, 111.7, 20.1, 19.9, 11.5; FTIR, *v_max_*: 3209, 2922, 1618, 1467, 1370, 1272, 1183, 1085 cm^−1^; HRMS (ESI TOF) *m*/*z*: calc’d for C_12_H_13_NNaO [M+Na]^+^: 210.0889, found 210.0888 (0.8 ppm). 

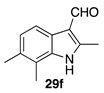



2,6,7-Trimethyl-1*H*-indole-3-carbaldehyde (**29f**): This compound was prepared using Typical Procedure C employing 2,6,7-trimethyl-1*H*-indole (14f) (493 mg, 3.10 mmol). The titled compound was purified using column chromatography (EtOAc/hexane, 1:3, *v*/*v*). The titled compound was obtained as colorless solid, m.p. 239.4–240.3 °C (EtOH), R*_f_* 0.25 (EtOAc/hexane, 1:2). Yield 499 mg (2.67 mmol, 86%) ^1^H NMR (400 MHz, DMSO-*d_6_*) δ 11.95 (s, 1H), 10.13 (s, 1H), 7.10 (d, *J* = 8.1 Hz, 1H), 6.98 (d, *J* = 8.1 Hz, 1H), 2.66 (s, 3H), 2.65 (s, 3H), 2.30 (s, 3H); ^13^C{^1^H} NMR (101 MHz, DMSO-*d_6_*) δ 184.4, 148.6, 134.4, 129.1, 128.2, 125.7, 125.0, 114.6, 108.8, 20.0, 18.3, 13.2; FTIR, *v_max_*: 3086, 2863, 1614, 1486, 1439, 1379, 1292, 1141, 1053 cm^−1^; HRMS (ESI TOF) *m*/*z*: calc’d for C_12_H_13_NNaO [M+Na]^+^: 210.0889, found 210.0888 (0.7 ppm).

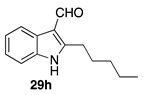



2-Pentyl-1*H*-indole-3-carbaldehyde (**29h**): This compound was prepared using Typical Procedure C employing 2-pentyl-1*H*-indole (**14h**) (580 mg, 3.10 mmol). The titled compound was purified using column chromatography (EtOAc/hexane, 1:4, *v*/*v*). The titled compound was obtained as colorless solid, m.p. 119–120 °C, R*_f_* 0.38 (EtOAc/hexane, 1:3, *v*/*v*). Yield 660 mg (3.07 mmol, 99%). ^1^H NMR (400 MHz, CDCl_3_) δ 10.19 (s, 1H), 9.75 (s, 1H), 8.33–8.24 (m, 1H), 7.42–7.37 (m, 1H), 7.29–7.24 (m, 2H), 3.10 (t, *J* = 7.7 Hz, 2H), 1.80 (q, *J* = 7.4 Hz, 2H), 1.39–1.31 (m, 4H), 0.88 (t, *J* = 7.0 Hz, 3H); ^13^C{^1^H} NMR (101 MHz, CDCl_3_) δ 184.8, 152.8, 135.5, 126.1, 123.6, 122.9, 121.1, 114.4, 111.3, 31.5, 30.0, 26.5, 22.5, 14.0; FTIR, *v_max_*: 3182, 2937, 2864, 1927, 1683, 1618, 1577, 1454, 1371, 1225 cm^−1^; HRMS (ESI TOF) *m*/*z* calc’d. for C_14_H_17_NNaO [M+Na]^+^: 238.1202, found: 238.1201 (0.7 ppm).

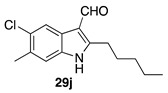



5-Chloro-6-methyl-2-pentyl-1*H*-indole-3-carbaldehyde (**29j**): This compound was prepared using Typical Procedure C employing 5-chloro-6-methyl-2-pentyl-1*H*-indole (**14j**) (729 mg, 3.10 mmol). The titled compound was purified using column chromatography (EtOAc/hexane, 1:4, *v*/*v*). The titled compound was obtained as colorless solid, m.p. 210–212 °C, R*_f_* 0.46 (EtOAc/hexane, 1:3, *v*/*v*). Yield of 774 mg (2.95 mmol, 95%). ^1^H NMR (400 MHz, DMSO-*d_6_*) δ 11.95 (br. s, 1H), 10.02 (s, 1H), 7.99 (s, 1H), 7.43 (s, 1H), 3.03 (t, *J* = 7.4 Hz, 2H), 2.38 (s, 3H), 1.75–1.65 (m, 2H), 1.33–1.24 (m, 4H), 0.84 (t, *J* = 6.3 Hz, 3H); ^13^C{^1^H} NMR (101 MHz, DMSO-*d_6_*) δ 184.2, 153.6, 134.6, 128.6, 128.0, 124.7, 121.8, 113.0, 111.6, 30., 29.4, 25.4, 21.9, 20.1, 13.9; FTIR, *v_max_*: 3092, 2808, 1922, 1734, 1681, 1656, 1623, 1558, 1505, 1454, 1373, 1237 cm^−1^; HRMS (ESI TOF) *m*/*z* calc’d. for C_15_H_18_ClNNaO [M+Na]^+^: 286.0969, found: 286.0969 (0.1 ppm).

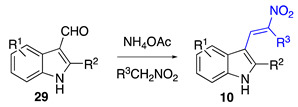



#### 3.2.4. Synthesis of Starting 3-(2-nitrovinyl)-1H-indoles (**10**)

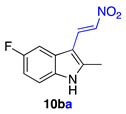



(*E*)-5-Fluoro-2-methyl-3-(2-nitrovinyl)-1*H*-indole (10ba), Typical Procedure D for preparation of 3-(2-nitrovinyl)-1*H*-indoles (**10**): Solid ammonium acetate (93 mg, 1.20 mmol) was added to a suspension of 5-fluoro-2-methyl- 1*H*-indole-3-carbaldehyde (**29b**) (354 mg, 2.00 mmol) in nitromethane (1 mL). The mixture was vigorously stirred at reflux for 6 h. Then, the mixture was cooled in an ice bath, and the solid that appeared was filtered and purified using column chromatography (EtOAc/hexane, 1:2, *v*/*v*). The titled compound was obtained as red solid, m.p. 214.9–216.5 °C (EtOH), R*_f_* 0.83 (EtOAc/hexane, 1:1). Yield of 405 mg (1.84 mmol, 92%). ^1^H NMR (400 MHz, DMSO-*d_6_*) δ 12.32 (br. s, 1H), 8.27 (d, *J* = 13.2 Hz, 1H), 7.93 (d, *J* = 13.3 Hz, 1H), 7.72 (dd, *J* = 10.2, 2.5 Hz, 1H), 7.40 (dd, *J* = 8.8, 4.6 Hz, 1H), 7.04 (td, *J* = 9.1, 2.4 Hz, 1H), 2.59 (s, 3H); ^13^C{^1^H} NMR (101 MHz, DMSO-*d_6_*) δ 158.7 (d, *J* = 234.8 Hz), 149.0 (2C), 133.0, 130.3, 125.9 (d, *J* = 10.5 Hz), 113.0 (d, *J* = 9.9 Hz), 110.6 (d, *J* = 25.6 Hz), 106.0 (d, *J* = 24.9 Hz), 105.3 (d, *J* = 4.0 Hz), 12.1; ^19^F NMR (376 MHz, DMSO-*d*_6_) δ -120.95; FTIR, *v_max_*: 3275, 1603, 1466, 1310,1250, 1226, 1168cm^−1^; HRMS (ESI TOF) *m*/*z*: calc’d for C_11_H_9_FN_2_NaO_2_ [M+Na]^+^: 243.0540, found 243.0537 (1.5 ppm).

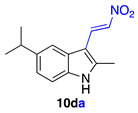



(*E*)-5-Isopropyl-2-methyl-3-(2-nitrovinyl)-1*H*-indole (**10da**): This compound was prepared using Typical Procedure D employing 2-methyl-5-isopropyl- 1*H*-indole-3-carbaldehyde (**29d**) (402 mg, 2.00 mmol) and nitromethane (1 mL). The titled compound was purified using column chromatography (EtOAc/hexane, 1:2, *v*/*v*). The titled compound was obtained as red solid, m.p. 225.3–227.0 °C (EtOH), R*_f_* 0.51 (EtOAc/hexane, 1:1). Yield of 459 mg (1.88 mmol, 94%). ^1^H NMR (400 MHz, DMSO-*d_6_*) δ 12.17 (s, 1H), 8.30 (d, *J* = 13.1 Hz, 1H), 7.93 (d, *J* = 13.2 Hz, 1H), 7.64 (s, 1H), 7.32 (d, *J* = 8.3 Hz, 1H), 7.19–7.02 (m, 1H), 3.14–2.96 (m, 1H), 2.59 (s, 3H), 1.27 (s, 3H), 1.26 (s, 3H); ^13^C{^1^H} NMR (101 MHz, DMSO-*d_6_*) δ 147.9, 142.6, 135.0, 133.5, 129.4, 125.5, 121.5, 117.7, 111.8, 105.3, 33.7, 24.6 (2C), 12.0; FTIR, *v_max_*: 3199, 2960, 1598, 1580, 1476, 1299, 1272, 1237, 1183, 1096 cm^−1^; HRMS (ESI TOF) *m*/*z*: calc’d for C_14_H_16_N_2_NaO_2_ [M+Na]^+^: 267.1104, found 267.1101 (1.0 ppm). 

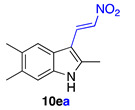



(*E*)-2,5,6-Trimethyl-3-(2-nitrovinyl)-1*H*-indole (**10ea**): This compound was prepared using Typical Procedure D employing 2,5,6-trimethyl-1*H*-indole-3-carbaldehyde (**29e**) (374 mg, 2.00 mmol) and nitromethane (1 mL). The titled compound was purified using column chromatography (EtOAc/hexane, 1:2, *v*/*v*). The titled compound was obtained as red solid, m.p. 228.9–230.5 °C (EtOH), R*_f_* 0.54 (EtOAc/hexane, 1:1). Yield of 419 mg (1.82.00 mmol, 91%) ^1^H NMR (400 MHz, DMSO-*d_6_*) δ 12.05 (s, 1H), 8.26 (d, *J* = 13.1 Hz, 1H), 7.90 (d, *J* = 13.0 Hz, 1H), 7.62 (s, 1H), 7.17 (s, 1H), 2.56 (s, 3H), 2.33 (s, 3H), 2.29 (s, 3H); ^13^C{^1^H} NMR (101 MHz, DMSO-*d_6_*) δ 147.4, 135.3, 133.6, 131.6, 130.6, 129.0, 123.5, 120.6, 112.3, 105.2, 19.9, 19.7, 11.8; FTIR, *v_max_*: 3273, 2918, 1743, 1598, 1572, 1476, 1298, 1226, 1171, 1077 cm^−1^; HRMS (ESI TOF) *m*/*z*: calc’d for C_13_H_14_N_2_NaO_2_ [M+Na]^+^: 253.0947, found 253.0942 (2.2 ppm). 

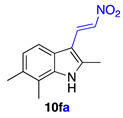



(*E*)-2,6,7-Trimethyl-3-(2-nitrovinyl)-1*H*-indole (**10fa**): This compound was prepared using Typical Procedure D employing 2,6,7-trimethyl-1*H*-indole- 3-carbaldehyde (**29f**) (374 mg, 2.00 mmol) and nitromethane (1 mL). The titled compound was purified using column chromatography (EtOAc/hexane, 1:4, *v*/*v*). The titled compound was obtained as red solid, m.p. 219.8–221.0 °C (EtOH), R*_f_* 0.25 (EtOAc/hexane, 1:2). Yield of 427 mg (1.86 mmol, 93%) ^1^H NMR (400 MHz, DMSO-*d_6_*) δ 12.14 (s, 1H), 8.77 (d, *J* = 13.2 Hz, 1H), 7.51 (d, *J* = 13.2 Hz, 1H), 7.13 (d, *J* = 8.1 Hz, 1H), 6.99 (d, *J* = 8.1 Hz, 1H), 2.58 (s, 3H), 2.55 (s, 3H), 2.34 (s, 3H); ^13^C{^1^H} NMR (101 MHz, DMSO-*d_6_*) δ 143.8, 135.7, 134.4, 130.7, 129.1, 127.1, 126.5, 125.1, 109.3, 105.4, 19.8, 16.3, 15.2; FTIR, *v_max_*: 3209, 1602, 1584, 1461, 1421, 1185, 1041 cm^−1^; HRMS (ESI TOF) *m*/*z*: calc’d for C_13_H_14_N_2_Na_1_O_2_ [M+Na]^+^: 253.0947, found 253.0945 (1.0 ppm). 

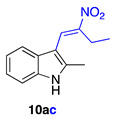



(*E*)-2-methyl-3-(2-nitrobut-1-en-1-yl)-1*H*-indole (**10ac**): This compound was prepared using Typical Procedure D employing 2-methyl-1*H*-indole-3- carbaldehyde (**29a**) (318 mg, 2.00 mmol) and nitropropane (1 mL). The titled compound was purified using column chromatography (EtOAc/hexane, 1:4, *v*/*v*). The titled compound was obtained as red solid, m.p. 134–138 °C, R*_f_* 0.4 (EtOAc/hexane, 1:3, *v*/*v*). Yield of 345 mg (1.5 mmol, 75%). ^1^H NMR (400 MHz, CDCl_3_) δ 8.44 (s, 1H), 8.20 (s, 1H), 7.51 (d, *J* = 7.3 Hz, 1H), 7.36 (dd, *J* = 6.9, 1.5 Hz, 1H), 7.25–7.18 (m, 2H), 2.87 (q, *J* = 7.3 Hz, 2H), 2.51 (s, 3H), 1.23 (t, *J* = 7.3 Hz, 3H); ^13^C{^1^H} NMR (101 MHz, CDCl_3_) δ 150.2, 138.6, 135.8, 128.1, 126.3, 122.7, 121.2, 119.7, 111.2, 106.9, 22.2, 13.2, 12.5; FTIR, *v_max_*: 3261, 2990, 1772, 1618, 1582, 1459, 1436, 1316, 1270, 1230 cm^−1^; HRMS (ESI TOF) *m*/*z* calc’d. for C_13_H_14_N_2_NaO_2_ [M+Na]^+^: 253.0947, found: 253.0950 (−0.8 ppm).

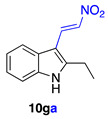



(*E*)-3-(2-nitrovinyl)-2-ethyl-1*H*-indole (**10ga**): This compound was prepared using Typical Procedure D employing 2-ethyl-1*H*-indole-3-carbaldehyde (**29g**) [40] (290 mg, 2.00 mmol) and nitromethane (1 mL). The titled compound was purified using column chromatography (EtOAc/hexane, 1:4, *v*/*v*). The titled compound was obtained as yellow solid, m.p. 188–189 °C, R*_f_* 0.35 (EtOAc/hexane, 1:3, *v*/*v*). Yield of 367 mg (1.7 mmol, 85%). ^1^H NMR (400 MHz, DMSO-*d_6_*) δ 12.21 (s, 1H), 8.32 (d, *J* = 13.2 Hz, 1H), 7.92 (d, *J* = 13.2 Hz, 1H), 7.85 (dd, *J* = 6.5, 1.9 Hz, 1H), 7.44 (dd, *J* = 6.7, 1.8 Hz, 1H), 7.28–7.14 (m, 2H), 2.98 (q, *J* = 7.6 Hz, 2H), 1.30 (t, *J* = 7.6 Hz, 3H); ^13^C{^1^H} NMR (101 MHz, DMSO-*d_6_*) δ 153.1, 136.7, 133.1, 129.9, 125.2, 123.1, 122.1, 120.5, 112.2, 104.3, 19.2, 14.3; FTIR, *v_max_*: 3224, 2911, 2903, 1624, 1588, 1556, 1434, 1266, 1159 cm^−1^; HRMS (ESI TOF) *m*/*z* calc’d. for C_12_H_12_N_2_NaO_2_ [M+Na]^+^: 239.0791, found: 239.0788 (1.3 ppm).

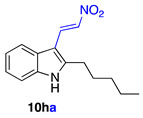



(*E*)-3-(2-nitrovinyl)-2-pentyl-1*H*-indole (**10ha**): This compound was prepared using Typical Procedure D employing 2-pentyl-1*H*-indole- 3-carbaldehyde (**29h**) (430 mg, 2.00 mmol) and nitromethane (1 mL). The titled compound was purified using column chromatography (EtOAc/hexane, 1:4, *v*/*v*). The titled compound was obtained as colorless solid, m.p. 113–115 °C, R*_f_* 0.4 (EtOAc/hexane, 1:3, *v*/*v*). Yield of 413 mg (1.60 mmol, 80%). ^1^H NMR (400 MHz, CDCl_3_) δ 9.05 (s, 1H), 8.37 (d, *J* = 13.2 Hz, 1H), 7.83 (d, *J* = 13.2 Hz, 1H), 7.74–7.64 (m, 1H), 7.48–7.38 (m, 1H), 7.32–7.26 (m, 2H), 2.96 (t, *J* = 7.7 Hz, 2H), 1.80–1.72 (m, 2H), 1.43–1.35 (m, 4H), 0.92 (t, 3H); ^13^C{^1^H} NMR (101 MHz, CDCl_3_) δ 150.0, 136.3, 133.3, 131.2, 125.6, 123.7, 122.7, 120.4, 111.9, 106.2, 31.5, 29.7, 26.6, 22.5, 14.0; FTIR, *v_max_*: 3265, 2960, 2933, 1603, 1560, 1510, 1454, 1238, 1159 cm^−1^; HRMS (ESI TOF) *m*/*z* calc’d. for C_15_H_18_N_2_NaO_2_ [M+Na]^+^: 281.1260, found: 281.1252 (2.9 ppm).

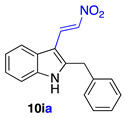



(*E*)-2-Benzyl-3-(2-nitrovinyl)-1*H*-indole (**10ia**): This compound was prepared using Typical Procedure D employing 2-benzyl-1*H*-indole-3-carbaldehyde (**29i**) [40] (470 mg, 2.00 mmol) and nitromethane (1 mL). The titled compound was purified using column chromatography (EtOAc/hexane, 1:4, *v*/*v*). The titled compound was obtained as red solid, m.p. 153.2–155.6 °C (EtOH), R*_f_* 0.56 (EtOAc/hexane, 1:2). Yield of 470 mg (1.86 mmol, 93%) ^1^H NMR (400 MHz, DMSO-*d_6_*) δ 12.34 (s, 1H), 8.43 (dd, *J* = 13.2, 2.3 Hz, 1H), 7.95 (dd, *J* = 13.2, 2.4 Hz, 1H), 7.90 (d, *J* = 7.3 Hz, 1H), 7.48–7.41 (m, 1H), 7.36–7.20 (m, 7H), 4.39 (s, 2H); ^13^C{^1^H} NMR (101 MHz, DMSO-*d_6_*) δ 149.2, 138.2, 136.7, 133.2, 130.6, 128.8 (2C), 128.4 (2C), 126.7, 125.1, 123.3, 122.1, 120.7, 112.3, 105.2, 31.6; FTIR, *v_max_*: 3237, 1614, 1576, 1478, 1461, 1298, 1274, 1244, 1226, 1171, 1097 cm^−1^; HRMS (ESI TOF) *m*/*z*: calc’d for C_17_H_14_N_2_NaO_2_ [M+Na]^+^: 301.0947, found 301.0941 (2.2 ppm). 

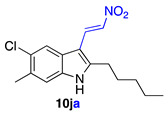



(*E*)-5-chloro-6-methyl-3-(2-nitrovinyl)-2-pentyl-1*H*-indole (**10ja**): This compound was prepared using Typical Procedure D employing 5-chloro-6-methyl-2-pentyl-1*H*-indole-3-carbaldehyde (**29j**) (526 mg, 2.00 mmol) and nitromethane (1 mL). The titled compound was purified using column chromatography (EtOAc/hexane, 1:4, *v*/*v*). The titled compound was obtained as yellow solid, m.p. 169–171 °C, R*_f_* 0.49 (EtOAc/hexane, 1:3, *v*/*v*). Yield of 490 mg (1.60 mmol, 80%). ^1^H NMR (400 MHz, CDCl_3_) δ 8.66 (s, 1H), 8.29 (d, *J* = 13.3 Hz, 1H), 7.77 (d, *J* = 13.3 Hz, 1H), 7.52 (s, 1H), 7.40 (s, 1H), 2.94 (t, *J* = 7.7 Hz, 2H), 2.50 (s, 3H), 1.79–1.69 (m, 2H), 1.43–1.34 (m, 4H), 0.92 (t, 3H); ^13^C{^1^H} NMR (101 MHz, CDCl_3_) δ 150.0, 135.1, 132.6, 131.7, 130.4, 130.2, 124.6, 121.8, 112.0, 105.7, 31.5, 29.6, 26.7, 22.5, 20.7, 14.1; FTIR, *v_max_*: 3265, 2960, 2933, 1603, 1560, 1510, 1454, 1238, 1159 cm^−1^; HRMS (ESI TOF) *m*/*z* calc’d. for C_16_H_19_ClN_2_NaO_2_ [M+Na]^+^: 329.1027, found: 329.1032 (−1.5 ppm).

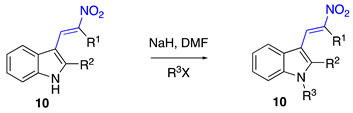



#### 3.2.5. Preparation of N-Substituted 3-(2-nitrovinyl)-1*H*-indoles

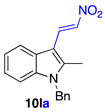



(*E*)-1-Benzyl-2-methyl-3-(2-nitrovinyl)-1*H*-indole (**10la**), Typical Procedure E for preparation of *N*-substituted 3-(2-nitrovinyl)-1*H*-indoles (**10**): This method was developed in analogy to that described in the literature [41]. To the solution of (*E*)-2-methyl-3-(2-nitrovinyl)-1*H*-indole (**10aa**) (202 mg, 2.00 mmol) in DMF (2 mL), NaH (120 mg, 3.00 mmol, 60% in mineral oil) was added portionwise at 0 °C; then, the mixture was stirred for 30 min. The alkyl halide benzyl bromide (513 mg, 356 µL, 3.00 mmol) was added dropwise; then, the reaction mixture was allowed to warm to room temperature under constant stirring. The reaction progress was monitored using TLC. After completion of the reaction, water was added and extracted with EtOAc. The combined organic layer was washed with water and brine and then dried over anhydrous Na_2_SO_4_. The solvents were removed under reduced pressure to obtain the crude product, which was then purified using column chromatography (EtOAc/hexane, 1:6, *v*/*v*). The titled compound was obtained as yellow solid, m.p. 45–47 °C, lit [42] m.p. 114–116 °C, R*_f_* 0.6 (EtOAc/hexane, 1:3, *v*/*v*). Yield of 237 mg (0.81.00 mmol, 81%). ^1^H NMR (400 MHz, CDCl_3_) δ 8.39 (d, *J* = 13.3 Hz, 1H), 7.84 (d, *J* = 13.2 Hz, 1H), 7.79–7.74 (m, 1H), 7.33–7.27 (m, 6H), 7.00 (dd, *J* = 7.6, 1.9 Hz, 2H), 5.39 (s, 2H), 2.55 (s, 3H); ^13^C{^1^H} NMR (101 MHz, CDCl_3_) δ 146.1, 138.0, 136.4, 133.0, 131.6, 129.3 (2C), 128.1, 126.0 (2C), 125.4, 124.4, 122.9, 120.5, 110.6, 106.7, 47.4, 11.1; FTIR, *v_max_*: 3030, 2920, 1893, 1869, 1650, 1623, 1557, 1505, 1495, 1474, 1446, 1328 cm^−1^; HRMS (ESI TOF) *m*/*z* calc’d. for C_18_H_16_N_2_NaO_2_ [M+Na]^+^: 315.1104, found: 315.1098 (2.0 ppm).

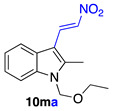



(*E*)-1-(Ethoxymethyl)-2-methyl-3-(2-nitrovinyl)-1*H*-indole (**10ma**): This compound was prepared using Typical Procedure E employing (*E*)-2-methyl-3-(2-nitrovinyl)-1*H*-indole (**10aa**) (404 mg, 2.00 mmol), (chloromethoxy)ethane (282 mg, 277 µL, 3.00 mmol). The titled compound was purified using column chromatography (EtOAc/hexane, 1:6, *v*/*v*). The titled compound was obtained as yellow solid, m.p. 79–81 °C, R*_f_* 0.37 (EtOAc/hexane, 1:3, *v*/*v*). Yield of 380 mg (1.46 mmol, 73%). ^1^H NMR (400 MHz, CDCl_3_) δ 8.33 (d, *J* = 13.3 Hz, 1H), 7.77 (d, *J* = 13.3 Hz, 1H), 7.70–7.65 (m, 1H), 7.52–7.47 (m, 1H), 7.35–7.28 (m, 2H), 5.52 (s, 2H), 3.49 (q, *J* = 7.0 Hz, 2H), 2.63 (s, 3H), 1.18 (t, *J* = 7.0 Hz, 3H); ^13^C{^1^H} NMR (101 MHz, CDCl_3_) δ 145.9, 138.0, 132.6, 132.1, 125.2, 123.7, 122.9, 120.2, 110.3, 107.1, 73.0, 64.5, 15.0, 10.8; FTIR, *v_max_*: 2963, 2930, 2861, 1734, 1716, 1701, 1686, 1653, 1558, 1505, 1457, 1373, 1340, 1240 cm^−1^; HRMS (ESI TOF) *m*/*z* calc’d. for C_14_H_16_N_2_NaO_3_ [M+Na]^+^: 283.1053, found: 283.1059 (−1.9 ppm).

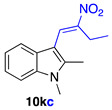



(*E*)-1,2-Dimethyl-3-(2-nitrobut-1-en-1-yl)-1*H*-indole (**10kc**): This compound was prepared using Typical Procedure E employing (*E*)-2-methyl-3-(2-nitrobut-1-en-1-yl)-1*H*-indole (**10ac**) (460 mg, 2.00 mmol), methyl iodide (426 mg, 187 µL, 3.00 mmol). The titled compound was purified using column chromatography (EtOAc/hexane, 1:6, *v*/*v*). The titled compound was obtained as yellow solid, m.p. 149–151 °C, R*_f_* 0.43 (EtOAc/hexane, 1:3, *v*/*v*). Yield of 414 mg (1.7 mmol, 85%). ^1^H NMR (400 MHz, CDCl_3_) δ 8.23 (s, 1H), 7.52 (d, *J* = 7.8 Hz, 1H), 7.34 (d, *J* = 8.1 Hz, 1H), 7.27 (t, *J* = 7.4 Hz, 1H), 7.21 (t, *J* = 7.5 Hz, 1H), 3.74 (s, 3H), 2.86 (q, *J* = 7.2 Hz, 2H), 2.47 (s, 3H), 1.22 (t, *J* = 7.3 Hz, 3H); ^13^C{^1^H} NMR (101 MHz, CDCl_3_) δ 149.7, 140.4, 137.5, 128.5, 125.5, 122.3, 121.1, 119.7, 109.6, 106.2, 30.2, 22.2, 12.5, 11.7; FTIR, *v_max_*: 3298, 3202, 2821, 1869, 1736, 1721, 1694, 1625, 1558, 1509, 1472, 1376, 1242 cm^−1^; HRMS (ESI TOF) *m*/*z* calc’d. for C_14_H_16_N_2_NaO_2_ [M+Na]^+^: 267.1104, found: 267.1111 (−2.7 ppm).

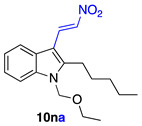



(*E*)-1-(Ethoxymethyl)-3-(2-nitrovinyl)-2-pentyl-1*H*-indole (**10na**): This compound was prepared using Typical Procedure E employing (*E*)-3-(2- nitrovinyl)-2-pentyl-1*H*-indole (**10ha**) (516 mg, 2.00 mmol), (chloromethoxy)ethane (282 mg, 277 µL, 3.00 mmol). The titled compound was purified using column chromatography (EtOAc/hexane, 1:6, *v*/*v*). The titled compound was obtained as yellow solid, m.p. 75–76 °C, R*_f_* 0.29 (EtOAc/hexane, 1:3, *v*/*v*). Yield of 334 mg (1.06 mmol, 53%). ^1^H NMR (400 MHz, CDCl_3_) δ 8.33 (d, *J* = 13.3 Hz, 1H), 7.79 (d, *J* = 13.3 Hz, 1H), 7.73–7.66 (m, 1H), 7.55–7.49 (m, 1H), 7.37–7.27 (m, 2H), 5.53 (s, 2H), 3.52 (q, *J* = 7.0 Hz, 2H), 3.06–2.94 (m, 2H), 1.72–1.63 (m, 2H), 1.47–1.36 (m, 4H), 1.19 (t, *J* = 7.0 Hz, 3H), 0.93 (t, *J* = 7.0 Hz, 3H); ^13^C{^1^H} NMR (101 MHz, CDCl_3_) δ 150.5, 138.0, 132.5, 132.0, 125.2, 123.7, 122.9, 120.3, 110.6, 106.7, 72.9, 64.5, 31.6, 30.5, 24.7, 22.4, 15.0, 14.0; FTIR, *v_max_*: 3324, 3248, 2983, 1769, 1684, 1633, 1557, 1504, 1461, 1386, 1374, 1245 cm^−1^; HRMS (ESI TOF) *m*/*z* calc’d. for C_18_H_24_N_2_NaO_3_ [M+Na]^+^: 339.1679, found: 339.1672 (2.2 ppm).

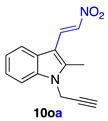



(*E*)-2-Methyl-3-(2-nitrovinyl)-1-(prop-2-yn-1-yl)-1*H*-indole (**10oa**): This compound was prepared using Typical Procedure E employing (*E*)-2-methyl-3-(2′-nitrovinyl)indole (**10aa**) (1.01 g, 5 mmol), propargyl bromide (1.11 g, 0.8 mL 7.5 mmol, 80 wt. % in toluene) with the yield of 1.14 g (4.75 mmol, 95%). Purification was performed via recrystallization from ethanol. The titled compound was obtained as an orange solid, m.p. 105–106 °C (EtOH), R*_f_* 0.64 (EtOAc/PE, 1:2, *v*/*v*). ^1^H NMR (400 MHz, DMSO-*d*_6_) δ 8.34 (d, *J* = 13.3 Hz, 1H), 7.97 (d, *J* = 13.2 Hz, 1H), 7.91 (d, *J* = 7.7 Hz, 1H), 7.67 (d, *J* = 8.0 Hz, 1H), 7.32 (t, *J* = 7.5 Hz, 1H), 7.27 (t, *J* = 7.4 Hz, 1H), 5.19 (s, 2H), 3.43 (s, 1H), 2.66 (s, 3H). ^13^C NMR (101 MHz, DMSO) δ 147.3, 136.8, 132.9, 131.0, 124.6, 123.2, 122.6, 120.5, 110.9, 105.7, 78.2, 75.7, 32.9, 10.5. IR, *v_max_*: 3273, 2918, 1743, 1572, 1476, 1298, 1226, 1171, 1077 cm^−1^; HRMS (ESI TOF) *m*/*z* calcd. for C_14_H_12_N_2_NaO_2_ [M+Na]^+^: 263.0791, found: 263.0798 (−2.7 ppm).

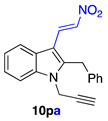



(*E*)-2-benzyl-3-(2-nitrovinyl)-1-(prop-2-yn-1-yl)-1*H*-indole (**10pa**): This compound was prepared using Typical Procedure E employing (*E*)-2-benzyl-3-(2′-nitrovinyl)indole (**10ai**) (1.39 g, 5 mmol), propargyl bromide (1.11 g, 0.8 mL 7.5 mmol, 80 wt. % in toluene) with the yield of 1.26 g (4 mmol, 80%). Purification was performed via recrystallization from ethanol. The titled compound was obtained as an orange solid, m.p. 105–106 °C (EtOH), R*_f_* 0.72 (EtOAc/PE, 1:2, *v*/*v*). ^1^H NMR (400 MHz, Chloroform-*d*) δ 8.41 (d, *J* = 13.3 Hz, 1H), 7.88 (d, *J* = 13.3 Hz, 1H), 7.84–7.80 (m, 1H), 7.48–7.44 (m, 1H), 7.40–7.36 (m, 2H), 7.34–7.27 (m, 3H), 7.12 (d, *J* = 7.0 Hz, 2H), 4.71 (d, *J* = 2.5 Hz, 2H), 4.45 (s, 2H), 2.29 (t, *J* = 2.5 Hz, 1H).^13^C NMR (101 MHz, CDCl_3_) δ 145.9, 137.3, 136.0, 132.9, 132.5, 129.3 (2C), 128.2 (2C), 127.5, 125.3, 124.1, 123.1, 120.8, 110.5, 107.9, 76.5, 73.9, 33.6, 30.5. IR, *v_max_*: 3233, 2962, 1523, 1442, 1297, 1243, 1183, 1096 cm^−1^; HRMS (ESI TOF) *m*/*z* calcd. for C_20_H_16_N_2_NaO_2_ [M+Na]^+^: 339.1104, found: 339.1097 (2.1 ppm).

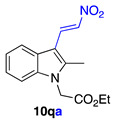



(*E*)-Ethyl 2-(2-methyl-3-(2-nitrovinyl)-1*H*-indol-1-yl)acetate (**10qa**): This compound was prepared using Typical Procedure E employing E-2-methyl-3-(2′-nitrovinyl)indole (**10aa**) (1.01 g, 5 mmol), ethyl 2-chloroacetate (915 mg, 7.5 mmol) with the yield of 1.037 g (3.6 mmol, 72%). The titled compound was purified using column chromatography (EtOAc/hexane, 1:1, *v*/*v*). The titled compound was obtained as yellow solid, m.p. 110–112 °C, R_f_ 0.25 (EtOAc/hexane, 1:1, *v*/*v*). ^1^H NMR (400 MHz, DMSO-*d*_6_) δ 8.36 (d, *J* = 13.2 Hz, 1H), 7.99 (d, *J* = 13.2 Hz, 1H), 7.95–7.87 (m, 1H), 7.60–7.53 (m, 1H), 7.31–7.23 (m, 2H), 5.27 (s, 2H), 4.17 (q, *J* = 7.1 Hz, 2H), 2.55 (s, 3H), 1.22 (t, *J* = 7.1 Hz, 3H).^13^C NMR (101 MHz, DMSO) δ 168.2, 148.4, 137.7, 133.1, 130.9, 124.5, 123.2, 122.6, 120.4, 110.7, 105.6, 61.5, 45.0, 14.1, 10.5. IR, *v_max_*: 3098, 2981, 2925, 2853, 1744, 1671, 1629, 1136, 1098, 1010, 910, 862 cm^−1^; HRMS (ESI TOF) *m*/*z* calcd. For C_15_H_16_N_2_NaO_4_ [M+Na]^+^: 311,1002, found: 311,1006 (−1.3 ppm).

#### 3.2.6. Synthesis of (3R,4′S)-2-methyl-4′-phenyl-4′H-spiro[indole-3,5′-isoxazole]

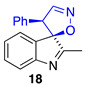



The reaction vessel was charged with 2-methyl-1*H*-indole (131 mg, 1.00 mmol), (2- nitrovinyl)benzene (164 mg, 1.10 mmol), phosphorous acid (1000 mg), and formic acid (1000 mg). The mixture was vigorously stirred for 2 h at 10 °C. The resulting dark-red homogeneous solution was poured into water (100 mL), and the formed precipitate was filtered and washed consecutively with water (four times). After drying, the resulting crystalline material was purified using flash column chromatography on silica gel, eluting with a mixture of hexane and ethyl acetate (4:1) [34].

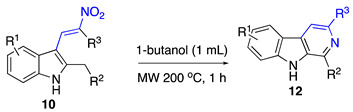



### 3.3. Synthesis of β-Carbolines Starting from 3-Nitrovinylindoles as Shown in Figure 7

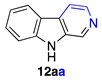



β-Carboline (norharmane) (**12aa**), Typical Procedure A (Method A) for preparation of β-carbolines (**12**): (*E*)-3-(2-nitrovinyl)-2-methyl-1*H*-indole (**10aa**) (202 mg, 1.00 mmol) and 2 mL of 1-butanol were charged in a G10 vial. The vial was sealed and heated in the microwave apparatus at 200 °C for 1 h. After completion of the reaction, the vial was opened, and the reaction mixture was concentrated in vacuo. Crude material was purified using column chromatography (benzene/ethanol, 9:1, *v*/*v*). The titled compound was obtained as colorless solid, m.p. 197–198 °C, lit [43] m.p. 196–197 °C, R*_f_* 0.43 (benzene/ethanol, 1:9, *v*/*v*). Yield of 82 mg (0.49 mmol, 49%). ^1^H NMR (400 MHz, CDCl_3_) δ 8.93 (s, 1H), 8.54 (s, 1H), 8.48 (d, *J* = 5.3 Hz, 1H), 8.15 (d, *J* = 7.8 Hz, 1H), 7.98 (d, *J* = 5.4 Hz, 1H), 7.60–7.51 (m, 2H), 7.31 (t, *J* = 6.5 Hz, 1H); ^13^C{^1^H} NMR (101 MHz, CDCl_3_) δ 140.4, 139.4, 135.9, 133.8, 129.1, 128.7, 122.0, 121.6, 120.3, 114.9, 111.7; FTIR, *v_max_*: 3384, 2937, 2364, 2321, 1653, 1621, 1557, 1529, 1472, 1444, 1417 cm^−1^; HRMS (ESI TOF) *m*/*z* calc’d. for C_11_H_9_N_2_ [M+H]^+^: 169.0760, found: 169.0758 (1.4 ppm).

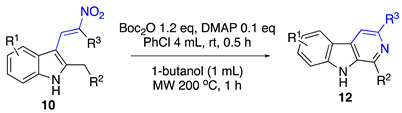



Typical Procedure B (Method B) for preparation of β-carbolines (**12**): (*E*)-3-(2-nitrovinyl)-2-methyl-1*H*-indole (**10aa**) (202 mg, 1.00 mmol) and 4 mL of chlorobenzene were charged in a G10 vial. Then, Boc_2_O (262 mg, 1.20 mmol) and dimethylaminopyridine (DMAP) (12 mg, 0.100 mmol) were added, and the resulting mixture was stirred at room temperature for 30 min. After this period of time, the resulting mixture was diluted with 1 mL of BuOH, and the vial was sealed and heated in the microwave oven at 200 °C for 1 h. After completion of the reaction, water (20 mL) was added, followed by extraction with EtOAc (4 × 20 mL). Combined organic phases were concentrated in vacuo. Crude material was purified using column chromatography (benzene/ethanol, 9:1, *v*/*v*). Yield of 116 mg (0.69 mmol, 69%). The obtained sample of **12aa** was identical to that obtained using Typical Procedure A.

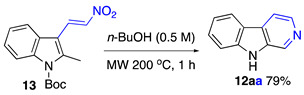



Preparation (Procedure F) of β-carboline 12aa from *tert*-butyl (*E*)-2-methyl-3-(2-nitrovinyl)-1H-indole-1-carboxylate (**13**): *tert*-butyl (*E*)-2-methyl-3-(2-nitrovinyl)-1*H*-indole-1-carboxylate (**15**) [33] (302 mg, 1.00 mmol) and 2 mL of 1-butanol were charged in a G10 vial. The vial was sealed and heated in the microwave apparatus at 200 °C for 1 h. After completion of the reaction, the vial was opened, and the reaction mixture was concentrated in vacuo. Crude material was purified using column chromatography (benzene/ethanol, 9:1, *v*/*v*). Yield of 133 mg (0.79 mmol, 79%). The obtained sample of norharmane (**12aa**) was identical to that obtained using Typical Procedure A.

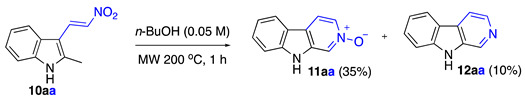

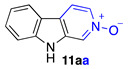





β-Carboline-2-oxide (**11aa**): 3-(2-Nitrovinyl)-2-methylindole (**10aa**) (1.00 mmol) and 20 mL of 1-butanol were charged in a G30 vial. The vial was sealed and heated in the microwave apparatus at 200 °C for 60 min. After completion of the reaction, the vial was opened, and the reaction mixture was evaporated to dryness. Crude material was purified using column chromatography (benzene/ethanol, 1:1, *v*/*v*), giving β-carboline-2-oxide (**11aa**) in the yield of 64 mg (0.35 mmol, 35%), along with β-carboline (**12aa**) in the yield of 17 mg (0.100 mmol, 10 mol%). The titled compound was obtained as colorless solid, m.p. 269–270 °C, lit [44]. m.p. 238–240 °C, R*_f_* 0.4 (benzene/ethanol, 1:1, *v*/*v*). ^1^H NMR (400 MHz, DMSO-*d_6_*) 11.64 (s, 1H), 8.56 (s, 1H), 8.13 (dd, *J* = 10.3, 7.3 Hz, 2H), 8.03 (dd, *J* = 6.7, 1.4 Hz, 1H), 7.55 (d, *J* = 8.2 Hz, 1H), 7.50–7.44 (m, 1H), 7.26–7.21 (m, 1H); ^13^C{^1^H} NMR (101 MHz, DMSO-*d_6_*) δ 141.5, 137.1, 130.9, 127.4, 123.3, 121.1, 120.8, 120.2, 119.7, 116.9, 111.9; FTIR, *v_max_*: 3443, 3221, 1658, 1526, 1476, 1382, 1293, 1148 cm^−1^; HRMS (ESI TOF) *m*/*z* calc’d. for C_11_H_8_N_2_NaO [M+Na]^+^: 207.0529, found: 207.0527 (1.1 ppm).

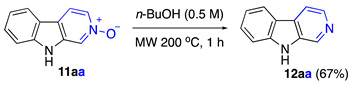



Preparation (Procedure G) of β-carboline (**12aa**) from β-carboline-2-oxide (**11aa**): β-Carboline-2-oxide (**11aa**) (92 mg, 0.5 mmol) and 1 mL of 1-butanol were charged in a G10 vial. The vial was sealed and heated in the microwave apparatus at 200 °C for 60 min. After completion of the reaction, the vial was opened, and the reaction mixture was concentrated in vacuo. Crude material was purified using column chromatography (benzene/ethanol, 9:1, *v*/*v*). Yield of 56 mg (0.34 mmol, 67%). The obtained sample was identical to that obtained by Typical Procedure A.

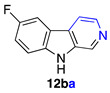



6-Fluoro-β-carboline (**12ba**): This compound was prepared using Method A employing (*E*)-5-fluoro-2-methyl-3-(2-nitrovinyl)-1*H*-indole (**10ba**) (220 mg, 1.00 mmol) in the yield of 73 mg (0.39 mmol, 39%). Alternatively, this compound was prepared using Method B employing (*E*)-5-fluoro-2-methyl-3-(2-nitrovinyl)-1*H*-indole (**10ba**) (220 mg, 1.00 mmol) in the yield of 99 mg (0.53 mmol, 53%). Purification was performed using column chromatography (EtOAc). The titled compound was obtained as white solid, m.p. 248.8–250.6 °C, lit [45]. m.p. 254 °C, R*_f_* 0.16 (EtOAc). ^1^H NMR (400 MHz, DMSO-*d_6_*) δ 11.68 (s, 1H), 8.92 (s, 1H), 8.33 (d, *J* = 5.3 Hz, 1H), 8.20–7.96 (m, 2H), 7.61 (dd, *J* = 8.9, 4.4 Hz, 1H), 7.41 (td, *J* = 9.2, 2.7 Hz, 1H); ^13^C{^1^H} NMR (101 MHz, DMSO-*d_6_*) δ 156.6 (d, *J* = 233.9 Hz), 138.0, 137.1, 137.0, 134.6, 127.2 (d, *J* = 4.5 Hz), 121.0 (d, *J* = 10.0 Hz), 116.4 (d, *J* = 25.8 Hz), 115.1, 113.2 (d, *J* = 9.2 Hz), 107.2 (d, *J* = 23.6 Hz); ^19^F NMR (376 MHz, DMSO-*d*_6_) δ -121.59; FTIR, *v_max_*: 3053, 2924, 2854, 1587, 1507, 1464, 1441, 1279, 1179, 1153 cm^−1^; HRMS (ESI TOF) *m*/*z* calc’d. for C_11_H_8_FN_2_ [M+H]^+^: 187.0666, found: 187.0666 (−0.1 ppm).

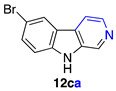



6-Bromo-β-carboline (eudistomin N) (**12ca**): This compound was prepared using Method A employing (*E*)-5-bromo-2-methyl-3-(2-nitrovinyl)-1*H*-indole (**10ca**) [32] (280 mg, 1.00 mmol) in the yield of 86 mg (0.35 mmol, 35%). Alternatively, this compound was prepared using Method B employing (*E*)-5-bromo-2- methyl-3-(2-nitrovinyl)- 1*H*-indole (**10ca**) [35] (280 mg, 1.00 mmol) in the yield of 152 mg (0.62 mmol, 62%). Purification was performed using column chromatography (benzene/ethanol, 9:1, *v*/*v*). The titled compound was obtained as yellowish solid, m.p. 270–271 °C, lit [43]. m.p. 266–268 °C, R*_f_* 0.47 (benzene/ethanol, 1:9, *v*/*v*). ^1^H NMR (400 MHz, DMSO-*d_6_*) δ 11.80 (s, 1H), 8.93 (d, *J* = 1.0 Hz, 1H), 8.51 (d, *J* = 2.1 Hz, 1H), 8.36 (d, *J* = 5.3 Hz, 1H), 8.16 (d, *J* = 5.3 Hz, 1H), 7.66 (dd, *J* = 8.7, 2.1 Hz, 1H), 7.57 (d, *J* = 8.7 Hz, 1H).; ^13^C{^1^H} NMR (101 MHz, DMSO-*d_6_*) δ 139.2, 138.5, 136.3, 134.5, 130.6, 126.6, 124.5, 122.6, 115.1, 114.1, 111.3 FTIR, *v_max_*: 3122, 3063, 2738, 1867, 1721, 1684, 1656, 1630, 1558, 1540, 1487, 1434, 1273, 1243 cm^−1^; HRMS (ESI TOF) *m*/*z* calc’d. for C_11_H_8_BrN_2_ [M+H]^+^: 246.9865, found: 246.9862 (1.5 ppm).

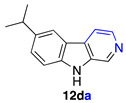



6-Isopropyl-β-carboline (**12da**): This compound was prepared using Method A employing (*E*)-5-isopropyl-2-methyl-3-(2-nitrovinyl)-1*H*-indole (**10da**) (244 mg, 1.00 mmol) in the yield of 101 mg (0.48 mmol, 48%). Alternatively, this compound was prepared using Method B employing (*E*)-5-isopropyl-2-methyl- 3-(2-nitrovinyl)-1*H*-indole (**10da**) (244 mg, 1.00 mmol) in the yield of 139 mg (0.66 mmol, 66%). Purification was performed using column chromatography (EtOAc). The titled compound was obtained as colorless solid, m.p. 173.9–176.2 °C, R*_f_* 0.18 (EtOAc). ^1^H NMR (400 MHz, DMSO-*d_6_*) δ 11.49 (s, 1H), 8.87 (s, 1H), 8.30 (d, *J* = 5.2 Hz, 1H), 8.11–8.05 (m, 2H), 7.51 (d, *J* = 8.4 Hz, 1H), 7.44 (dd, *J* = 8.5, 1.6 Hz, 1H), 3.14–2.98 (m, 1H), 1.31 (s, 3H), 1.29 (s, 3H); ^13^C{^1^H} NMR (101 MHz, DMSO) δ 139.6, 139.2, 137.9, 136.4, 134.0, 127.5, 127.3, 120.7, 118.6, 114.7, 111.8, 33.6, 24.6 (2C); FTIR, *v_max_*: 3133, 3036, 2960, 1635, 1554, 1498, 1461, 1269, 1250 cm^−1^; HRMS (ESI TOF) *m*/*z*: calc’d for C_14_H_15_N_2_ [M+H]^+^: 211.1230, found 211.1228 (0.8 ppm).

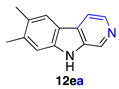



6,7-Dimethyl-β-carboline (**12ea**): This compound was prepared using Method A employing (*E*)-2,5,6-trimethyl-3-(2-nitrovinyl)-1*H*-indole (**10ea**) (230 mg, 1.00 mmol) in the yield of 90 mg (0.46 mmol, 46%). Alternatively, this compound was prepared using Method B employing (*E*)-2,5,6-trimethyl-3-(2-nitrovinyl)- 1*H*-indole (**10ea**) (230 mg, 1.00 mmol) in the yield of 125 mg (0.64 mmol, 64%). Purification was performed using column chromatography (EtOAc). The titled compound was obtained as colorless solid, m.p. 263.2–264.8 °C, R*_f_* 0.12 (EtOAc). ^1^H NMR (400 MHz, DMSO-*d_6_*) δ 11.39 (s, 1H), 8.82 (d, *J* = 1.0 Hz, 1H), 8.28 (d, *J* = 5.2 Hz, 1H), 7.99 (d, *J* = 5.2 Hz, 1H), 7.97 (s, 1H), 7.38 (s, 1H), 2.40 (s, 3H), 2.37 (s, 3H); ^13^C{^1^H} NMR (101 MHz, DMSO) δ 139.6, 137.9, 137.4, 136.1, 133.7, 127.7, 127.4, 121.7, 118.8, 114.3, 112.2, 20.62, 19.7; FTIR, *v_max_*: 3053, 2924, 1638, 1553, 1452, 1317, 1262, 1246 cm^−1^; HRMS (ESI TOF) *m*/*z*: calc’d for C_13_H_13_N_2_ [M+H]^+^: 197.1073, found 197.1070 (1.9 ppm). 

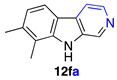



7,8-Dimethyl-β-carboline (**12fa**): This compound was prepared using Method A employing (*E*)-2,6,7-trimethyl-3-(2-nitrovinyl)-1*H*-indole (**10fa**) (230 mg, 1.00 mmol) in the yield of 92 mg (0.47 mmol, 47%). Alternatively, this compound was prepared using Method B employing (*E*)-2,6,7-trimethyl-3-(2-nitrovinyl)- 1*H*-indole (230 mg, 1.00 mmol) (**10fa**) in the yield of 125 mg (0.64 mmol, 64%). Purification was performed using column chromatography (EtOAc). The titled compound was obtained as colorless solid, m.p. 178.9–181.1 °C, R*_f_* 0.14 (EtOAc). ^1^H NMR (400 MHz, Chloroform-d) δ 9.18 (s, 1H), 8.91 (s, 1H), 8.45 (d, *J* = 5.4 Hz, 1H), 8.08 (d, *J* = 5.4 Hz, 1H), 7.35 (d, *J* = 8.3 Hz, 1H), 7.26 (d, *J* = 8.2 Hz, 1H), 2.79 (s, 3H), 2.47 (s, 3H); ^13^C{^1^H} NMR (101 MHz, CDCl_3_) δ 139.5, 138.7, 136.4, 133.5, 132.9, 130.9, 129.4, 127.7, 120.8, 117.0, 108.7, 19.5, 16.6; FTIR, *v_max_*: 3123, 2924, 2854, 1648, 1623, 1504, 1457, 1327, 1300, 1277, 1149 cm^−1^; HRMS (ESI TOF) *m*/*z*: calc’d for C_13_H_13_N_2_ [M+H]^+^: 197.1073, found 197.1070 (1.5 ppm). 

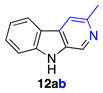



3-Methyl-β-carboline (**12ab**): This compound was prepared using Method A employing (*E*)-2-methyl-3-(2-nitroprop-1-en-1-yl)-1*H*-indole (**10ab**) (216 mg, 1.00 mmol) in the yield of 84 mg (0.46 mmol, 46%). Alternatively, this compound was prepared using Method B employing (*E*)-2-methyl-3-(2-nitroprop-1-en-1-yl)-1*H*- indole (**10ab**) (216 mg, 1.00 mmol) in the yield of 116 mg (0.64 mmol, 64%). Purification was performed using column chromatography (benzene/ethanol, 9:1, *v*/*v*). The titled compound was obtained as colorless solid, m.p. 210–212 °C, lit [46]. m.p. 208 °C, R*_f_* 0.40 (benzene/ethanol, 1:9, *v*/*v*). ^1^H NMR (400 MHz, DMSO-*d_6_*) δ 11.45 (s, 1H), 8.76 (s, 1H), 8.18 (d, *J* = 7.8 Hz, 1H), 7.94 (s, 1H), 7.57–7.49 (m, 2H), 7.23–7.18 (m, 1H), 2.61 (s, 3H); ^13^C{^1^H} NMR (101 MHz, DMSO-*d_6_*) δ 146.2, 141.4, 134.8, 133.1, 129.1, 128.6, 122.2, 120.9, 119.5, 113.7, 112.3, 24.2; FTIR, *v_max_*: 3119, 2993, 1918, 1683, 1648, 1631, 1563, 1504, 1471, 1457, 1330, 1245 cm^−1^; HRMS (ESI TOF) *m*/*z* calc’d. for C_12_H_11_N_2_ [M+H]^+^: 183.0917, found: 183.0915 (0.8 ppm).

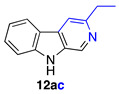



3-Ethyl-β-carboline (**12ac**): This compound was prepared using Method A employing (*E*)-2-methyl-3-(2-nitrobut-1-en-1-yl)-1*H*-indole (**10ac**) (230 mg, 1.00 mmol) in the yield of 92 mg (0.47 mmol, 47%). Alternatively, this compound was prepared using Method B employing (*E*)-2-methyl-3-(2-nitrobut-1-en-1-yl)- 1*H*-indole (**10ac**) (230 mg, 1.00 mmol) in the yield of 127 mg (0.65 mmol, 65%). Purification was performed using column chromatography (benzene/ethanol, 9:1, *v*/*v*). The titled compound was obtained as colorless solid, m.p. 120–121 °C, R*_f_* 0.27 (benzene/ethanol, 1:9, *v*/*v*). ^1^H NMR (400 MHz, CDCl_3_) δ 8.85 (s, 1H), 8.80 (s, 1H), 8.12 (d, *J* = 7.9 Hz, 1H), 7.83 (s, 1H), 7.65–7.38 (m, 2H), 7.36–7.12 (m, 1H), 3.03 (q, *J* = 7.6 Hz, 2H), 1.42 (t, *J* = 7.6 Hz, 3H); ^13^C{^1^H} NMR (101 MHz, CDCl_3_) δ 153.1, 141.1, 134.6, 132.7, 130.2, 128.5, 121.9, 121.6, 120.0, 112.5, 111.7, 31.3, 15.0; FTIR, *v_max_*: 2957, 2930, 1734, 1703, 1686, 1563, 1623, 1558, 1504, 1471, 1452, 1439, 1343, 1245 cm^−1^; HRMS (ESI TOF) *m*/*z* calc’d. for C_13_H_13_N_2_ [M+H]^+^: 197.1073, found: 197.1069 (2.3 ppm). 

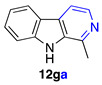



1-Methyl-β-carboline (harmane) (**12ga**): This compound was prepared using Method A employing (*E*)-2-ethyl-3-(2-nitrovinyl)-1*H*-indole (**10ga**) (216 mg, 1.00 mmol) in the yield of 87 mg (0.48 mmol, 48%). Alternatively, this compound was prepared using Method B employing (*E*)-2-ethyl-3-(2-nitrovinyl)-1*H*-indole (**10ga**) (216 mg, 1.00 mmol) in the yield of 120 mg (0.66 mmol, 66%). The titled compound was purified using column chromatography (benzene/ethanol, 9:1, *v*/*v*). The titled compound was obtained as colorless solid, m.p. 234–235 °C, lit [43]. m.p. 235–236 °C, R*_f_* 0.40 (benzene/ethanol, 1:9, *v*/*v*). ^1^H NMR (400 MHz, CDCl_3_) δ 8.57 (s, 1H), 8.37 (d, *J* = 5.4 Hz, 1H), 8.12 (d, *J* = 7.9 Hz, 1H), 7.83 (d, *J* = 5.4 Hz, 1H), 7.57–7.50 (m, 2H), 7.29 (ddd, *J* = 8.1, 5.8, 2.4 Hz, 1H), 2.83 (s, 3H); ^13^C{^1^H} NMR (101 MHz, CDCl_3_) δ 141.9, 140.2, 138.8, 134.7, 128.5, 128.4, 122.2, 122.0, 120.3, 113.1, 111.7, 20.4; FTIR (film, NaCl, cm^−1^): 3123, 2991, 1932, 1632, 1533, 1512, 1461, 1488, 1322, 1211, 1011 cm^−1^; HRMS (ESI TOF) *m*/*z* Calc’d. for C_12_H_11_N_2_ [M+H]^+^: 183.0917, found: 183.0914 (1.3 ppm).

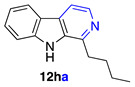



1-Butyl-β-carboline (**12ha**): This compound was prepared using Method A employing (*E*)-3-(2-nitrovinyl)-2-pentyl-1*H*-indole (**10ha**) (258 mg, 1.00 mmol) in the yield of 99 mg (0.44 mmol, 44%). Alternatively, this compound was prepared using Method B employing (*E*)-3-(2-nitrovinyl)-2-pentyl-1*H*-indole (**10ha**) (258 mg, 1.00 mmol) in the yield of 137 mg (0.61 mmol, 61%). Purification was performed using column chromatography (benzene/ethanol, 9:1, *v*/*v*). The titled compound was obtained as yellowish solid, m.p. 55–57 °C, R*_f_* 0.53 (benzene/ethanol, 1:9, *v*/*v*). ^1^H NMR (400 MHz, CDCl_3_) δ 8.67 (s, 1H), 8.40 (d, *J* = 5.4 Hz, 1H), 8.12 (d, *J* = 7.8 Hz, 1H), 7.82 (d, *J* = 5.3 Hz, 1H), 7.56–7.50 (m, 2H), 7.31–7.26 (m, 1H), 3.12 (t, 2H), 1.92–1.84 (m, 2H), 1.49–1.39 (m, 2H), 0.93 (t, *J* = 7.3 Hz, 3H); ^13^C{^1^H} NMR (101 MHz, CDCl_3_) δ 146.1, 140.2, 138.8, 134.3, 128.7, 128.3, 122.8, 121.9, 120.2, 113.0, 111.7, 34.3, 31.0, 23.0, 14.1 FTIR, *v_max_*: 3063, 2953, 2927, 2857, 1734, 1718, 1699, 1684, 1655, 1507, 1456, 1374, 1238 cm^−1^; HRMS (ESI TOF) *m*/*z* calc’d. for C_15_H_17_N_2_ [M+H]^+^: 225.1386, found: 225.1381 (2.3 ppm).

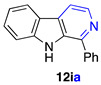



1-Phenyl-β-carboline (**12ia**): This compound was prepared using Method A employing (*E*)-2-benzyl-3-(2-nitrovinyl)-1*H*-indole (**10ia**) (278 mg, 1.00 mmol) in the yield of 120 mg (0.49 mmol, 49%). Alternatively, this compound was prepared using Method B employing (*E*)-2-benzyl-3-(2-nitrovinyl)-1*H*-indole (**10ia**) (278 mg, 1.00 mmol) in the yield of 166 mg (0.68 mmol, 68%). Purification was performed using column chromatography (EtOAc/hexane, 1:2, *v*/*v*). The titled compound was obtained as colorless solid, m.p. 239.6–241.0 °C, lit [43]. m.p. 242–244 °C, R*_f_* 0.51 (EtOAc/hexane, 1:1). ^1^H NMR (400 MHz, DMSO-*d_6_*) 11.53 (s, 1H), 8.46 (d, *J* = 5.2 Hz, 1H), 8.27 (d, *J* = 7.8 Hz, 1H), 8.12 (d, *J* = 5.2 Hz, 1H), 8.08–8.00 (m, 2H), 7.67–7.50 (m, 5H), 7.26 (ddd, *J* = 8.0, 7.0, 1.0 Hz, 1H); ^13^C{^1^H} NMR (101 MHz, DMSO) δ 142.2, 141.1, 138.4, 138.4, 133.0, 129.2, 128.8 (2C), 128.6, 128.4 (2C), 128.2, 121.7, 120.9, 119.6, 114.0, 112.5; FTIR, *v_max_*: 2924, 2857, 1621, 1558, 1499, 1468, 1456, 1445, 1415, 1325 cm^−1^; HRMS (ESI TOF) *m*/*z*: calc’d for C_17_H_13_N_2_ [M+H]^+^: 245.1073, found 245.1067 (2.4 ppm). 

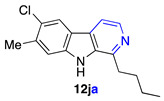



1-Butyl-6-chloro-7-methyl-β-carboline (**12ja**): This compound was prepared using Method A employing (*E*)-5-chloro-6-methyl-3-(2-nitrovinyl)-2-pentyl-1*H*-indole (**10ja**) (307 mg, 1.00 mmol) in the yield of 131 mg (0.48 mmol, 48%). Alternatively, this compound was prepared using Method B employing (*E*)-5-chloro-6-methyl-3-(2-nitrovinyl)-2-pentyl- 1*H*-indole (10ja) (307 mg, 1.00 mmol) in the yield of 185 mg (0.68 mmol, 68%). Purification was performed using column chromatography (benzene/ethanol, 9:1, *v*/*v*). The titled compound was obtained as yellowish solid, m.p. 181–182 °C, R*_f_* 0.5 (benzene/ethanol, 1:9, *v*/*v*). ^1^H NMR (400 MHz, CDCl_3_) δ 8.48 (s, 1H), 8.38 (d, *J* = 5.4 Hz, 1H), 7.93 (s, 1H), 7.74 (d, *J* = 5.4 Hz, 1H), 7.52 (s, 1H), 3.08 (t, *J* = 7.9 Hz, 2H), 2.53 (s, 3H), 1.91–1.81 (m, 2H), 1.48–1.40 (m, 2H), 0.94 (t, *J* = 7.3 Hz, 3H); ^13^C{^1^H} NMR (101 MHz, CDCl_3_) δ 146.1, 139.1, 139.0, 134.8, 134.6, 128.2, 128.0, 123.1, 121.2, 112.8, 111.9, 34.1, 30.9, 23.0, 20.5, 14.1; FTIR, *v_max_*: 2924, 2857, 1917, 1733, 1701, 1626, 1558, 1454, 1374, 1328, 1243 cm^−1^; HRMS (ESI TOF) *m*/*z* calc’d. for C_16_H_18_ClN_2_ [M+H]^+^: 273.1153, found: 273.1145 (3.0 ppm).

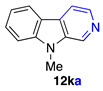



9-Methyl-β-carboline (**12ka**): This compound was prepared using Method A employing (*E*)-1,2-dimethyl-3-(2-nitrovinyl)-1*H*-indole (**10ka**) (216 mg, 1.00 mmol). Purification was performed using column chromatography (benzene/ethanol, 9:1, *v*/*v*). The titled compound was obtained as yellowish solid, m.p. 107–108 °C, R*_f_* 0.47 (benzene/ethanol, 1:9, *v*/*v*). Yield of 84 mg (0.46 mmol, 46%). ^1^H NMR (400 MHz, CDCl_3_) δ 8.89 (s, 1H), 8.48 (d, *J* = 5.2 Hz, 1H), 8.15 (d, *J* = 7.8 Hz, 1H), 7.96 (dd, *J* = 5.2, 1.0 Hz, 1H), 7.62 (ddd, *J* = 8.3, 7.2, 1.1 Hz, 1H), 7.47 (d, *J* = 8.3 Hz, 1H), 7.33–7.27 (m, 1H), 3.95 (s, 3H).; ^13^C{^1^H} NMR (101 MHz, CDCl_3_) δ 141.8, 139.1, 137.1, 132.0, 128.5, 128.4, 122.0, 121.1, 119.7, 114.6, 109.4, 29.5; FTIR, *v_max_*: 2930, 2851, 1771, 1718, 1701, 1686, 1626, 1557, 1500, 1474, 1451, 1328, 1248, 1151 cm^−1^; HRMS (ESI TOF) *m*/*z* calc’d. for C_12_H_11_N_2_ [M+H]^+^: 183.0917, found: 183.0914 (1.7 ppm).

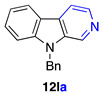



9-Benzyl-β-carboline (**12la**): This compound was prepared using Method A employing (*E*)-1-benzyl-2-methyl-3-(2-nitrovinyl)-1*H*-indole (**10la**) (292 mg, 1.00 mmol). Purification was performed using column chromatography (benzene/ethanol, 9:1, *v*/*v*). The titled compound was obtained as colorless solid, m.p. 122–123 °C, R*_f_* 0.53 (benzene/ethanol, 1:9, *v*/*v*). Yield of 103 mg (0.40 mmol, 40%). ^1^H NMR (400 MHz, CDCl_3_) δ 8.85 (s, 1H), 8.49 (d, *J* = 5.1 Hz, 1H), 8.18 (d, *J* = 7.7 Hz, 1H), 7.99 (dd, *J* = 5.3, 1.0 Hz, 1H), 7.57 (t, *J* = 7.1 Hz, 1H), 7.45 (d, *J* = 8.3 Hz, 1H), 7.34–7.25 (m, 4H), 7.15 (dd, *J* = 7.5, 2.1 Hz, 2H), 5.58 (s, 2H); ^13^C{^1^H} NMR (101 MHz, CDCl_3_) δ 141.5, 139.4, 136.8, 136.5 (2C), 132.5, 129.1 (2C), 128.7, 128.0, 126.6 (2C), 122.1, 121.4, 120.1, 114.7, 109.8, 47.0; FTIR, *v_max_*: 2963, 2924, 1736, 1719, 1698, 1686, 1651, 1623, 1562, 1539, 1504, 1467, 1441, 1330, 1247 cm^−1^; HRMS (ESI TOF) *m*/*z* calc’d. for C_18_H_15_N_2_ [M+H]^+^: 259.1230, found: 259.1229 (0.1 ppm).

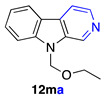



9-(Ethoxymethyl)-β-carboline (**12ma**): This compound was prepared using Method A employing (*E*)-1-(ethoxymethyl)-2-methyl-3-(2-nitrovinyl)-1*H*-indole (**10ma**) (260 mg, 1.00 mmol). The titled compound was purified using column chromatography (benzene/ ethanol, 9:1, *v*/*v*). The titled compound was obtained as yellowish solid, m.p. 79–80 °C, R*_f_* 0.57 (benzene/ethanol, 1:9, *v*/*v*). Yield of 90 mg (0.4 mmol, 40%). ^1^H NMR (400 MHz, CDCl_3_) δ 9.02 (s, 1H), 8.51 (d, *J* = 5.3 Hz, 1H), 8.14 (d, *J* = 7.0 Hz, 1H), 7.96 (d, *J* = 5.3 Hz, 1H), 7.64–7.57 (m, 2H), 7.36–7.31 (m, 1H), 5.80 (s, 2H), 3.50 (q, *J* = 7.0 Hz, 2H), 1.16 (t, *J* = 7.0 Hz, 3H); ^13^C{^1^H} NMR (101 MHz, CDCl_3_) δ 141.4, 140.1, 136.8, 132.7, 129.2, 128.8, 122.0, 121.7, 120.7, 114.7, 110.2, 73.1, 64.5, 15.1; FTIR, *v_max_*: 2960, 2871, 1734, 1714, 1684, 1651, 1626, 1562, 1510, 1489, 1474, 1462, 1376 cm^−1^; HRMS (ESI TOF) *m*/*z* calc’d. for C_14_H_15_N_2_O [M+H]^+^: 227.1179, found: 227.1178 (0.6 ppm).

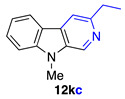



3-Ethyl-9-methyl-β-carboline (**12kc**): This compound was prepared using Method A employing (*E*)-1,2-dimethyl-3-(2-nitrobut-1-en-1-yl)-1*H*-indole (**10kc**) (244 mg, 1.00 mmol). The titled compound was purified using column chromatography (benzene/ethanol, 9:1, *v*/*v*). The titled compound was obtained as colorless solid, m.p. 45–47 °C, R*_f_* 0.47 (benzene/ethanol, 1:9, *v*/*v*). Yield of 63 mg (0.3 mmol, 30%). ^1^H NMR (400 MHz, CDCl_3_) δ 8.80 (s, 1H), 8.13 (d, *J* = 8.9 Hz, 1H), 7.83 (s, 1H), 7.60 (t, *J* = 7.8 Hz, 1H), 7.44 (d, *J* = 7.5 Hz, 1H), 7.29–7.27 (m, 1H), 3.93 (s, 3H), 3.02 (q, *J* = 7.6 Hz, 2H), 1.41 (t, *J* = 7.9 Hz, 3H); ^13^C{^1^H} NMR (101 MHz, CDCl_3_) δ 152.65, 142.30, 135.8, 130.8, 129.6, 128.4, 122.0, 121.1, 119.5, 112.4, 109.3, 31.2, 29.6, 15.1; FTFTIR, *v_max_*: 2930, 2854, 1736, 1716, 1686, 1653, 1560, 1514, 1471, 1461, 1333, 1248 cm^−1^; HRMS (ESI TOF) *m*/*z* calc’d. for C_14_H_15_N_2_ [M+H]^+^: 211.1230, found: 211.1228 (0.8 ppm).

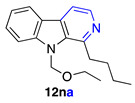



1-Butyl-9-(ethoxymethyl)-β-carboline (**12na**): This compound was prepared using Method A employing (*E*)-1-(ethoxymethyl)-3-(2-nitrovinyl)-2-pentyl1*H*-indole (**10na**) (316 mg, 1.00 mmol). The titled compound was purified using column chromatography (benzene/ethanol, 9:1, *v*/*v*). The titled compound was obtained as yellow oil, R*_f_* 0.6 (benzene/ethanol, 1:9, *v*/*v*). Yield of 71 mg (0.25 mmol, 25%). ^1^H NMR (400 MHz, DMSO-*d_6_*) 8.33 (d, *J* = 5.1 Hz, 1H), 8.24 (d, *J* = 7.8 Hz, 1H), 8.01 (d, *J* = 5.1 Hz, 1H), 7.88 (d, *J* = 8.4 Hz, 1H), 7.61 (t, *J* = 7.7 Hz, 1H), 7.30 (t, *J* = 7.5 Hz, 1H), 5.88 (s, 2H), 3.51–3.45 (m, 2H), 3.31–3.26 (m, 2H), 1.85–1.74 (m, 2H), 1.48–1.42 (m, 2H), 1.06 (t, *J* = 7.0 Hz, 3H), 0.96 (t, *J* = 7.4 Hz, 3H); ^13^C{^1^H} NMR (101 MHz, DMSO-*d_6_*) δ 146.7, 142.3, 139.3, 134.2, 129.8, 128.9, 122.0, 121.3, 120.8, 113.1, 111.0, 73.7, 63.5, 34.4, 31.6, 22.7, 15.4, 14.4; FTIR, *v_max_*: 2969, 2831, 1719, 1684, 1651, 1514, 1451, 1328, 1248, 1151 cm^−1^; HRMS (ESI TOF) *m*/*z* calc’d. for C_18_H_23_N_2_O [M+H]^+^: 283.1805, found: 283.1813 (−2.7 ppm).

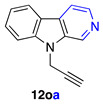



9-(Prop-2-yn-1-yl)-9*H*-pyrido [3,4-b]indole (**12oa**): This compound was prepared using Method A employing (*E*)-2-methyl-3-(2-nitrovinyl)-1-(prop-2-yn-1-yl)-1*H*-indole (**10oa**) (240 mg, 1 mmol) with the yield of 82 mg (0.40 mmol, 40%). Purification was performed using column chromatography (EtOAc/hexane, 1:1, *v*/*v*). The titled compound was obtained as white solid, m.p. 120 °C (EtOH), lit [47]. 113 °C, R*_f_* 0.59 (EtOAc). ^1^H NMR (400 MHz, DMSO-*d*_6_) δ 9.13 (s, 1H), 8.44 (d, *J* = 5.2 Hz, 1H), 8.29 (d, *J* = 7.8 Hz, 1H), 8.17 (d, *J* = 5.2 Hz, 1H), 7.80 (d, *J* = 8.3 Hz, 1H), 7.65 (t, *J* = 7.7 Hz, 1H), 7.33 (t, *J* = 7.4 Hz, 1H), 5.45 (d, *J* = 2.4 Hz, 2H), 3.34 (t, *J* = 2.4 Hz, 1H). ^13^C NMR (101 MHz, DMSO) δ 140.4, 139.0, 135.7, 132.8, 128.6, 128.0, 122.1, 120.8, 120.2, 114.8, 110.5, 78.8, 75.1, 32.2. IR, *v_max_*: 3296, 2925, 2857, 1621, 1558, 1499, 1468, 1456, 1445, 1415, 1325, 1211 cm^−1^; HRMS (ESI TOF) *m*/*z* calcd. for C_14_H_10_N_2_Na [M+Na]^+^: 229.0736, found: 229.0731 (2.2 ppm).

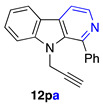



1-Phenyl-9-(prop-2-yn-1-yl)-9*H*-pyrido[3,4-b]indole (**12pa**): This compound was prepared using Method A employing (*E*)-2-benzyl-3-(2-nitrovinyl)-1-(prop-2-yn-1-yl)-1*H*-indole (**10pa**) (316 mg, 1 mmol) with the yield of 116 mg (0.41 mmol, 41%). Purification using column chromatography (EtOAc/hexane, 1:1, *v*/*v*). The titled compound was obtained as a white solid, m.p. 151–152 °C (EtOH), R*_f_* 0.81 (EtOAc). ^1^H NMR (400 MHz, chloroform-*d*) δ 8.56 (d, *J* = 5.2 Hz, 1H), 8.19 (d, *J* = 7.8 Hz, 1H), 7.99 (d, *J* = 5.2 Hz, 1H), 7.77–7.72 (m, 2H), 7.68–7.62 (m, 1H), 7.61–7.53 (m, 4H), 7.39–7.35 (m, 1H), 4.64 (d, *J* = 2.4 Hz, 2H), 2.22 (t, *J* = 2.4 Hz, 1H).^13^C NMR (101 MHz, CDCl_3_) δ 144.0, 142.3, 139.0, 134.0, 131.2, 129.5 (2C), 129.0, 129.0, 128.7 (2C), 121.9, 121.8, 120.9, 113.9, 111.9, 110.7, 78.1, 73.0, 35.0. IR, *v_max_*: 3221, 2930, 2841, 1611, 1547, 1473, 1468, 1415, 1325, 1211 cm^−1^; HRMS (ESI TOF) *m*/*z* calcd. for C_20_H_14_N_2_Na [M+Na]^+^: 305.1049, found: 305.1055 (−2.0 ppm).

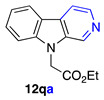



Ethyl 2-(9*H*-pyrido[3,4-b]indol-9-yl)acetate (**12qa**): This compound was prepared using Method A employing (*E*)-ethyl 2-(2-methyl-3-(2-nitrovinyl)-1*H*-indol-1-yl)acetate (**10qa**) (288 mg, 1.00 mmol), Purification was performed using column chromatography (EtOAc/hexane, 1:2, *v*/*v*, EtOAc). The titled compound was obtained as colorless oil, R*f* 0.33 (EtOAc). Yield of 89 mg (0.35 mmol, 35%). ^1^H NMR (400 MHz, chloroform-*d*) δ 8.83 (s, 1H), 8.50 (d, *J* = 5.3 Hz, 1H), 8.16 (d, *J* = 7.8, 1.0 Hz, 1H), 7.98 (dd, *J* = 5.3, 1.1 Hz, 1H), 7.61 (m, 1H), 7.41 (dt, *J* = 8.3, 0.9 Hz, 1H), 7.33 (m, 1.0 Hz, 1H), 5.08 (s, 2H), 4.22 (q, *J* = 7.1 Hz, 2H), 1.24 (t, *J* = 7.1 Hz, 3H); ^13^C NMR (101 MHz, CDCl_3_) δ 168.0, 141.5, 139.6, 136.8, 131.7, 129.2, 128.9, 122.2, 121.5, 120.6, 114.9, 109.33, 62.1, 45.0, 14.3. IR, *v_max_*: 2969, 2852, 1732, 1650, 1458, 1365, 1328, 1241, 1198, 1016 cm^−1^; HRMS (ESI TOF) *m*/*z* calcd. for C_15_H_15_N_2_O_2_ [M+H]^+^: 255.1128, found: 255.1133 (−2.0 ppm).

### 3.4. Synthesis of β-Carbolines with the Reaction of Indoles with 4-(2-Nitrovinyl)morpholine as Shown in Figure 8

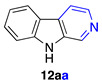



9*H*-Pyrido[3,4-*b*]indole (12aa): Typical Procedure H (as shown in Figure 8) involved employing 2-methyl-1H-indole (**14a**) (131 mg, 1.00 mmol), 4-(2-nitrovinyl)morpholine (**15a**) (316 mg, 2.00 mmol), 2,2,2-trichloroacetic acid (408 mg, 2.5 mmol) in 2 mL isoamyl alcohol. These starting materials and reagents were placed in a microwave reactor. The reactor was heated in the microwave apparatus at 70 °C for 0.5 h; then, the temperature was quickly raised to 200 °C, and the reaction mixture was kept at this temperature for additional 0.8 h. After completion of the reaction, the reaction mixture was removed from the microwave reactor and concentrated in vacuo. Crude material was purified using column chromatography (EtOAc/hexane, 1:3, *v*/*v*, EtOAc). The titled compound was obtained as light-yellow solid, m.p. 197–198 °C, R*_f_* 0.56 (benzene/isopropanol, 1:1, *v*/*v*). Yield of 71 mg (0.42 mmol, 42%). ^1^H NMR (400 MHz, Chloroform-*d*) δ 9.52 (br.s, 1H), 8.97 (s, 1H), 8.43 (d, *J* = 5.4 Hz, 1H), 8.13 (d, *J* = 7.9 Hz, 1H), 7.98 (d, *J* = 5.3 Hz, 1H), 7.57–7.53 (m, 2H), 7.30 (m, 1H); ^13^C NMR (101 MHz, CDCl_3_) δ 141.0, 138.0, 136.1, 133.1, 129.5, 129.0, 122.0, 121.3, 120.3, 115.1, 112.0; IR, *v_max_*: 3384, 2937, 2364, 2321, 1653, 1621, 1557, 1529, 1472, 1444, 1417 cm^−1^; HRMS (ESI TOF) *m*/*z* calcd. for C_11_H_9_N_2_ [M+H]^+^: 169.0760, found: 169.0765 (−3.0 ppm).

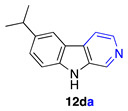



6-Isopropyl-9*H*-pyrido[3,4-*b*]indole (**12da**): This compound was prepared using Typical Procedure H employing 5-isopropyl-2-methyl-1*H*-indole (**14d**) (173 mg, 1.00 mmol), 4-(2-nitrovinyl)morpholine (**15a**) (316 mg, 2.00 mmol), 2,2,2-trichloroacetic acid (408 mg, 2.5 mmol) in 2 mL isoamyl alcohol. Purification was performed using column chromatography (EtOAc/hexane, 1:1, *v*/*v*, EtOAc). The titled compound was obtained as brown solid, m.p. 172–175 °C, R*_f_* 0.18 (EtOAc). Yield of 63 mg (0.30 mmol, 30%). ^1^H NMR (400 MHz, chloroform-*d*) δ 8.89 (s, 1H), 8.45 (d, *J* = 5.3 Hz, 1H), 8.29 (br.s, 1H), 7.98 (s, 1H), 7.96 (d, *J* = 5.3 Hz, 1H), 7.47–7.43 (m, 2H), 3.11 (m, 1H), 1.36 (d, *J* = 7.0 Hz, 6H); ^13^C{^1^H} NMR (101 MHz, CDCl_3_) δ 141.2, 139.2, 139.0, 136.3, 133.8, 129.1, 128.0, 121.7, 118.9, 114.8, 111.5, 34.3, 24.74 (2C). IR, *v_max_*: 3133, 3036, 2960, 1635, 1554, 1498, 1461, 1269, 1250 cm^−1^; HRMS (ESI TOF) *m*/*z* calcd. for C_14_H_15_N_2_ [M+H]^+^: 211.1230, found: 211.1228 (1.0 ppm).

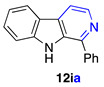



1-Phenyl-9*H*-pyrido[3,4-b]indole (**12ia**): This compound was prepared using Typical Procedure H employing 2-benzyl-1*H*-indole (**14i**) (207 mg, 1.00 mmol), 4-(2-nitrovinyl)morpholine (**15a**) (316 mg, 2.00 mmol), 2,2,2-trichloroacetic acid (408 mg, 2.5 mmol) in 2 mL isoamyl alcohol. Purification was performed using column chromatography (EtOAc/hexane, 1:2, *v*/*v*, EtOAc). The titled compound was obtained as light-yellow solid m.p. 240–242 °C, R*_f_* 0.71 (EtOAc). Yield of 98 mg (0.40 mmol, 40%). ^1^H NMR (400 MHz, chloroform-*d*) δ 8.65 (br.s, 1H), 8.57 (d, *J* = 5.3 Hz, 1H), 8.17 (d, *J* = 7.9 Hz, 1H), 7.95 (dd, *J* = 6.4, 3.4 Hz, 3H), 7.56 (m, 3H), 7.51–7.47 (m, 2H), 7.32 (dd, *J* = 8.0, 6.8 Hz, 1H); ^13^C NMR (101 MHz, CDCl_3_) δ 143.1, 140.5, 139.6, 138.6, 133.6, 129.98, 129.35 (2C), 128.99, 128.67, 128.26, 121.99, 121.96, 120.4, 111.0, 111.7; IR, *v_max_*: 924, 2857, 1621, 1558, 1499, 1456, 1445, 1415, 1325 cm^−1^; HRMS (ESI TOF) *m*/*z* calcd. for C_17_H_13_N_2_ [M+H]^+^: 245.1073, found 245.1067 (2.5 ppm).

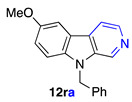



9-Benzyl-6-methoxy-9*H*-pyrido[3,4-b]indole (**12ra**): This compound was prepared using Typical Procedure H employing 1-benzyl-5-methoxy-2-methyl-1*H*-indole (**14r**) (251 mg, 1.00 mmol), 4-(2-nitrovinyl)morpholine (**15a**) (316 mg, 2.00 mmol), 2,2,2-trichloroacetic acid (408 mg, 2.5 mmol) in 2 mL isoamyl alcohol. Purification was performed using column chromatography (EtOAc/hexane, 1:2, *v*/*v*, EtOAc). The titled compound was obtained as brown oil, R*_f_* 0.38 (EtOAc). Yield of 90 mg (0.31 mmol, 31%). ^1^H NMR (400 MHz, CDCl_3_) δ 8.83 (br. s, 1H), 8.45 (d, *J* = 5.2 Hz, 1H), 7.95 (d, *J* = 5.3 Hz, 1H), 7.62 (d, *J* = 2.6 Hz, 1H), 7.35- (m, 1H), 7.27–7.30 (d, *J* = 3.3 Hz, 1H), 7.25–7.20 (m, 2H), 7.13 (dd, *J* = 6.2, 1.9 Hz, 2H), 5.56 (s, 2H), 3.94 (s, 3H); ^13^C NMR (101 MHz, CDCl_3_) δ 154.3, 138.8, 137.2, 136.7, 136.5, 132.6, 129.1 (2C), 128.3, 127.9, 126.6 (2C), 121.6, 118.6, 114.7, 110.8, 104.0, 56.2, 47.2; IR, *v_max_*: 2953, 2841, 1621, 1559, 1454, 1292, 1226, 1163 cm^−1^; HRMS (ESI TOF) *m*/*z* calcd. for C_19_H_17_N_2_O [M+H]^+^: 289.1335, found: 289.1332 (1.0 ppm).

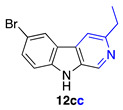



6-Bromo-3-ethyl-9*H*-pyrido[3,4-b]indole (**12cc**): This compound was prepared using Typical Procedure H employing 5-bromo-2-methyl-1*H*-indole (**14c**) (210 mg, 1.00 mmol), 4-(2-nitrobut-1-en-1-yl)morpholine (**15c**) (372 mg, 2.00 mmol), 2,2,2-trichloroacetic acid (408 mg, 2.5 mmol) in 2 mL isoamyl alcohol. Purification was performed using column chromatography (EtOAc/hexane, 1:2, *v*/*v*, EtOAc). The titled compound was obtained as a light-yellow oil, R*_f_* 0.2 (EtOAc). Yield of 75 mg (0.27 mmol, 27%). ^1^H NMR (400 MHz, CDCl_3_) δ 9.85 (br. S, 1H), 8.91 (s, 1H), 8.20 (d, *J* = 2.0 Hz, 1H), 7.76 (s, 1H), 7.59 (d, *J* = 9.0 Hz, 1H), 7.40 (d, *J* = 8.7 Hz, 1H), 3.03 (q, *J* = 7.7 Hz, 2H), 1.40 (t, *J* = 7.6 Hz, 3H); ^13^C NMR (101 MHz, CDCl_3_) δ 151.7, 140.3, 134.8, 131.9, 131.65, 130.1, 124.7, 122.8, 113.5, 113.0, 112.9, 30.3, 14.8; IR, *v_max_*: 3143, 2961, 2848, 1646, 1489, 1447, 1276, 1111, 1049, 2026 cm^−1^; HRMS (ESI TOF) *m*/*z* calcd. For C_13_H_12_BrN_2_ [M+H]^+^: 275. 0179, found: 275. 0185 (−2.2 ppm).

### 3.5. Synthesis of β-Carbolines Using the Henry Reaction of Indole-3-carbaldehydes as Shown in Figure 9

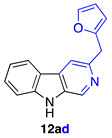



3-(Furan-2-ylmethyl)-9*H*-pyrido[3,4-b]indole (**12ad**): Typical Procedure I (Figure 9) involved employing 2-methyl-1*H*-indole-3-carbaldehyde (**16**) (159 mg, 1.00 mmol), 2-(2-nitroethyl)furan (**17d**) (282 mg, 2.00 mmol), ammonium acetate (8 mg, 0.1 mmol) in *i*-AmOH (2 mL). These were charged in a G10 vial. The mixture was sealed and heated in the microwave oven at 200 °C for 1 h. After completion of the reaction, water (20 mL) was added, followed by extraction with EtOAc (4 × 20 mL). Combined organic layers were concentrated in vacuo. Purification was performed using column chromatography (EtOAc/hexane, 1:2, *v*/*v*, EtOAc). The titled compound was obtained as brown oil, R*_f_* 0.25 (EtOAc). Yield of 20 mg (0.08 mmol, 8%), 85% of 2-methyl-1*H*-indole-3-carbaldehyde (**16**) was recycled. ^1^H NMR (400 MHz, chloroform-*d*) δ 9.48 (s, 1H), 9.02 (s, 1H), 8.09 (d, *J* = 7.9 Hz, 1H), 7.87 (s, 1H), 7.56 (d, *J* = 5.4 Hz, 2H), 7.41–7.34 (m, 1H), 7.31–7.27 (m, 1H), 6.33 (t, *J* = 2.6 Hz, 1H), 6.20 (d, *J* = 3.2 Hz, 1H), 4.41 (s, 2H); ^13^C NMR (101 MHz, CDCl_3_) δ 142.0, 141.9, 134.7 (2C), 131.5, 131.2, 129.6, 123.2, 122.2, 121.0, 120.5, 114.4, 112.2, 110.7, 107.3, 35.8. IR, *v_max_*: 3407, 1774, 1654, 1507 1239, 1024, 995 cm^−1^; HRMS (ESI TOF) *m*/*z* calcd. for C_16_H_12_N_2_NaO [M+Na]^+^: 271.0842, found: 271.0838 (1.5 ppm).

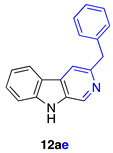



3-Benzyl-9*H*-pyrido[3,4-b]indole (**12ae**): This compound was prepared using Typical Procedure I employing 2-methyl-1*H*-indole-3-carbaldehyde (**16**) (159 mg, 1.00 mmol), (2-nitroethyl)benzene (**17e**) (477 mg, 3.00 mmol), ammonium acetate (8 mg, 0.1 mmol) in *i*-AmOH (2 mL). Purification was performed using column chromatography (EtOAc/hexane, 1:2, *v*/*v*, EtOAc). The titled compound was obtained as colorless oil, R*_f_* 0.37 (EtOAc). Yield of 16 mg (0.06 mmol, 6%), 75% of 2-methyl-1*H*-indole-3-carbaldehyde (**16**) was recycled. ^1^H NMR (400 MHz, Chloroform-*d*) δ 9.26 (br. S, 1H), 8.87 (d, *J* = 1.0 Hz, 1H), 8.07 (d, *J* = 1.0 Hz, 1H), 7.80 (d, *J* = 1.0 Hz, 1H), 7.54 (m, 1H), 7.48 (m, 1H), 7.322–7.20 (m, 5H), 4.38 (s, 2H); ^13^C NMR (101 MHz, CDCl_3_) δ 149.6, 141.4, 140.5, 134.7, 132.3, 130.6, 129.2 (2C), 128.9, 128.7 (2C), 126.4, 122.0, 121.3, 120.1, 114.1, 111.9, 44.0; IR, *v_max_*: 3647, 2973, 1868, 1771, 1699, 1678, 1643, 1557, 1522, 1474, 1245, 1047 cm^−1^; HRMS (ESI TOF) *m*/*z* calcd. For C_18_H_15_N_2_ [M+H]^+^: 259.1230, found: 259.1235 (−1.9 ppm).

### 3.6. Synthesis of β-Carbolines Involving the Formation of Spiro[indole-3,5′-isooxazoles] In Situ as Shown in Figure 11

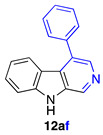



4-Phenyl-9*H*-pyrido[3,4-b]indole (**12af**): Typical Procedure K (Figure 11) involved charging 2-methyl-1*H*-indole (**14a**) (131 mg, 1 mmol), (*E*)-(2-nitrovinyl)benzene (**22f**) (164 mg, 1.1 mmol), AcOH (1 g), and H_3_PO_3_ (1 g) in a G30 vial. The mixture was stirred at room temperature for 0.5 h. After that, the mixture was sealed and heated in a microwave oven at 200 °C for 1 h. After completion of the reaction, water (50 mL) was added, followed by extraction with EtOAc (4 × 20 mL). Combined organic layers were concentrated in vacuo. Purification was performed using column chromatography (EtOAc/hexane, 1:1, *v*/*v*). The titled compound was obtained as a light-yellow solid, m.p. 262–263 °C (EtOH), lit. 259–260 °C [48]. The yield was 61 mg (0.25 mmol, 25%). R*_f_* 0.34 (EtOAc). ^1^H NMR (400 MHz, chloroform-*d*) δ 9.82 (s, 1H), 9.06 (s, 1H), 8.26 (s, 1H), 7.66 (d, *J* = 8.1 Hz, 1H), 7.64–7.58 (m, 3H), 7.57–7.51 (m, 4H), 7.08 (t, *J* = 7.2 Hz, 1H). ^13^C NMR (101 MHz, CDCl_3_) δ 141.6, 137.1, 136.6, 135.9, 132.6, 130.8, 129.4 (2C), 129.3, 128.9 (2C), 128.7, 127.8, 123.9, 120.8, 120.3, 112.1. IR, *v_max_*: 2925, 2851, 1611, 1510, 1477, 1445, 1401, 1325 cm^−1^; HRMS (ESI TOF) *m*/*z* calcd. for C_17_H_12_N_2_Na [M+Na]^+^: 267.0893, found: 267.0898 (−1.9 ppm).

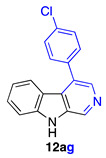



4-(4-Chlorophenyl)-9*H*-pyrido[3,4-b]indole (**12ag**): This product was obtained using Typical Procedure K employing 2-methyl-1*H*-indole (**14a**) (131 mg, 1 mmol), (*E*)-1-chloro-4-(2-nitrovinyl)benzene (**22g**) (202 mg, 1.1 mmol) with the yield of 59 mg (0.21 mmol, 21%). Purification was performed using column chromatography (EtOAc/hexane, 1:1, *v*/*v*). The titled compound was obtained as white solid, m.p. 254–255 °C (EtOH), lit. 251–253 °C [48], R*_f_* 0.31 (EtOAc). ^1^H NMR (400 MHz, DMSO-*d*_6_) δ 11.96 (s, 1H), 8.96 (s, 1H), 8.21 (s, 1H), 7.69–7.63 (m, 5H), 7.57–7.52 (m, 2H), 7.11–7.07 (m, 1H).^13^C NMR (101 MHz, DMSO- *d*_6_) δ 141.0, 137.7, 136.3, 135.9, 133.2, 133.1, 131.0 (2C), 129.8, 128.9 (2C), 128.3, 124.7, 122.6, 119.8, 119.4, 112.3. IR, *v_max_*: 2921, 1911, 1711, 1642, 1543, 1311, 1243, 751 cm^−1^; HRMS (ESI TOF) *m*/*z* calcd. for C_17_H_11_ClN_2_Na [M+Na]^+^: 301.0503, found: 301.0506 (−1.0 ppm).

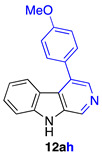



4-(4-Methoxyphenyl)-9*H*-pyrido[3,4-b]indole (**12ah**): This product was obtained using Typical Procedure K employing 2-methyl-1*H*-indole (**14a**) (131 mg, 1 mmol), (*E*)-1-methoxy-4-(2-nitrovinyl)benzene (**22h**) (197 mg, 1.1 mmol) with the yield of 41 mg (0.15 mmol, 15%). Purification was performed using column chromatography (EtOAc/hexane, 1:1, *v*/*v*). The titled compound was obtained as white solid, m.p. 271–273 °C (EtOH), lit. >250 °C [49], R*_f_* 0.29 (EtOAc). ^1^H NMR (400 MHz, DMSO-*d*_6_) δ 11.82 (s, 1H), 8.88 (s, 1H), 8.17 (s, 1H), 7.65–7.57 (m, 4H), 7.52–7.48 (m, 1H), 7.18–7.14 (m, 2H), 7.06 (t, *J* = 7.5 Hz, 1H), 3.87 (s, 3H).^13^C NMR (101 MHz, DMSO*-d*_6_) δ 159.3, 140.8, 138.3, 136.0, 132.8, 130.8, 130.3 (2C), 129.7, 127.9, 124.7, 122.6, 120.2, 119.1, 114.3 (2C), 112.1, 55.3. IR, *v_max_*: 3055, 2959, 1611, 1510, 1477, 1445, 1378, 1241, 1183 cm^−1^; HRMS (ESI TOF) *m*/*z* calcd. for C_18_H_14_N_2_NaO [M+Na]^+^: 297.0998, found: 297.0992 (2.0 ppm).

## 4. Conclusions

We have developed a number of strategies to access the β-carboline skeleton, common in drug discovery and present in numerous biologically active natural products. Our methodologies are based on the microwave-assisted electrocyclic cyclization of heterotrienic aci forms of 2-nitrovinylindoles. These can start with 3-nitrovinylindoles themselves or use several different methods allowing for the formation of 3-nitrovinylindoles in situ. The main advantage of our methodologies is the simplicity and brevity of the synthetic sequences, in many cases involving just one step from commercially available materials. This allows for easy scale-up preparation of β-carbolines with a required substitution pattern. The problems that still require further work are the moderate yield of many of our procedures and the high temperatures required to initiate the electrocyclization process. However, given the complexity of the reaction paths and the simplicity of the experimentation, as well as the multistep nature of the previously developed methodologies to access β-carbolines, the advantages outweigh these challenges by far. Further work in this area involves the utilization of the developed synthetic pathways to synthesize larger collections of compounds for biological studies, as well as the preparation of compounds designed to target specific biological receptors. The results of these investigations will be reported in due course.

## Figures and Tables

**Table 1:** Optimization of reaction conditions for the transformation of 2-nitrovinylindole **10aa** to β-carboline **12aa**.

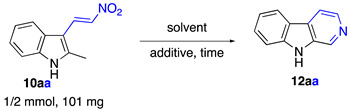					
#	Time, h	t, °C	Solvent	Additive	Yield
1	1	200 (MW)	1-butanol, 1 mL	-	49%
2	0.5	220 (MW)	1-butanol, 1 mL	-	47%
3	1	200 (MW)	ethylene glycol, 1 mL	-	32%
4	1	200 (MW)	undecane, 2 mL	-	0
5	1	200 (MW)	DMSO, 1 mL	-	45%
6	1	200 (MW)	quinoline, 1 mL	-	47%
7	1	160 (MW)	py•HCl, 2 g	-	50%
8	1	200 (MW)	H_2_O, 1 mL	-	35%
9	1	200 (MW)	no solvent	-	24%
10	1	200 (MW)	1-butanol, 1 mL	P(OMe)_3_ (124 mg, 1 mmol)	0
11	1	200 (MW)	1-butanol, 1 mL	Na_2_S_2_O_3_ (158 mg, 1 mmol)	0
12	1	200 (MW)	1-butanol, 1 mL	SnCl_2_ (190 mg, 1 mmol)	20%
13	1	200 (MW)	1-butanol, 1 mL	NH_4_Cl (212 mg, 4 mmol), Zn (64 mg, 1 mmol)	12%
14	1	200 (MW)	1-butanol, 1 mL	2-methylindene (131 mg, 1 mmol)	27%
15	1	200 (MW)	DMSO, 1 mL	β-nitrostyrene (75 mg, 0.5 mmol)	38%
16	1	200 (MW)	Ac_2_O, 1 mL	-	30%
17	1	200 (MW)	1-butanol, 1 mL	AcCl (78.5 mg, 1 mmol)	21%
18	1	200 (MW)	1-butanol, 1 mL	TsCl•H_2_O (190 mg, 1 mmol)	10%

**Table 2:** Optimization of conditions for the reaction of 2-methylindole (**14a**) and 1-(tertiary)amino-2-nitroethylenes **15**.

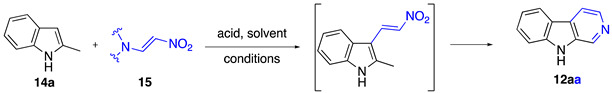					
#	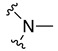	Acid	Solvent	Conditions	Yield
1	4-morpholine, 1 eq	CH3COOH, 1 eq	1-butanol	MW at 200 °C, 0.5 h	27%
2	4-morpholine, 1 eq	CH3COOH, 1 eq	xylene	MW at 200 °C, 0.5 h	21%
3	4-morpholine, 1 eq	CH_3_COOH, 1 eq	DMF	MW at 200 °C, 0.5 h	24%
4	4-morpholine, 2 eq	NH_2_SO_3_H, 2 eq	isoamyl alcohol	MW at 70 °C for 0.5 h, 200 °C for 0.5 h	20%
5	4-morpholine, 2 eq	CCl_3_COOH, 1 eq	isoamyl alcohol	MW at 70 °C for 0.5 h, 200 °C for 0.5 h	38%
6	4-morpholine, 2 eq	CCl_3_COOH, 2.5 eq	isoamyl alcohol	MW at 70 °C for 0.5 h, 200 °C for 0.8 h	42%
7	4-morpholine, 2 eq	MsOH, 1 eq	isoamyl alcohol	MW at 70 °C for 0.5 h, 200 °C for 0.5 h	27%
8	*N*,*N*-dimethylamine, 1.5 eq	CCl_3_COOH, 2 eq	isoamyl alcohol	MW at 70 °C for 0.5 h, 200 °C for 0.5 h	27%
9	*N*,*N*-dimethylamine, 2 eq	CCl_3_COOH, 3 eq	isoamyl alcohol	MW at 70 °C for 0.5 h, 200 °C for 0.8 h	35%
10	4-morpholine, 1.5 eq	CF_3_COOH, 1.5 eq	isoamyl alcohol	MW at 70 °C for 0.5 h, 200 °C for 0.5 h	30%

## Data Availability

The data presented in this study are available in the article and its Supplementary Materials.

## Supplementary Materials

The following supporting information can be downloaded at: https://www.mdpi.com/article/10.3390/ijms241713107/s1.
