# Supplementary material for: Synthesis of β-Carbolines with Electrocyclic Cyclization of 3-Nitrovinylindoles
Source: Int J Mol Sci. 2023 Aug 23;24(17):13107. doi: 10.3390/ijms241713107 (PMC10487476; doi:10.3390/ijms241713107)
Supplement: Supplementary file 1 [file ijms-24-13107-s001.zip › ijms-2573963-supplementary.pdf]

Synthesis of β-Carbolines through an Electrocyclic Cyclization of 3-Nitrovinylindoles

Nicolai A. Aksenov,[1,]* Nikolai A. Arutiunov,[1] Alexander V. Aksenov,[1] Nikita K. Kirilov,[1] Inna V. Aksenova,[1] Dmitrii A. Aksenov,[1] Elena V. Aleksandrova,[1] Michael Rubin[1] and Alexander Kornienko[2,]*

[1] Department of Chemistry, North Caucasus Federal University, 1a Pushkin St., Stavropol 355009, Russia

[2] Department of Chemistry and Biochemistry, Texas State University, 601 University Dr., San Marcos, Texas 78666, USA

* To whom correspondence should be addressed: naksenov@ncfu.edu; a_k76@txstate.edu

Supplementary Materials

NMR Spectral Charts... S2
^{1}H NMR Spectral Charts for starting (2-nitrovinyl)benzenes (**18**).. S2
^{1}H and ^{13}C Spectral Charts for starting indole (**14r**) S4
^{1}H and ^{13}C {^{1}H} Spectral Charts for starting 3-nitrovinylindoles **(10)** S6
^{1}H and ^{13}C {^{1}H} Spectral Charts for 9H-pyrido[3,4-b]indoles (**12**)…………………………………………………………… S27

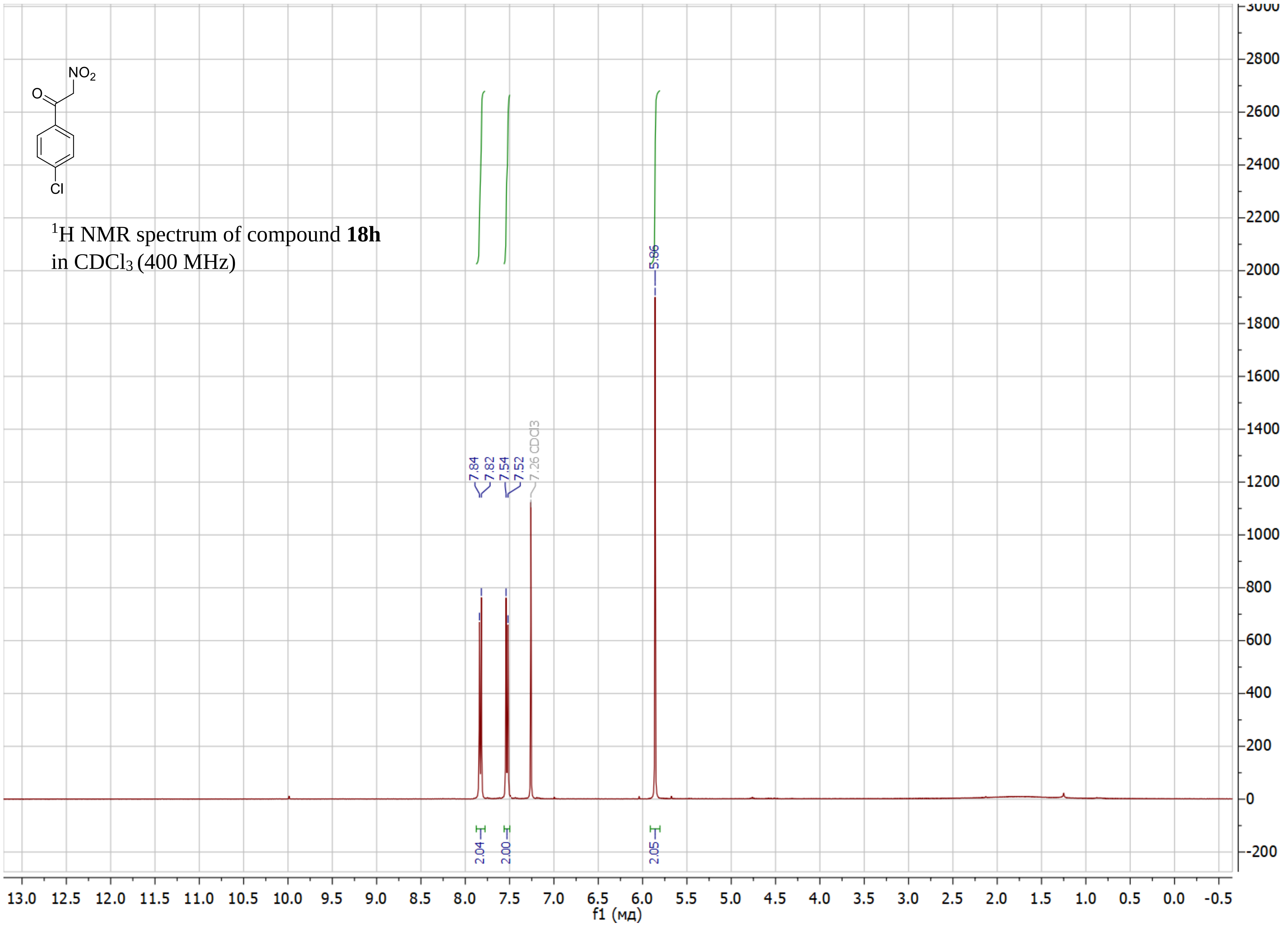

^{1}H NMR spectrum of compound **18h** in $CDCl_3$ (400 MHz)

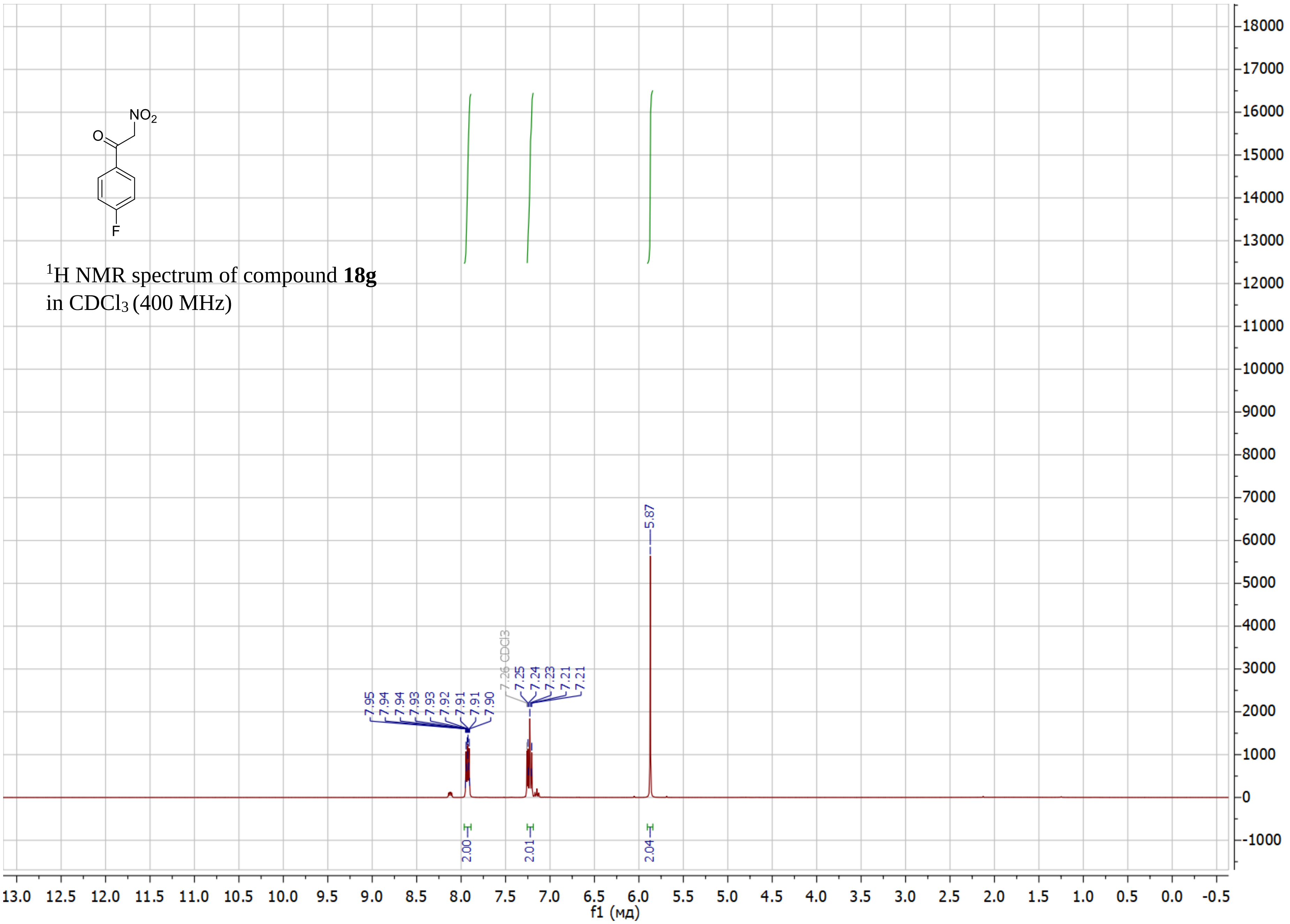

^{1}H NMR spectrum of compound **18g** in $CDCl_3$ (400 MHz)

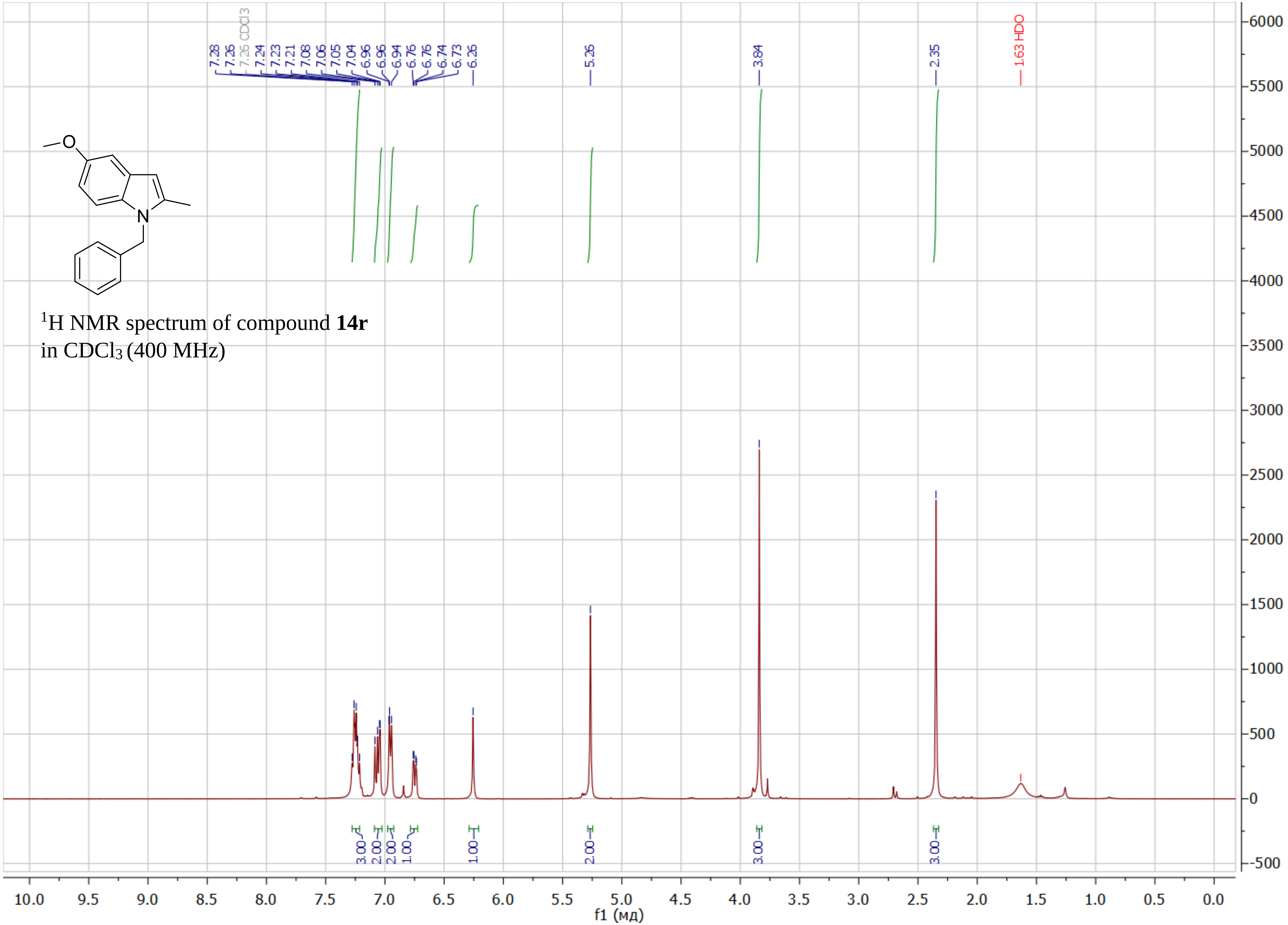

^{1}H NMR spectrum of compound **14r**
in $CDCl_3$ (400 MHz)

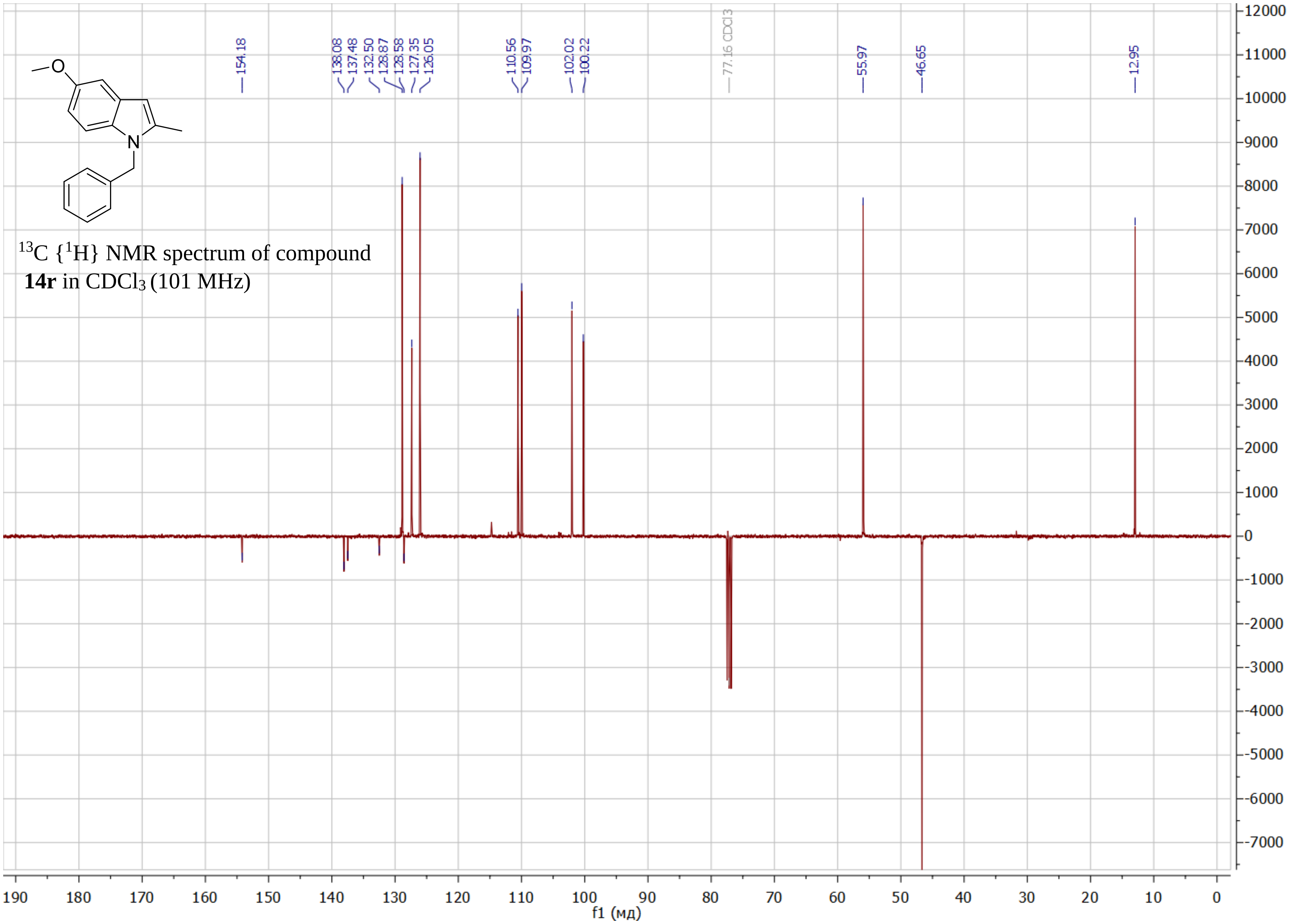

$^{13}C\ \{^1H\}$ NMR spectrum of compound **14r** in $CDCl_3$ (101 MHz)

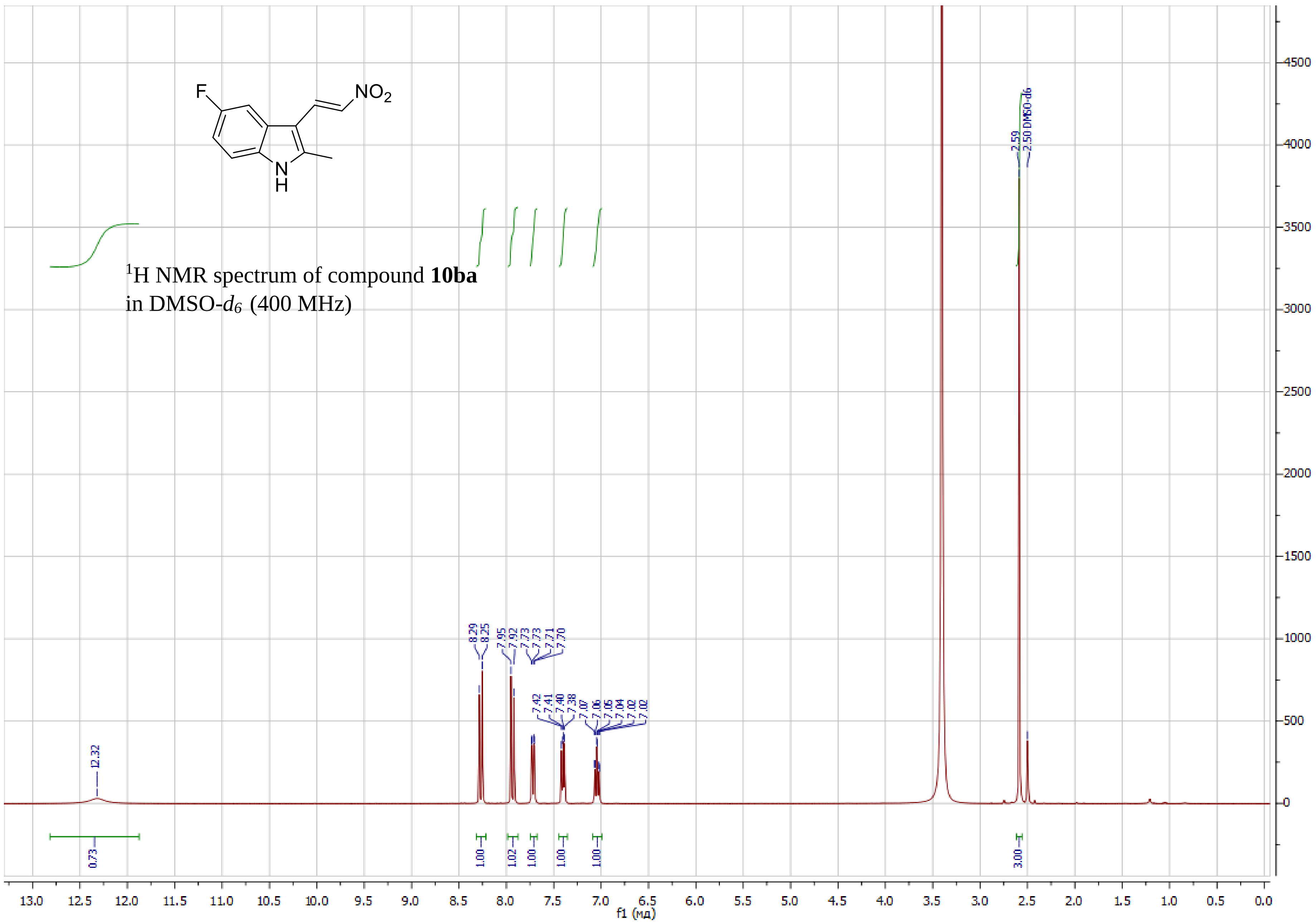

^{1}H NMR spectrum of compound **10ba** in DMSO-d_6 (400 MHz)

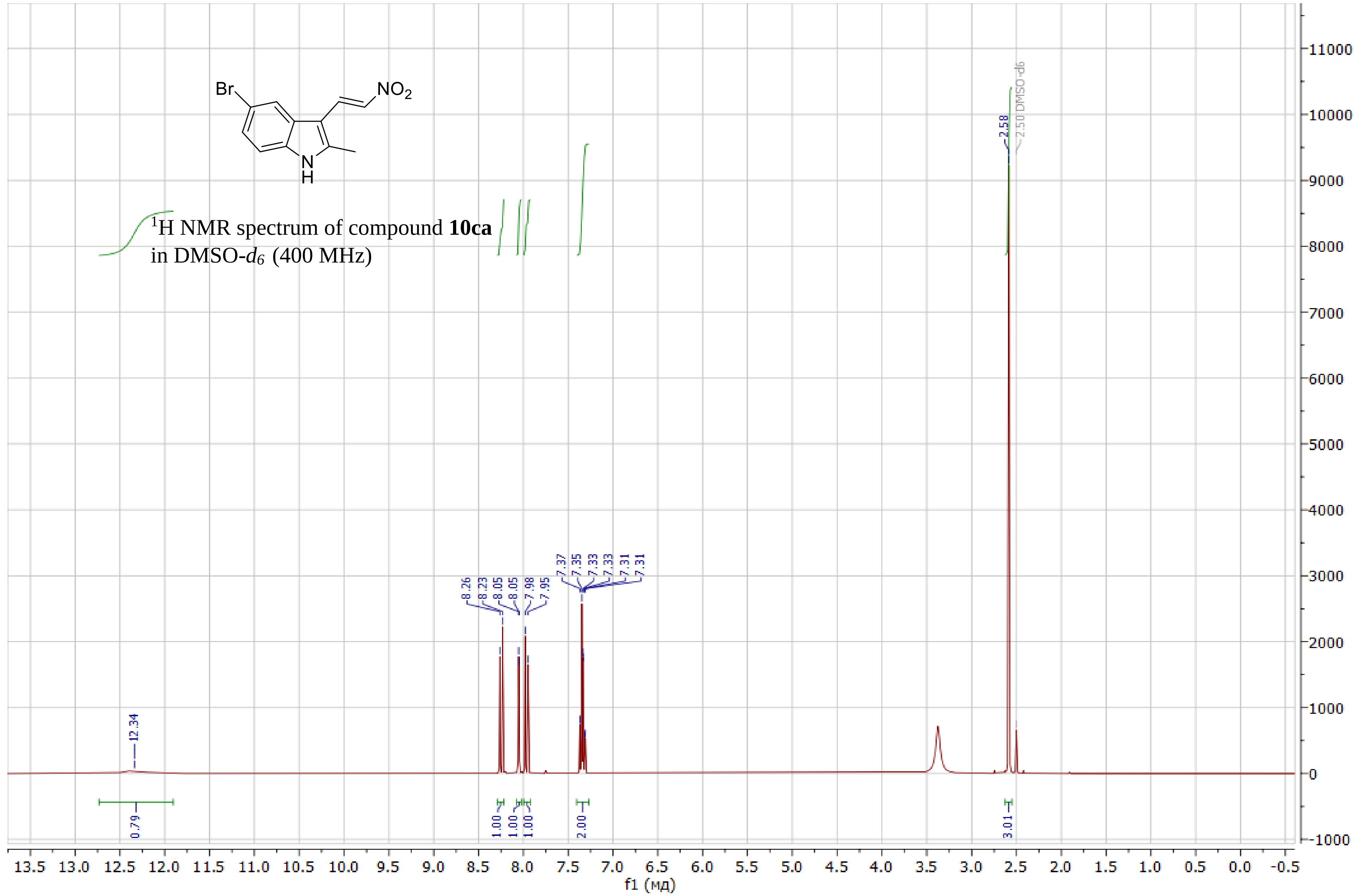

^{1}H NMR spectrum of compound **10ca** in DMSO-d_6 (400 MHz)

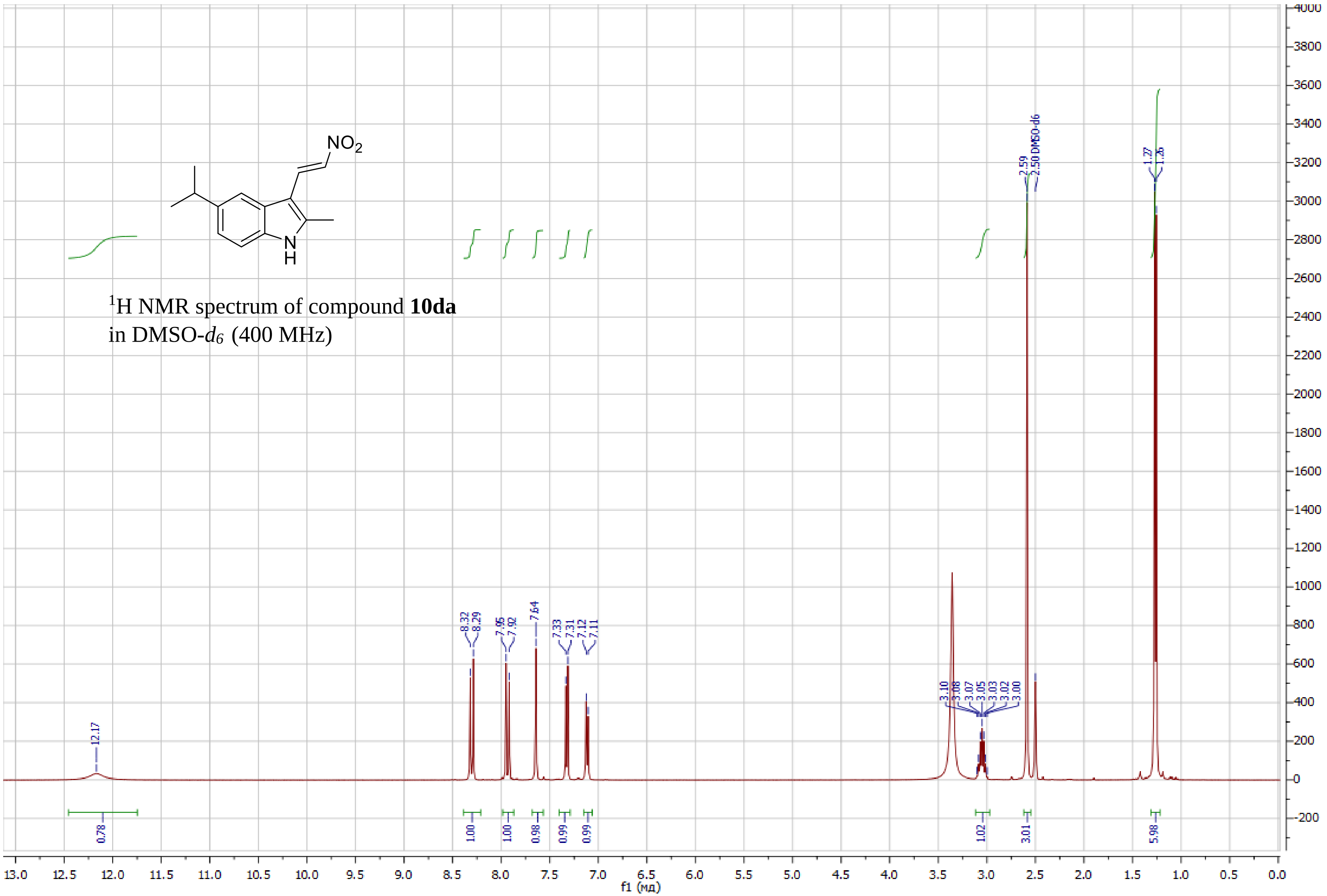

^{1}H NMR spectrum of compound **10da** in DMSO-d_6 (400 MHz)

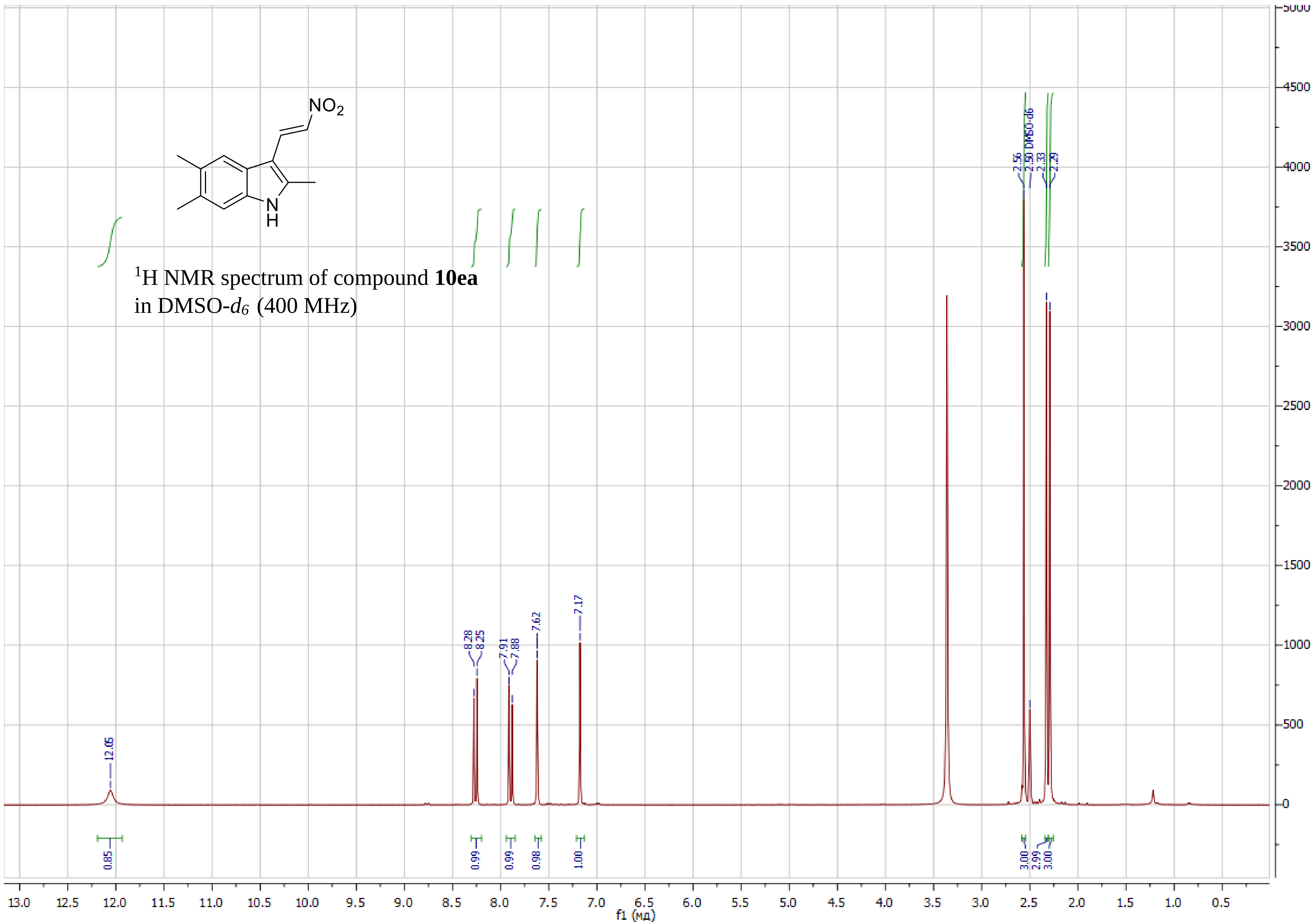

^{1}H NMR spectrum of compound **10ea** in DMSO-d_6 (400 MHz)

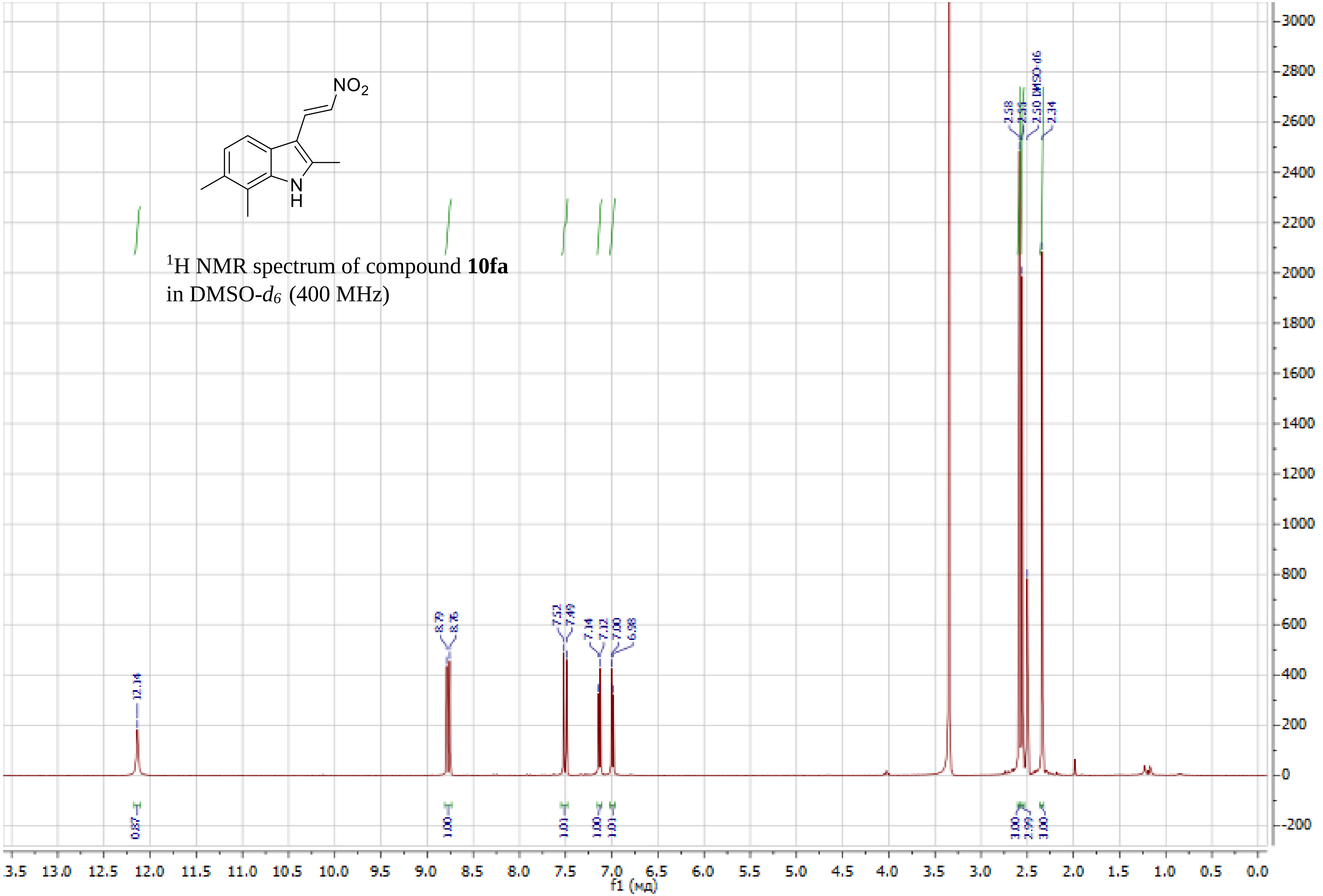

^{1}H NMR spectrum of compound **10fa** in DMSO-d_6 (400 MHz)

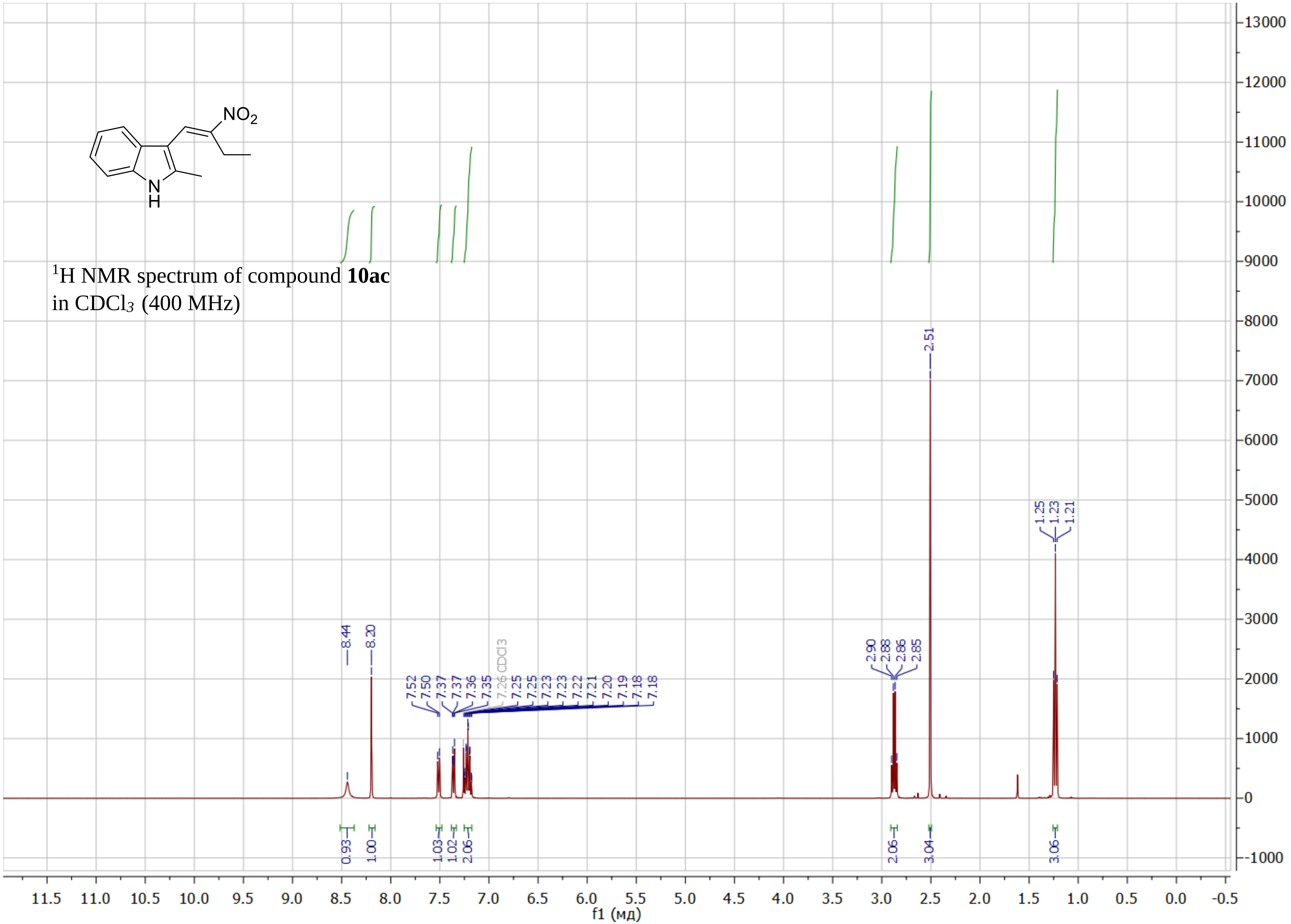

^{1}H NMR spectrum of compound **10ac** in $CDCl_3$ (400 MHz)

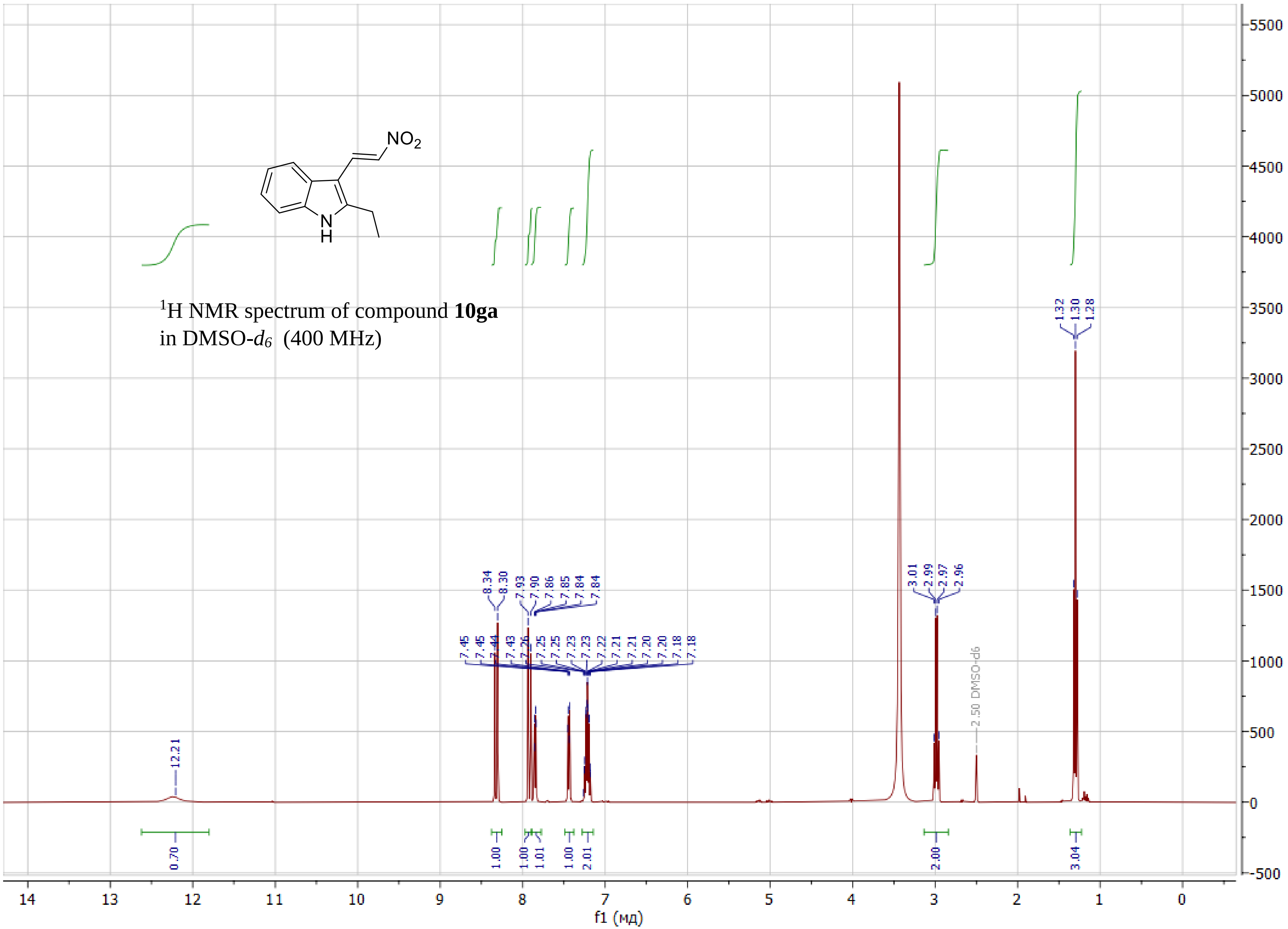
NO2
N
H
1H NMR spectrum of compound 10ga
in DMSO-d6 (400 MHz)
12.21
8.34
8.30
7.93
7.90
7.86
7.85
7.84
7.84
7.45
7.45
7.44
7.43
7.26
7.25
7.25
7.23
7.23
7.22
7.21
7.21
7.20
7.20
7.18
7.18
3.01
2.99
2.97
2.96
2.50 DMSO-d6
1.32
1.30
1.28
0.70
1.00
1.00
1.01
1.00
2.01
2.00
3.04
f1 (мд)

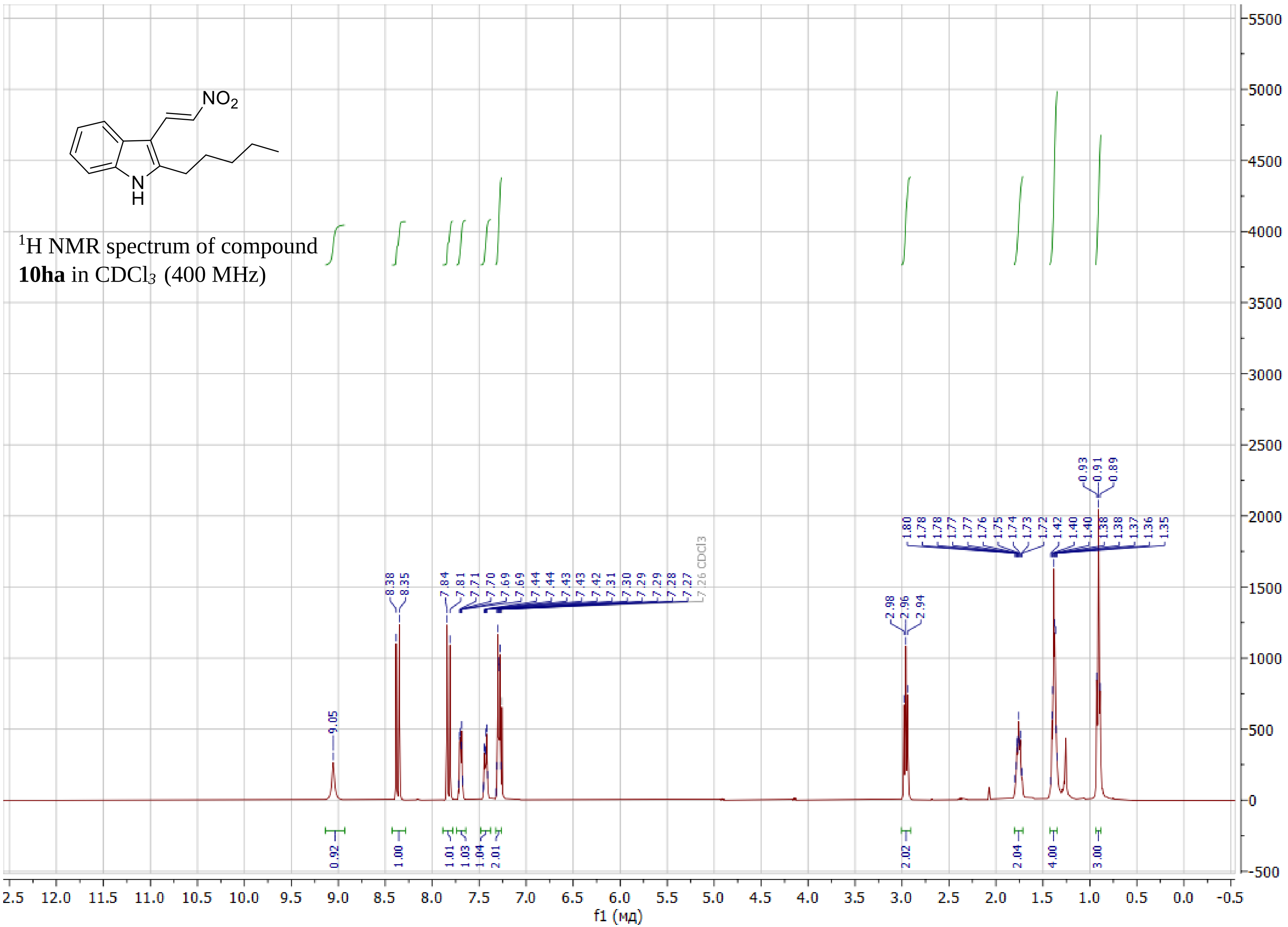

^{1}H NMR spectrum of compound **10ha** in $CDCl_3$ (400 MHz)

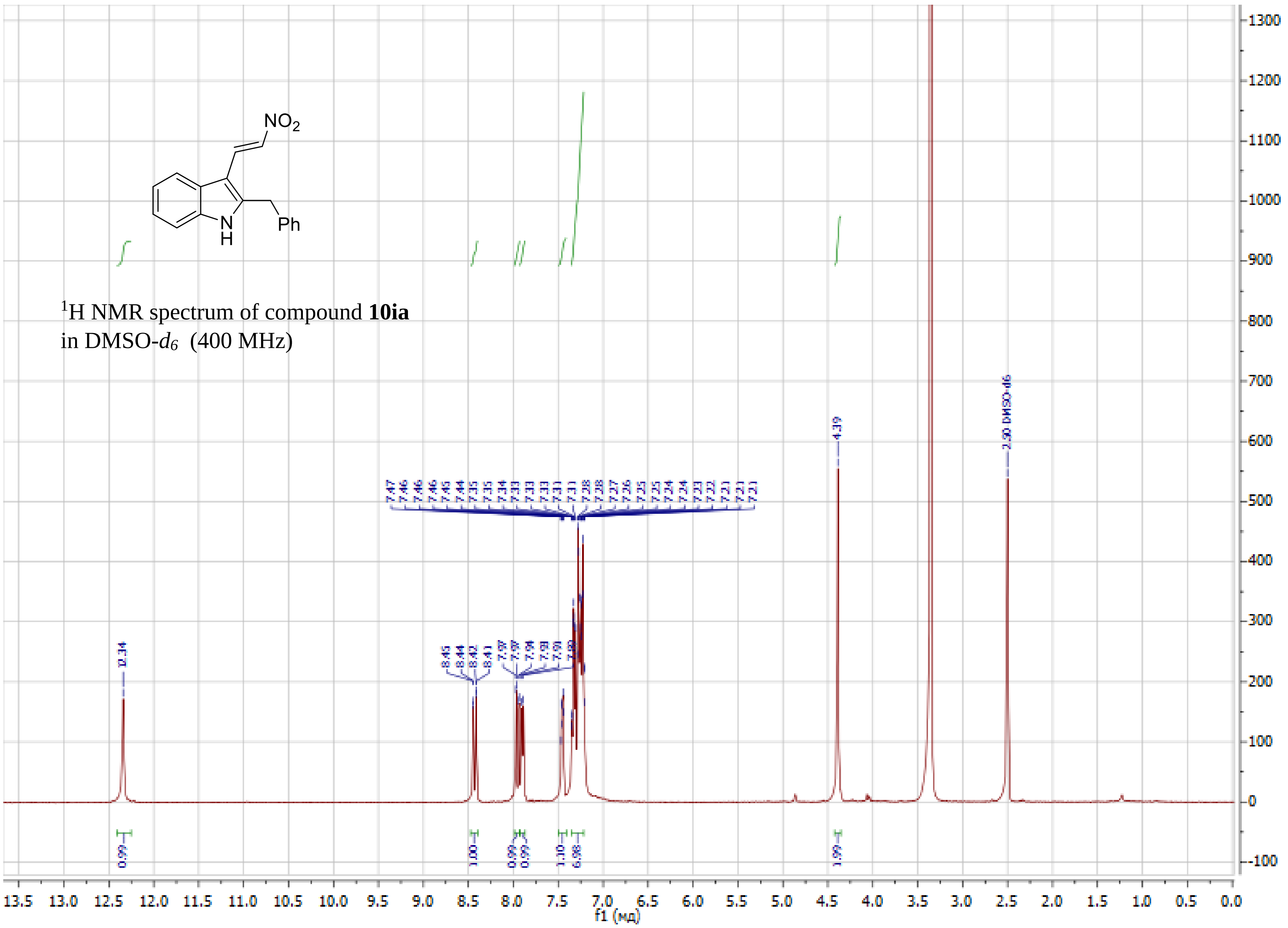

^{1}H NMR spectrum of compound **10ia** in DMSO-d_6 (400 MHz)

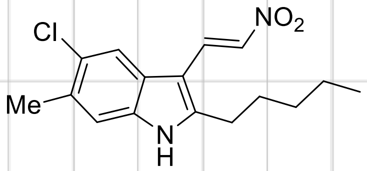

^{1}H NMR spectrum of compound **10ja** in $CDCl_3$ (400 MHz)

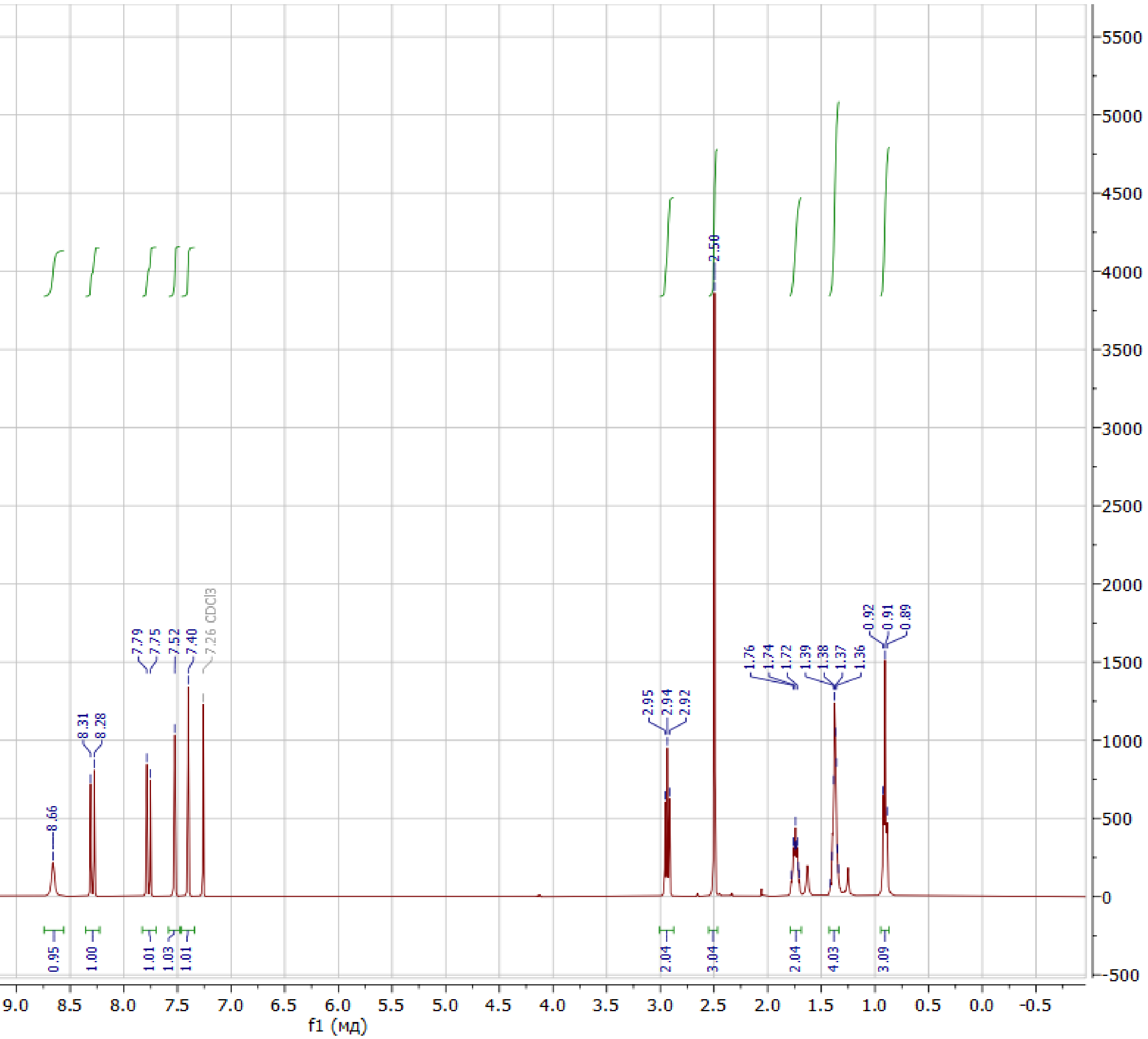

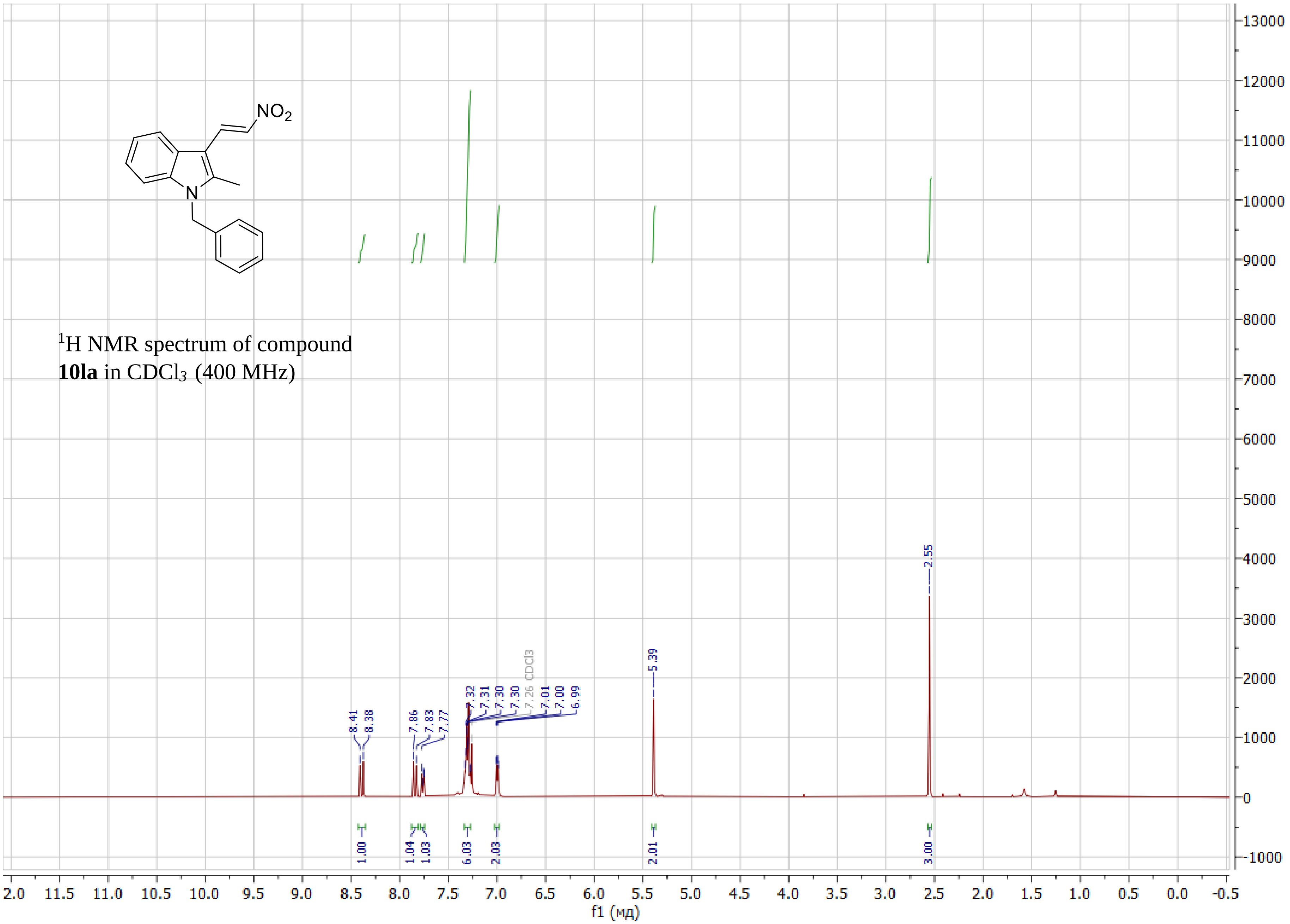

^{1}H NMR spectrum of compound **10la** in $CDCl_3$ (400 MHz)

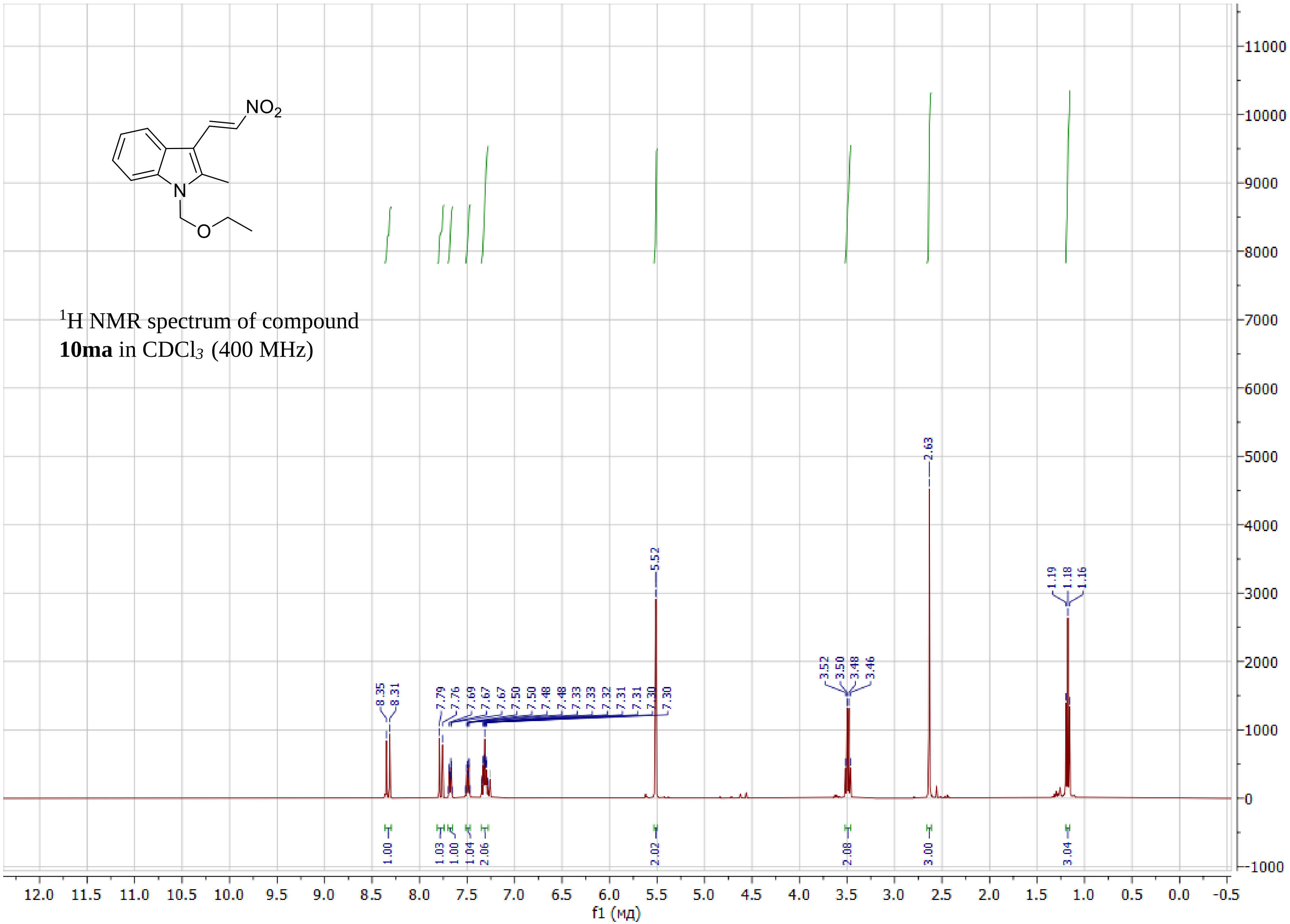

^{1}H NMR spectrum of compound **10ma** in $CDCl_3$ (400 MHz)

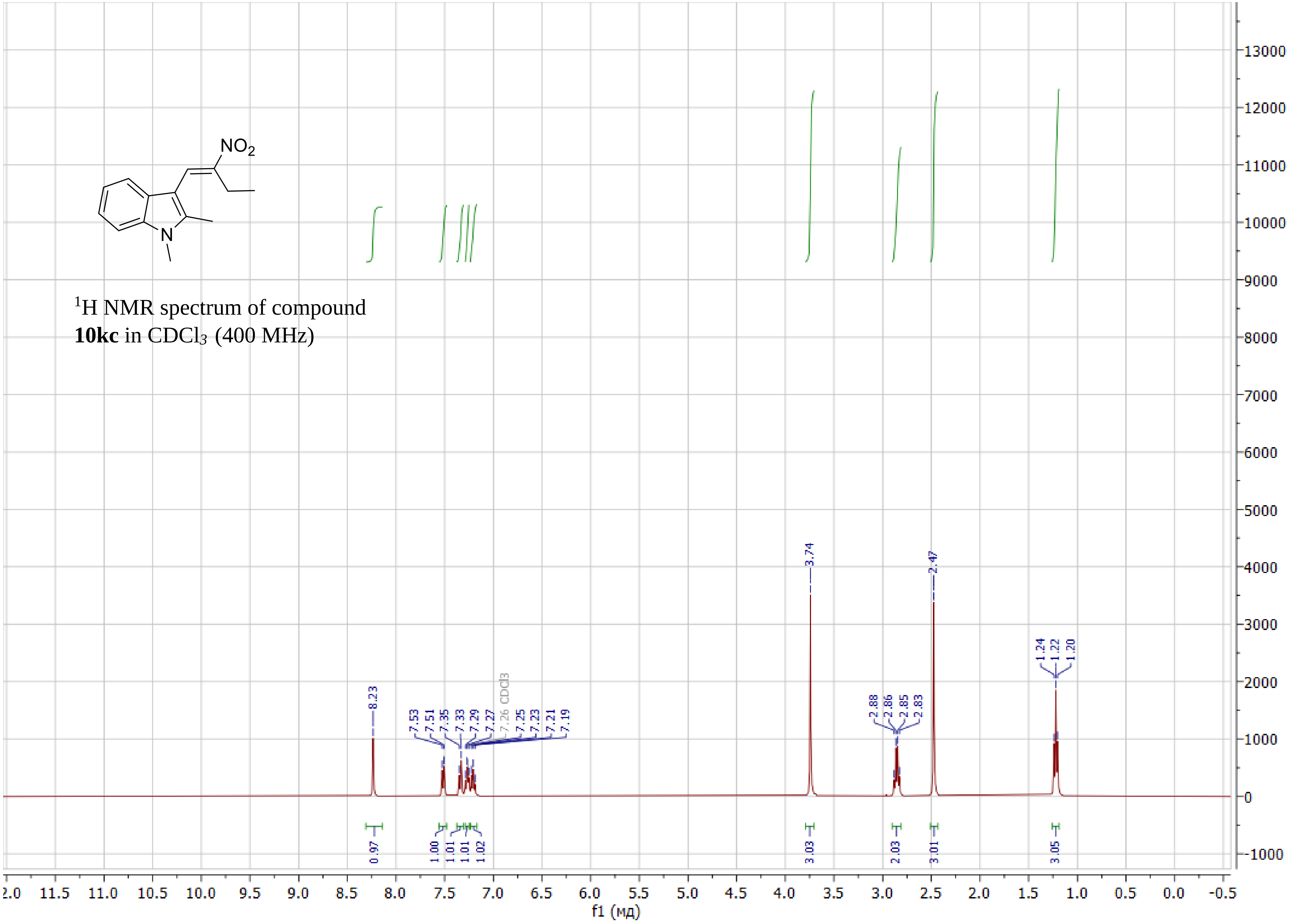

^{1}H NMR spectrum of compound **10kc** in $CDCl_3$ (400 MHz)

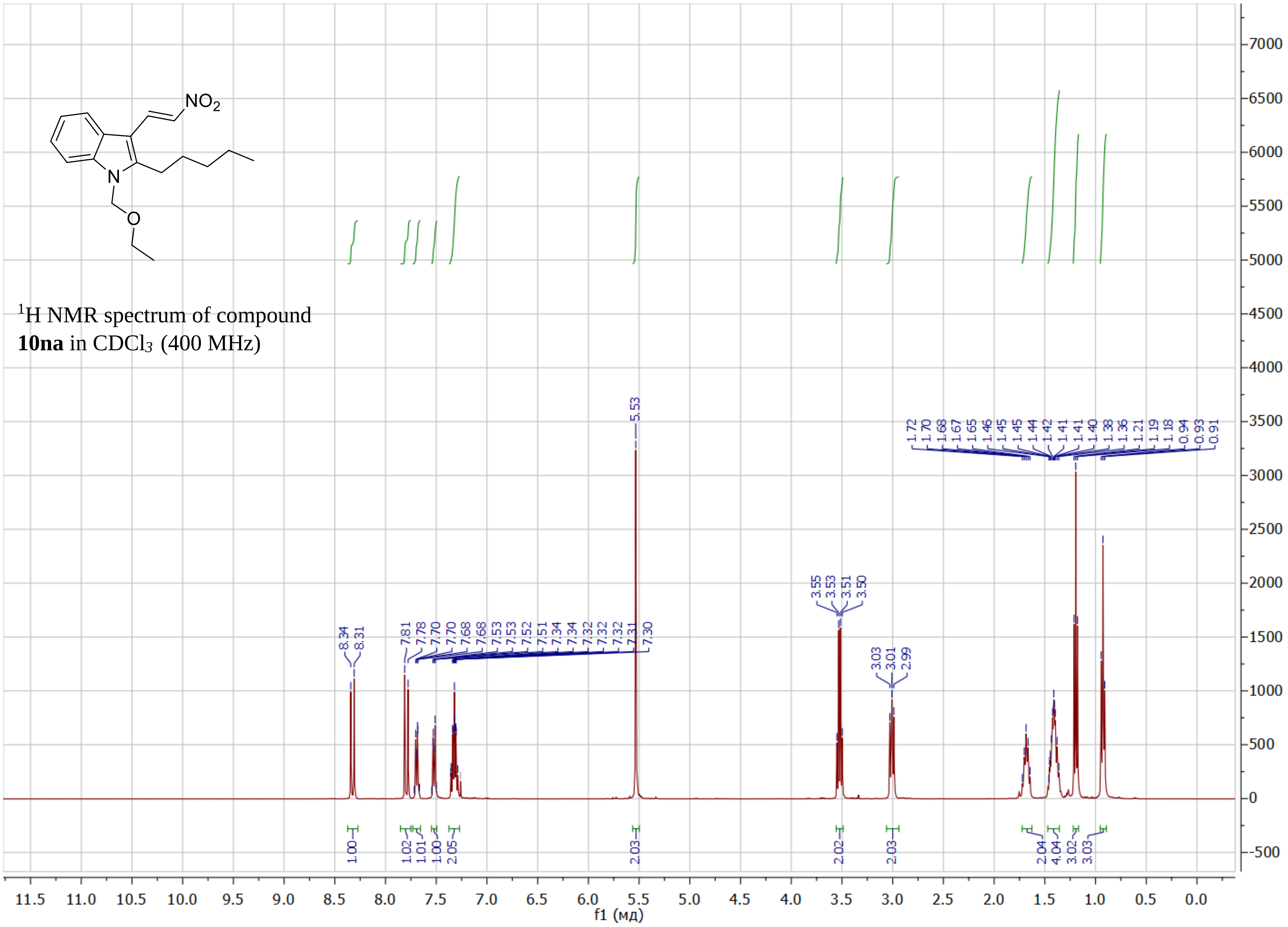

^{1}H NMR spectrum of compound **10na** in $CDCl_3$ (400 MHz)

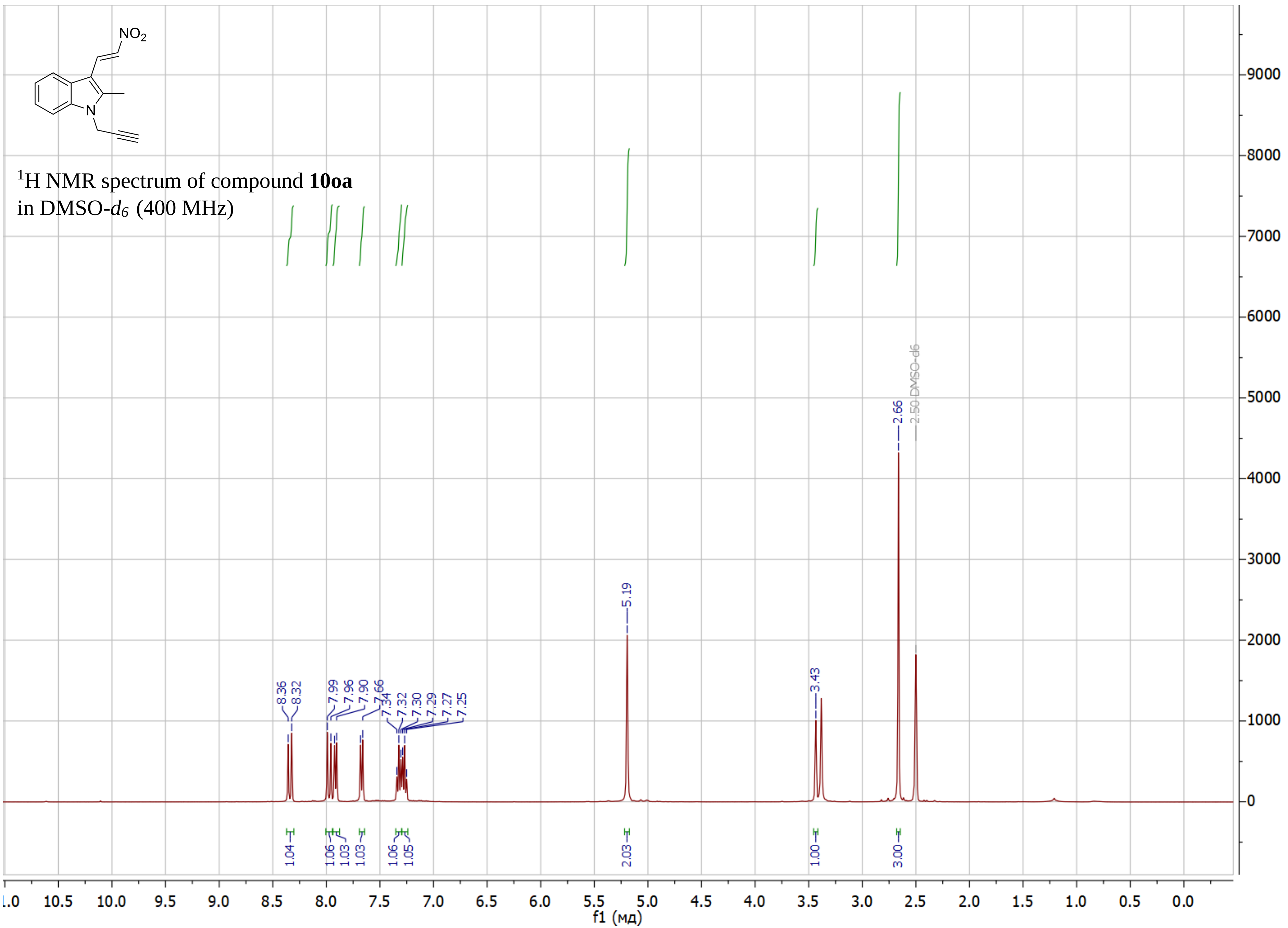

NO2
N
1H NMR spectrum of compound 10oa
in DMSO-d6 (400 MHz)
8.36
8.32
7.99
7.96
7.90
7.66
7.34
7.32
7.30
7.29
7.27
7.25
5.19
3.43
2.66
2.50 DMSO-d6
1.04
1.06
1.03
1.03
1.06
1.05
2.03
1.00
3.00
f1 (мд)

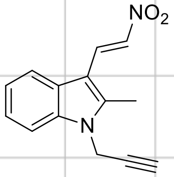

^{13}C NMR spectrum of compound **10oa** in DMSO-d_6 (101 MHz)

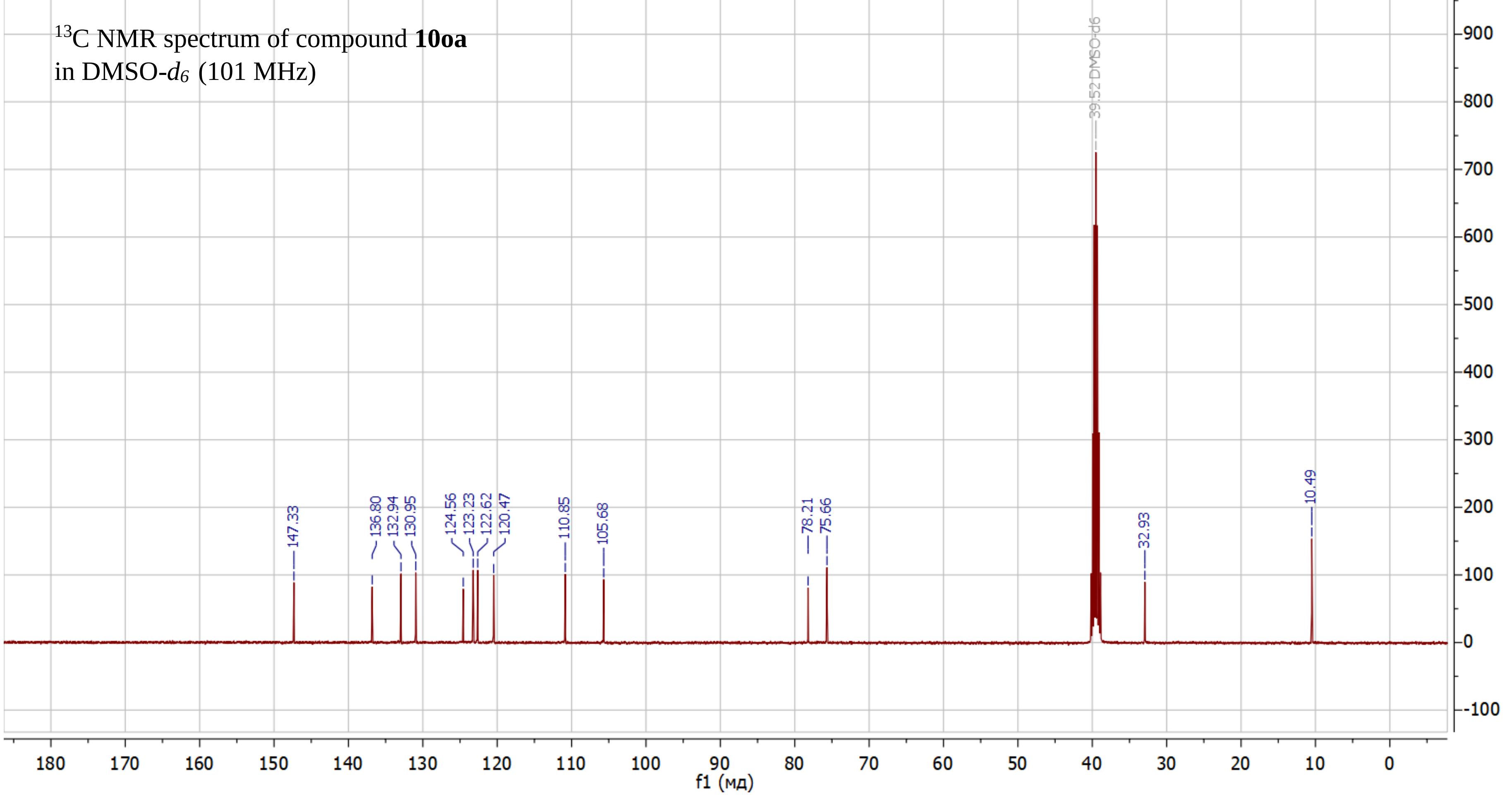

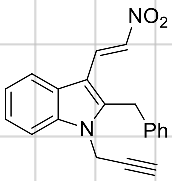

^{1}H NMR spectrum of compound **10pa** in $CDCl_3$ (400 MHz)

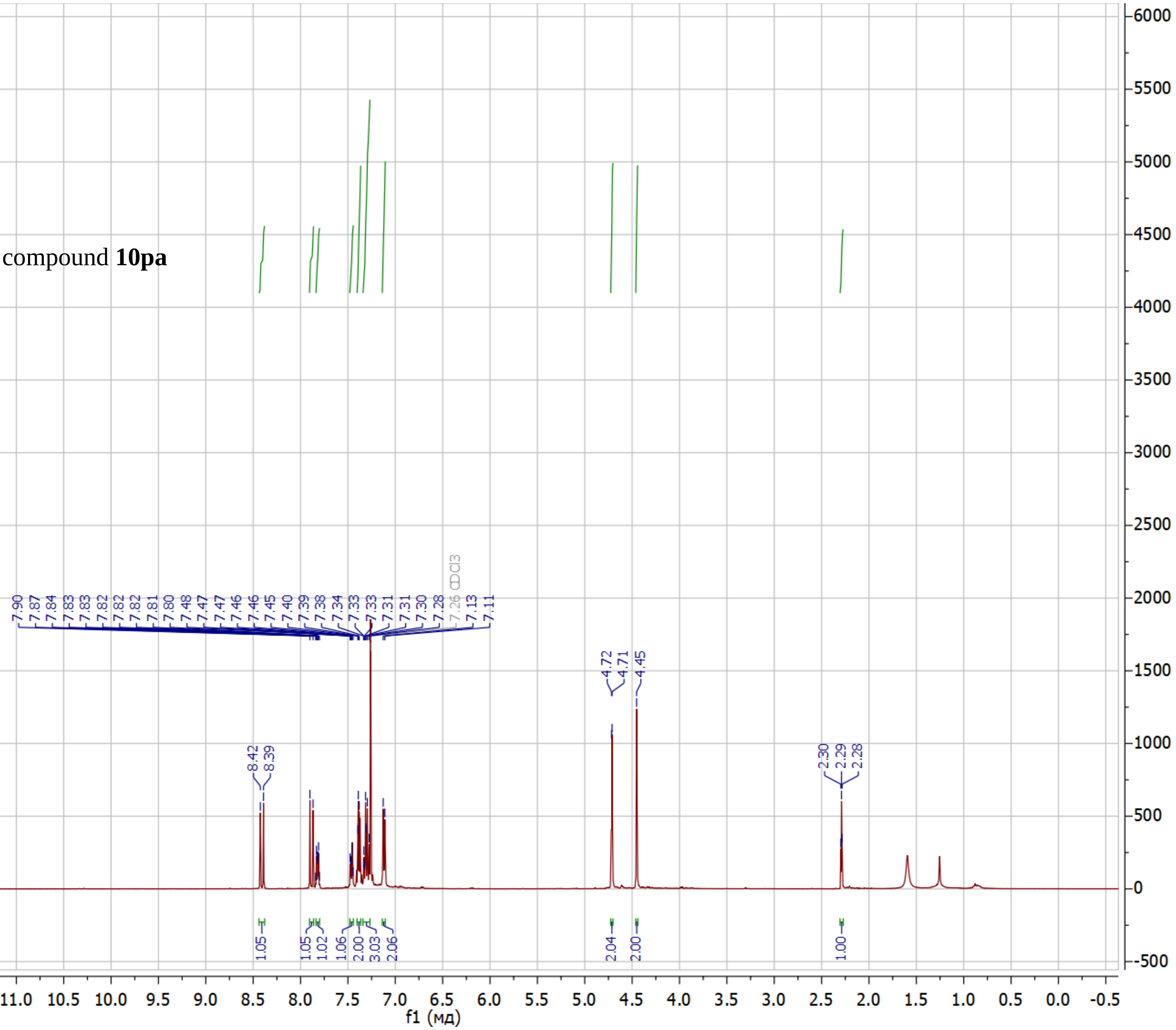

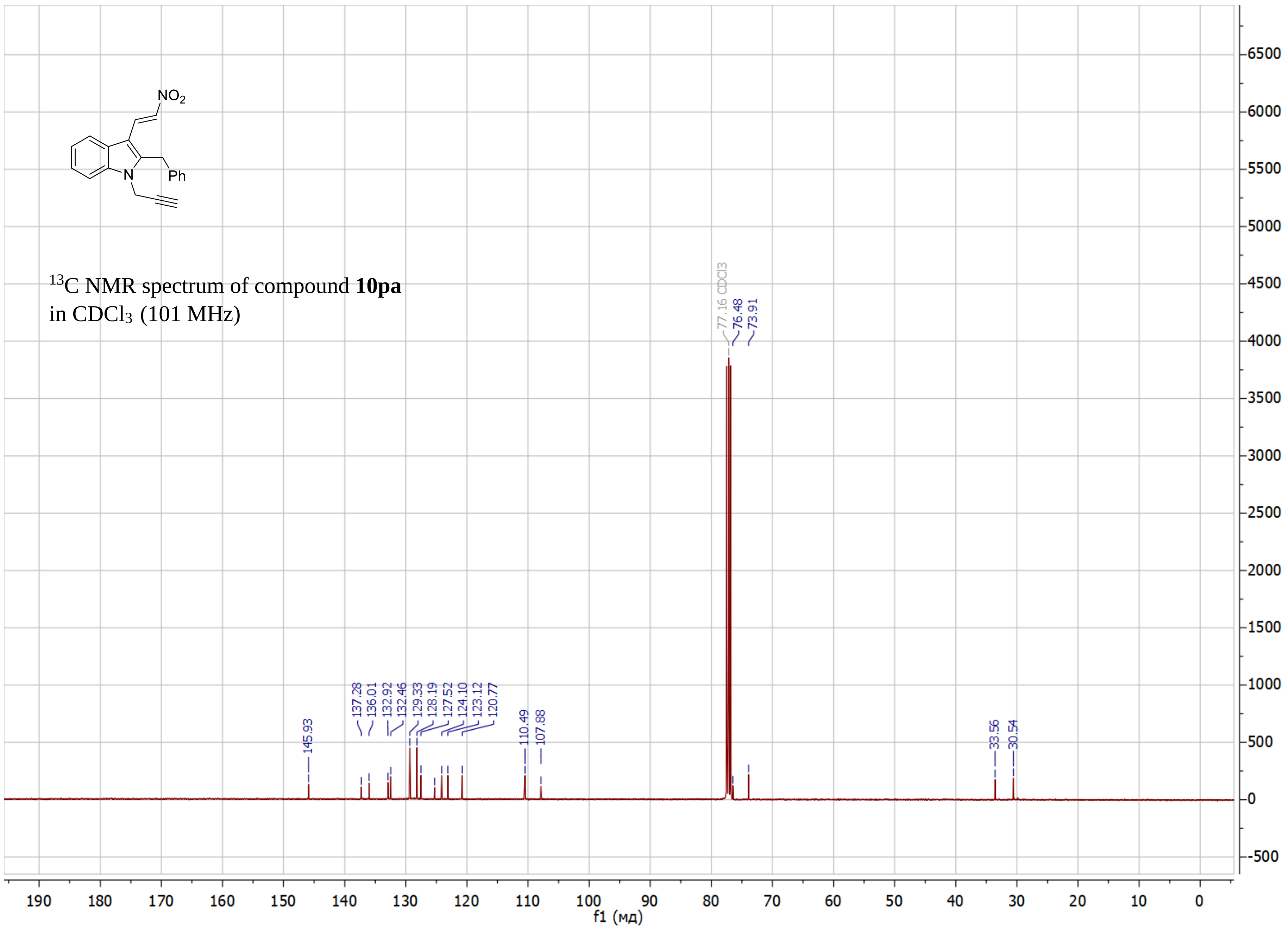
NO2
N
Ph
13C NMR spectrum of compound 10pa
in CDCl3 (101 MHz)
145.93
137.28
136.01
132.92
132.46
129.33
128.19
127.52
124.10
123.12
120.77
110.49
107.88
77.16 CDCl3
76.48
73.91
33.56
30.54
6500
6000
5500
5000
4500
4000
3500
3000
2500
2000
1500
1000
500
0
-500
190
180
170
160
150
140
130
120
110
100
90
80
70
60
50
40
30
20
10
0
f1 (мд)

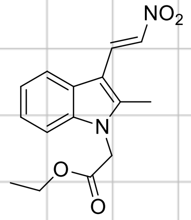

^{1}H NMR spectrum of compound **10qa** in DMSO-d_6 (400 MHz)

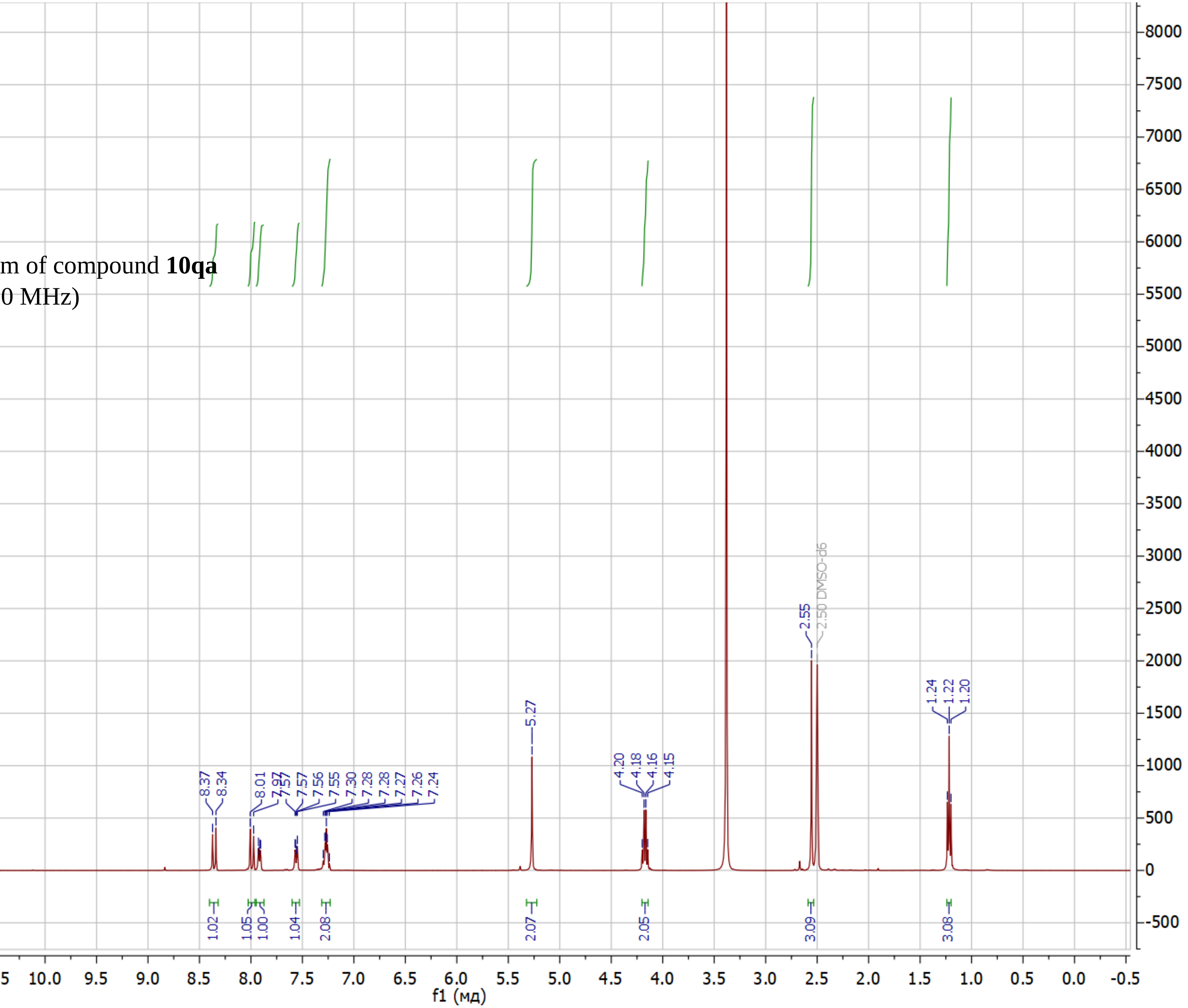

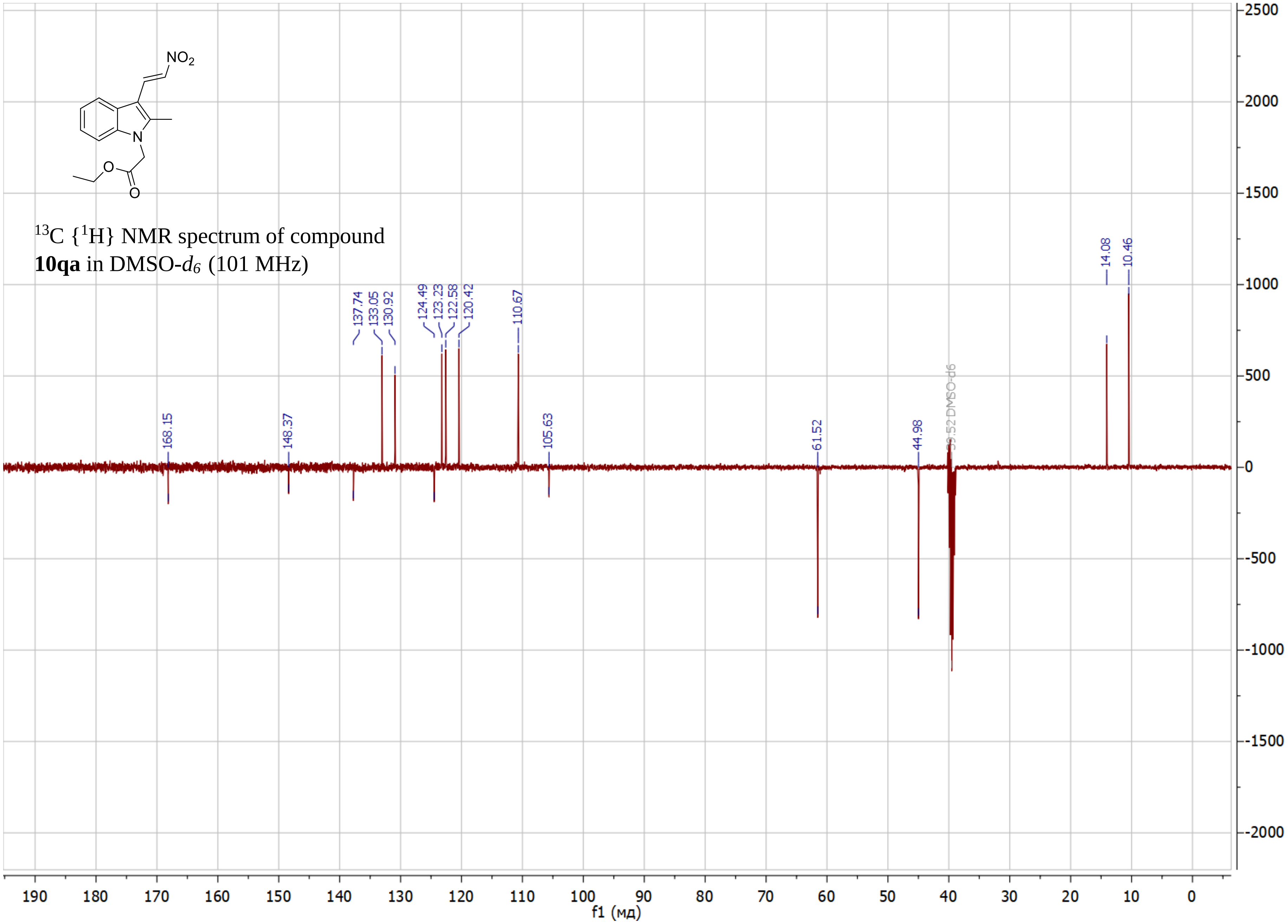

^{13}C {^{1}H} NMR spectrum of compound **10qa** in DMSO-d_6 (101 MHz)

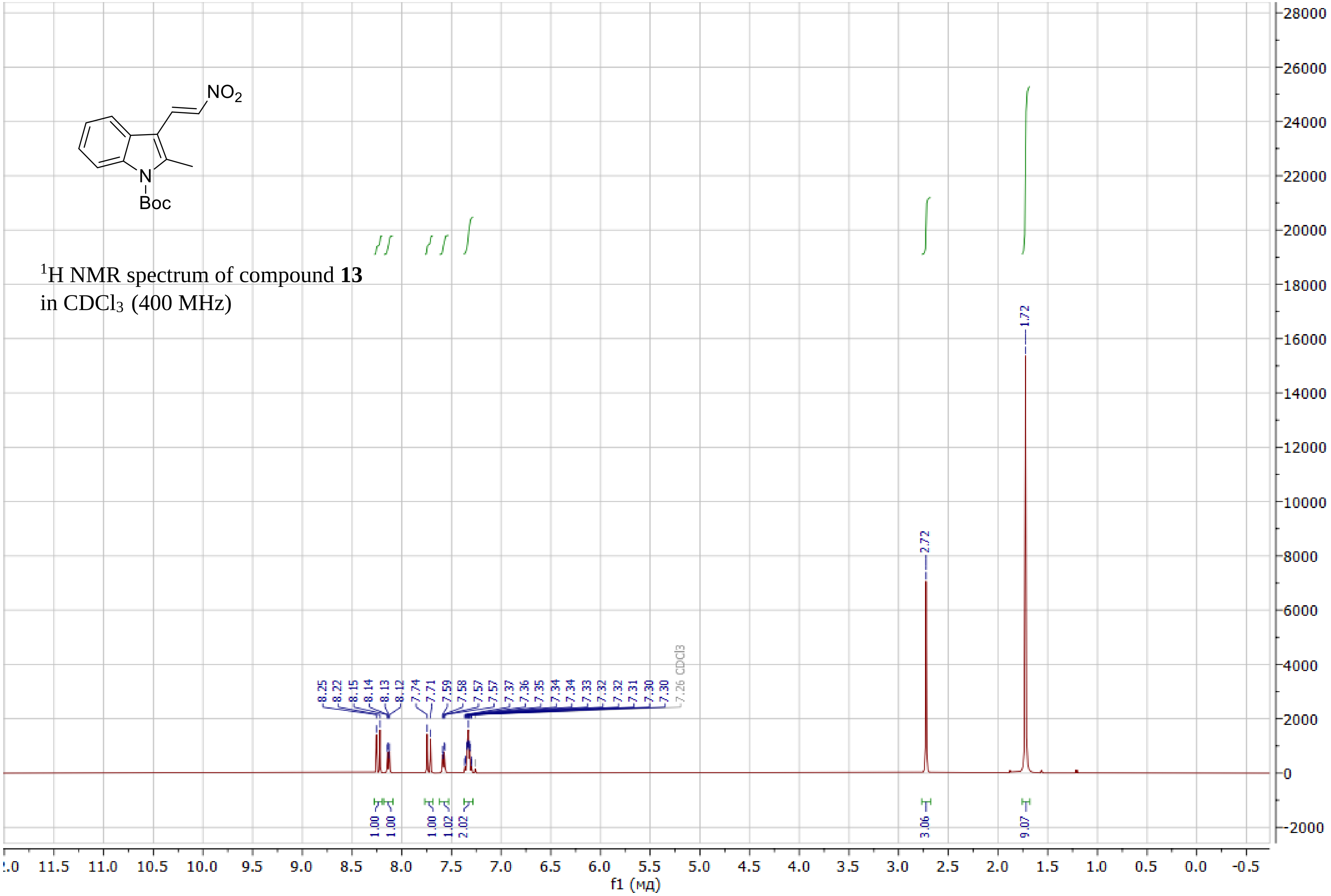

^{1}H NMR spectrum of compound **13** in $CDCl_3$ (400 MHz)

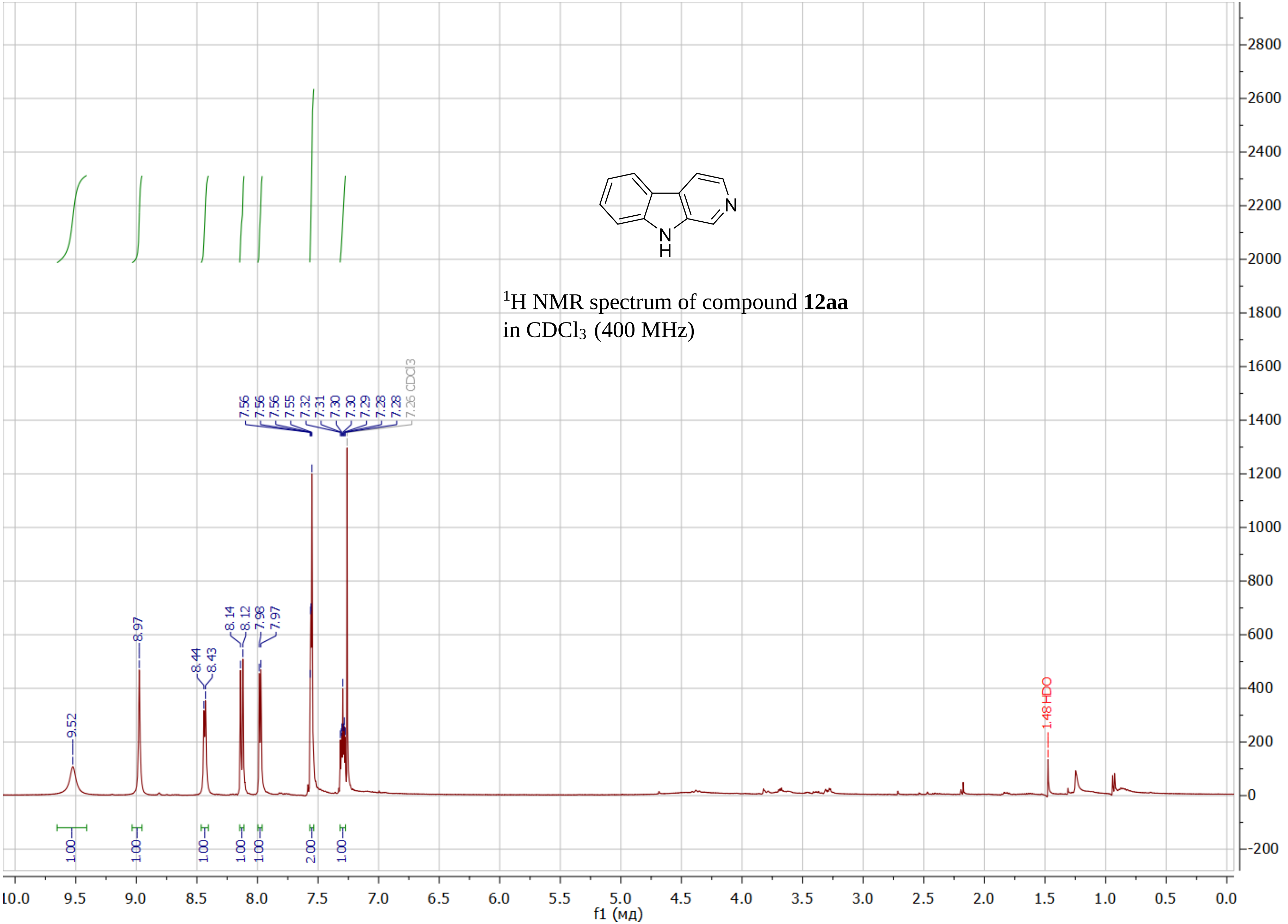

^{1}H NMR spectrum of compound **12aa** in $CDCl_3$ (400 MHz)

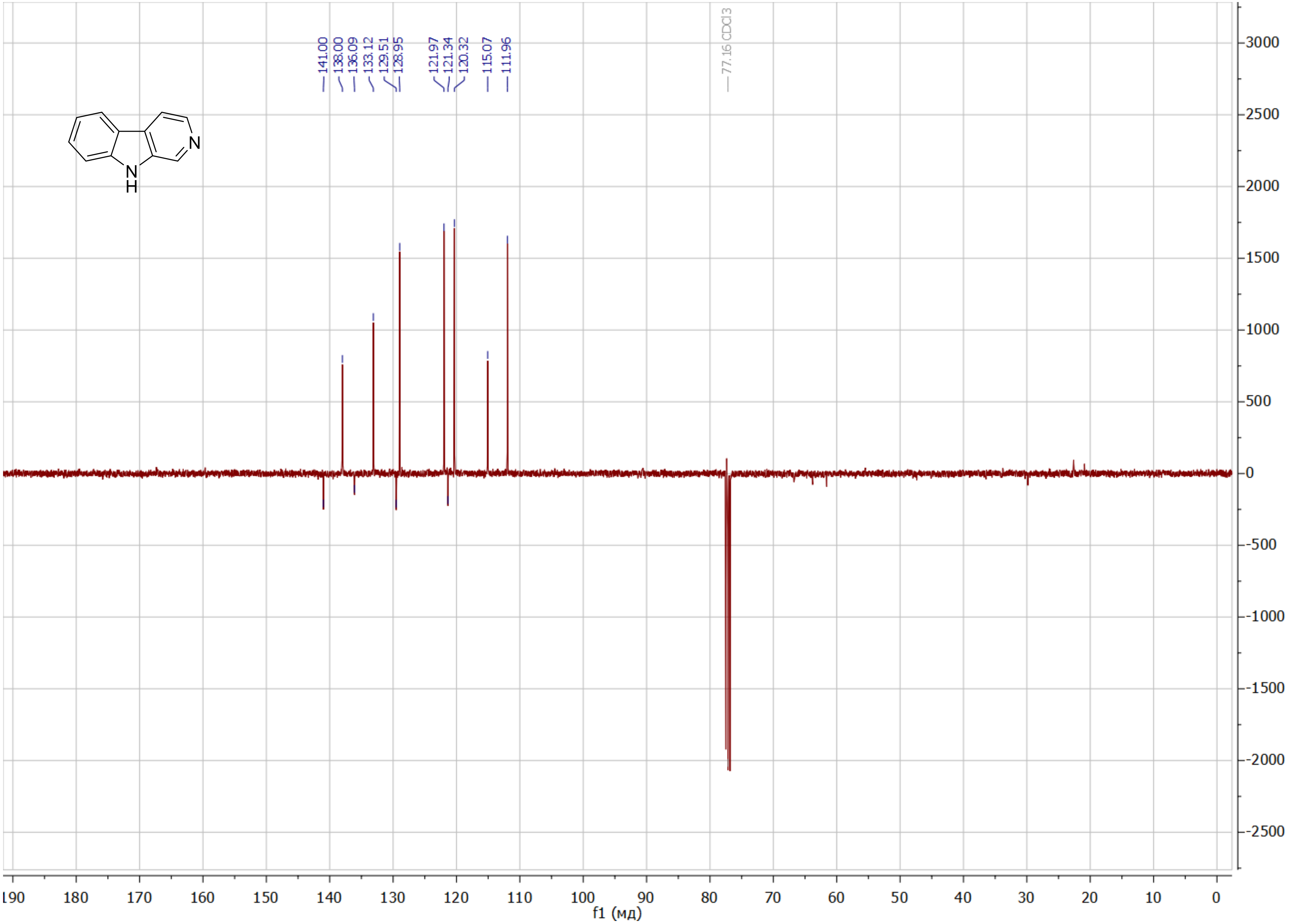
141.00
138.00
136.09
133.12
129.51
128.95
121.97
121.34
120.32
115.07
111.96
77.16 CDCl3
N
N
H
f1 (мд)

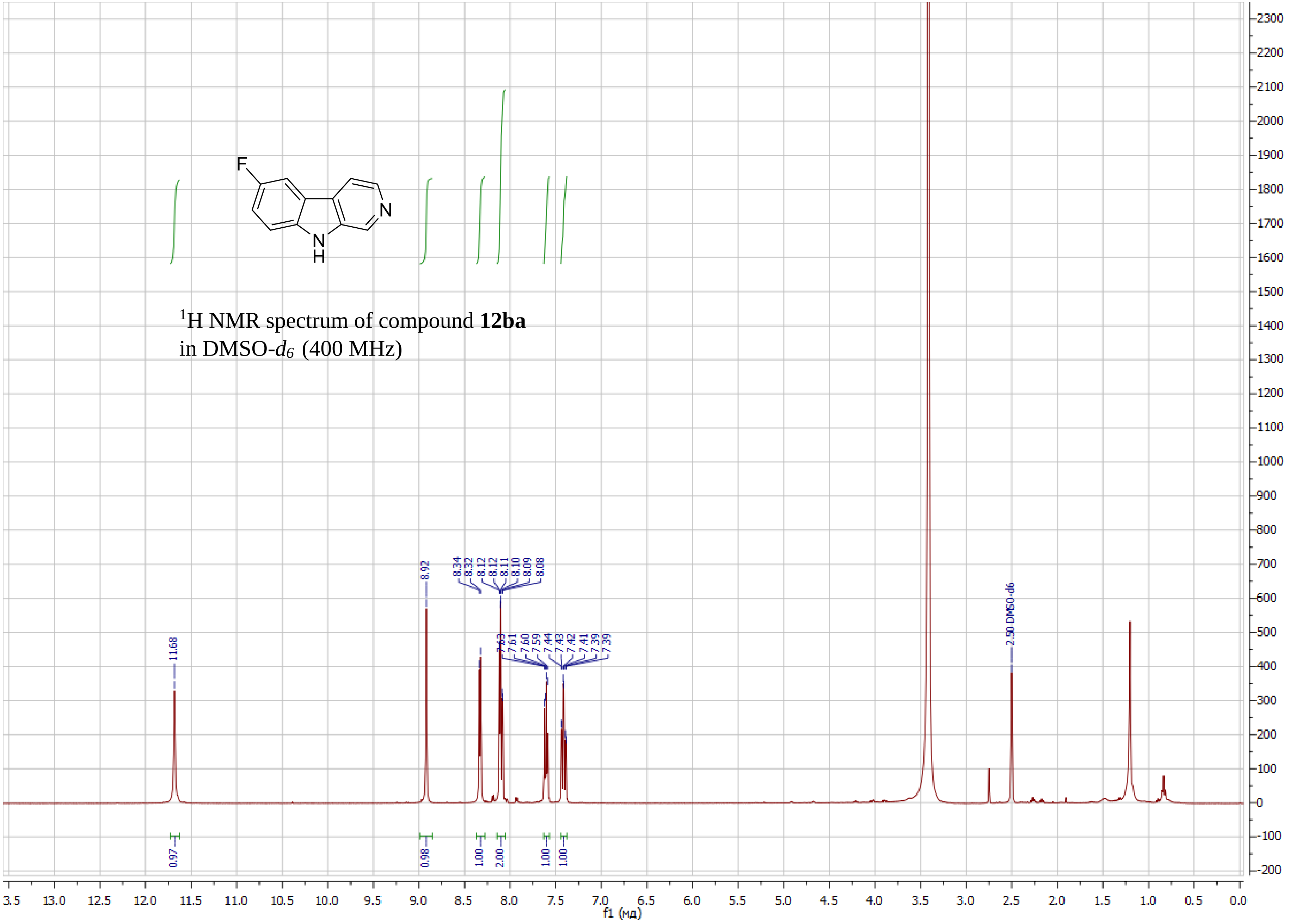

^{1}H NMR spectrum of compound **12ba** in DMSO-d_6 (400 MHz)

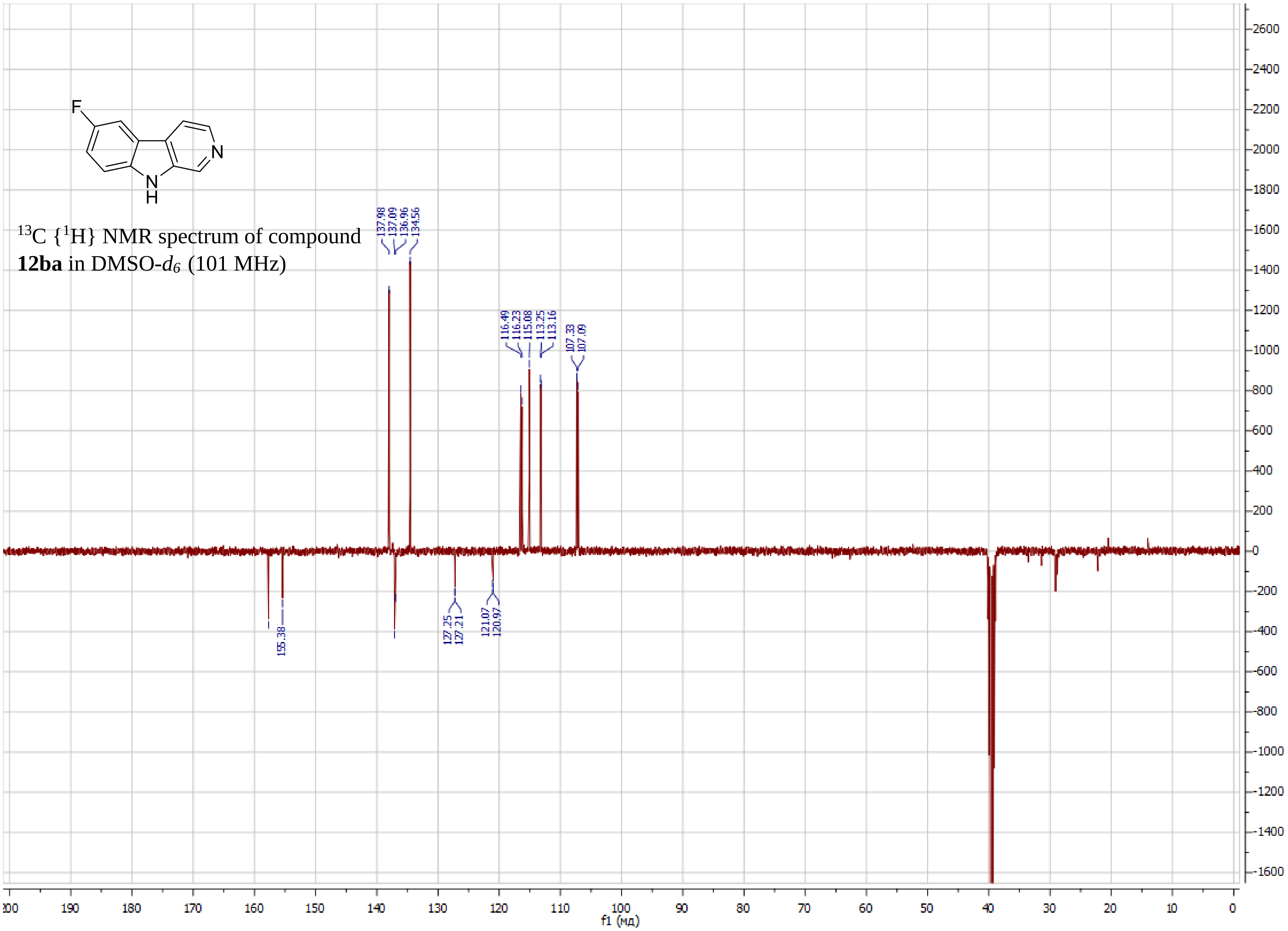

^{13}C {^{1}H} NMR spectrum of compound **12ba** in DMSO-d_6 (101 MHz)

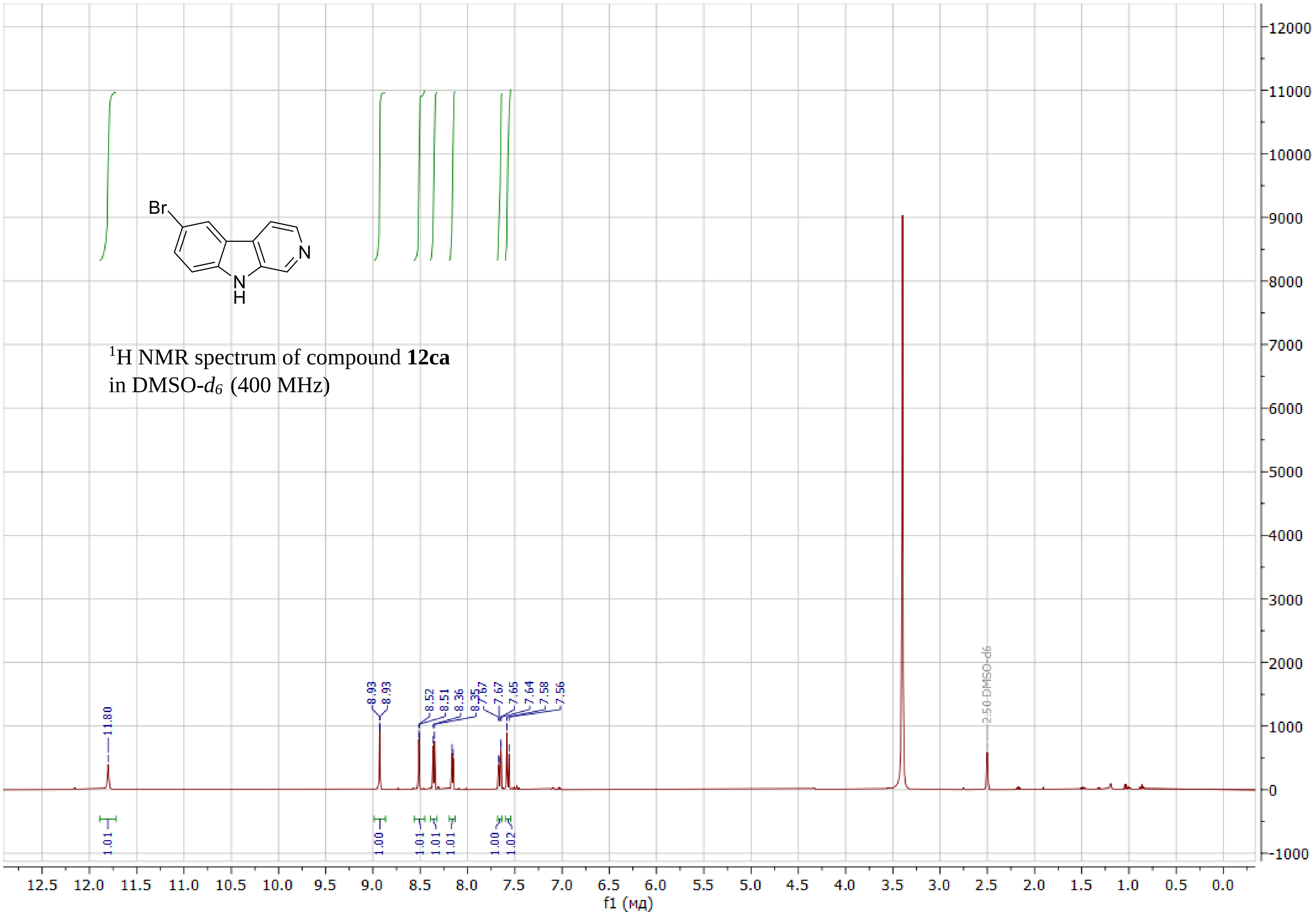

^{1}H NMR spectrum of compound **12ca** in DMSO-d_6 (400 MHz)

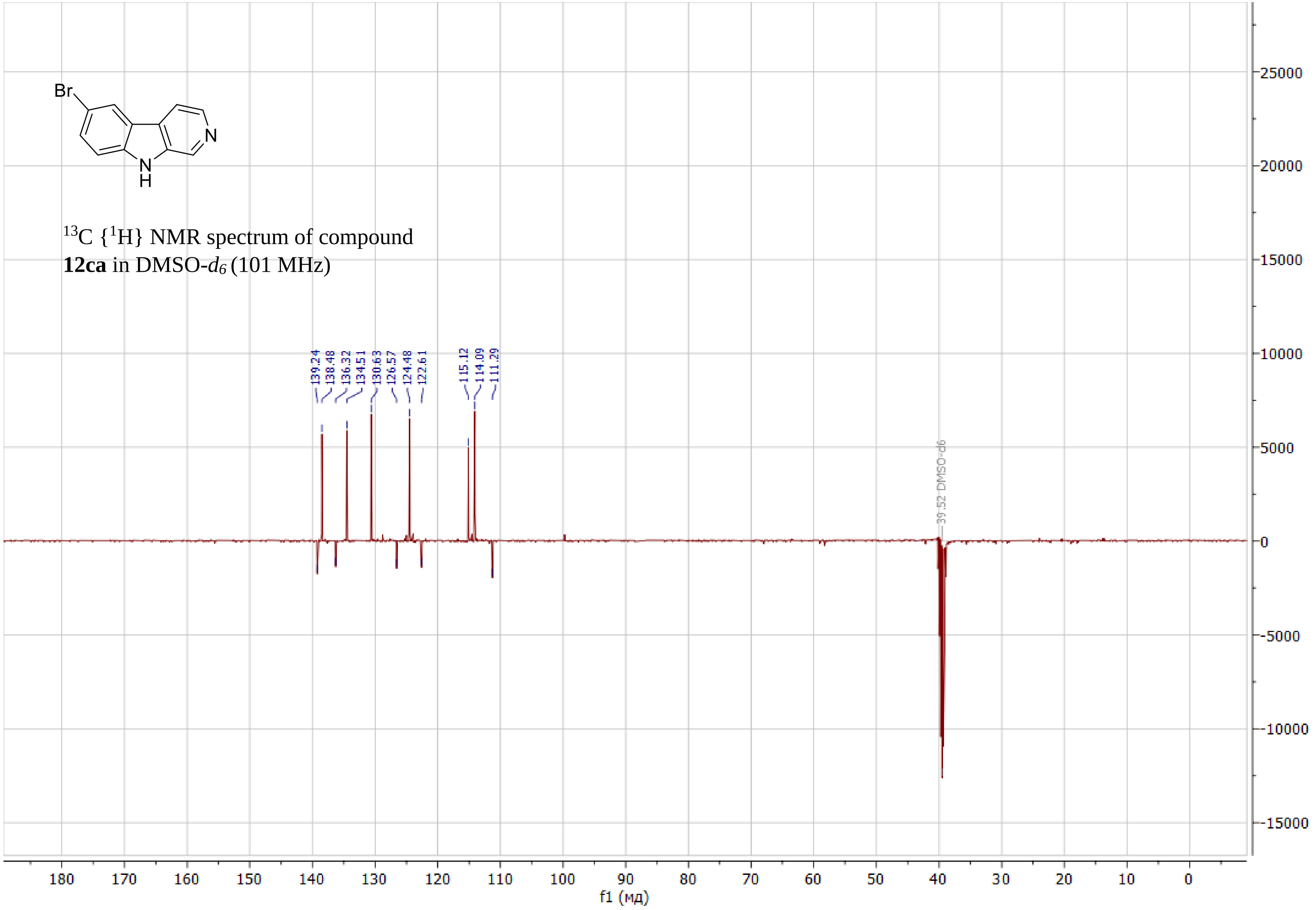

$^{13}C\ \{^{1}H\}$ NMR spectrum of compound **12ca** in DMSO-d_6 (101 MHz)

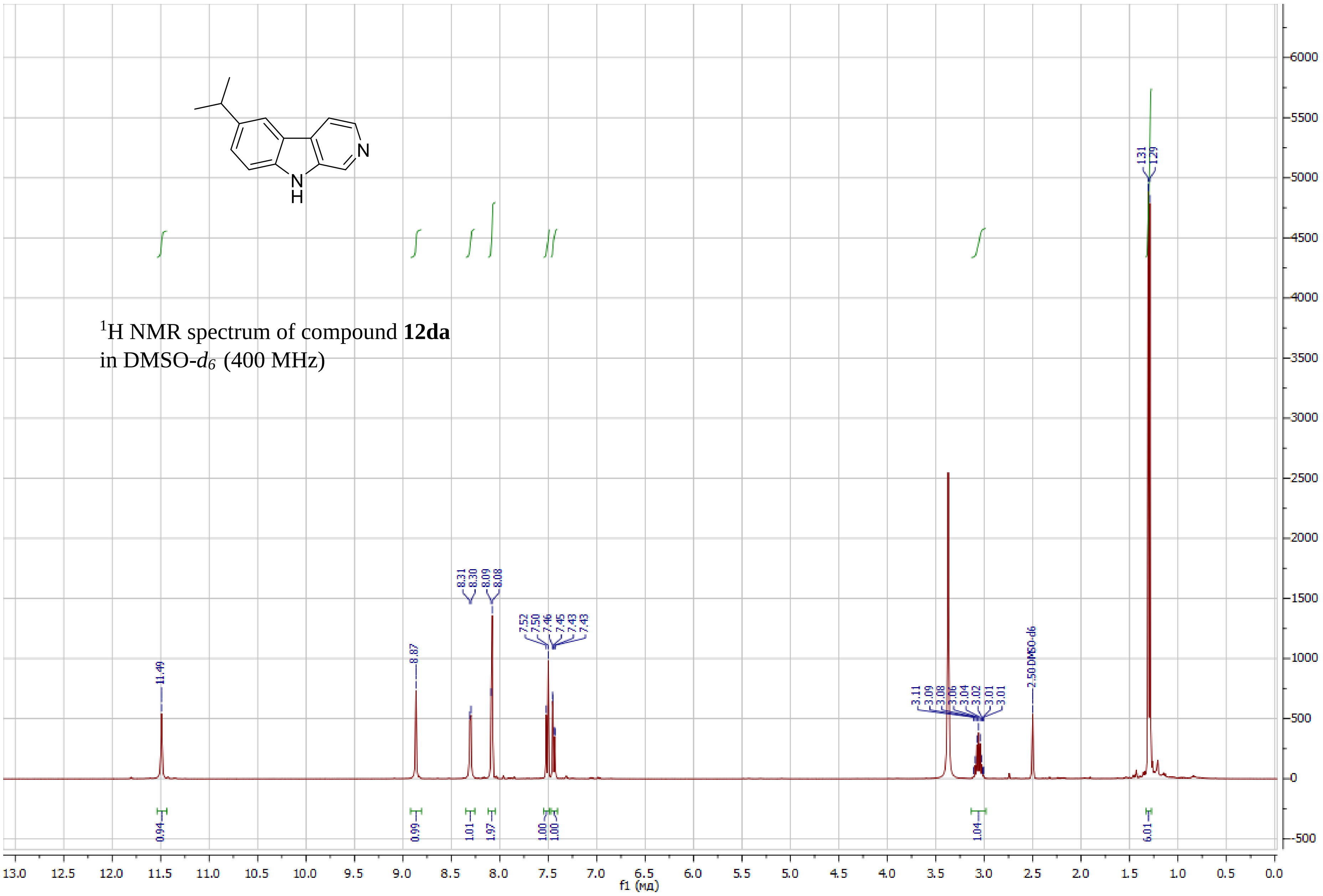

^{1}H NMR spectrum of compound **12da** in DMSO-d_6 (400 MHz)

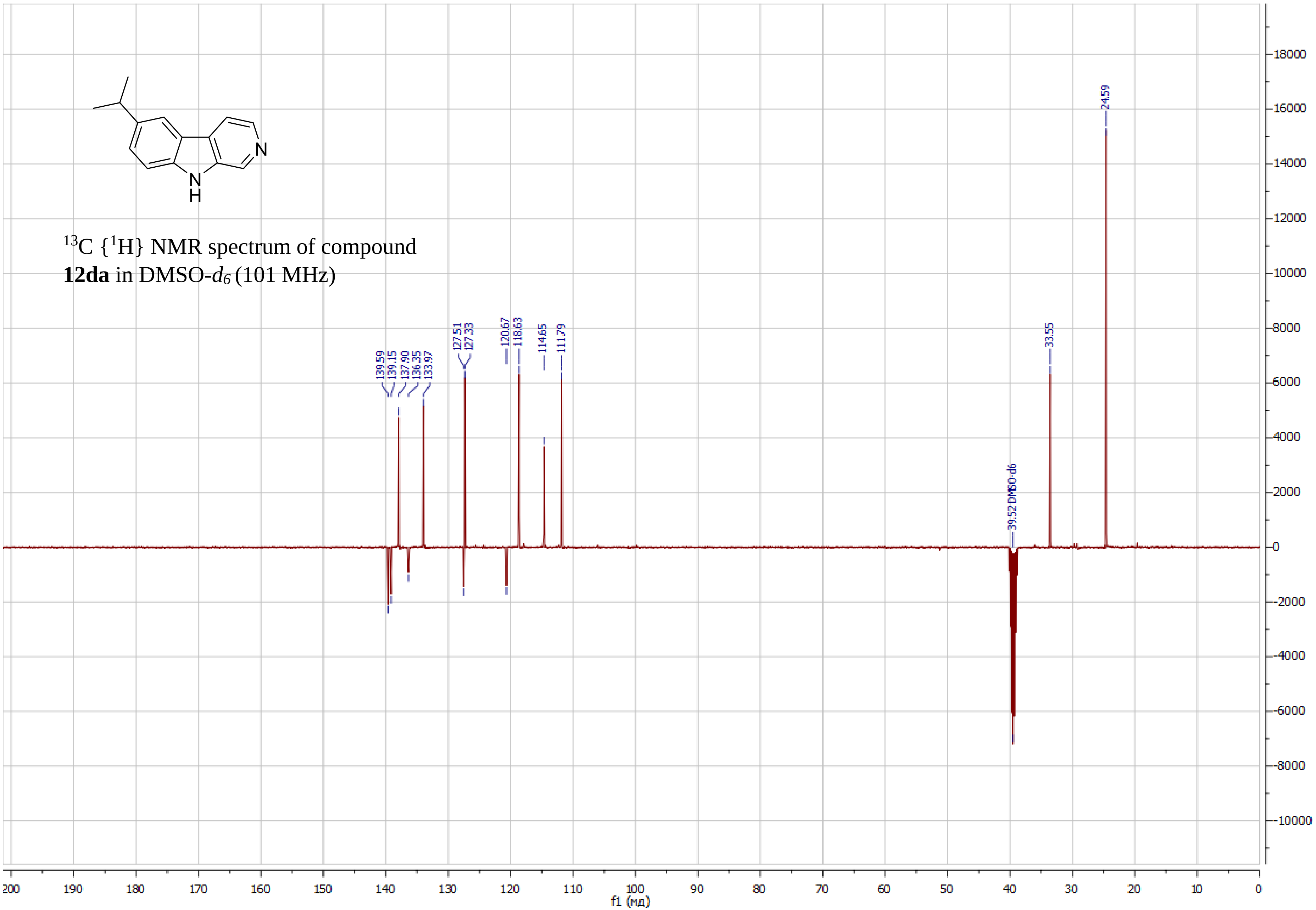

^{13}C {^{1}H} NMR spectrum of compound **12da** in DMSO-d_6 (101 MHz)

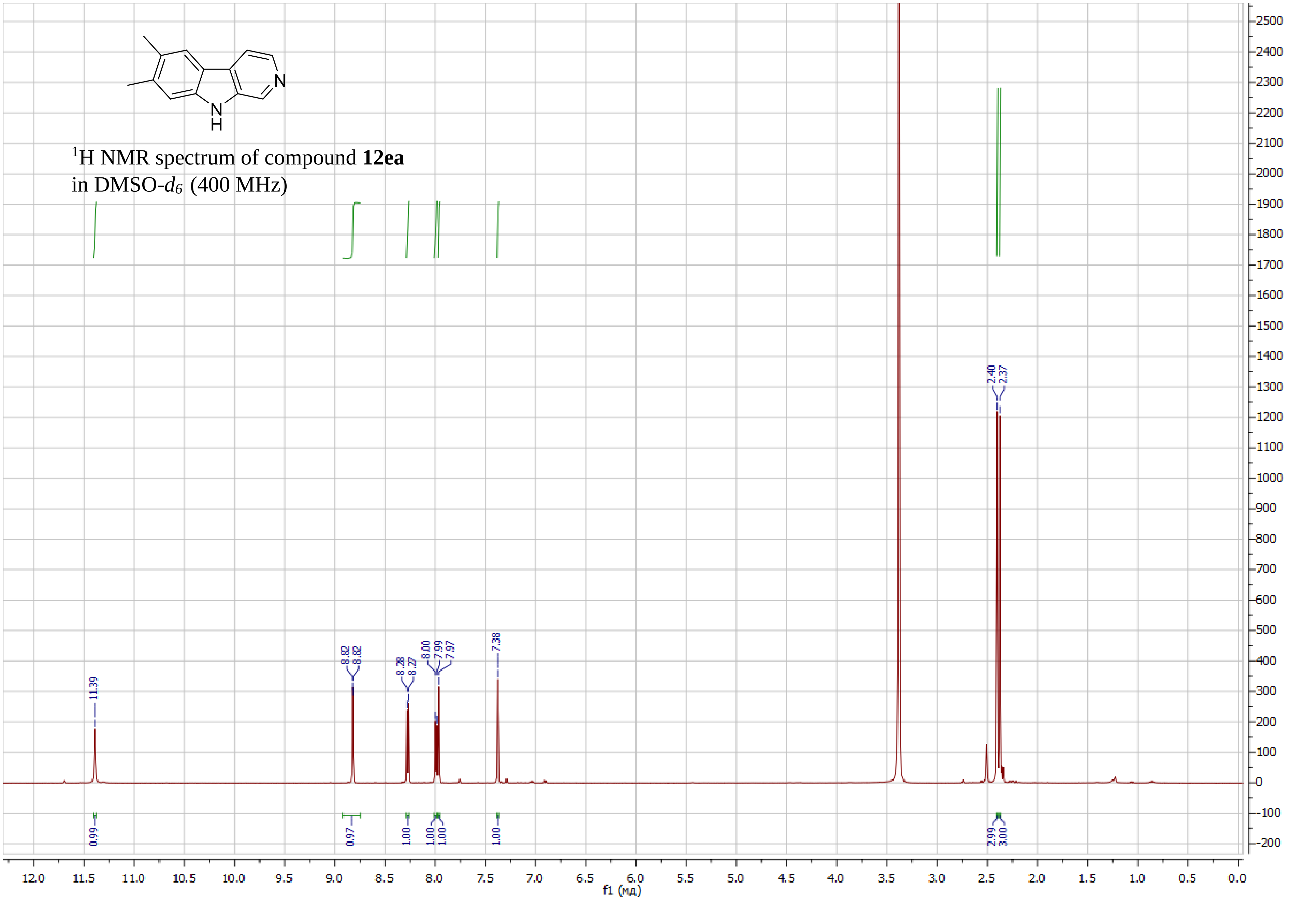

^{1}H NMR spectrum of compound **12ea** in DMSO-d_6 (400 MHz)

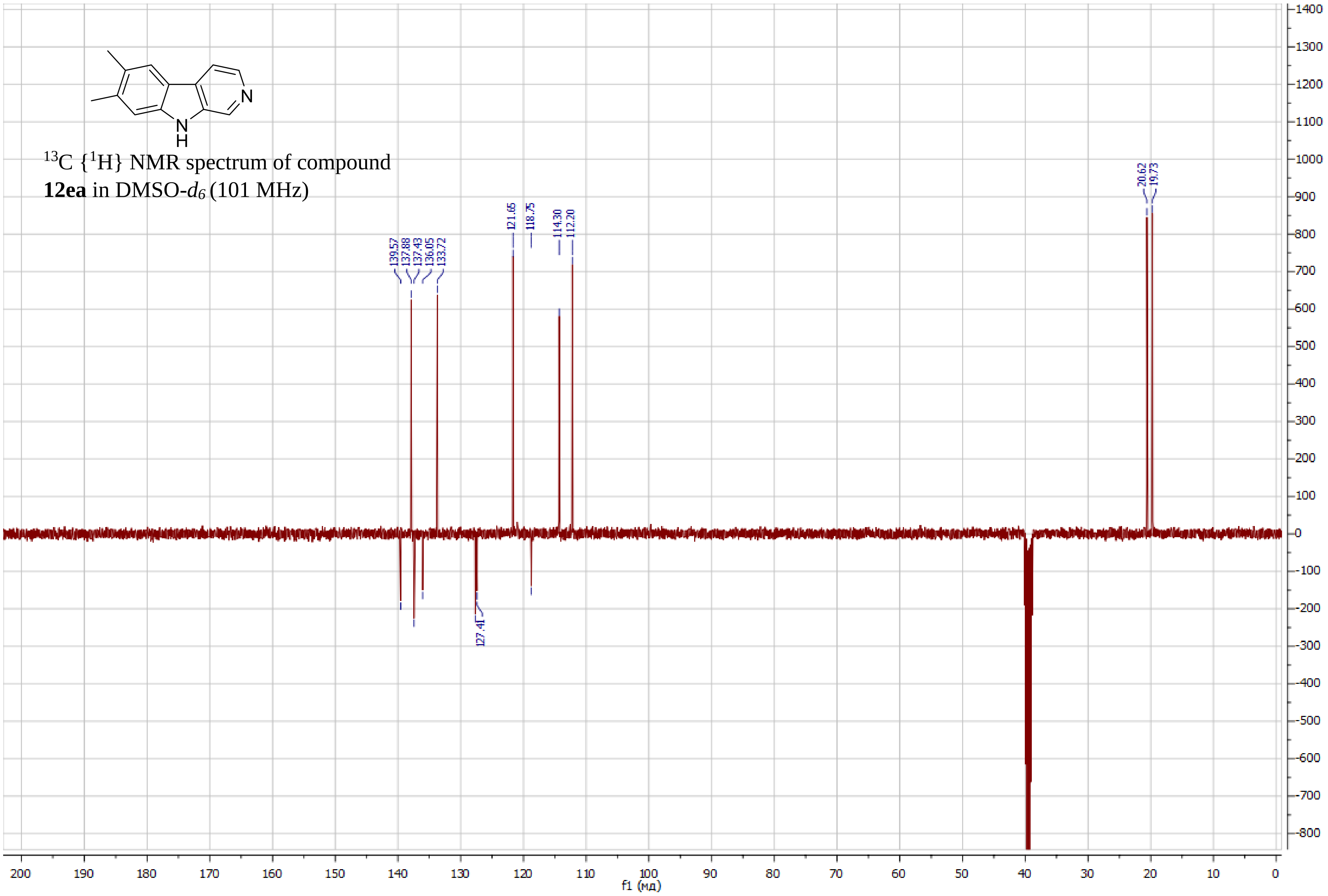

^{13}C {^{1}H} NMR spectrum of compound **12ea** in DMSO-d_6 (101 MHz)

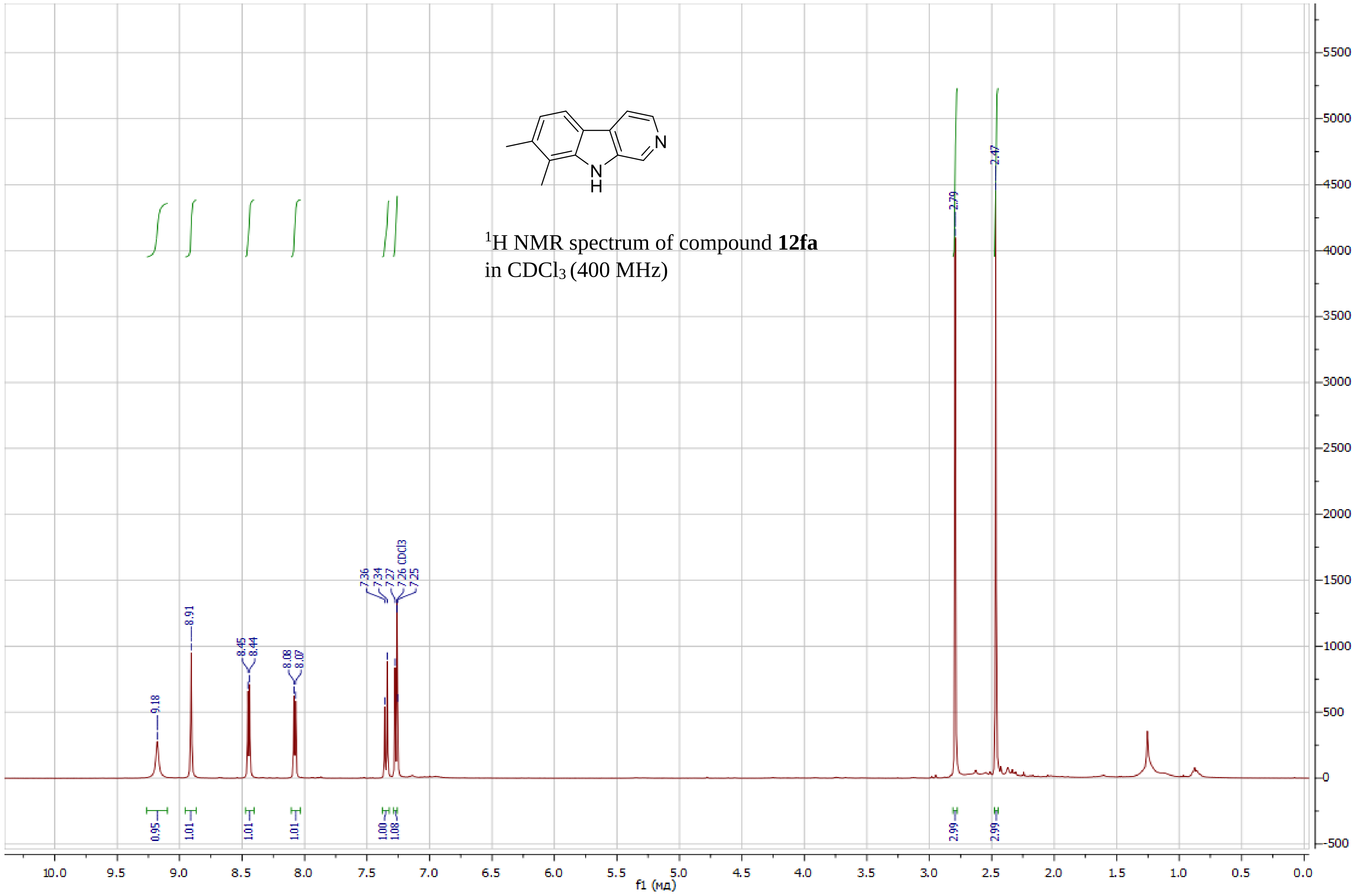

^{1}H NMR spectrum of compound **12fa** in $CDCl_3$ (400 MHz)

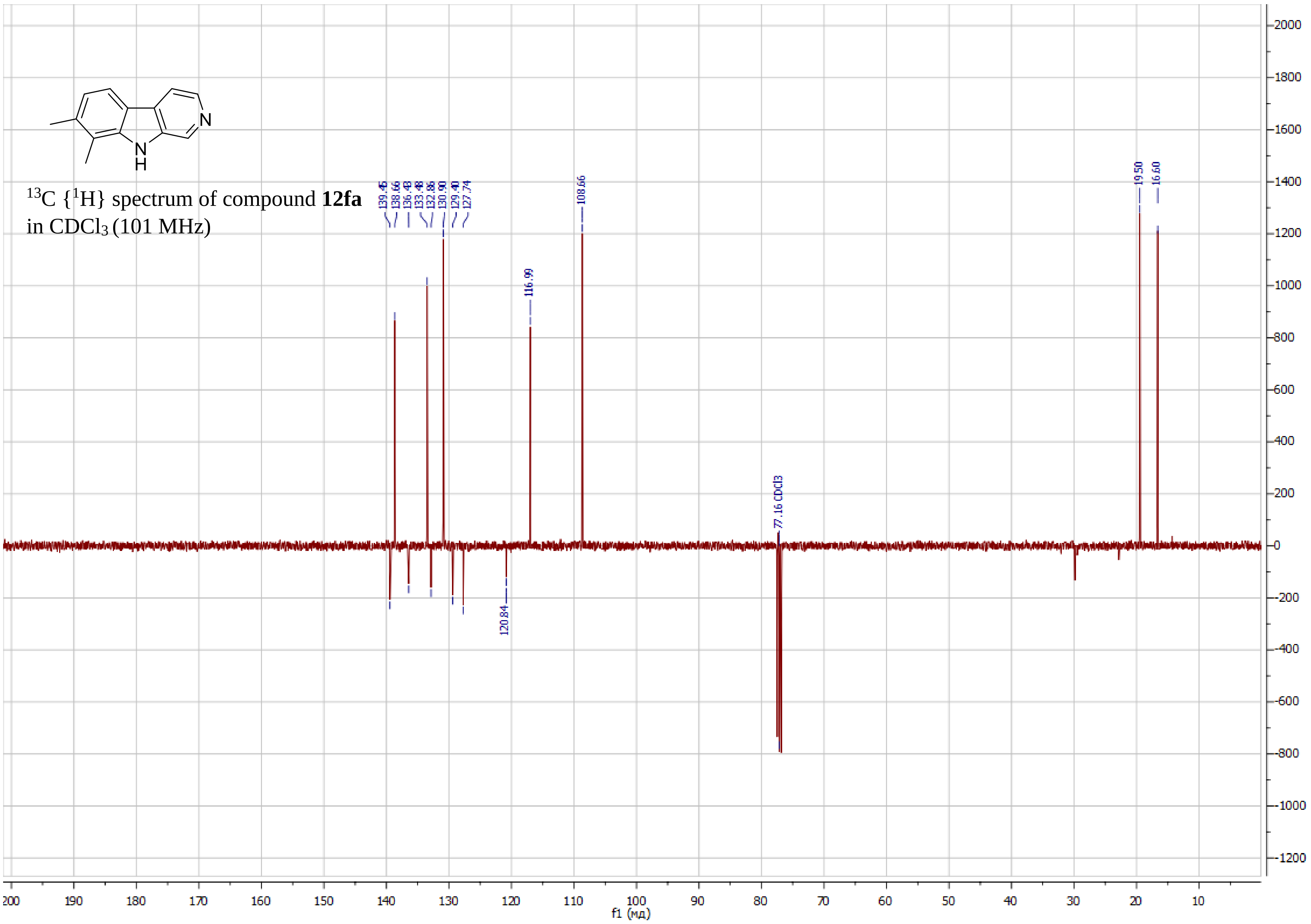

^{13}C {^{1}H} spectrum of compound **12fa** in $CDCl_3$ (101 MHz)

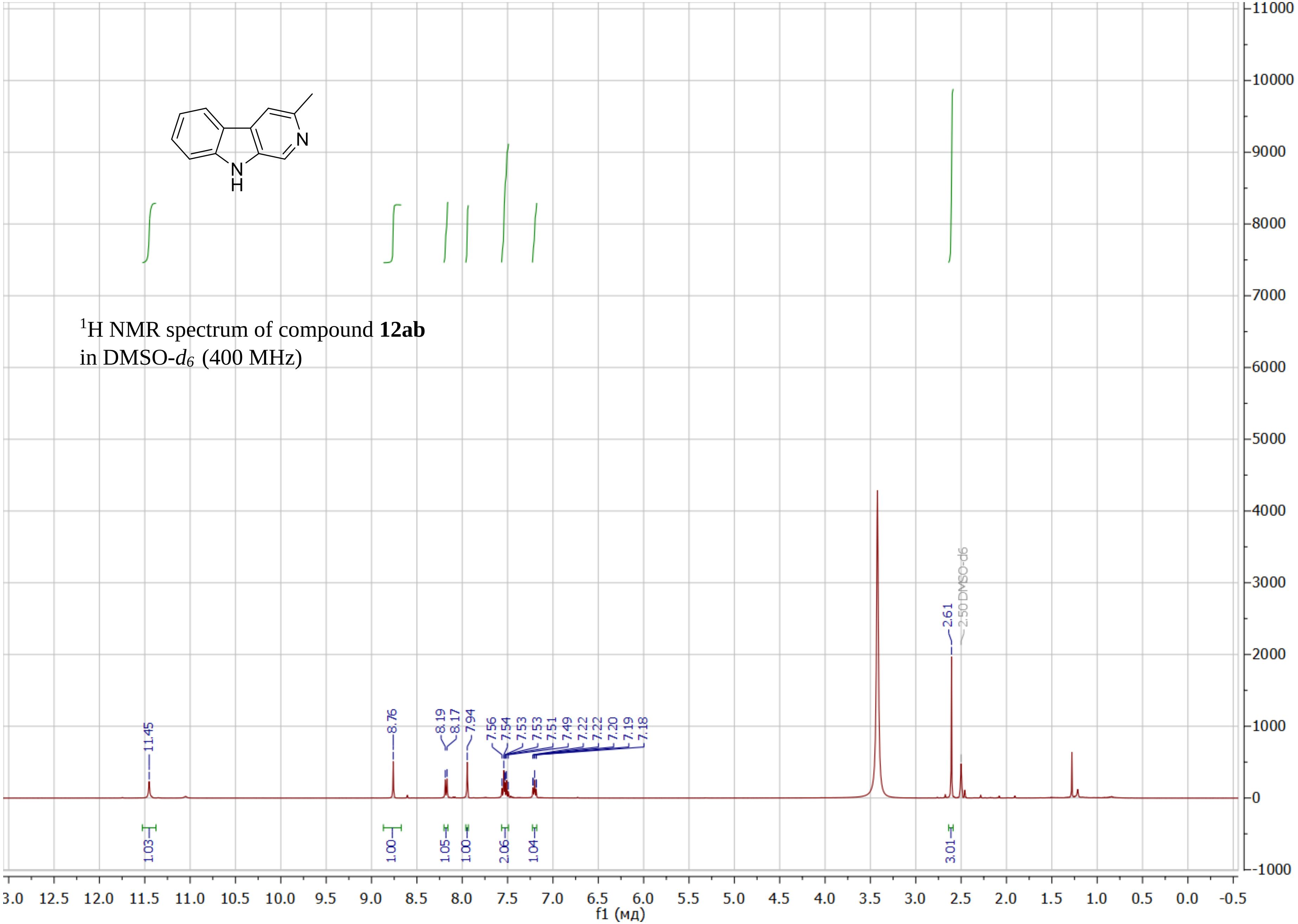

^{1}H NMR spectrum of compound **12ab** in DMSO-d_6 (400 MHz)

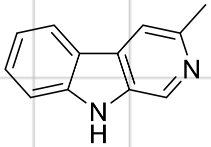

^{13}C {^{1}H} NMR spectrum of compound **12ab** in DMSO-d_6 (101 MHz)

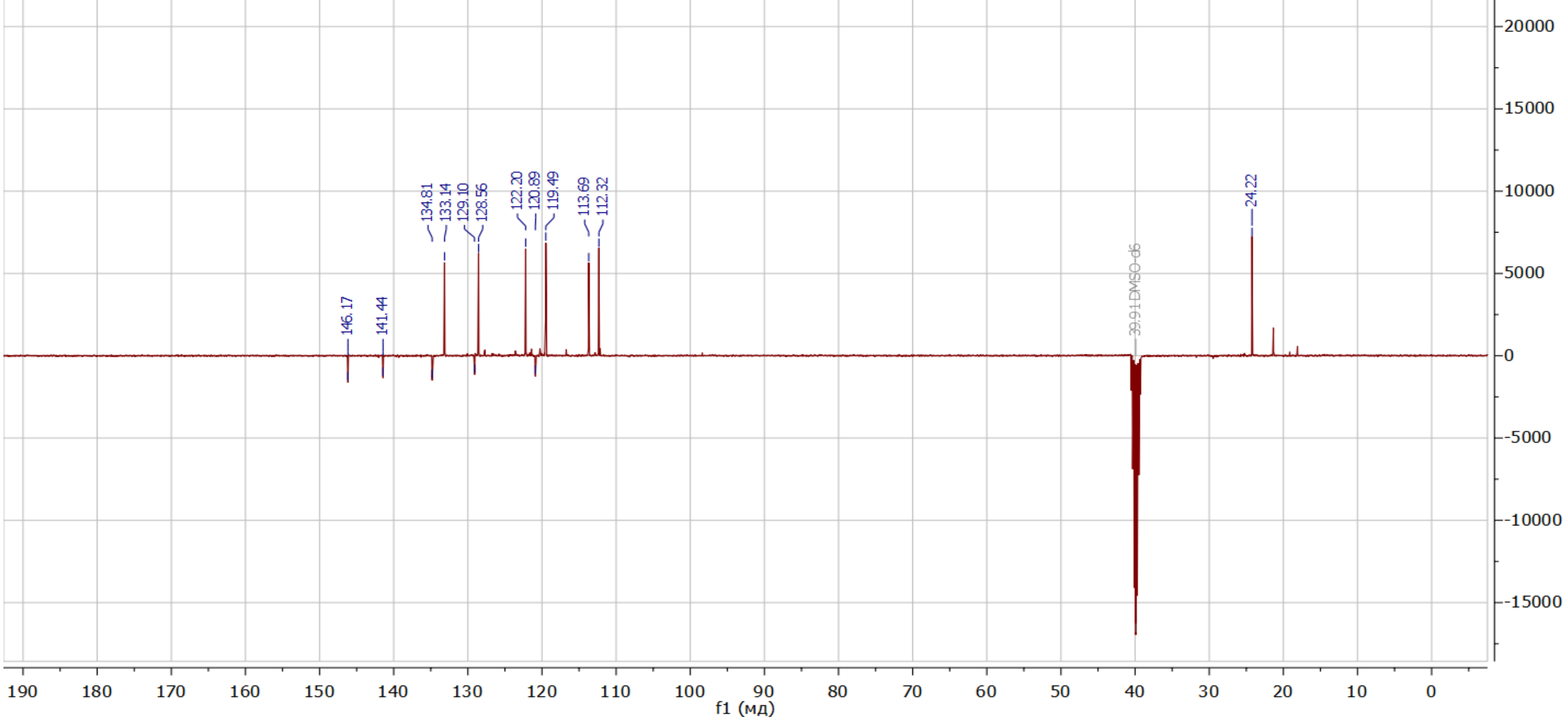

^{1}H NMR spectrum of compound **12ac** in $CDCl_3$ (400 MHz)

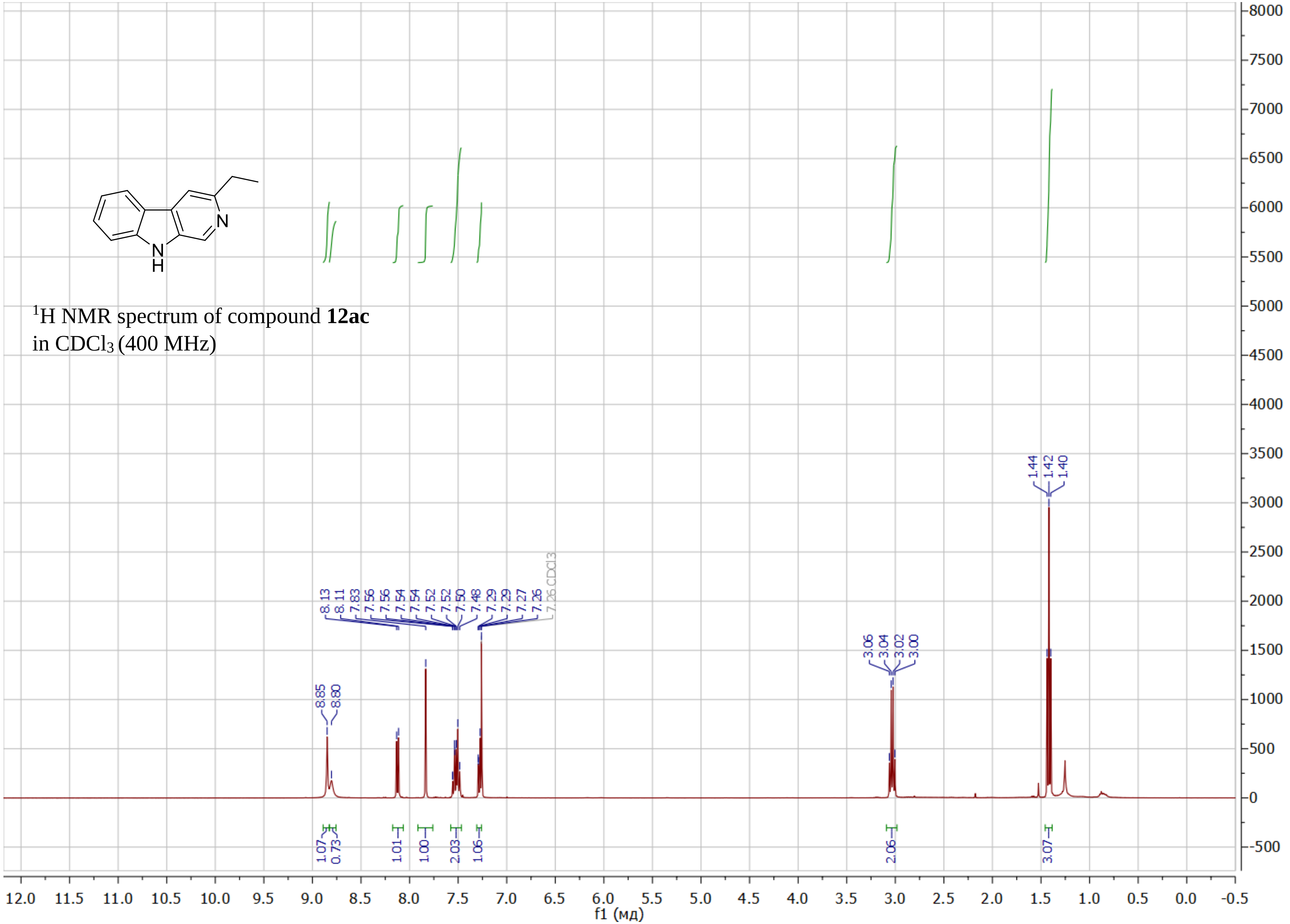

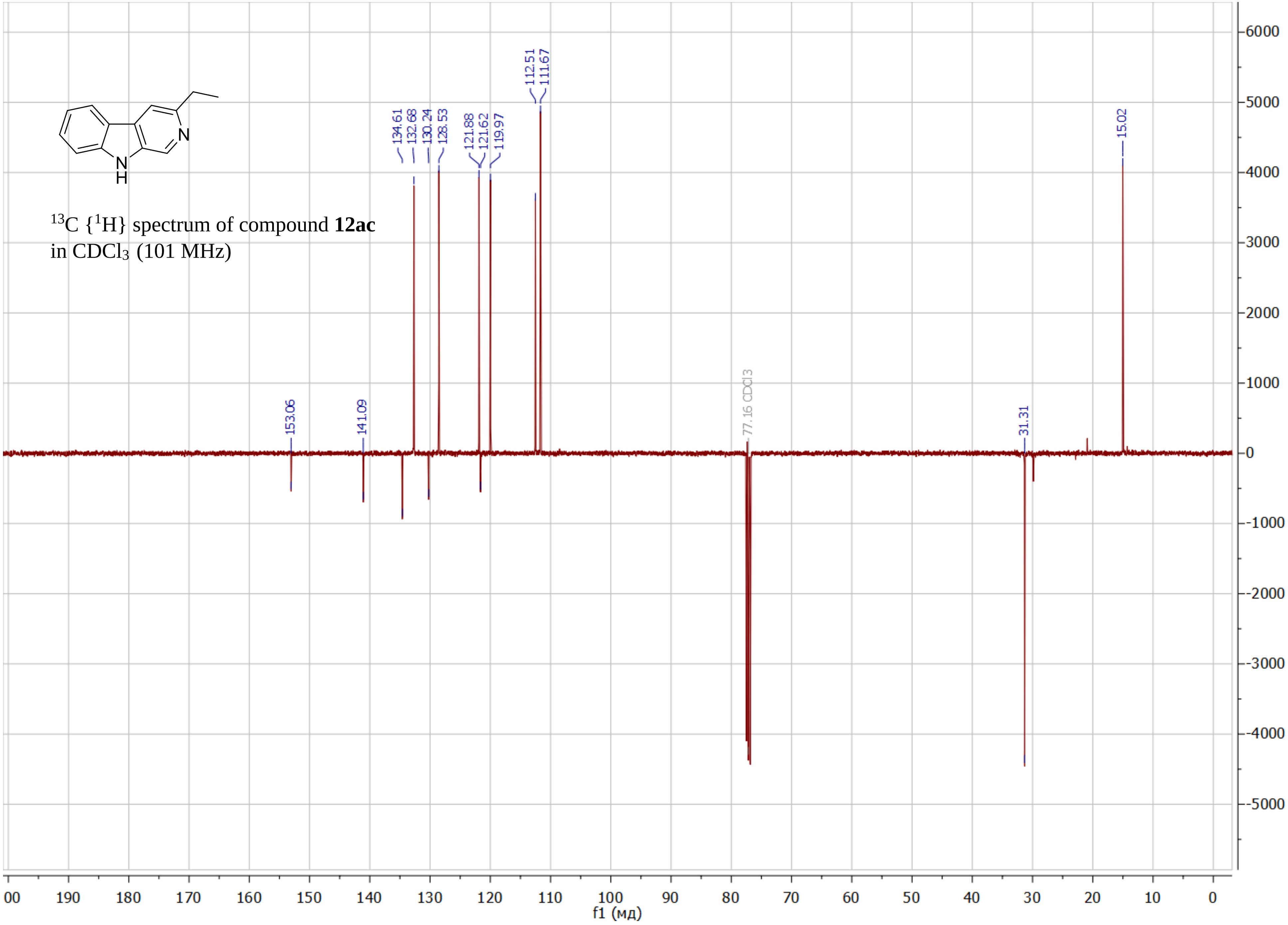

$^{13}C\ \{^{1}H\}$ spectrum of compound **12ac** in $CDCl_3$ (101 MHz)

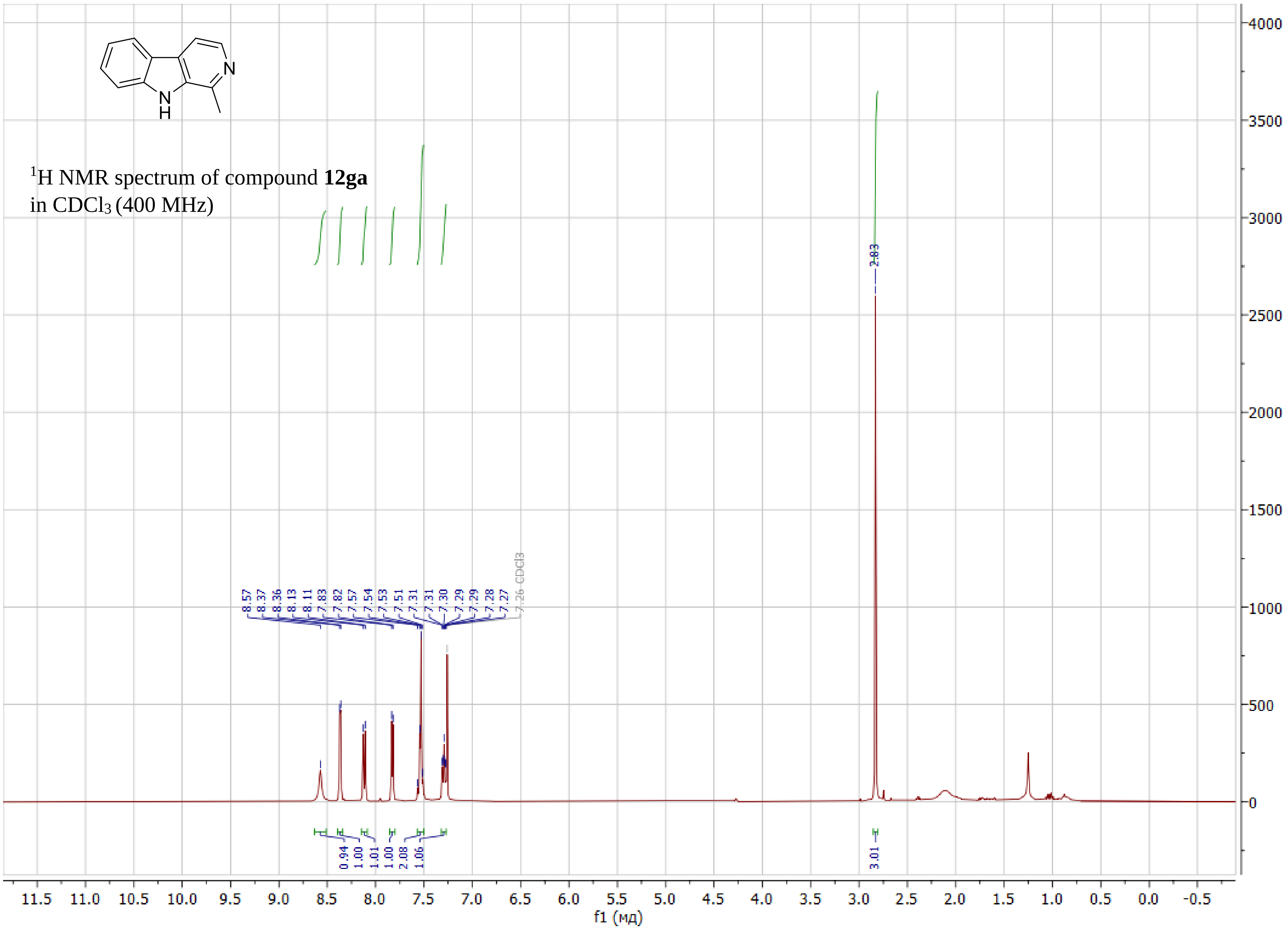

^{1}H NMR spectrum of compound **12ga** in $CDCl_3$ (400 MHz)

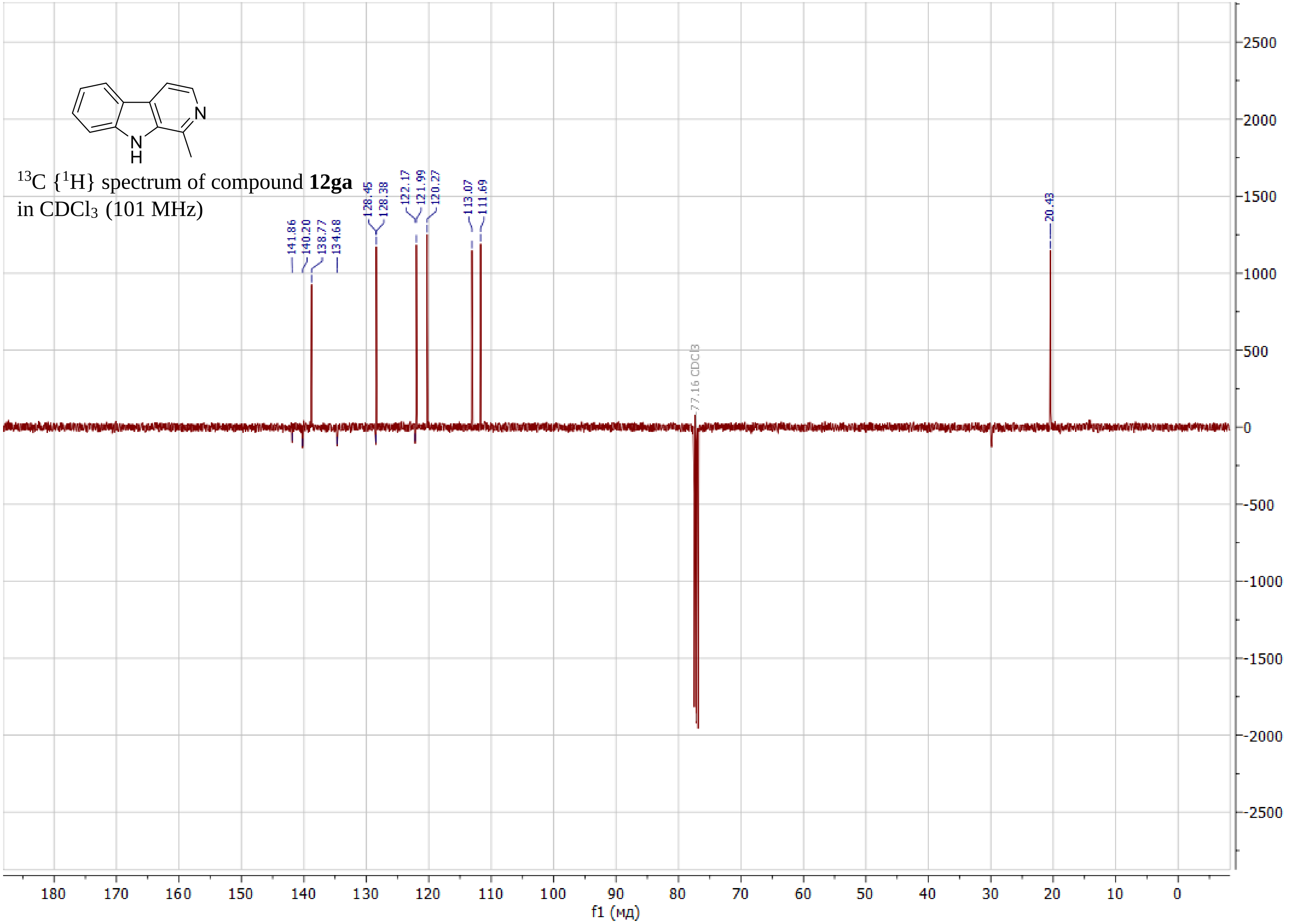

N
N
H
^{13}C {^{1}H} spectrum of compound **12ga**
in $CDCl_3$ (101 MHz)
141.86
140.20
138.77
134.68
128.45
128.38
122.17
121.99
120.27
113.07
111.69
77.16 CDCl3
20.43
f1 (мд)

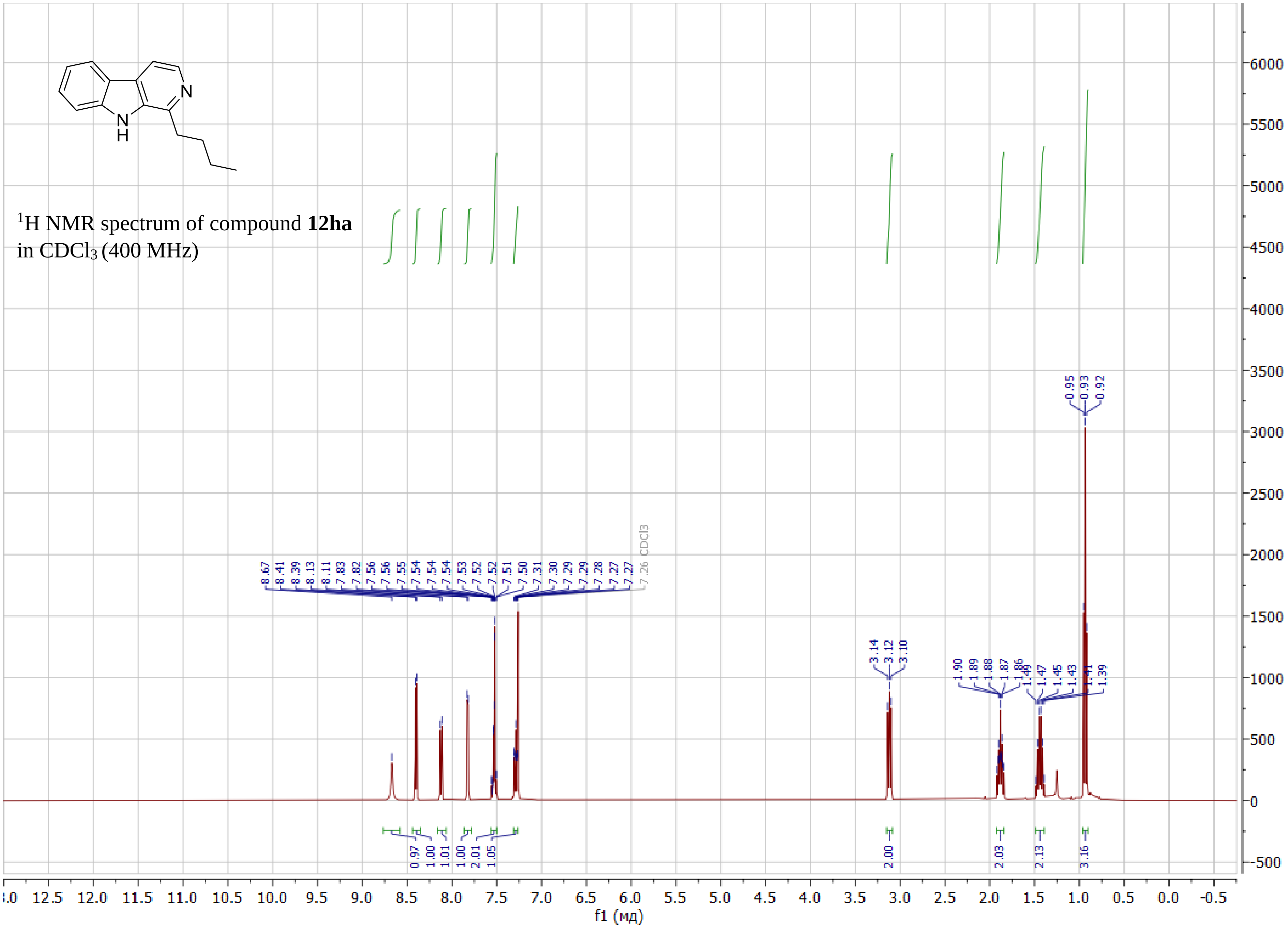

^{1}H NMR spectrum of compound **12ha** in $CDCl_3$ (400 MHz)

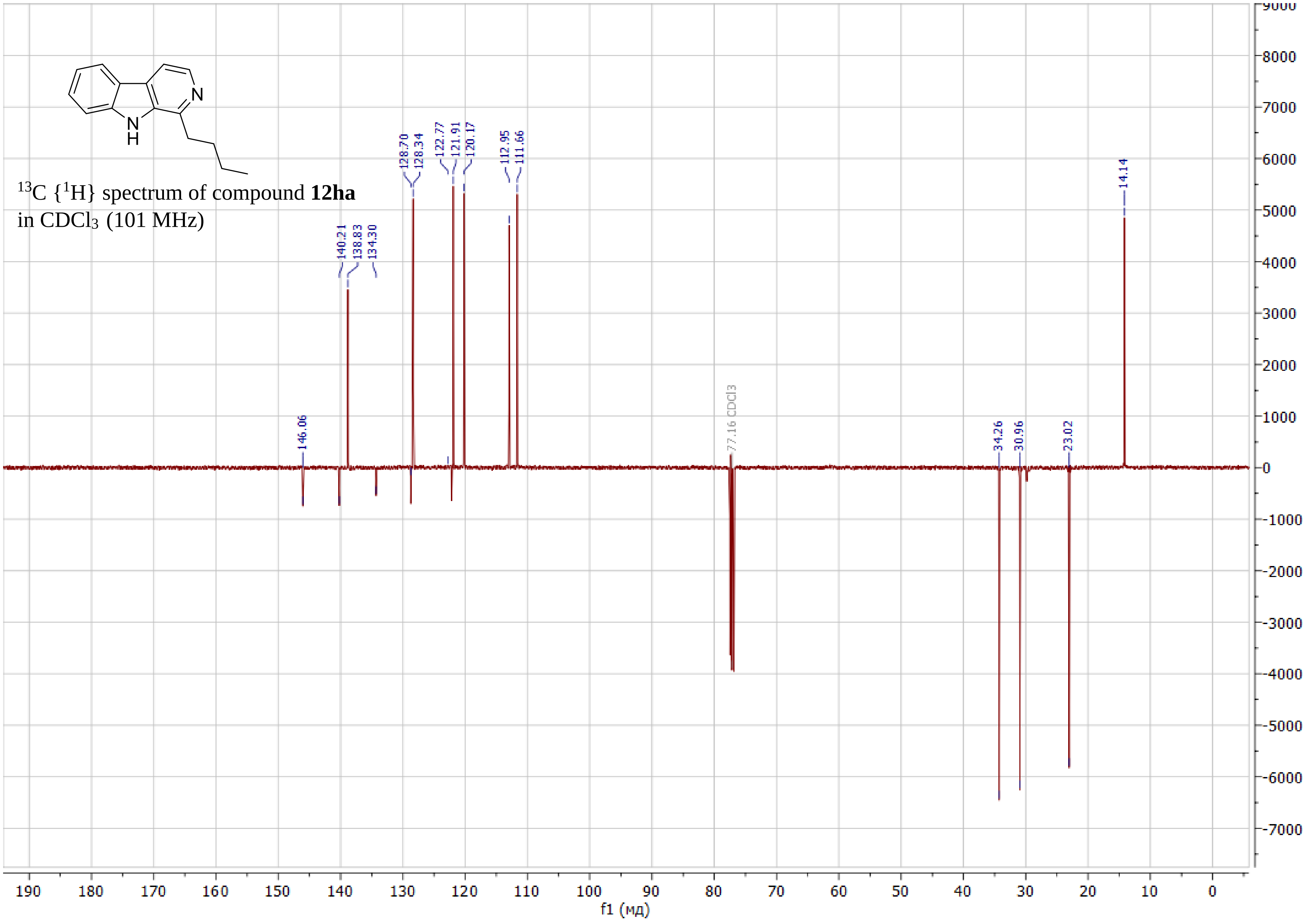

^{13}C {^{1}H} spectrum of compound **12ha** in $CDCl_3$ (101 MHz)

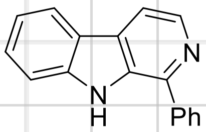

^{1}H NMR spectrum of compound **12ia** in DMSO-d_6 (400 MHz)

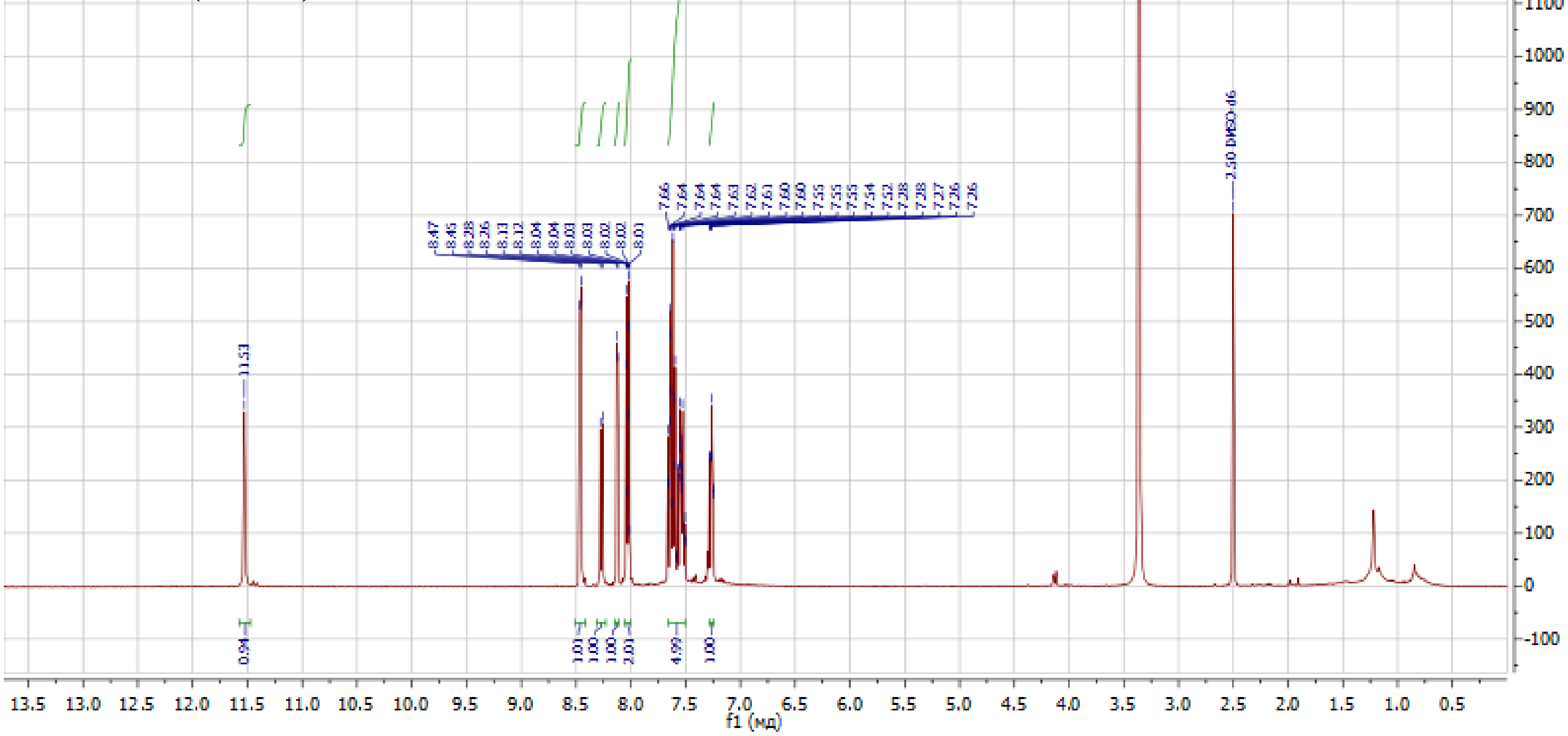

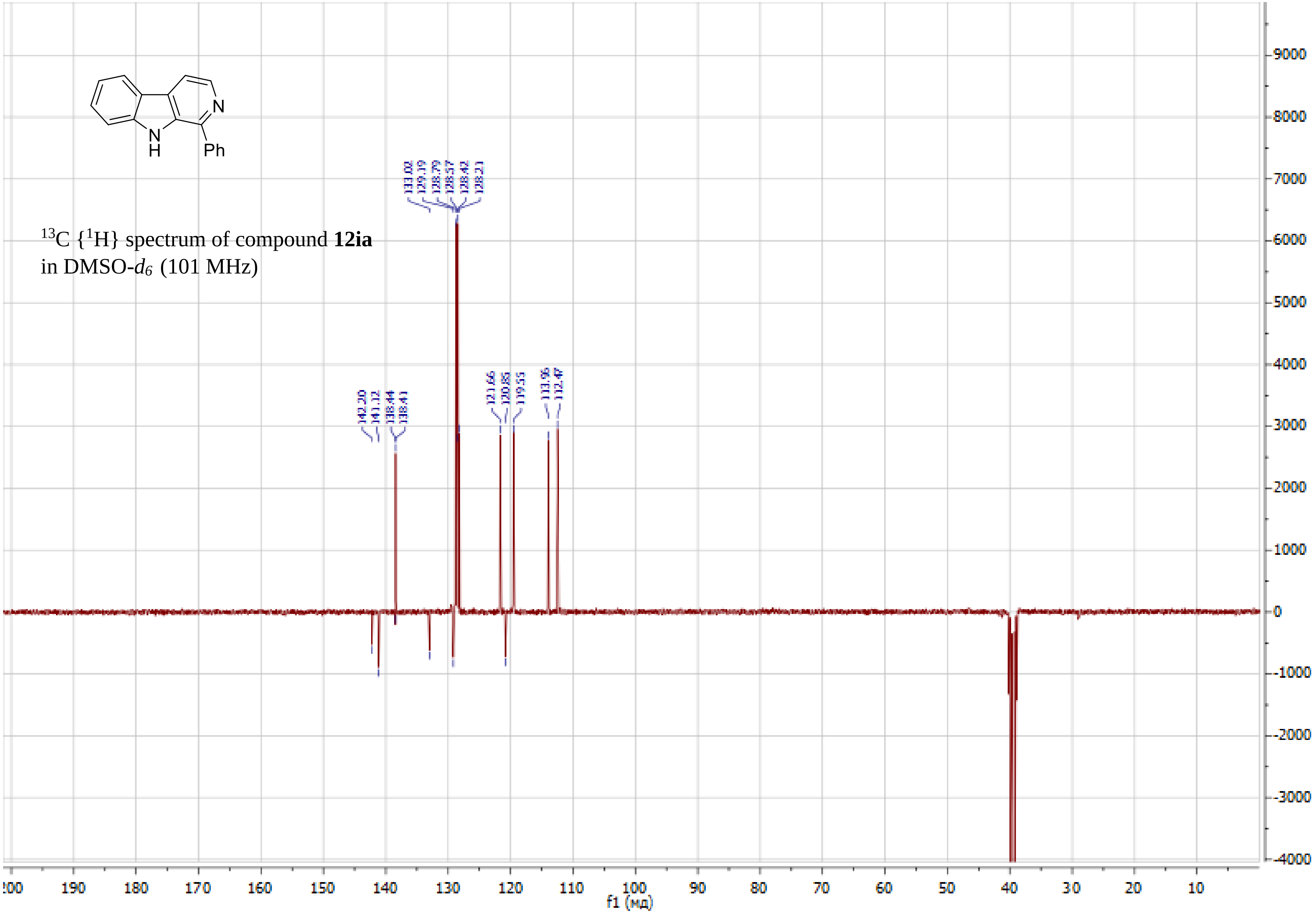

N
H
Ph
^{13}C {^{1}H} spectrum of compound **12ia**
in DMSO-d_6 (101 MHz)
133.02
129.19
128.79
128.57
128.42
128.21
142.20
141.12
138.44
138.41
121.66
120.85
119.55
113.96
112.47
9000
8000
7000
6000
5000
4000
3000
2000
1000
0
-1000
-2000
-3000
-4000
200
190
180
170
160
150
140
130
120
110
100
90
80
70
60
50
40
30
20
10
f1 (мд)

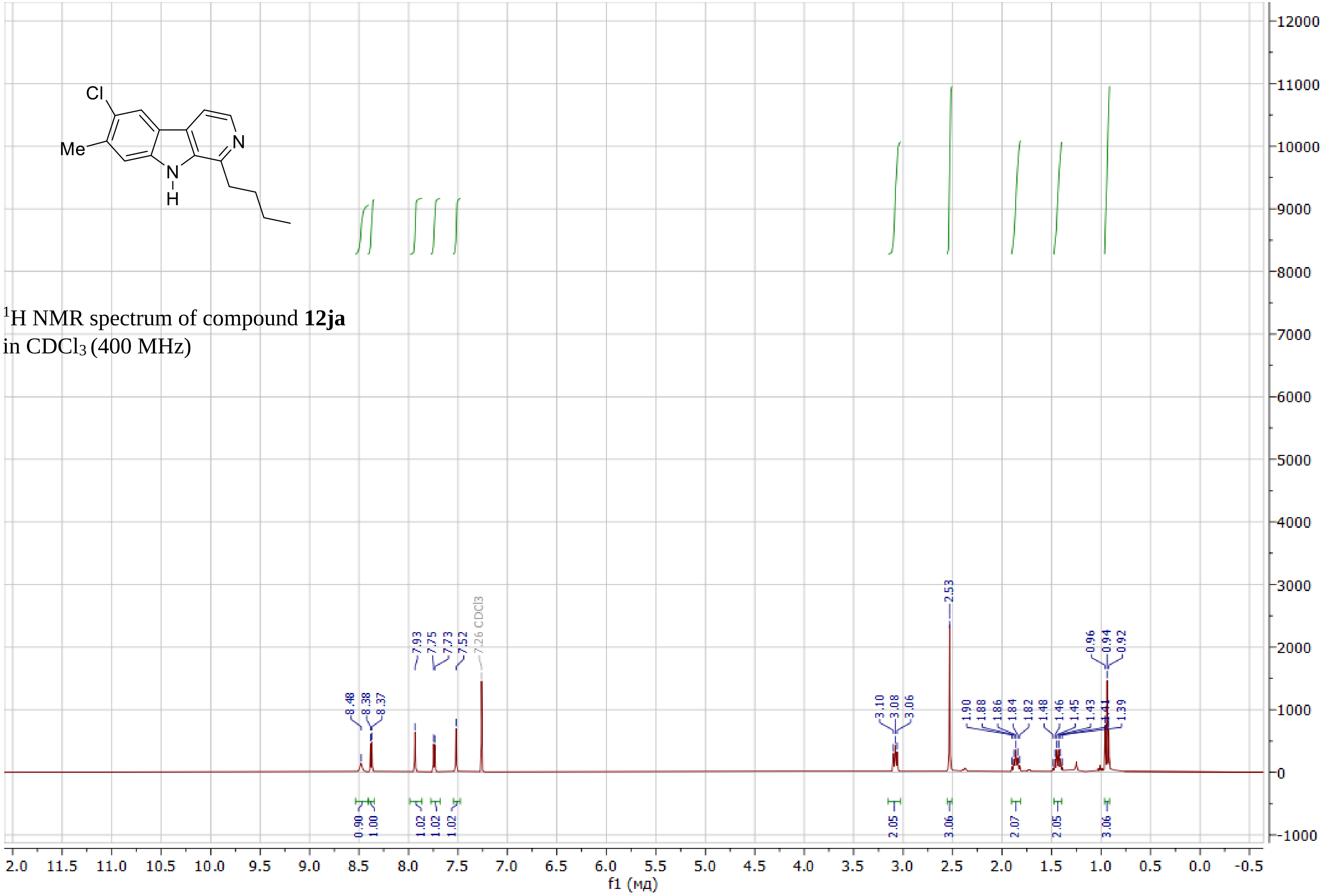

^{1}H NMR spectrum of compound **12ja** in $CDCl_3$ (400 MHz)

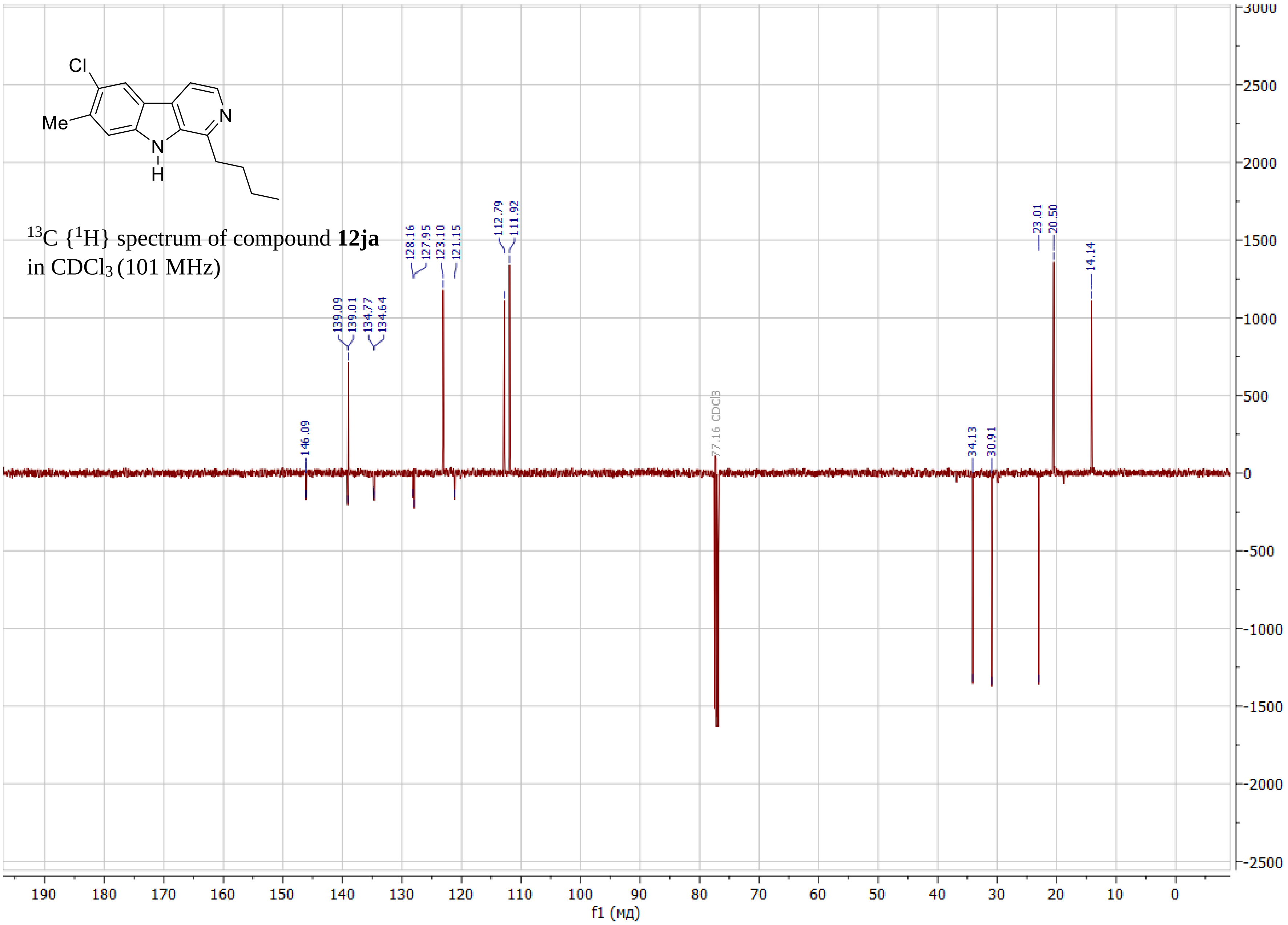

$^{13}C\ \{^{1}H\}$ spectrum of compound **12ja** in $CDCl_3$ (101 MHz)

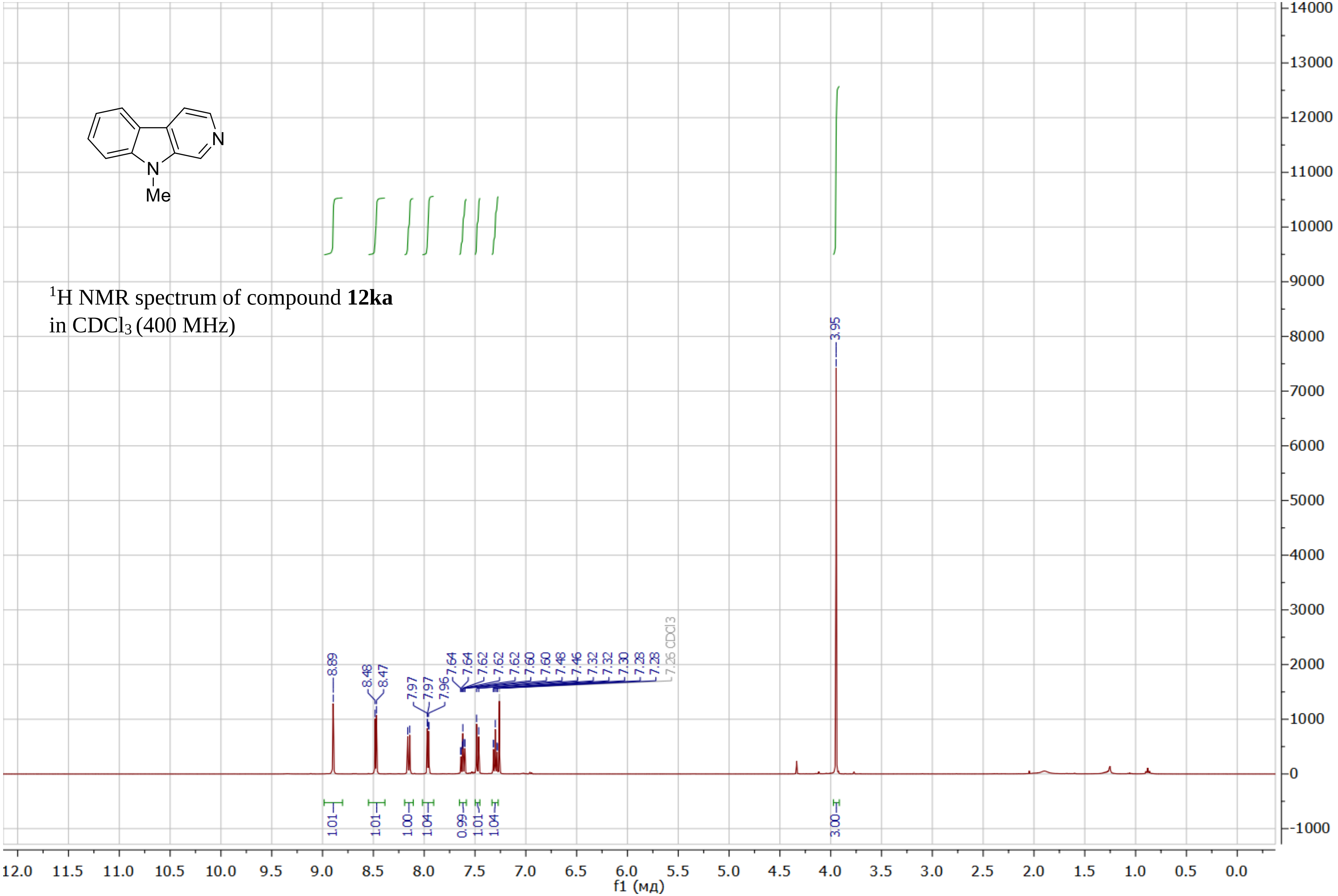

^{1}H NMR spectrum of compound **12ka** in $CDCl_3$ (400 MHz)

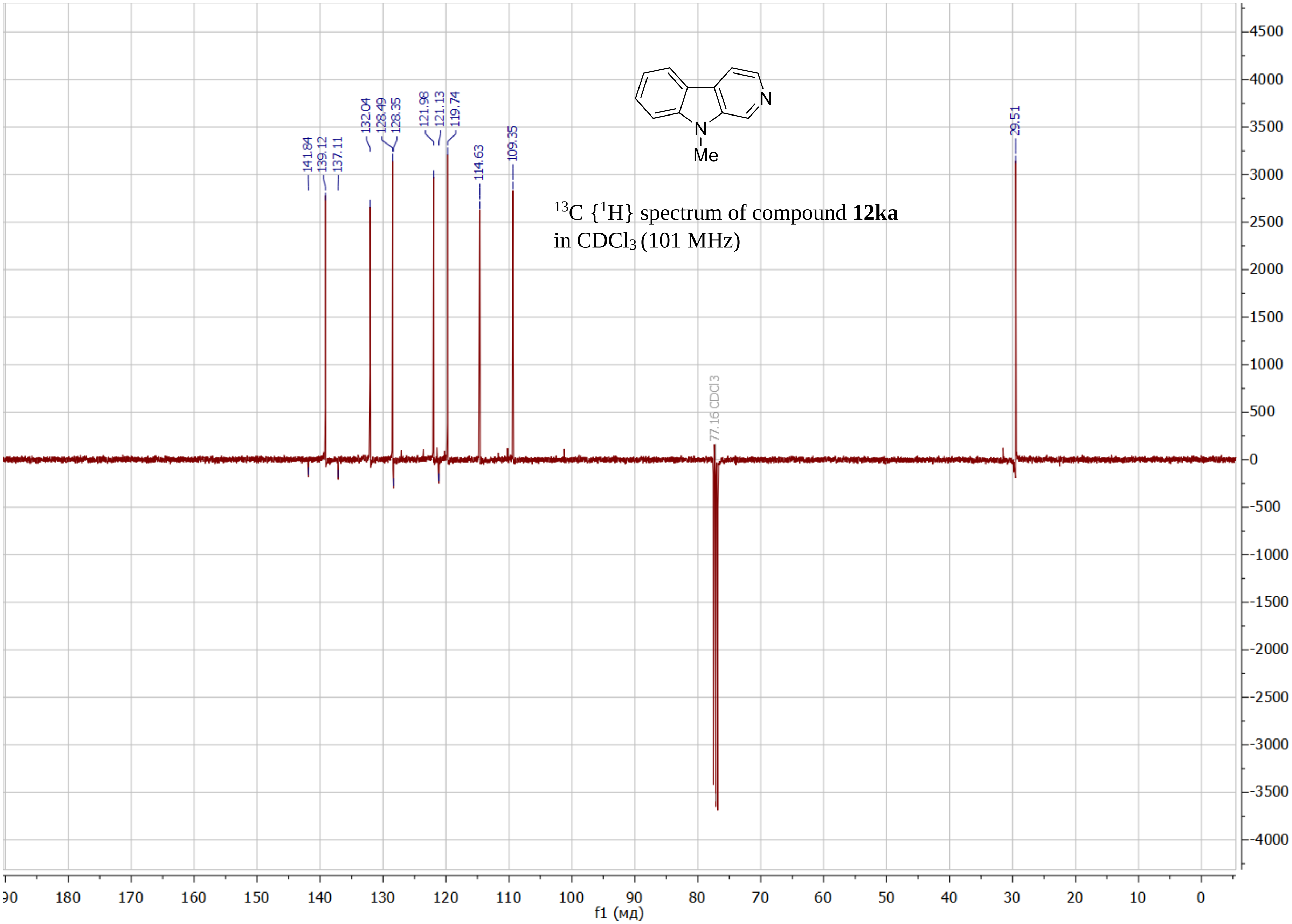

141.84
139.12
137.11
132.04
128.49
128.35
121.98
121.13
119.74
114.63
109.35
29.51
77.16 CDCl3
N
N
Me
13C {1H} spectrum of compound 12ka
in CDCl3 (101 MHz)
f1 (мд)

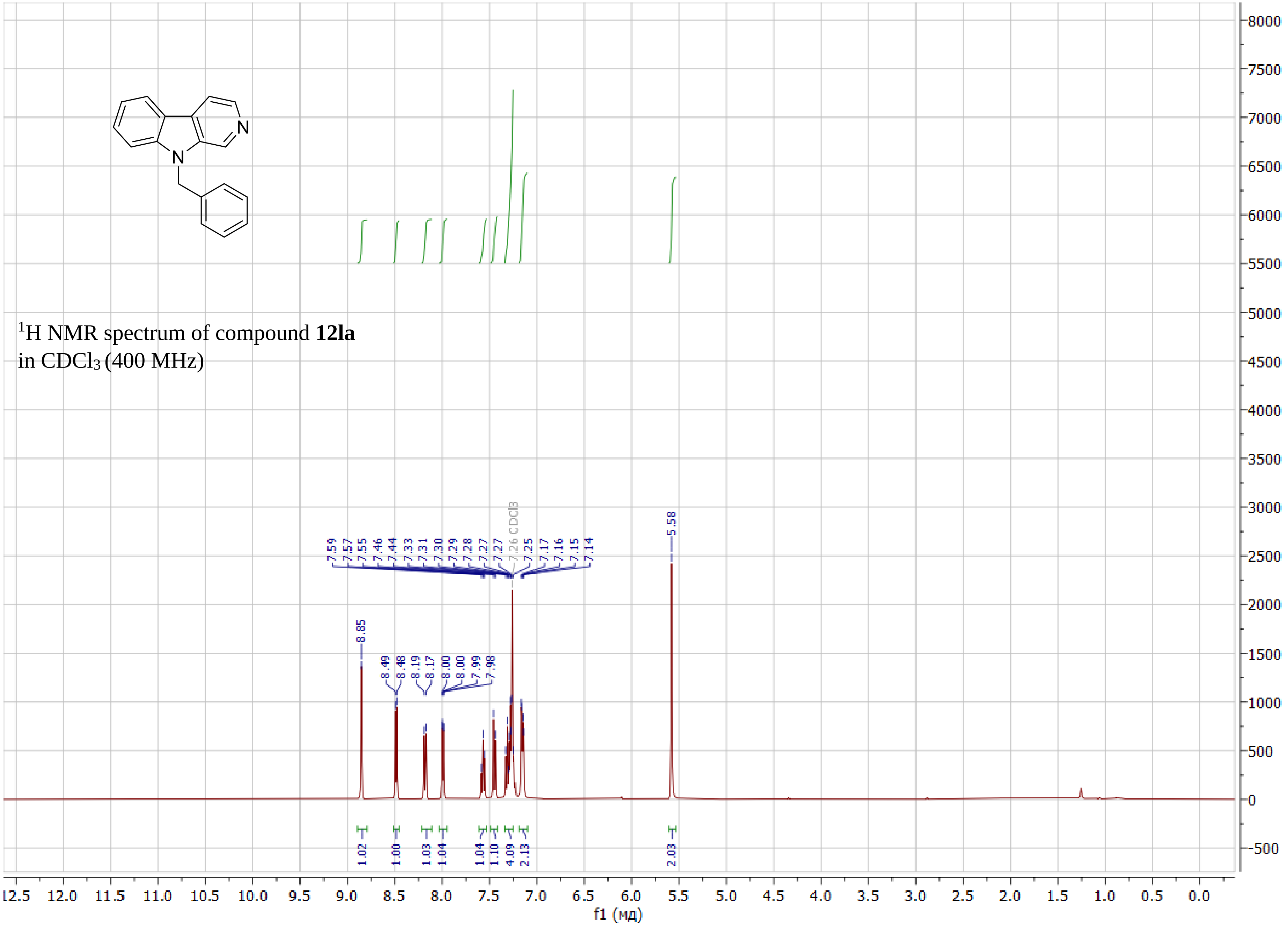

^{1}H NMR spectrum of compound **12la** in $CDCl_3$ (400 MHz)

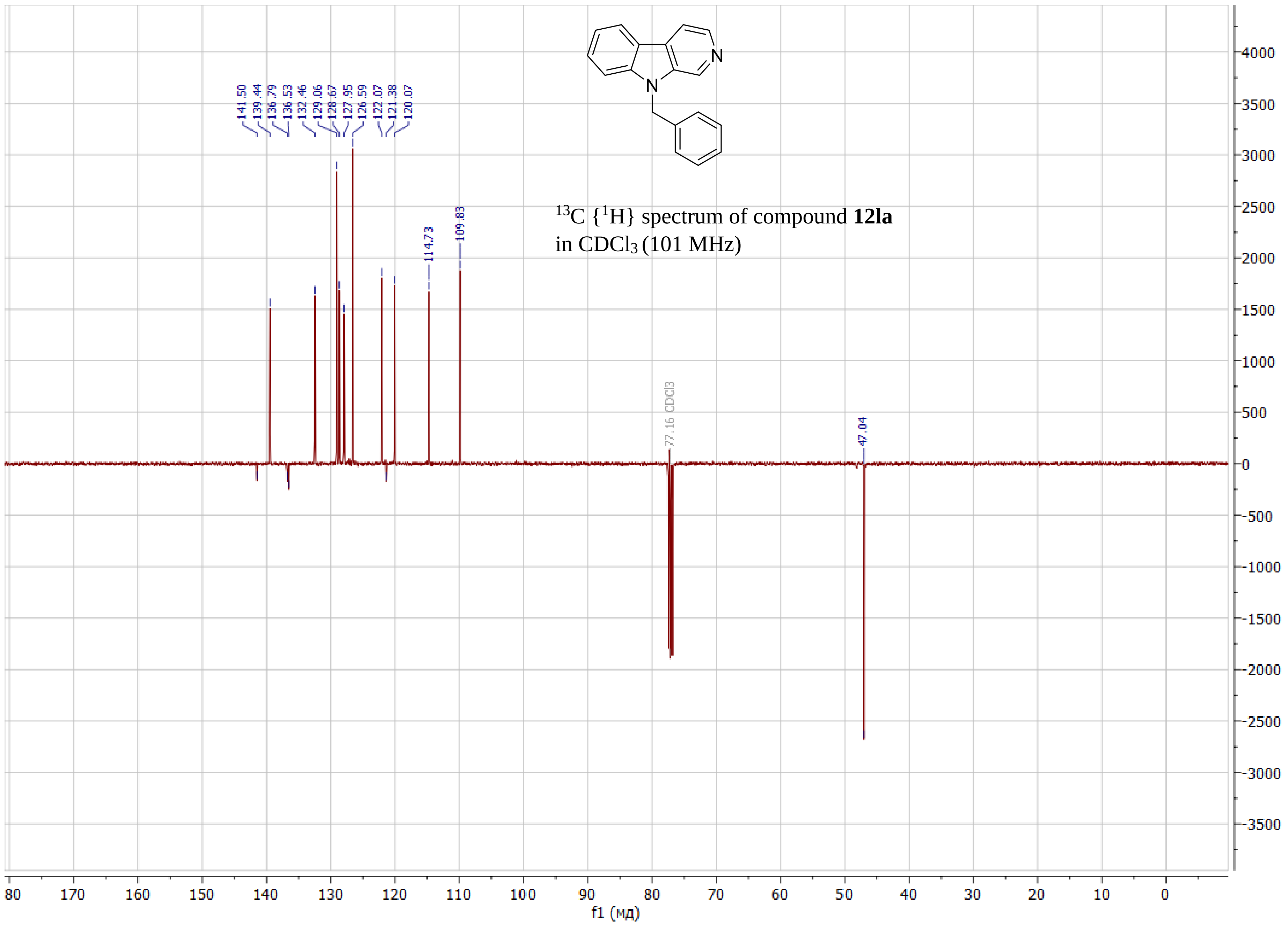

^{13}C {^{1}H} spectrum of compound **12la**
in $CDCl_3$ (101 MHz)
141.50
139.44
136.79
136.53
132.46
129.06
128.67
127.95
126.59
122.07
121.38
120.07
114.73
109.83
77.16 CDCl3
47.04
f1 (мд)

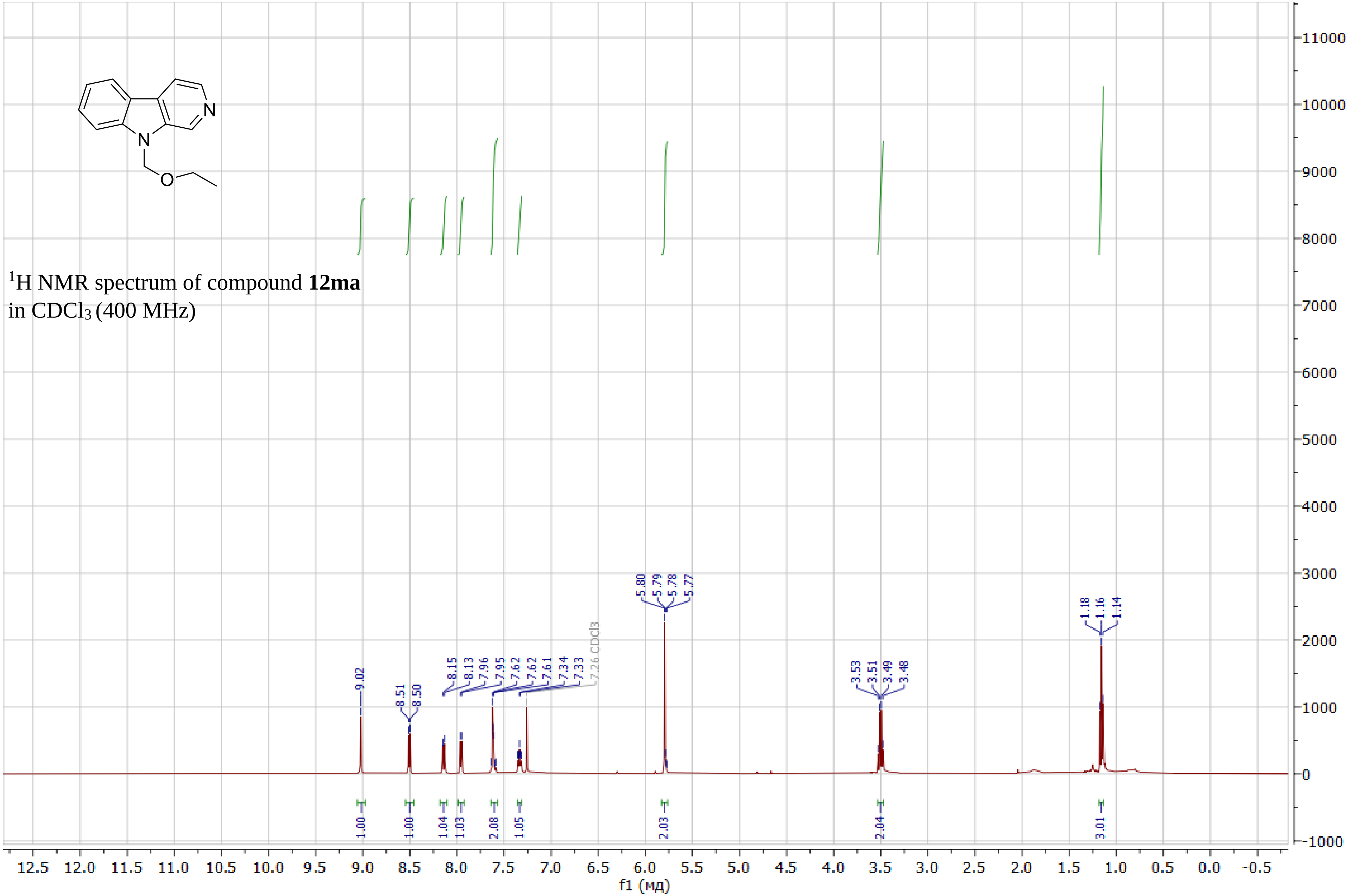

^{1}H NMR spectrum of compound **12ma** in $CDCl_3$ (400 MHz)

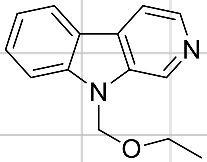

^{13}C {^{1}H} spectrum of compound **12ma** in $CDCl_3$ (101 MHz)

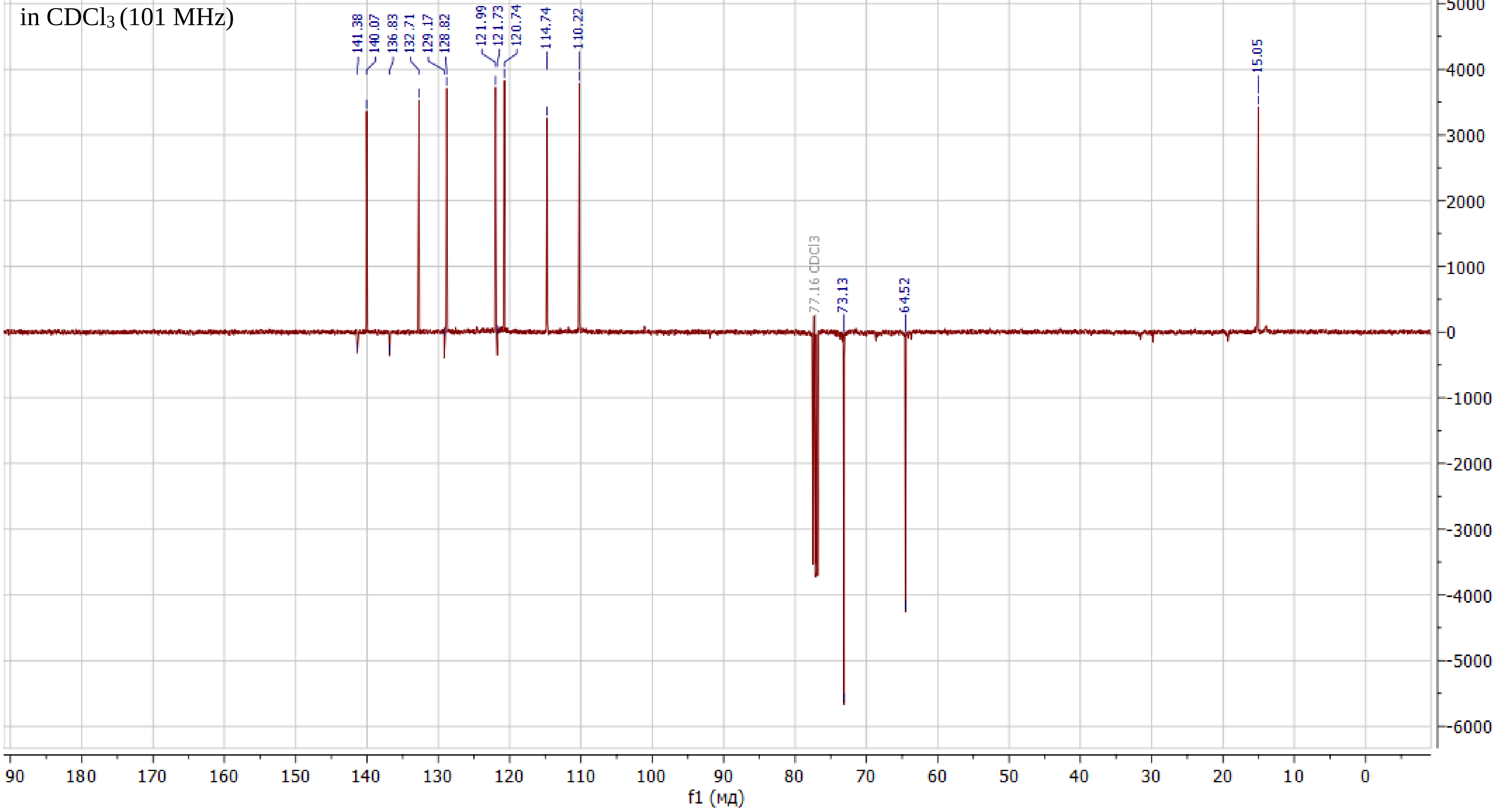

^{1}H NMR spectrum of compound **12kc**
in $CDCl_3$ (400 MHz)

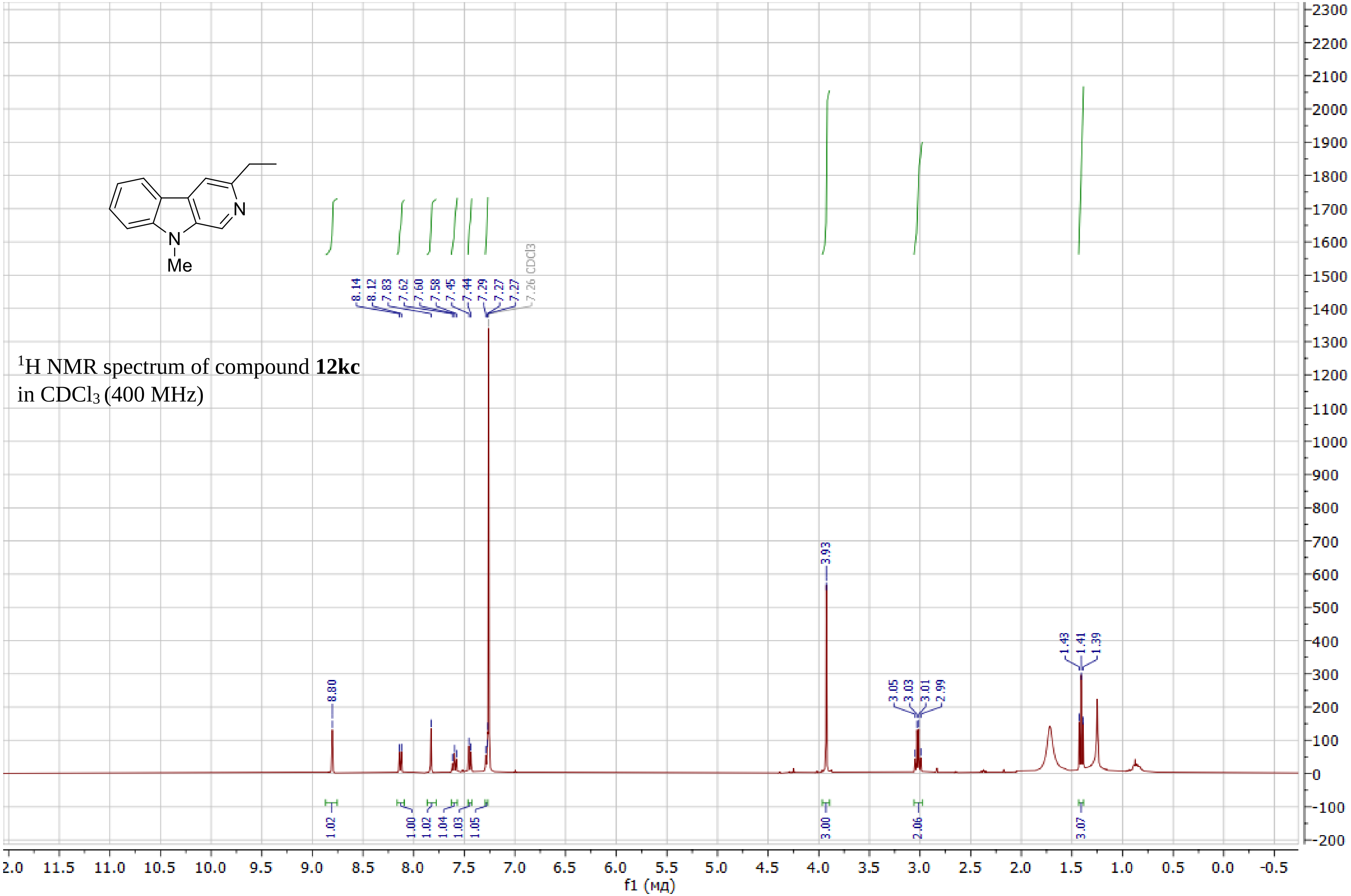

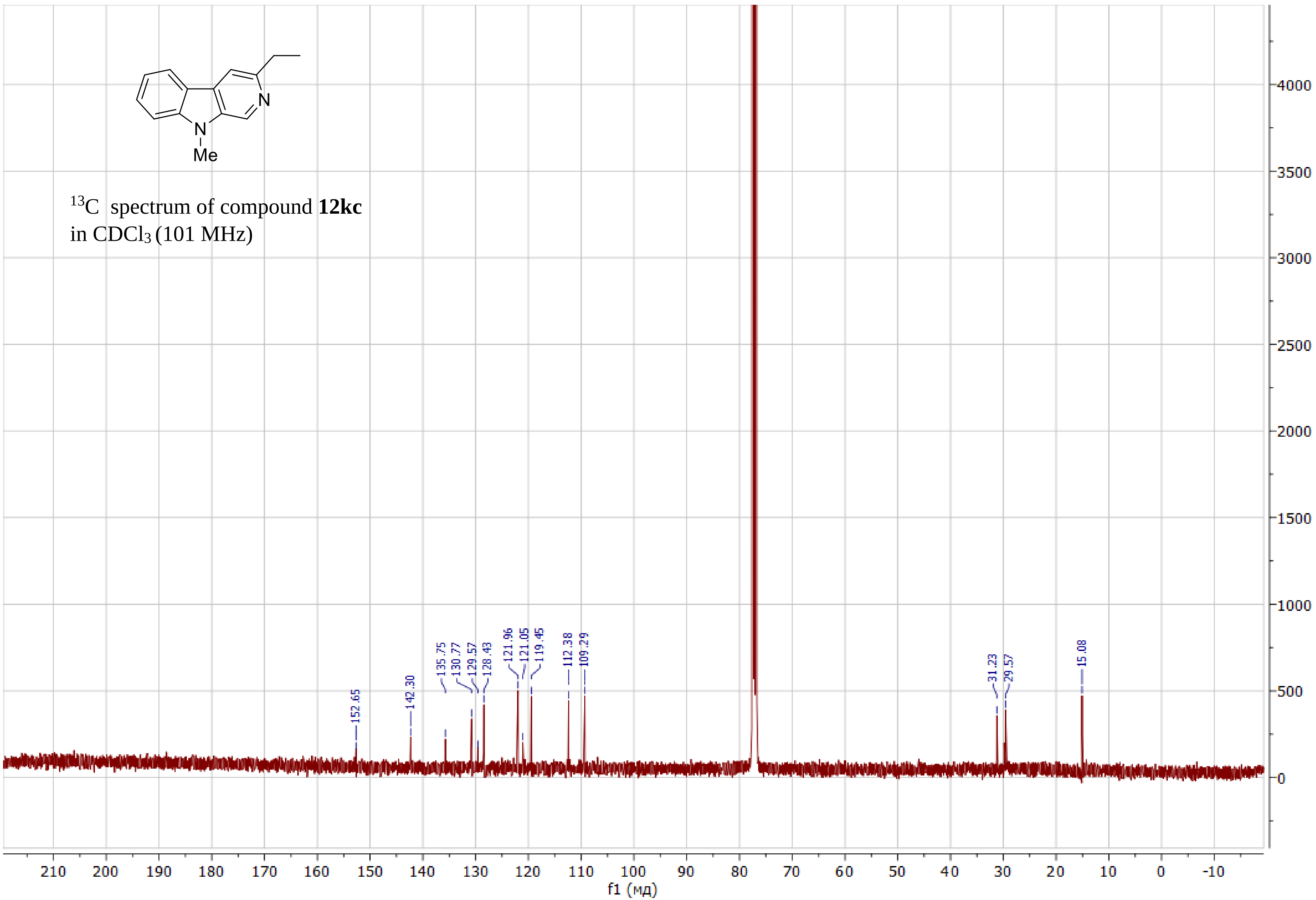

^{13}C spectrum of compound **12kc** in $CDCl_3$ (101 MHz)

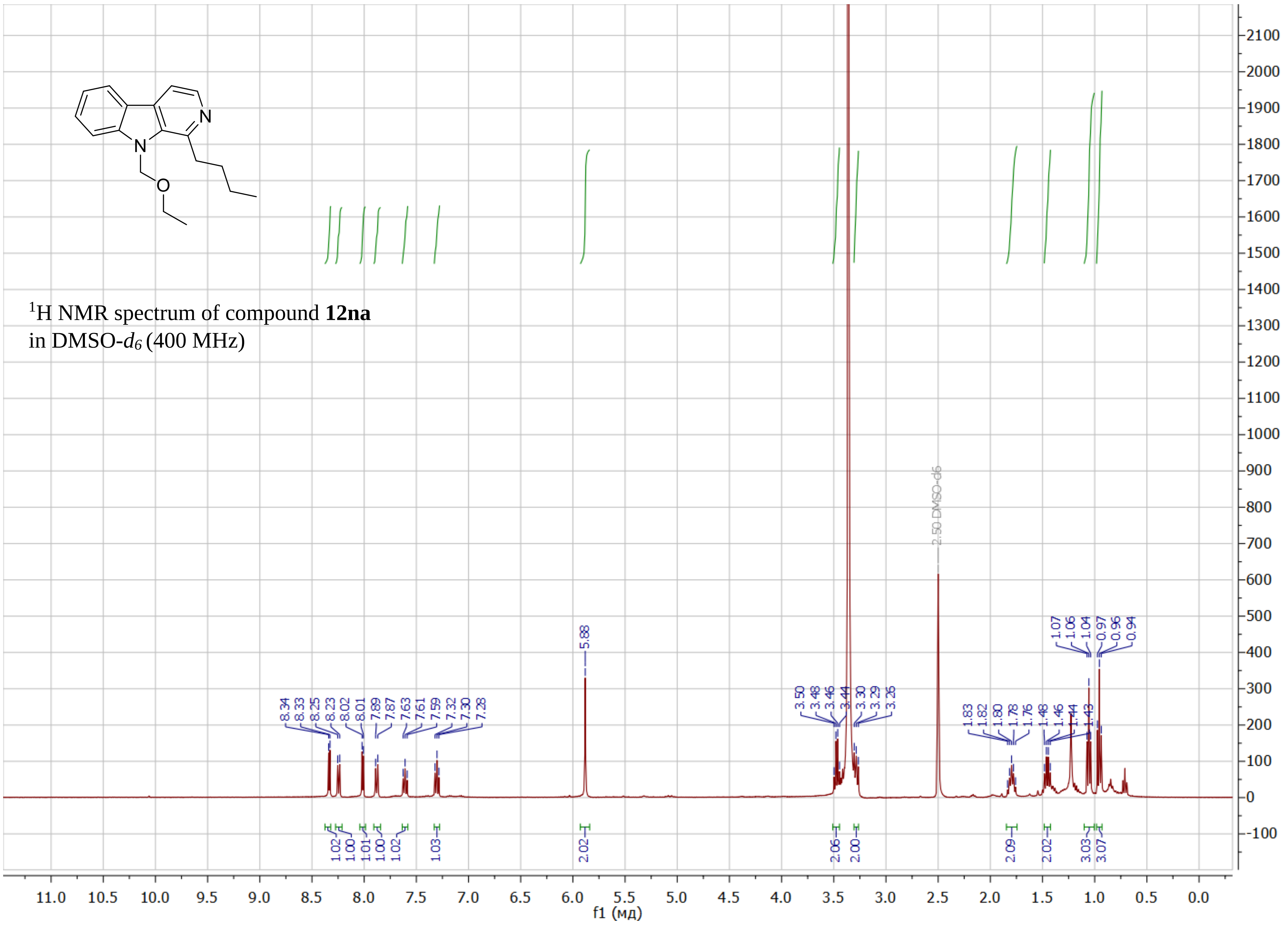

^{1}H NMR spectrum of compound **12na** in DMSO-d_6 (400 MHz)

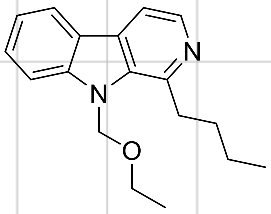

^{13}C {^{1}H} spectrum of compound **12na**
in DMSO-d_6 (101 MHz)

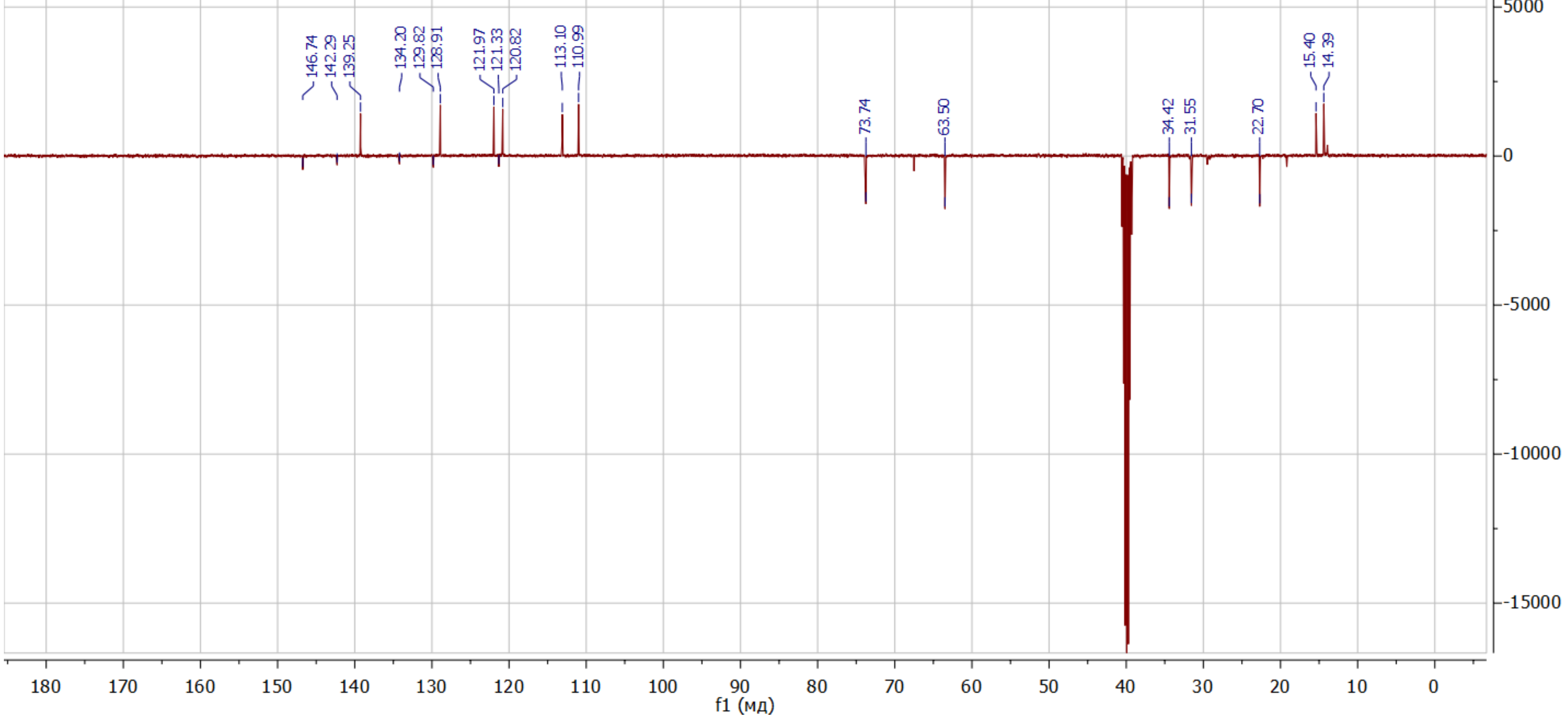

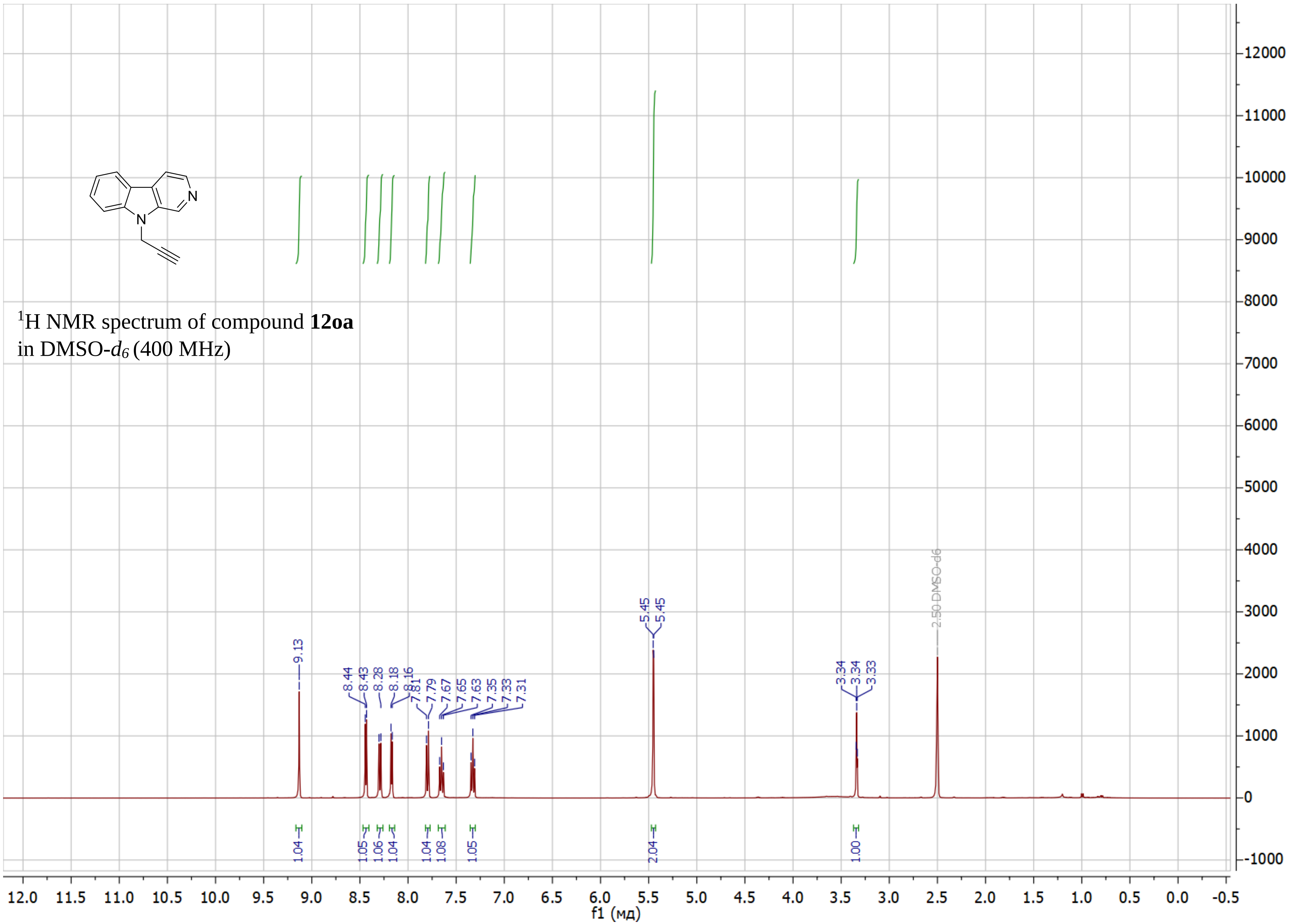

^{1}H NMR spectrum of compound **12oa** in DMSO-d_6 (400 MHz)

^{13}C spectrum of compound **12oa** in DMSO-d_6 (101 MHz)

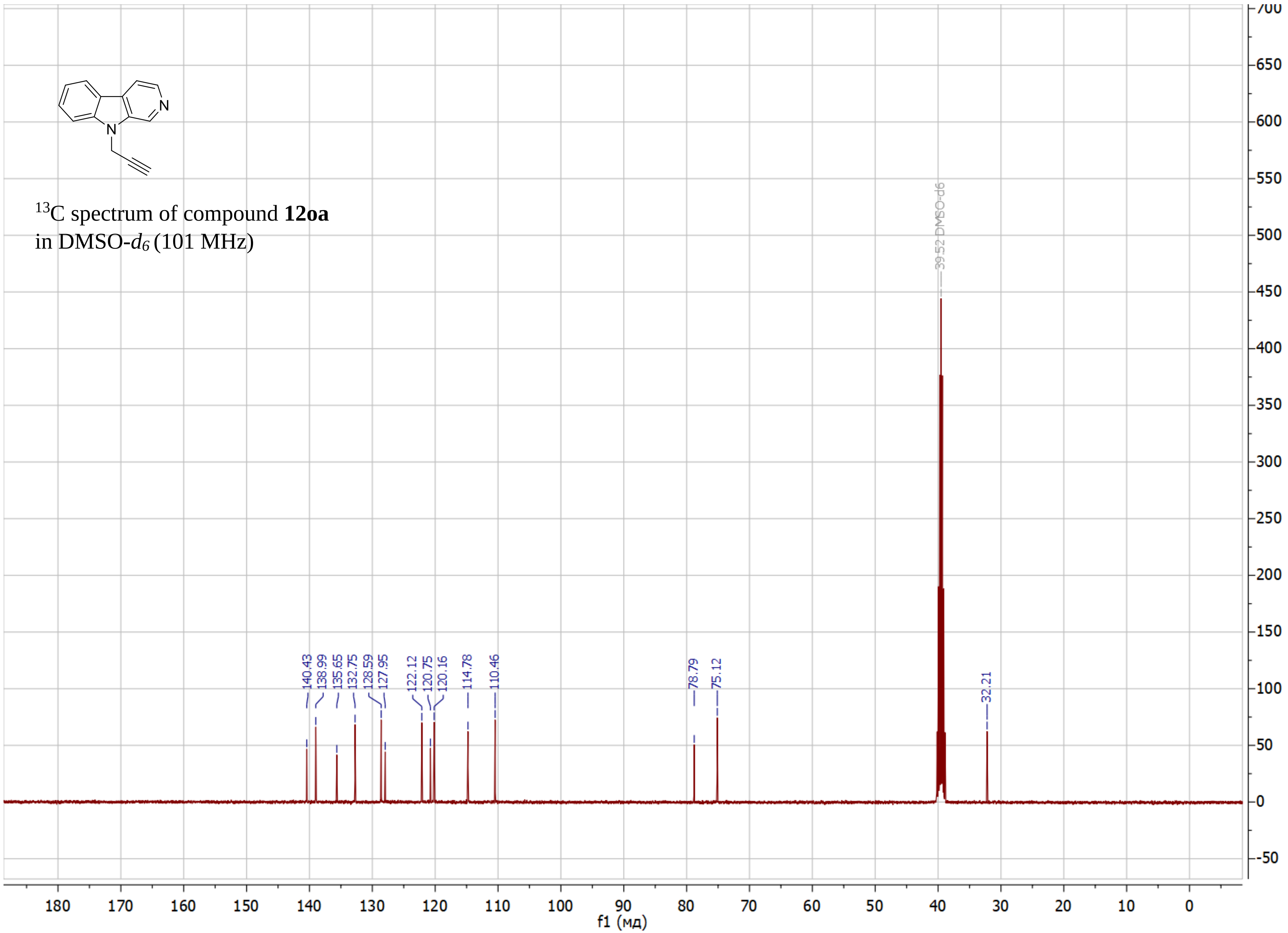

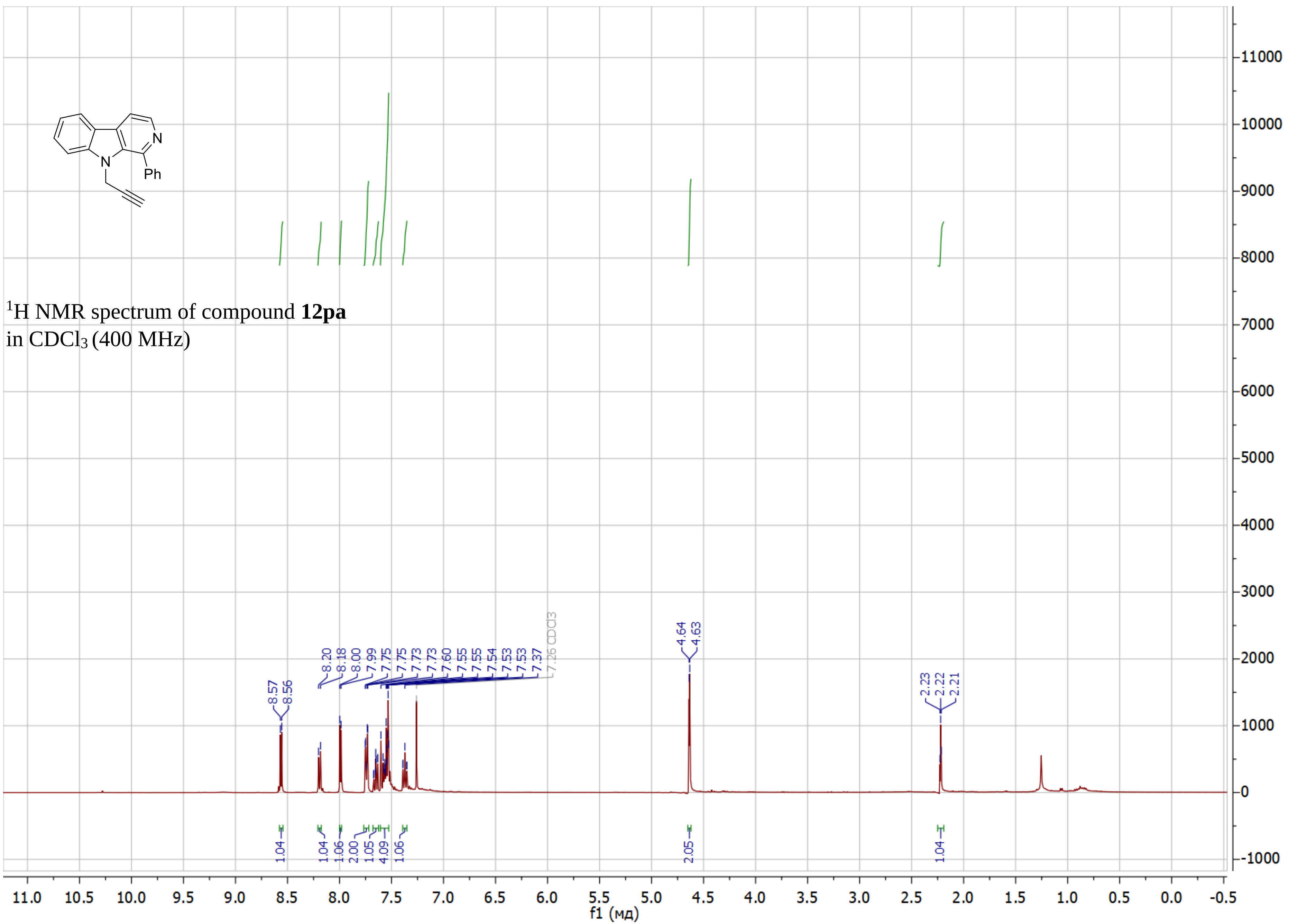

^{1}H NMR spectrum of compound **12pa** in $CDCl_3$ (400 MHz)

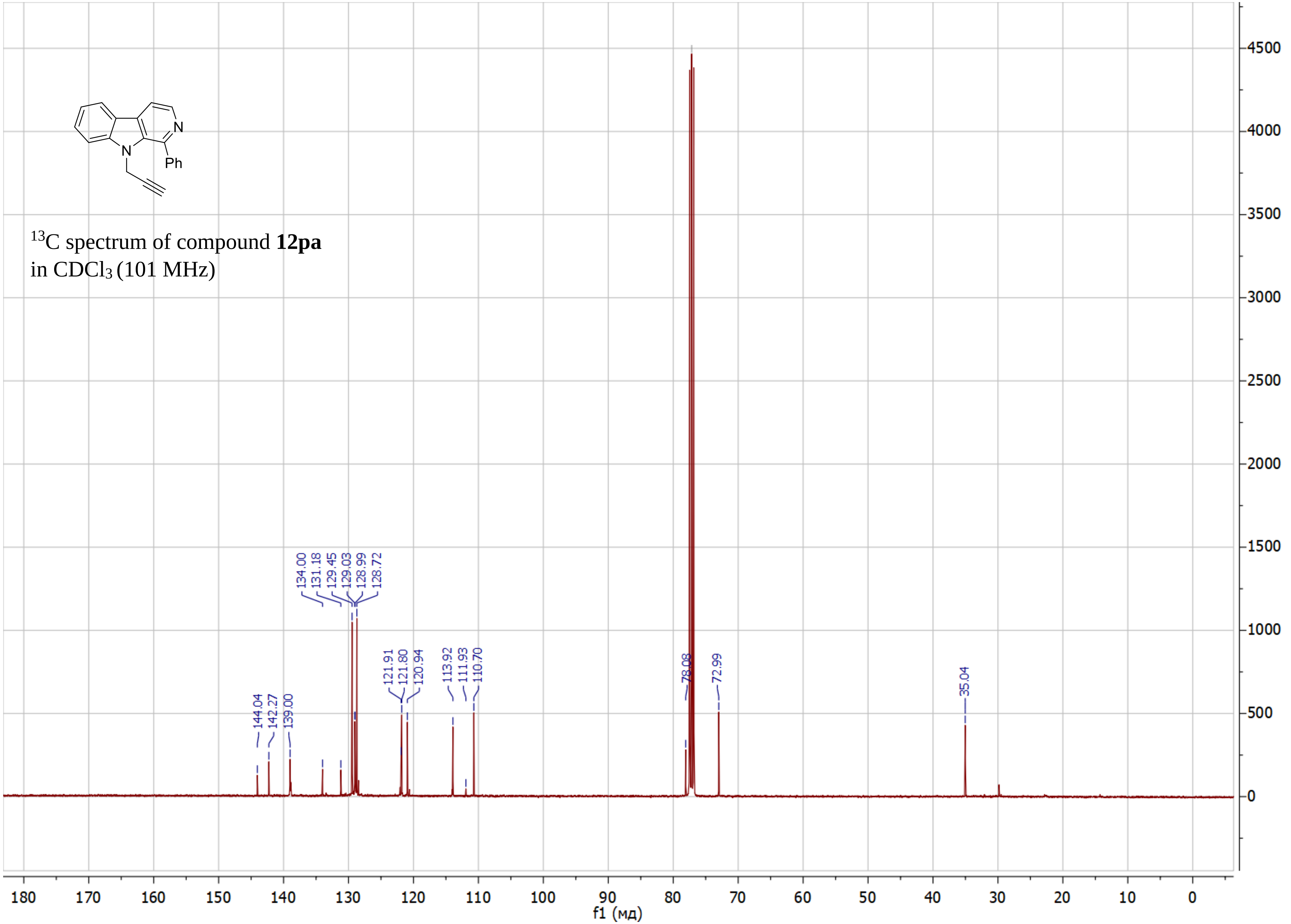

^{13}C spectrum of compound **12pa** in $CDCl_3$ (101 MHz)

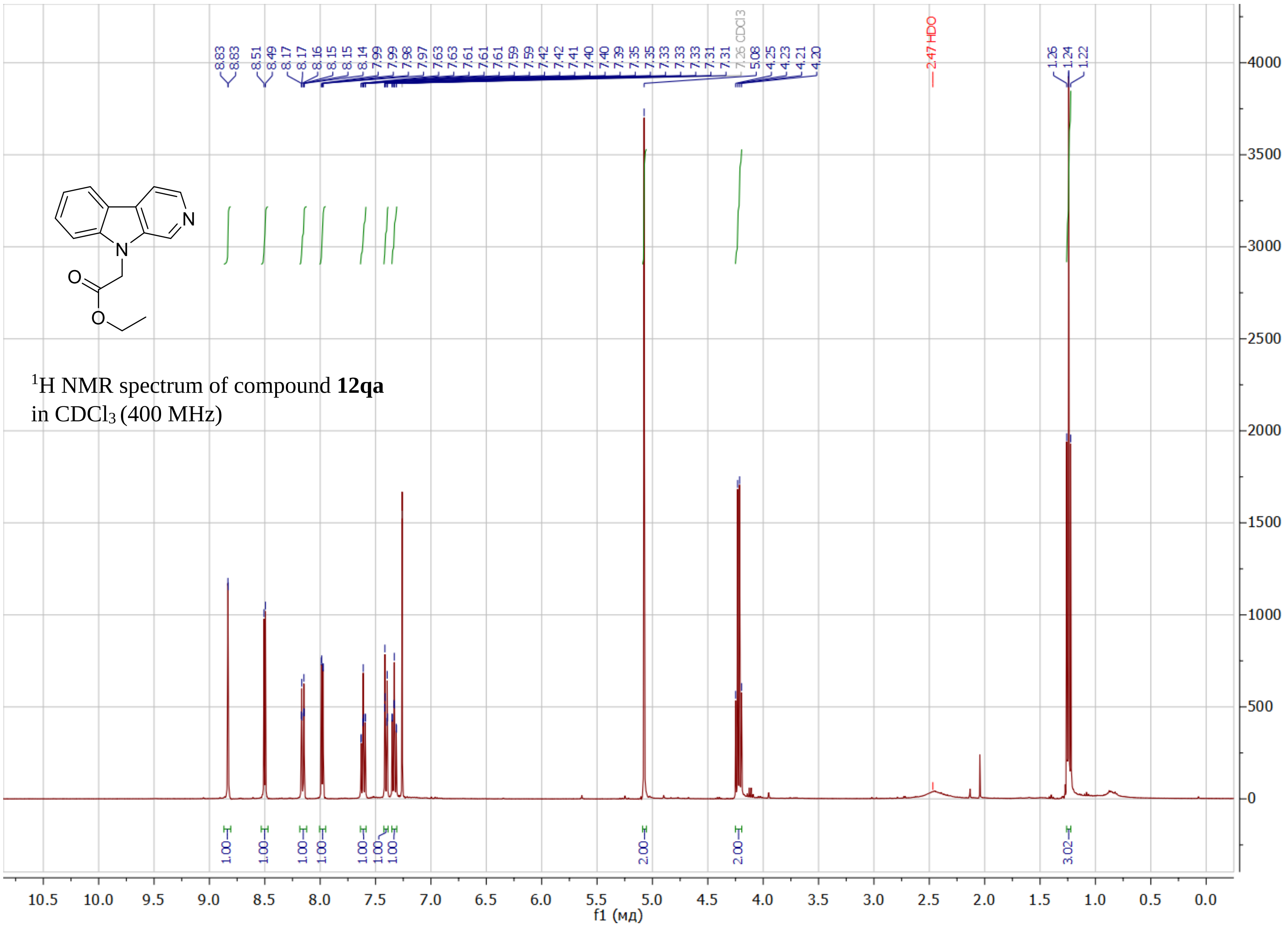

^{1}H NMR spectrum of compound **12qa** in $CDCl_3$ (400 MHz)

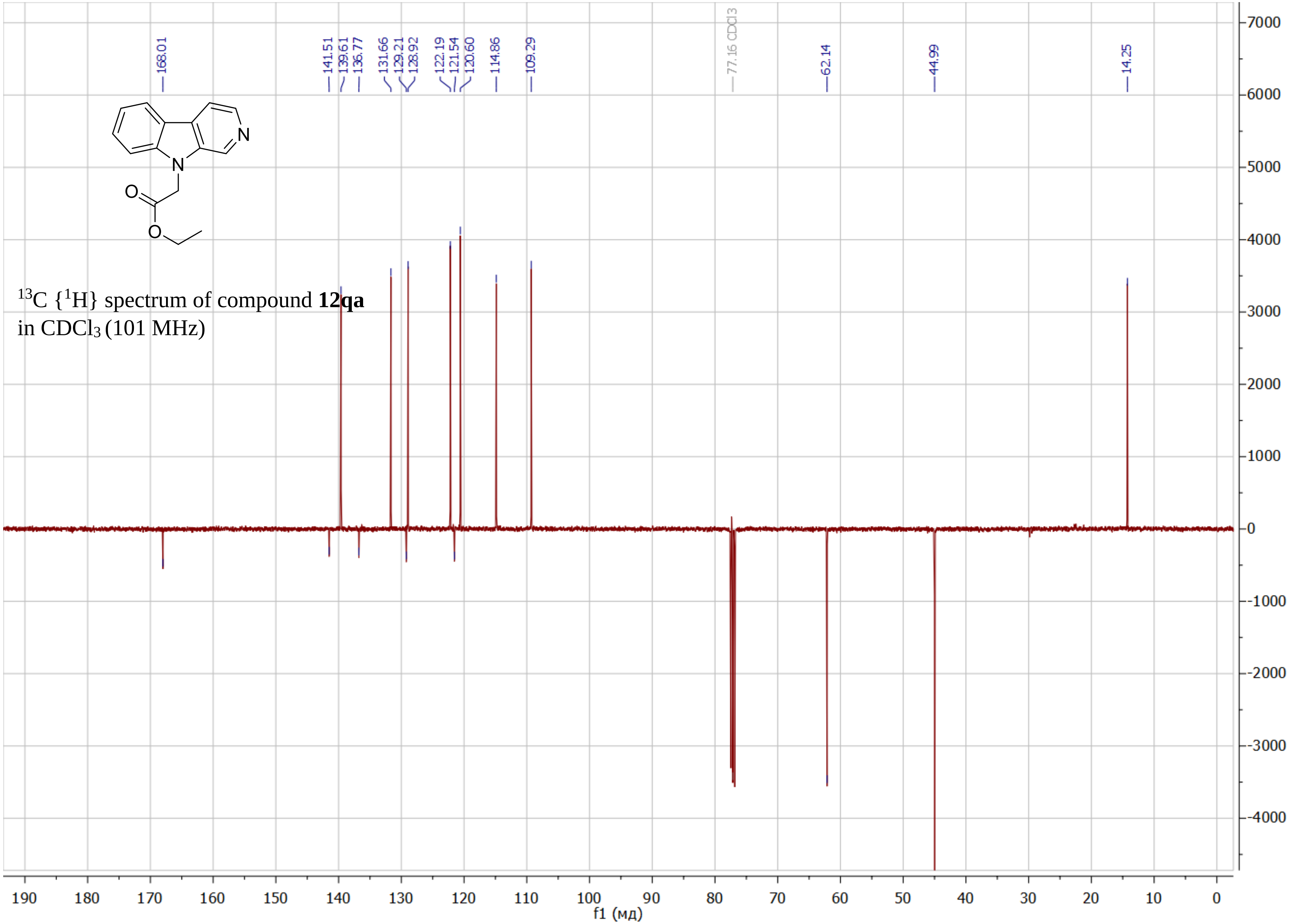

^{13}C {^{1}H} spectrum of compound **12qa** in $CDCl_3$ (101 MHz)

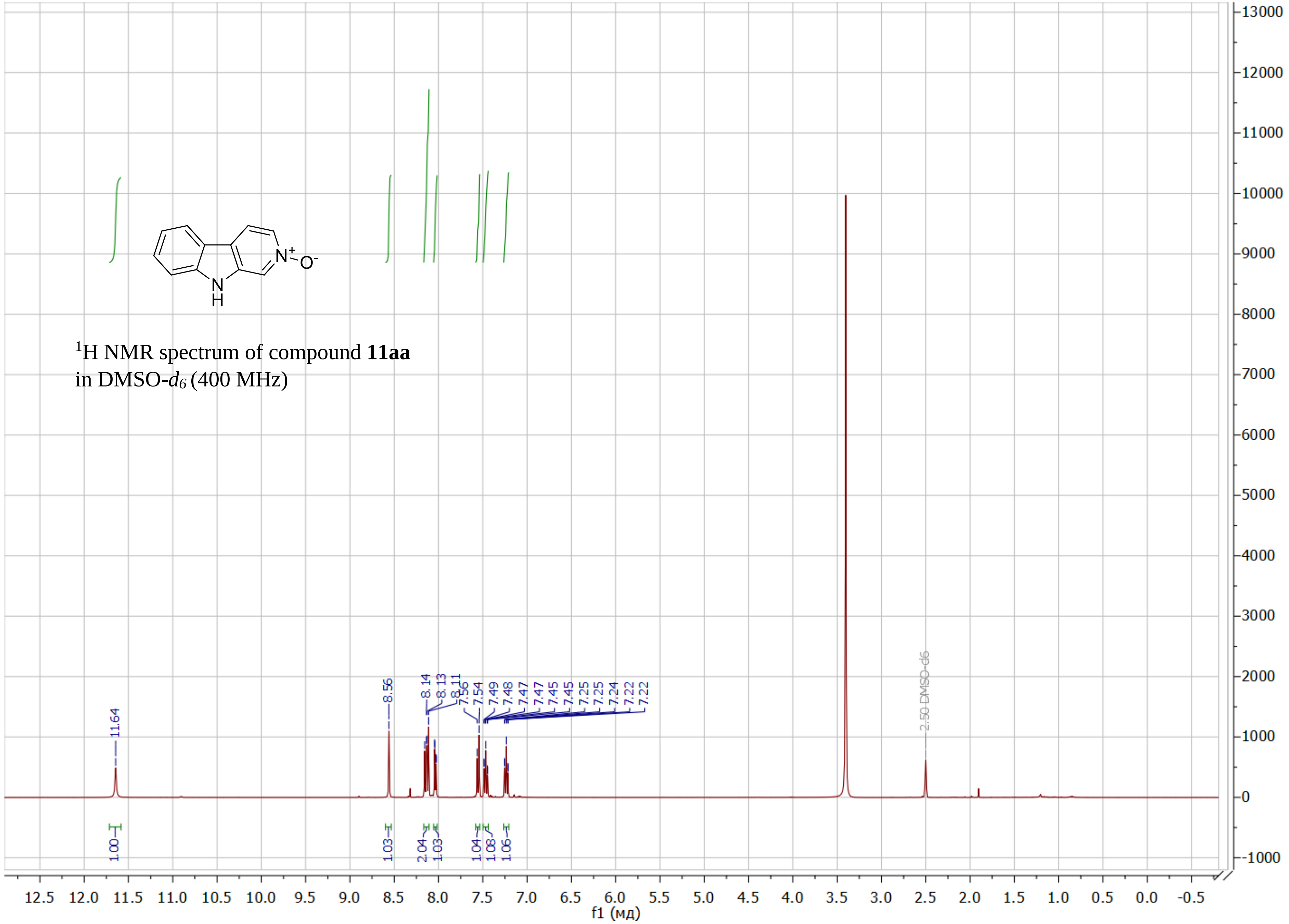

^{1}H NMR spectrum of compound **11aa** in DMSO-d_6 (400 MHz)

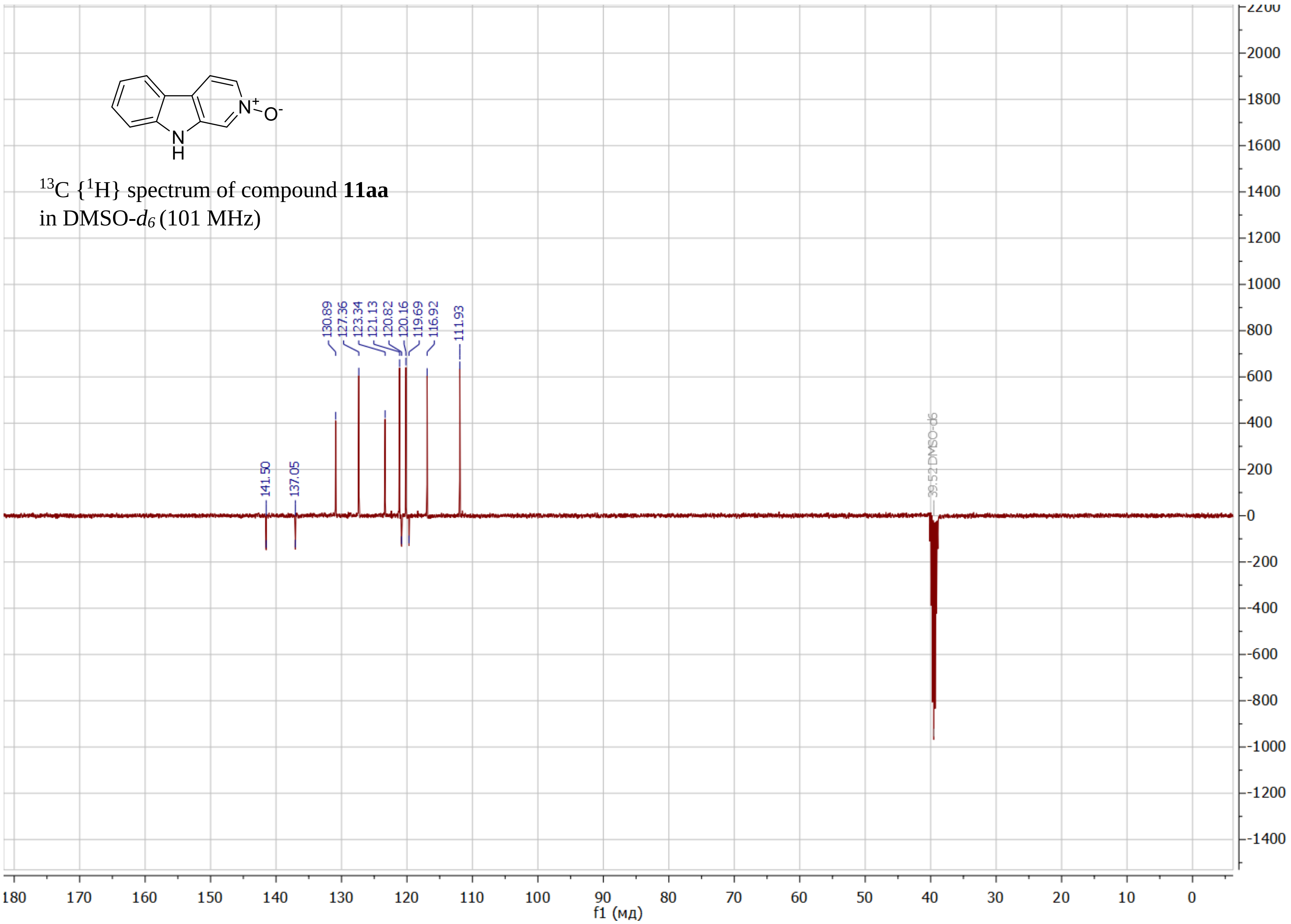

$^{13}C\ \{^{1}H\}$ spectrum of compound **11aa** in DMSO-d_6 (101 MHz)

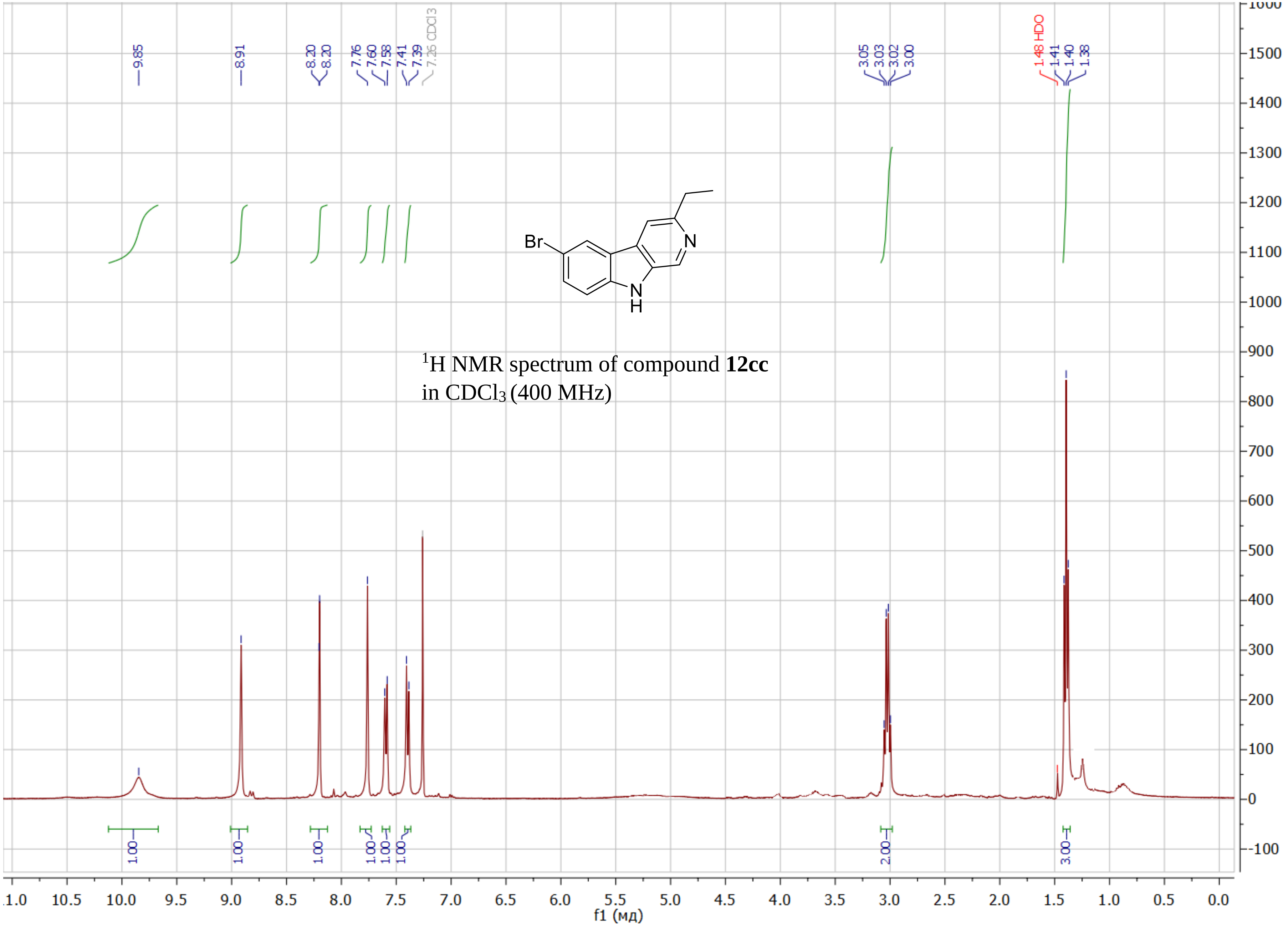

^{1}H NMR spectrum of compound **12cc** in $CDCl_3$ (400 MHz)

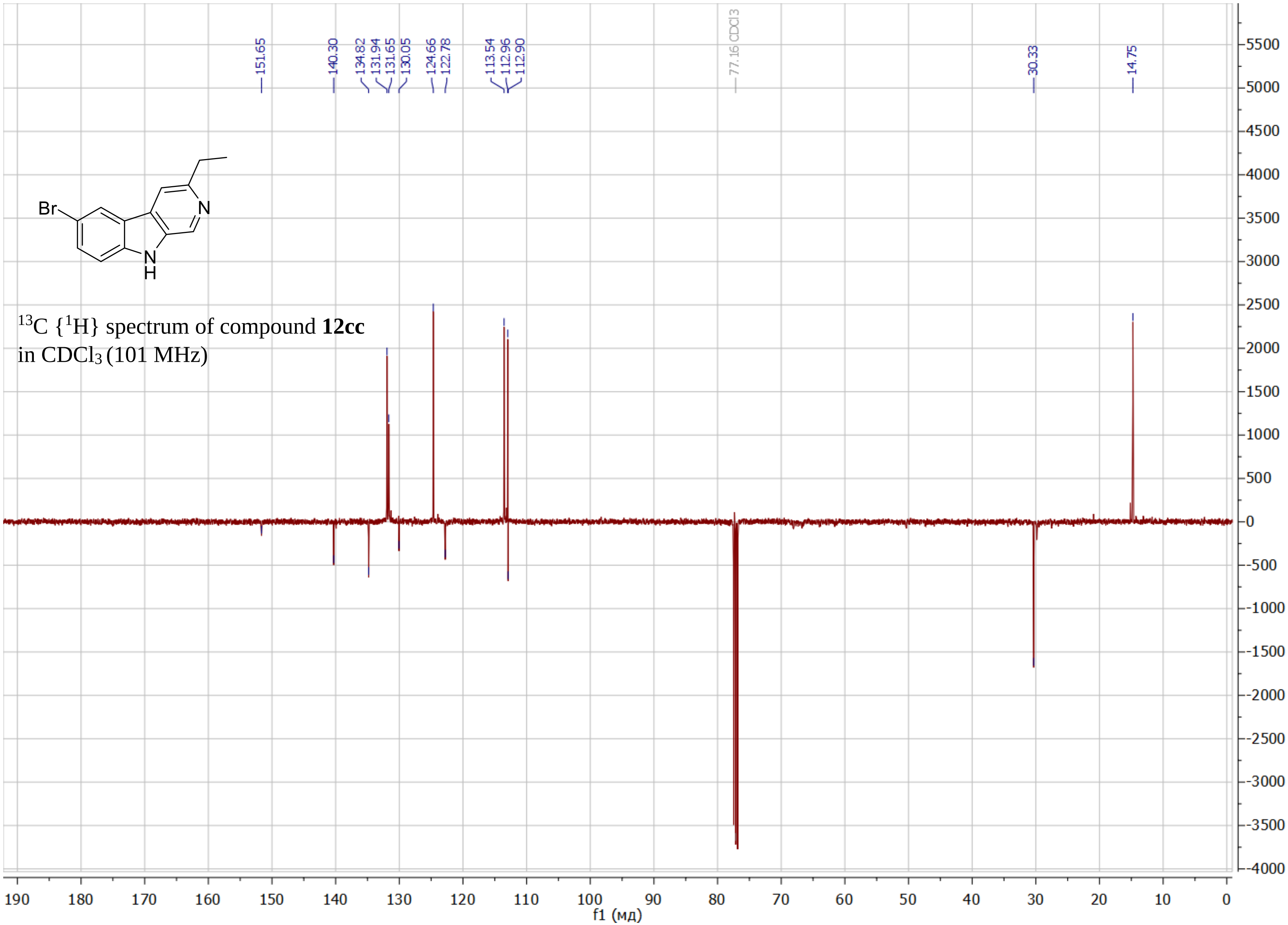

^{13}C {^{1}H} spectrum of compound **12cc** in $CDCl_3$ (101 MHz)

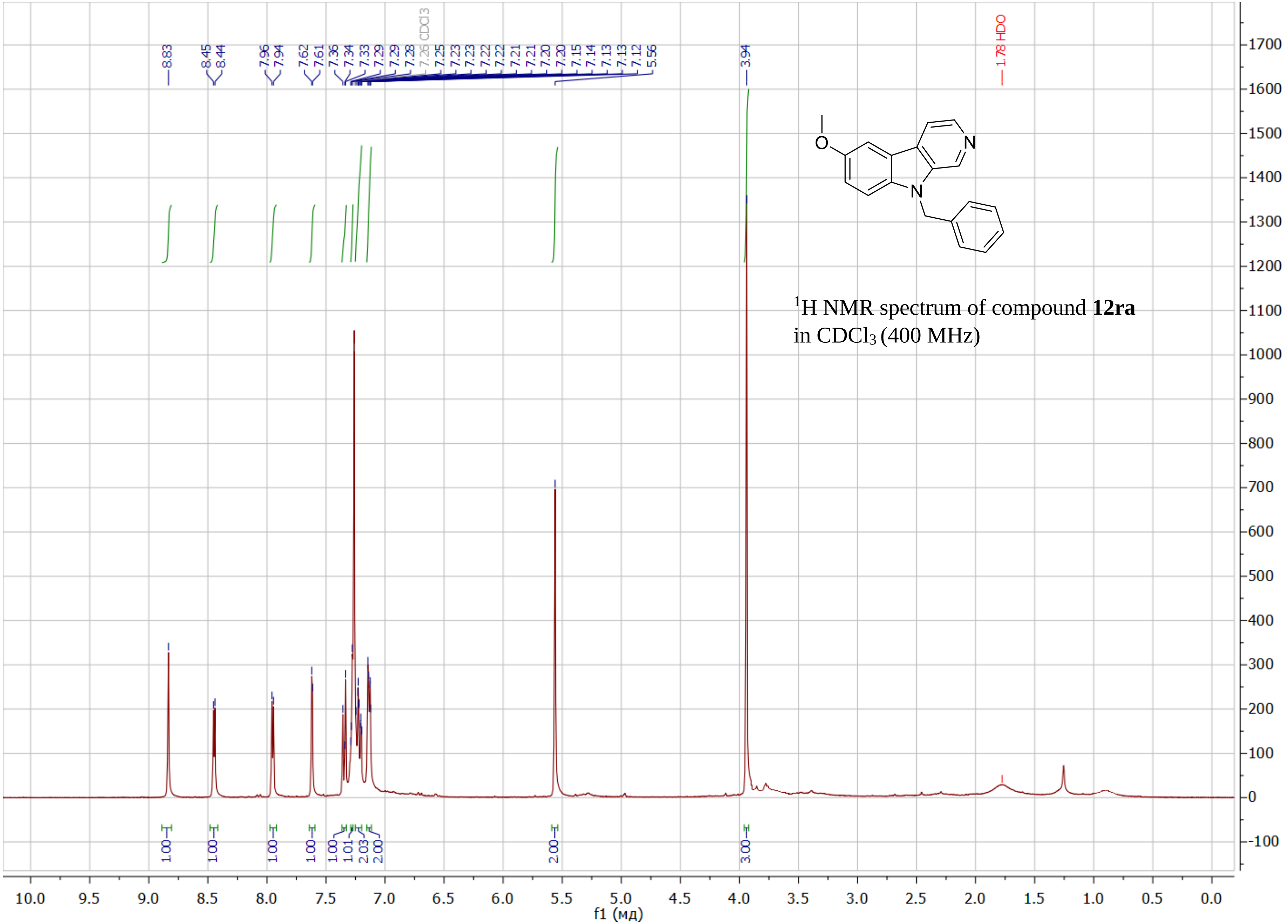

^{1}H NMR spectrum of compound **12ra** in $CDCl_3$ (400 MHz)

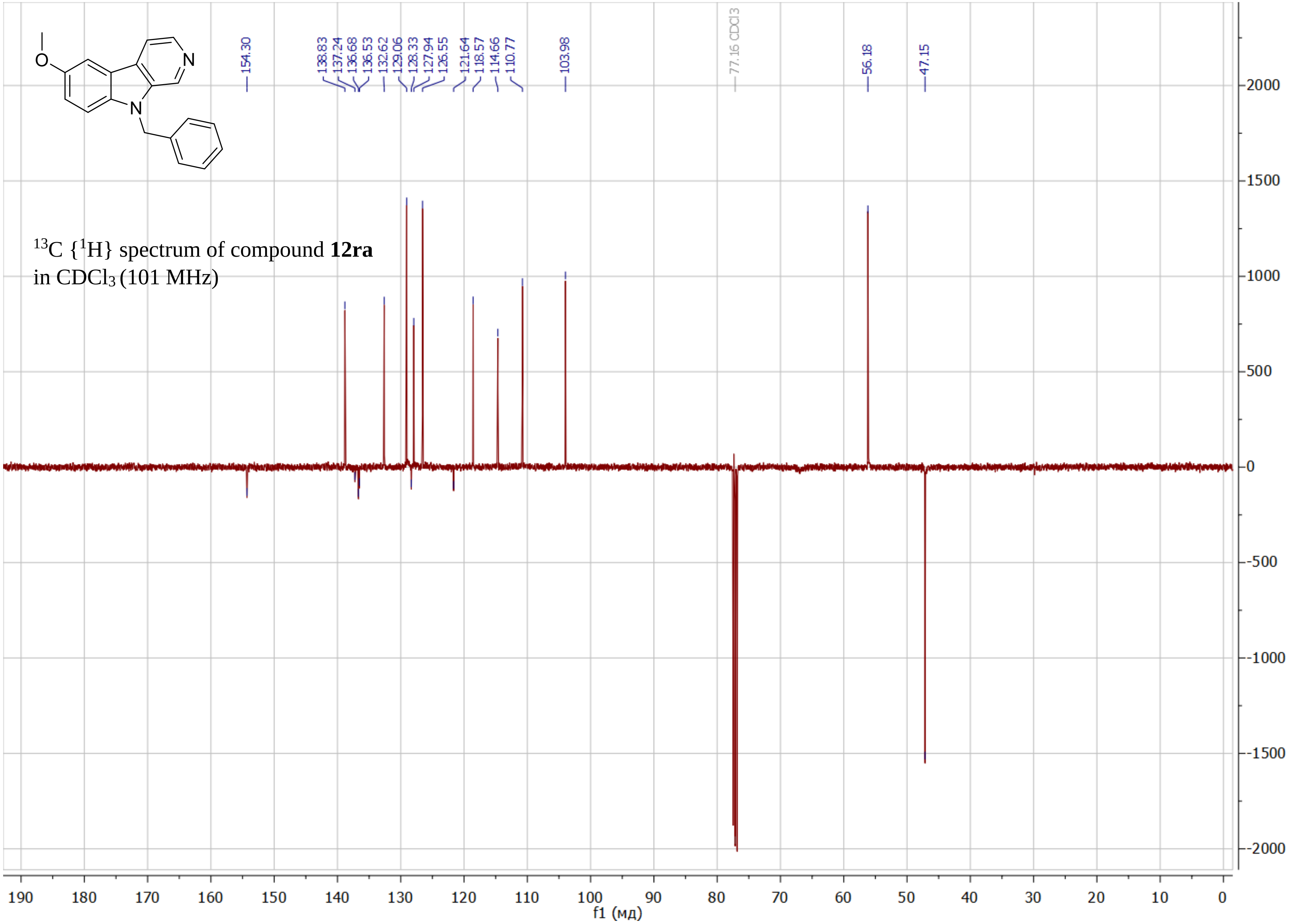

^{13}C {^{1}H} spectrum of compound **12ra** in $CDCl_3$ (101 MHz)

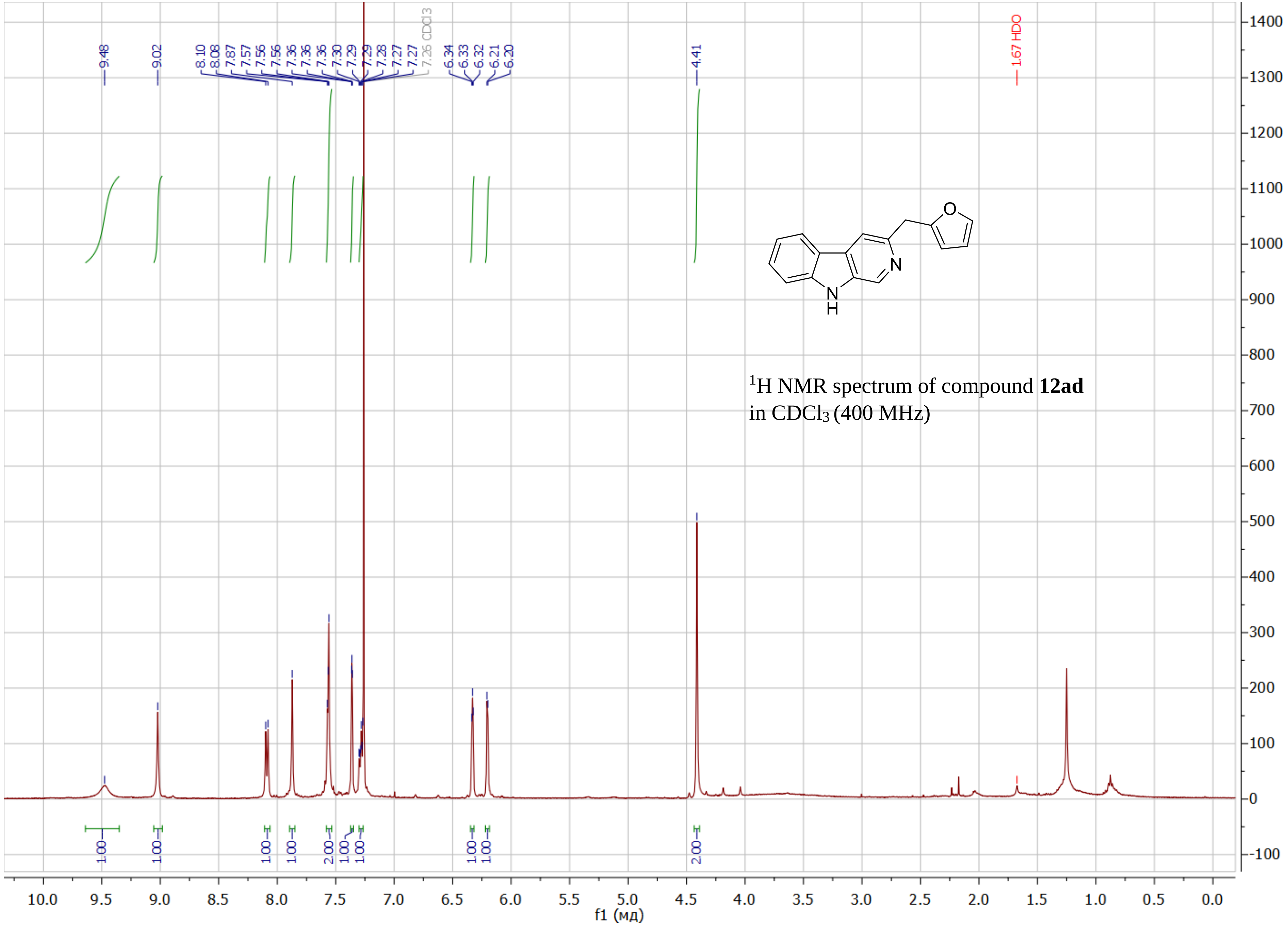

^{1}H NMR spectrum of compound **12ad**
in $CDCl_3$ (400 MHz)

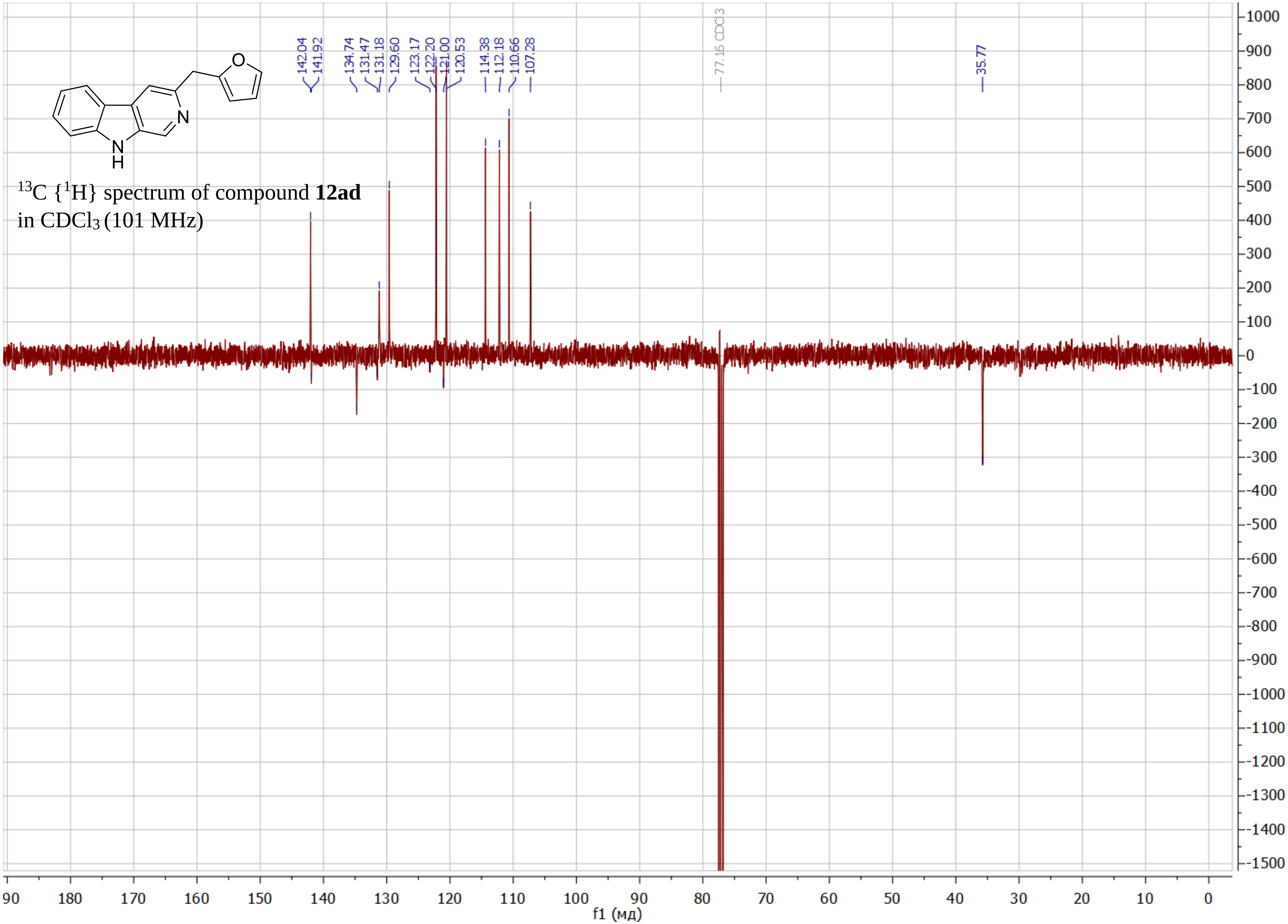

^{13}C {^{1}H} spectrum of compound **12ad** in $CDCl_3$ (101 MHz)

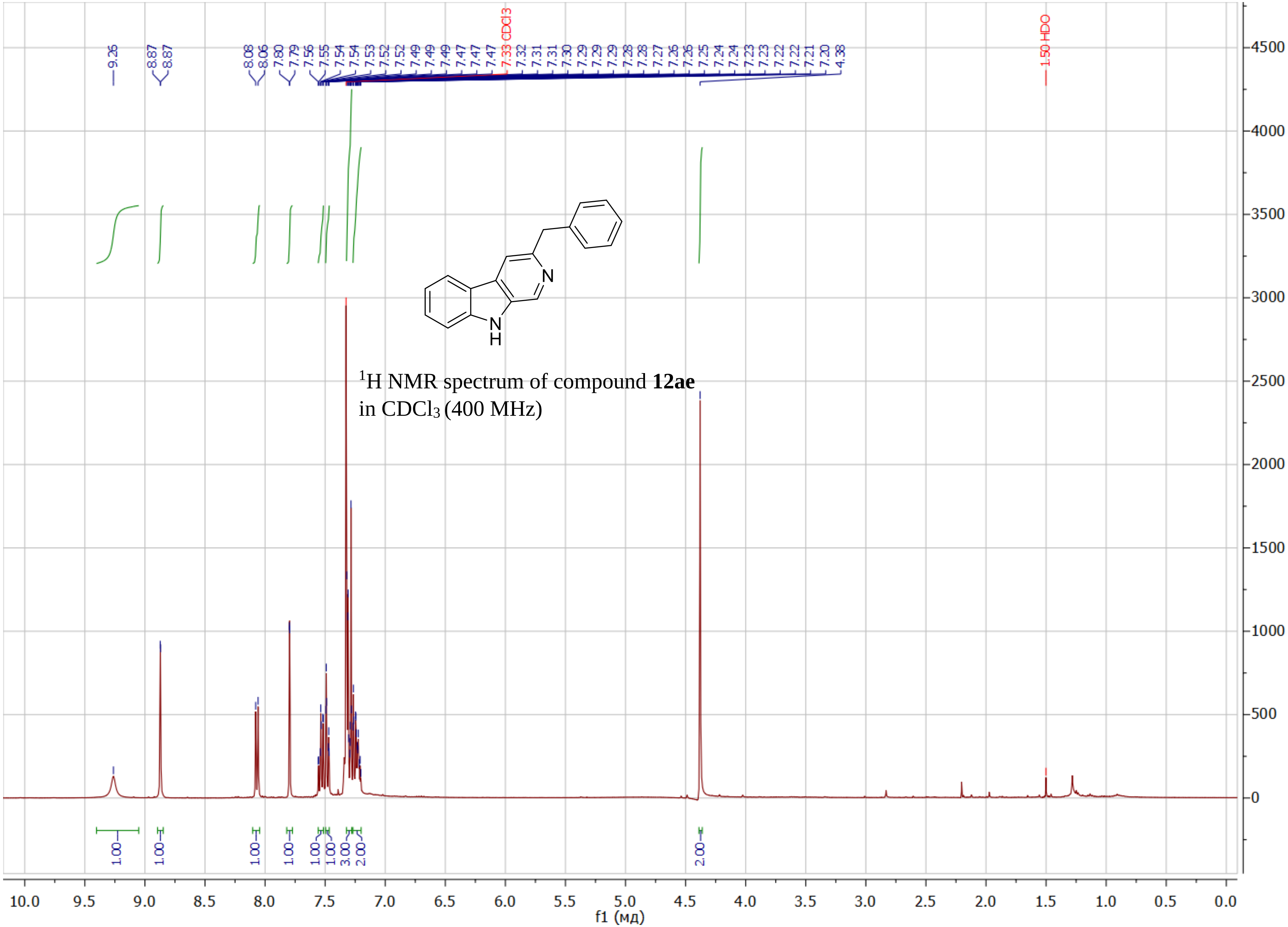

^{1}H NMR spectrum of compound **12ae** in $CDCl_3$ (400 MHz)

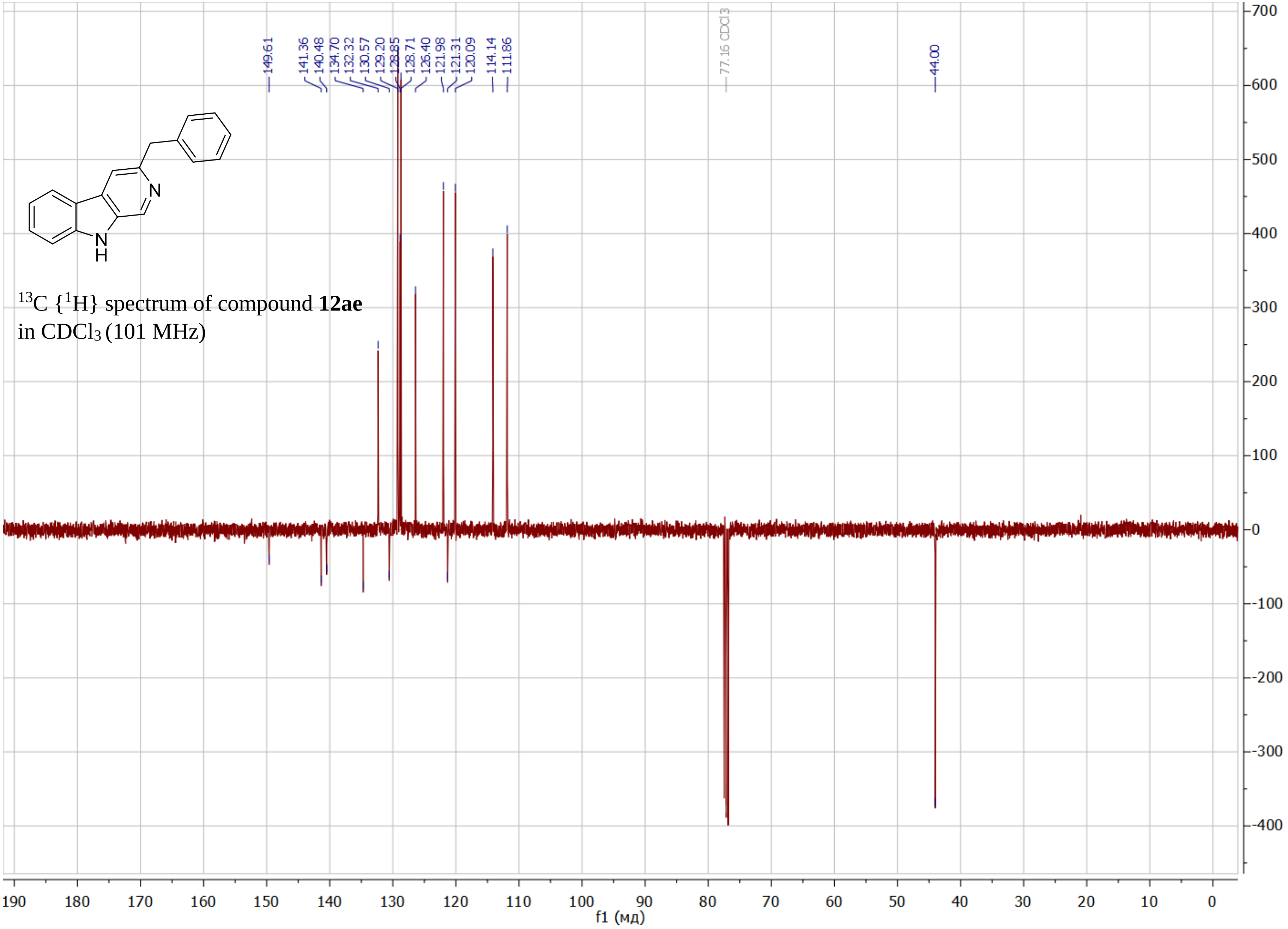

^{13}C {^{1}H} spectrum of compound **12ae** in $CDCl_3$ (101 MHz)

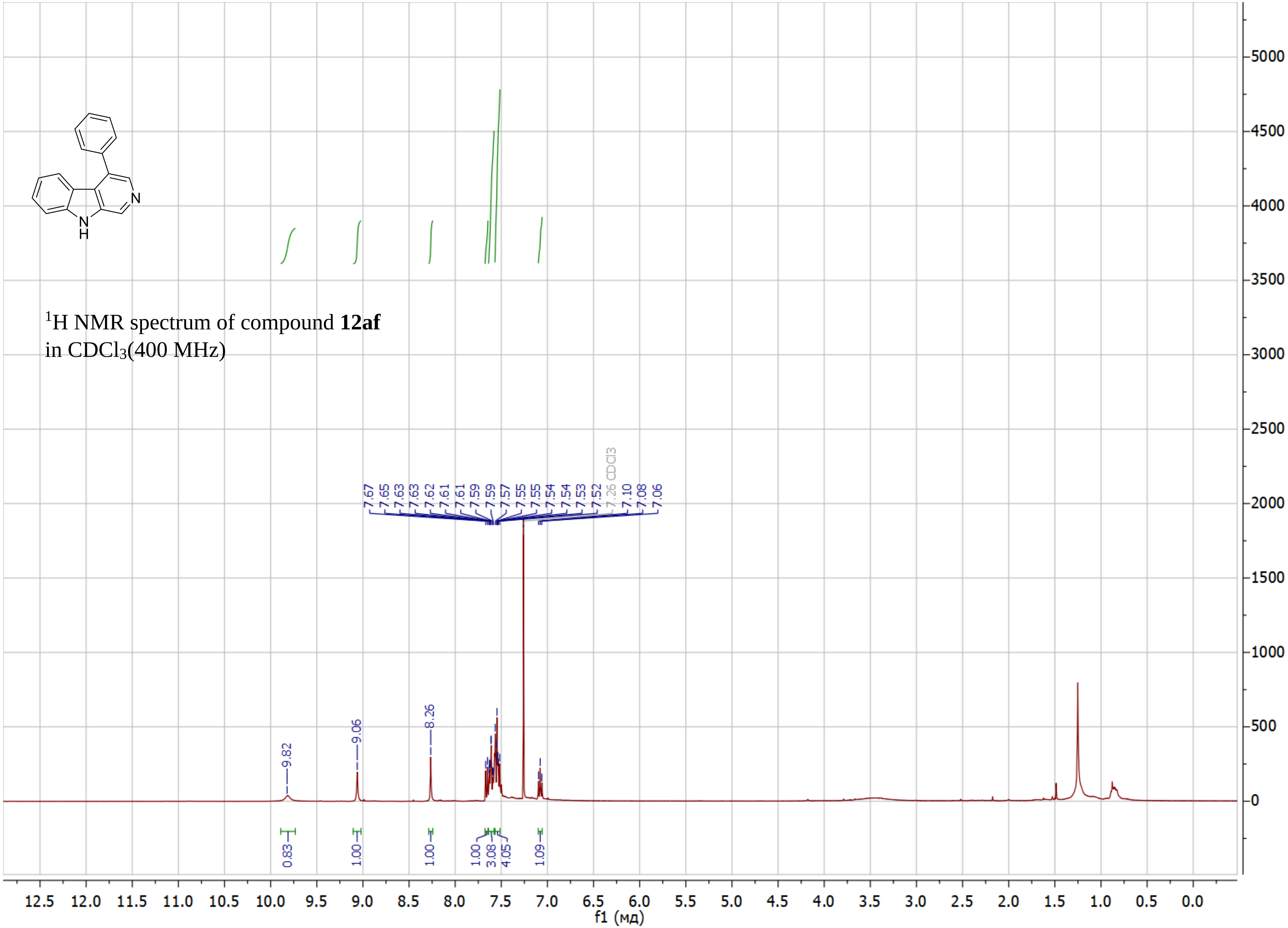

^{1}H NMR spectrum of compound **12af** in $CDCl_3$(400 MHz)

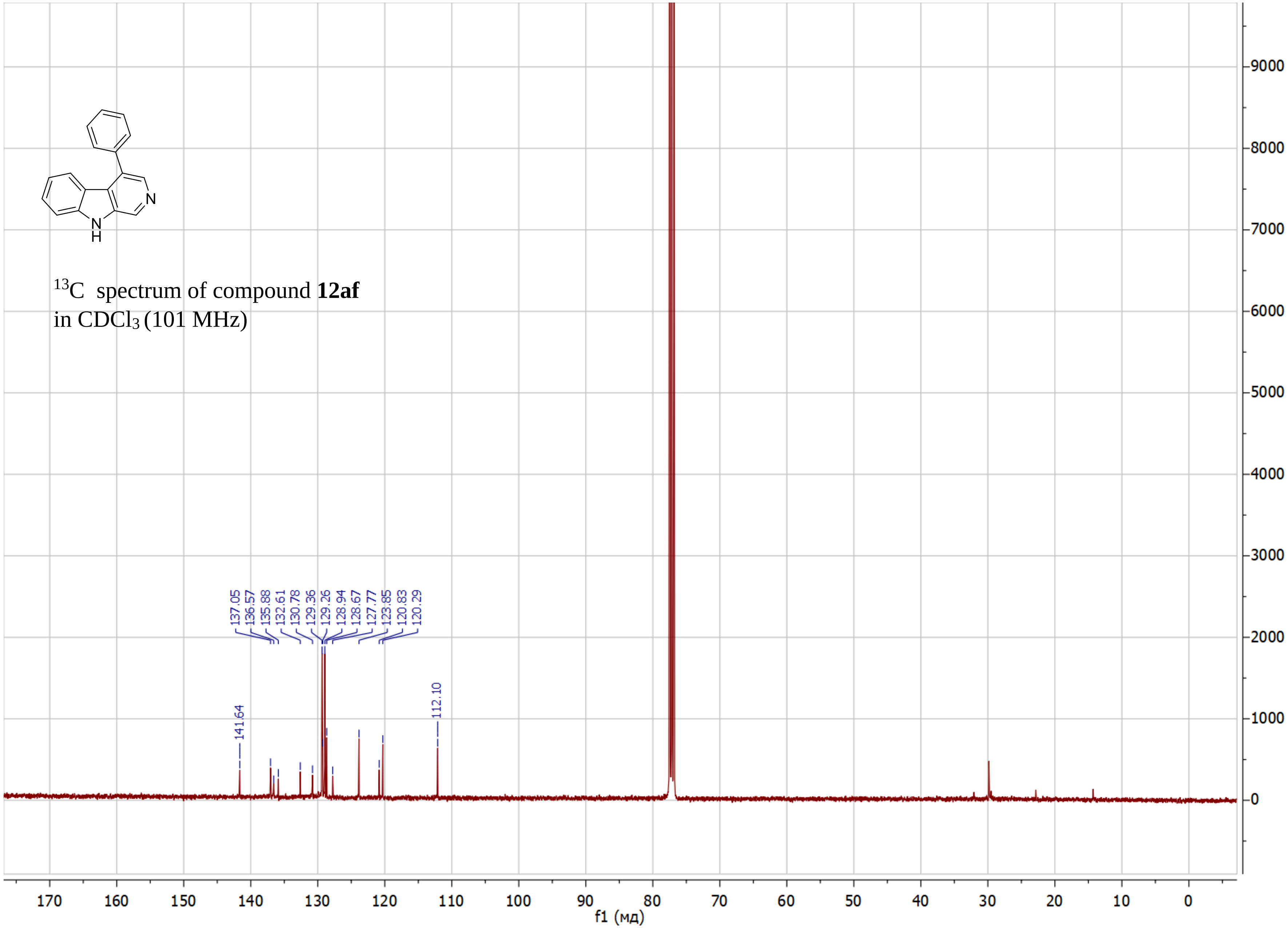

^{13}C spectrum of compound **12af** in $CDCl_3$ (101 MHz)

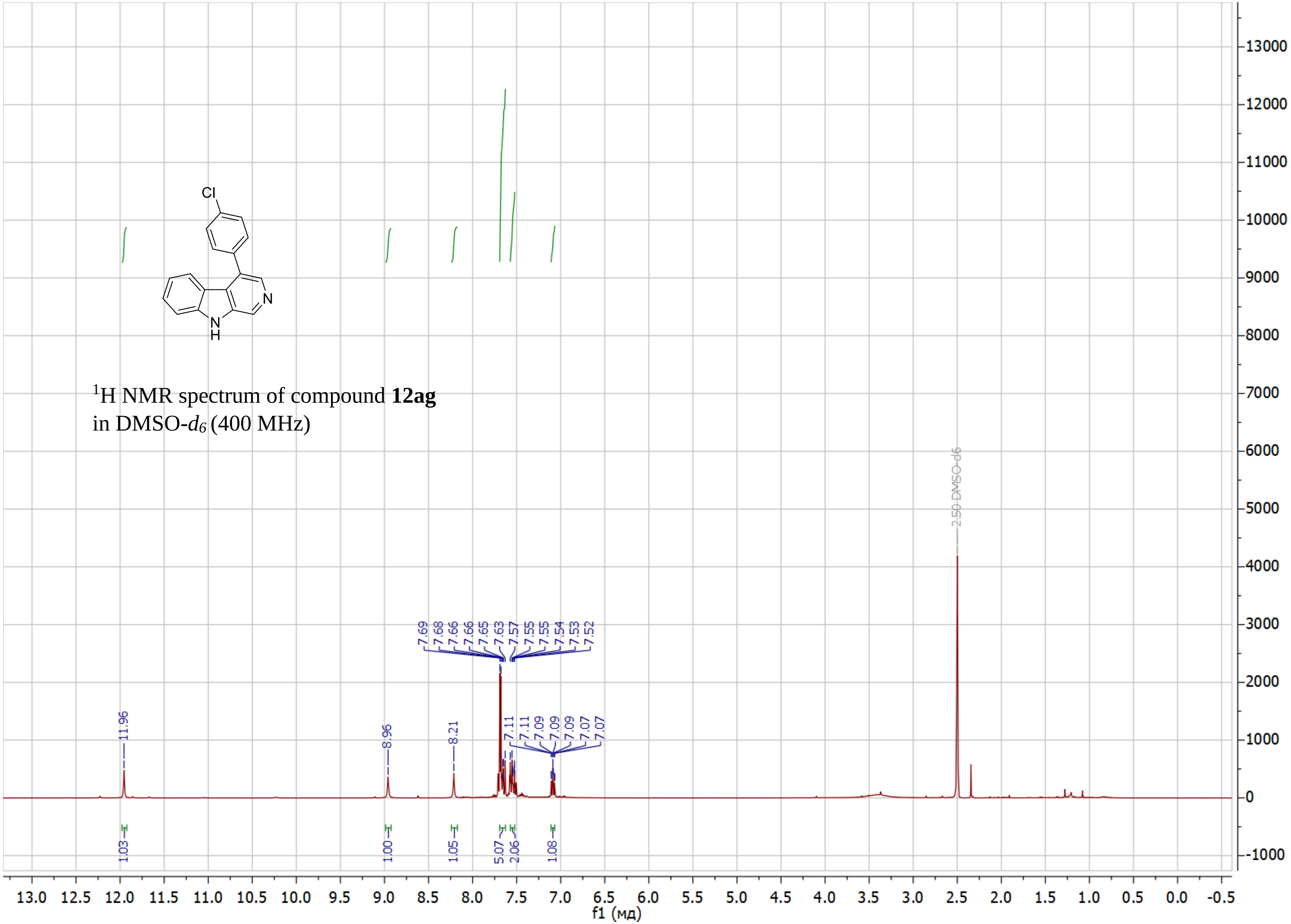

^{1}H NMR spectrum of compound **12ag** in DMSO-d_6 (400 MHz)

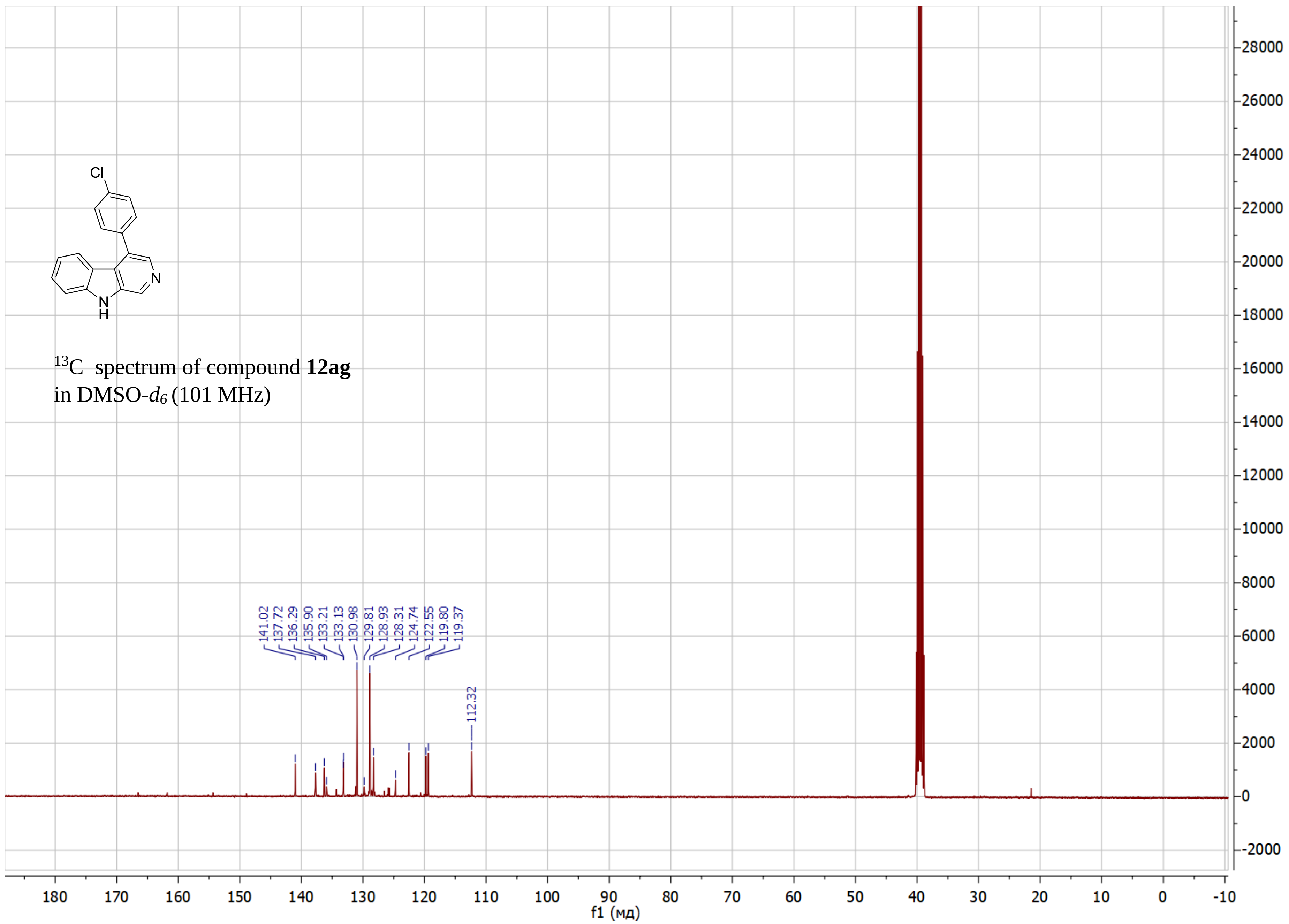

^{13}C spectrum of compound **12ag** in DMSO-d_6 (101 MHz)

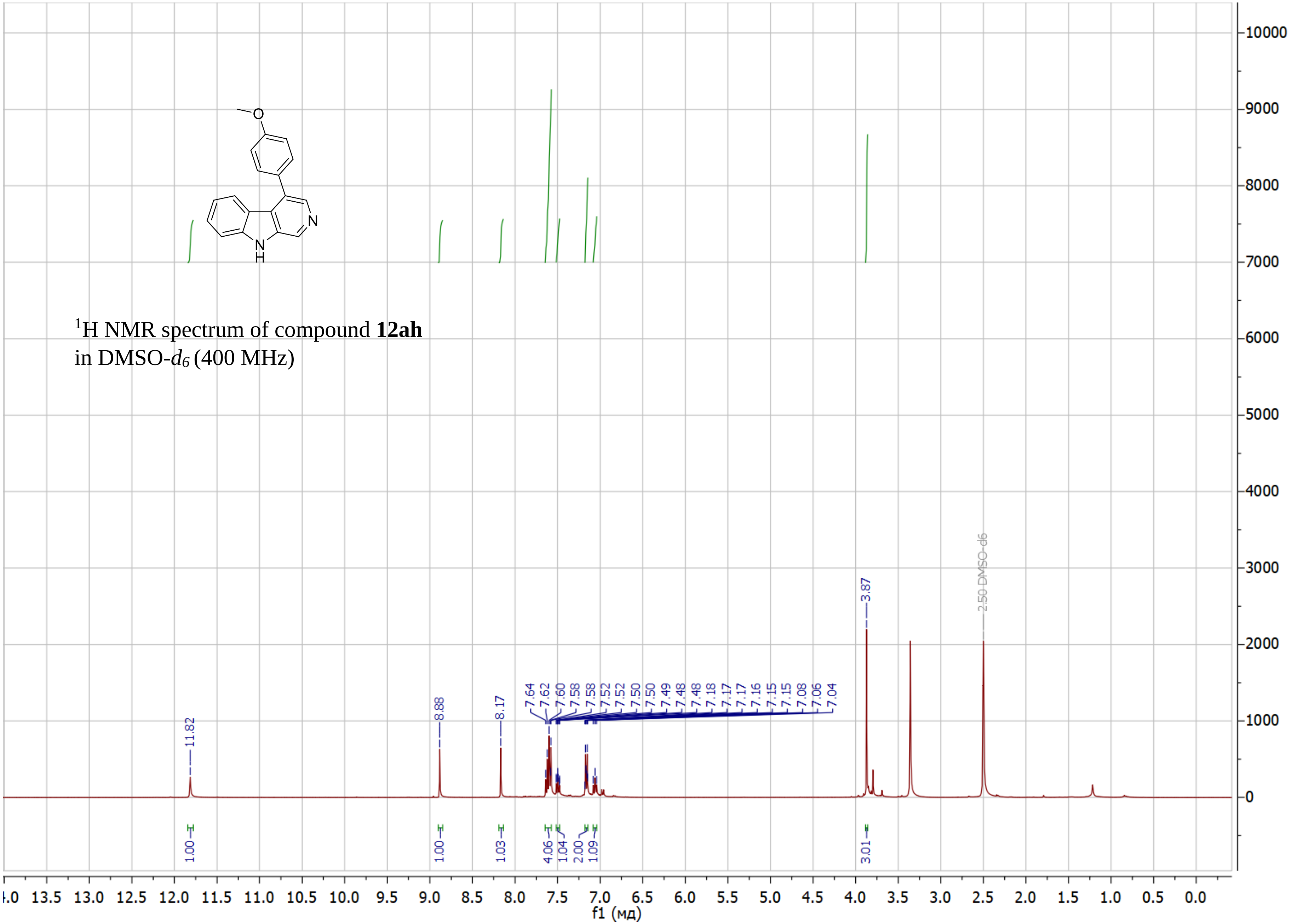

^{1}H NMR spectrum of compound **12ah** in DMSO-d_6 (400 MHz)

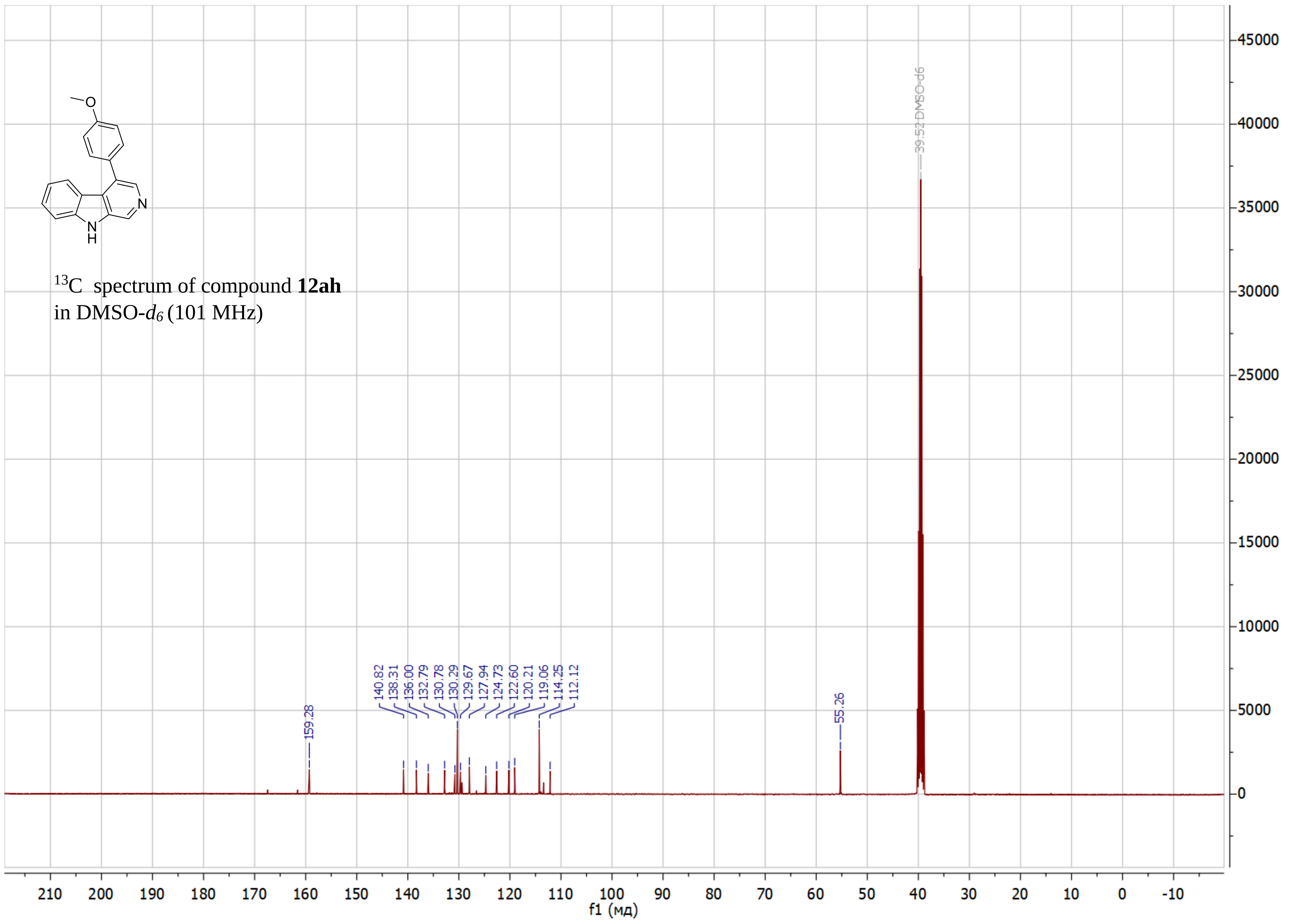

O
N
N
H
13C spectrum of compound 12ah
in DMSO-d6 (101 MHz)
159.28
140.82
138.31
136.00
132.79
130.78
130.29
129.67
127.94
124.73
122.60
120.21
119.06
114.25
112.12
55.26
39.52 DMSO-d6
45000
40000
35000
30000
25000
20000
15000
10000
5000
0
210 200 190 180 170 160 150 140 130 120 110 100 90 80 70 60 50 40 30 20 10 0 -10
f1 (мд)
